# Supplementary material for: CHK1 controls zygote pronuclear envelope breakdown by regulating F-actin through interacting with MICAL3
Source: EMBO Rep. 2024 Oct 2;25(11):4876–97. doi: 10.1038/s44319-024-00267-7 (PMC11549291; doi:10.1038/s44319-024-00267-7)
Supplement: Supplementary file 1 — Appendix [file 44319_2024_267_MOESM1_ESM.docx]

**CHK1 Controls Zygote Pronuclear Envelope Breakdown by Regulating F-actin through Interacting with MICAL3**

***Appendix***

Table of contents:

Appendix Figure S1———————————— 2

Appendix Figure S2———————————— 3

Appendix Figure S3———————————— 4

Appendix Figure S4———————————— 6

Appendix Figure S5———————————— 7

Appendix Table S1—————————————8

Appendix Table S2—————————————9

**
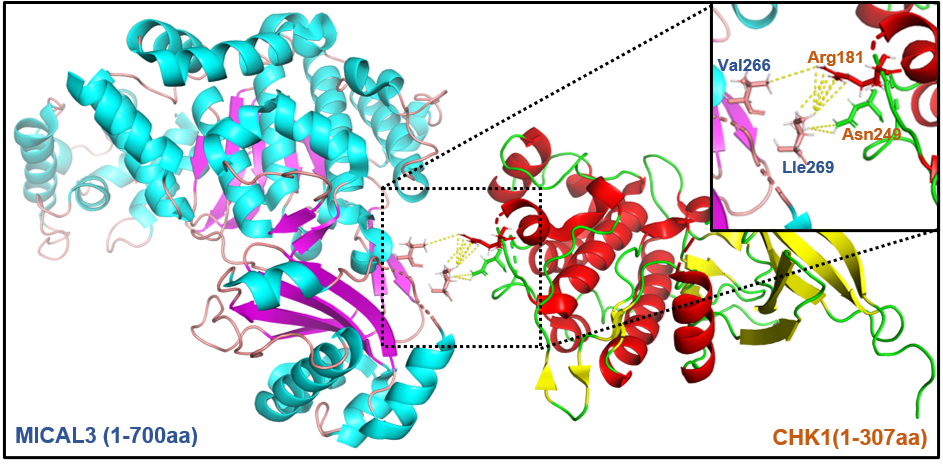
**

**Appendix Figure S1. Interaction analysis between MICAL3 and CHK1.** Crystal structure exhibition of first 700 amino acid residues of MICAL3 (left, pdb6ici) and first 307 amino acid residues of CHK1 (right, pdb2qhn). The yellow dotted lines between MICAL3 (Val266 and Lle269) and CHK1 (Arg181 and Asn249) represent non-polar interactions analyzed by PyMOL software.

**
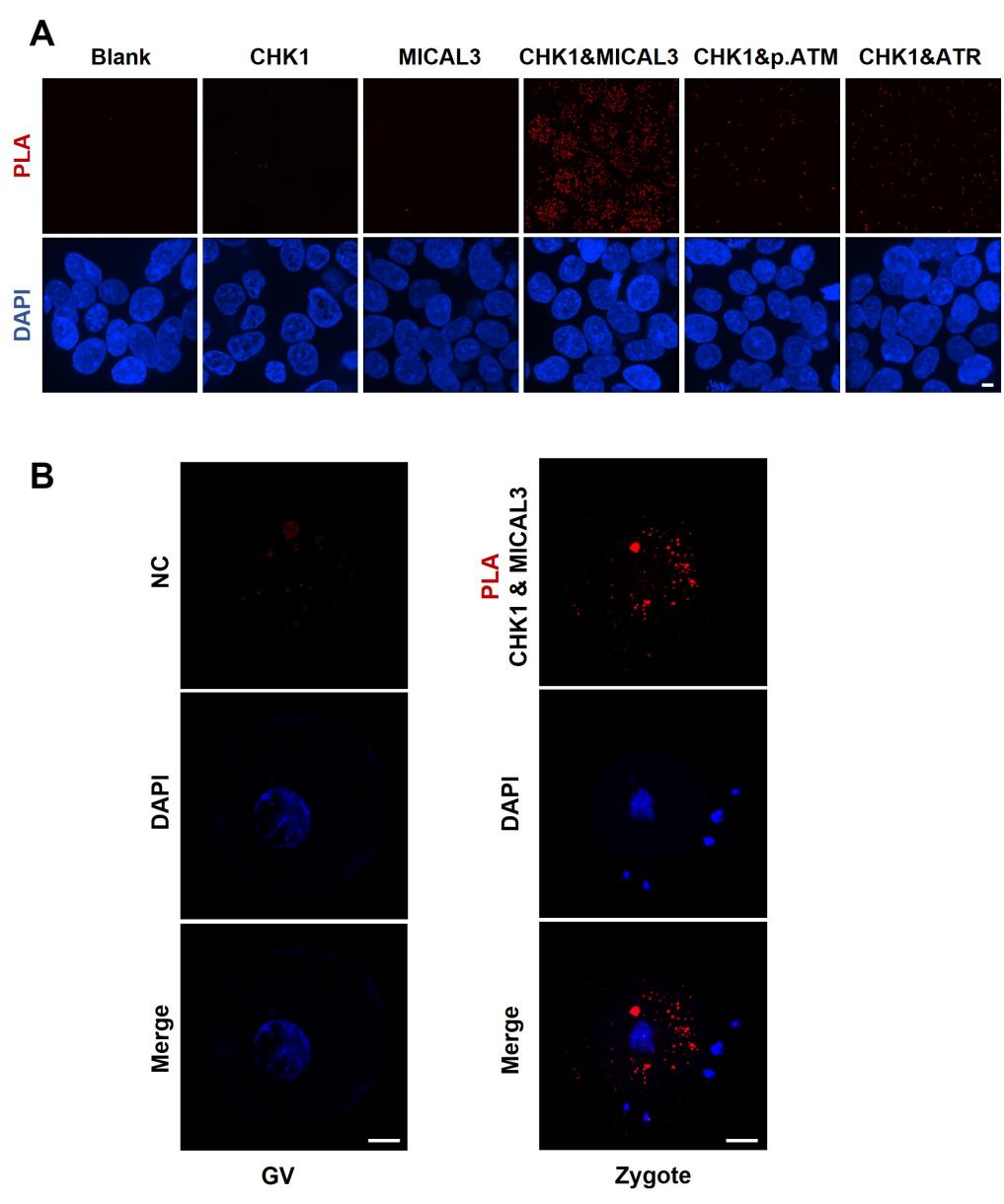
**

**Appendix Figure S2. Interaction analysis between MICAL3 and CHK1 by PLA.** (A) Red PLA signals in HEK-293T cells indicate the interactions between CHK1 and MICAL3. The signals between CHK1 and p.ATM or ATR were used as positive controls. The z-scan model were applied to collect signals through the confocal microscope. Scale bar: 5 µm. (B) PLA analysis of a human 3PN zygote displays significant red PLA signals. The human GV-stage oocyte without primary antibodies is regarded as normal control (NC). GV, germinal vesicle; PN, pronuclei. Scale bar: 20 µm.

**
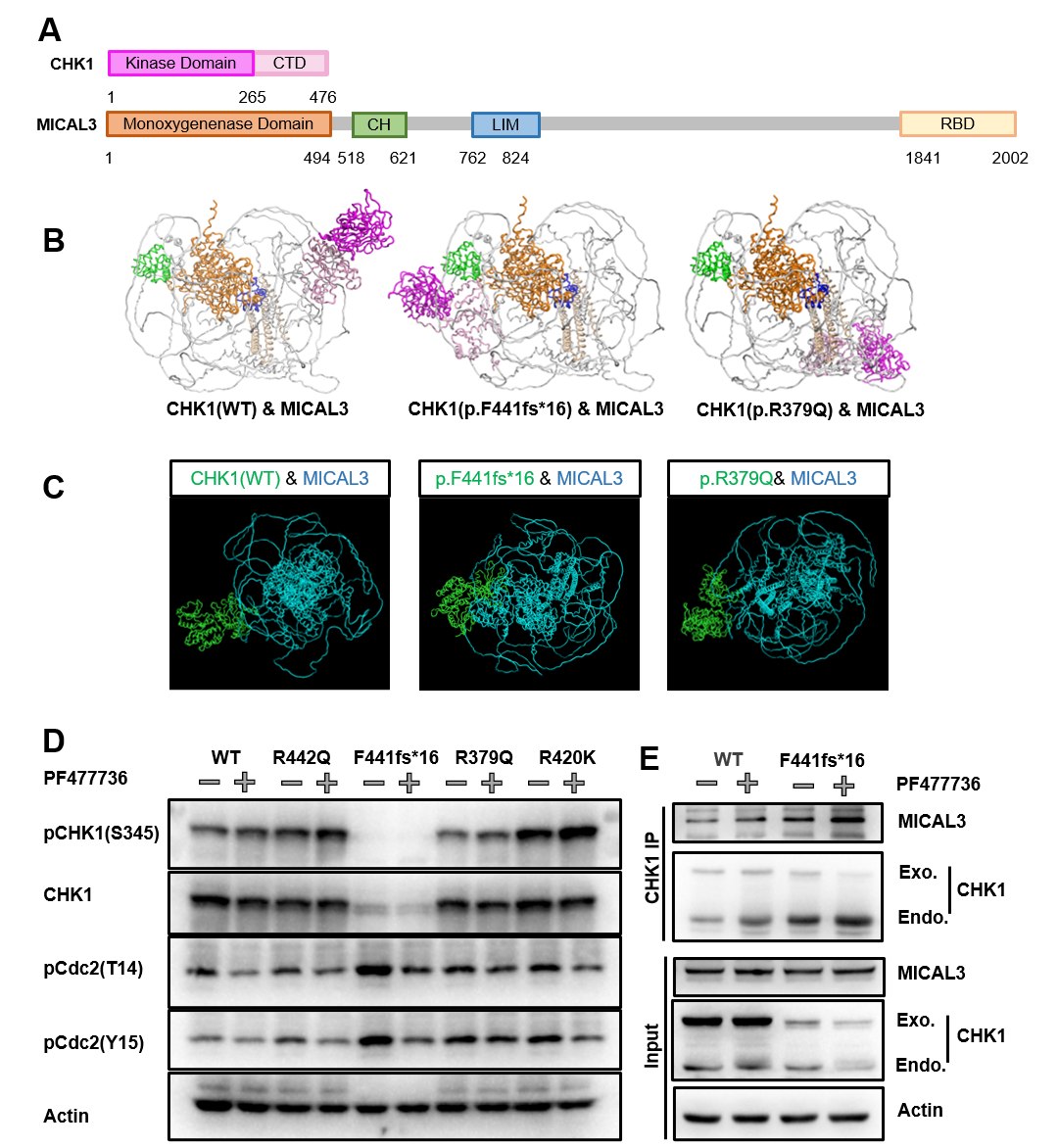
**

**Appendix Figure S3. The protein-protein docking results and the impact of PF477736 treatment on interaction between CHK1s and MICAL3.** (A) Domains in CHK1 and MICAL3 are highlighted with different colors: CTD (C-terminal domain), CH (calponin homology domain), LIM (Lin-11, Isl-1, and Mec-3 domain), and RBD (Rab-binding domain). **(B)** Protein-protein docking models of CHK1s and MICAL3 are color-coded according to their domains, as illustrated in (A). These models were generated using the GRAMM-X public web server, with the 3D conformation of MICAL3 modeled by AlphaFold2 and CHK1s conformations constructed by RoseTTAFold due to the absence of full-length crystal structures. **(C)** Protein-protein docking models of CHK1s and MICAL3 are displayed in another way. **(D)** Western blot analysis of HEK-293 cells transfected with individual CHK1 C-terminal mutations. After transfection and subsequent treatment with or without PF477736, protein expression was assessed. **(E)** CO-IP analysis demonstrates the interaction between CHK1 and MICAL3, which is enhanced upon application of PF477736.

**
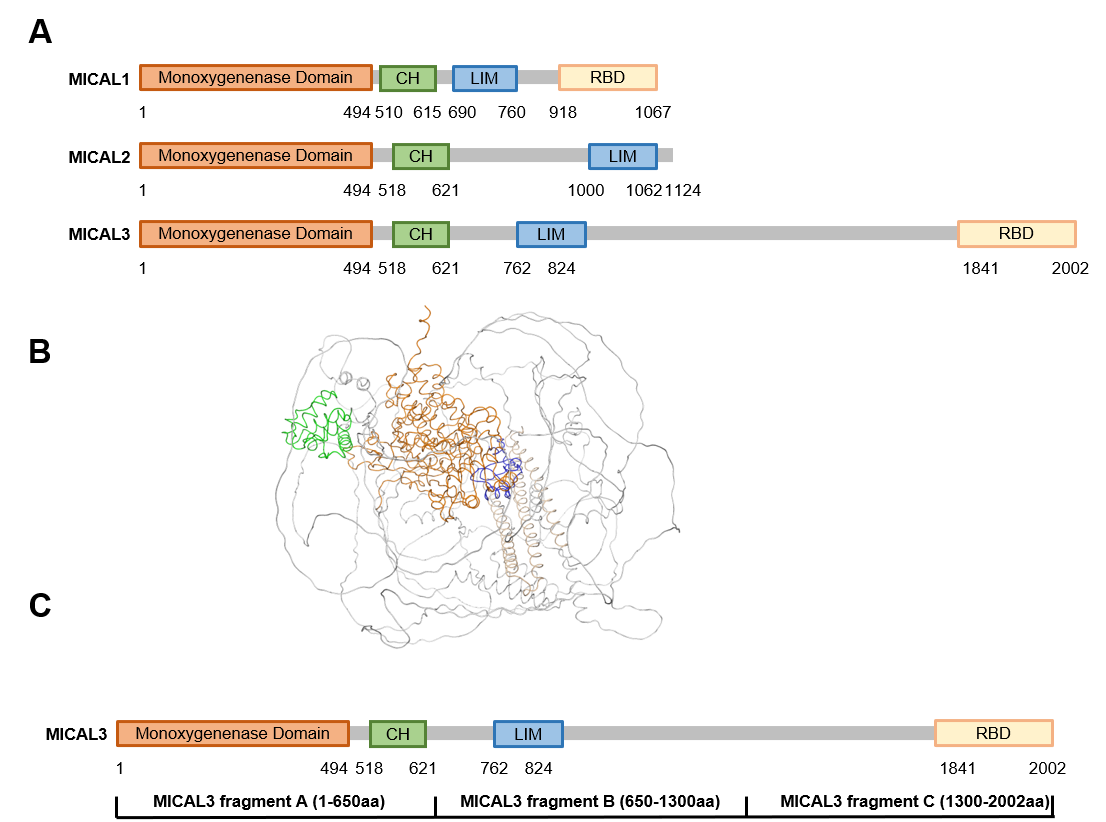
**

**Appendix Figure S4. The structure illustration of MICALs.** **(A)** The illustration depicts the structure of human MICAL family members, highlighting their distinctive domains. The N-terminal flavoprotein monooxygenase domain plays a pivotal role in the actin-depolymerizing activity of MICAL proteins. The calponin homology (CH) domain is a characteristic actin-binding region. Additionally, the LIM domain, found in Lin-11, Isl-1, and Mec-3 proteins, and the motifs of the Rab-binding domain (RBD) are also shown. **(B)** A 3D conformation of MICAL3, constructed using AlphaFold2, is displayed. The different domains are color-coded as (A). **(C)** The architecture of MICAL3 fragments, which were subject to purification attempts, is shown.

**
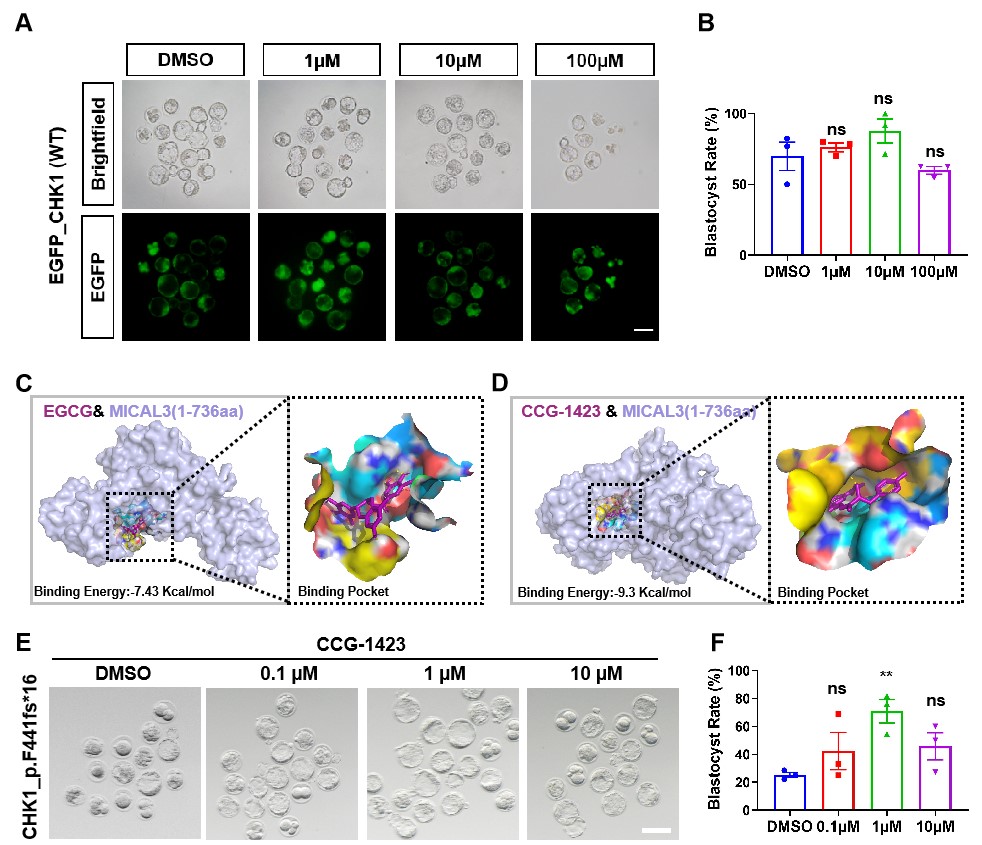
**

**Appendix Figure S5. CCG-1423 treatment can promote the development of zygotes carrying mutant CHK1. (A)** Figures show wild-type (WT) blastocyst development result under the treatment of EGCG at different concentrations. Scale bars: 100 µm. **(B)** Quantification of blastocyst development rates in (A). **(C-D)** Demonstration of the binding pocket between MICAL3 and EGCG (C) or CCG-1423 (D). The yellow dashed lines indicate the polar contacts between the inhibitor and the binding pocket of MICAL3 (PDB:6ICI). **(E)** CCG-1423 could replicate the effect of EGCG under 1 µM treatment. Scale bar: 100 µm. **(F)** Quantification of blastocyst development rates in (E). Two-tailed Student’s t-tests. ns, no significant difference. **P < 0.01. Error bar, SEM.

**Appendix Table S1:** **Embryo development record for pronuclear exchange between the patient carrying *CHK1* mutation (R379Q) and donors (WT).**

|  | **Patient’s pronuclear &**  **donor’s cytoplasm** | | | | **Donor’s pronuclear &**  **patient’s cytoplasm** | | |
| --- | --- | --- | --- | --- | --- | --- | --- |
| **Em ID** | Em-1 | Em-2 | Em-3 | Em-4 | Em-5 | Em-6 | Em-7 |
| **Zygote** | 2PN | 1PN | 2PN | 2PN | 2PN | 2PN | 2PN |
| **Em-D1** | 2C3' | 2C3' | 1C | 2C3' | UD | UD | UD |
| **Em-D2** | 4C4’ | 4C3’ | 2C2' | 4C4’ | UD | UD | UD |
| **Em-D3** | 8C4' | 6C3' | 2C3' | 8C3' | UD | UD | UD |
| **Em-D5/6** | 5AB | D | D | 4CC | 3C2' | UD | UD |
| a. “Em” represents embryo; “PN” represents pronuclear; “1C, 2C, 3C, 4C, 6C, 8C” represent 1-cell, 2-cell, 3-cell, 4-cell, 6-cell, 8-cell; “UD” represents undivided; “D” represents degenerated.  b. “2’, 3’, 4’” indicate the grading of early embryos in Day 1 to Day 3 according to the morphology and fragments of blastomeres and the higher the score, the greater the grade.  c. The blastocyst in Day5/6 is divided into 6 periods according to the formation of blastocele while the inner cell mass and trophoblast are rated by alphabetical order, A, B, C, respectively. “5AB” indicate the blastocyst is hatching with high-grade inner cell mass and trophoblast cells; “4CC” indicate the blastocyst is fully expansive with thinner zona pellucida, tinny inner cell mass and very sparse trophoblast cells. | | | | | | | |

| **Antibodies** | **Source** | **Identifier** | **Application** |
| --- | --- | --- | --- |
| OCT 3/4 Antibody | Santa Cruz | sc-5279 | IF |
| Sox2 (D6D9) Antibody | Cell Signaling Technology | 3579S | IF |
| SSEA4 Antibody | Abcam | ab16287 | IF |
| TRA-1-60 Antibody | Abcam | ab16288 | IF |
| TRA-1-81 Antibody | Santa Cruz | sc-21706 | IF |
| Anti-Chk1 Antibody [E250] | Abcam | ab32531 | CO-IP, WB |
| Chk1 Antibody (G-4) | Santa Cruz | ab32531 | IF, PLA |
| MICAL3 Antibody（3A6) | Santa Cruz | sc-517049 | WB |
| MICAL3 Antibody | Novus | nbp2-56826 | IF, PLA |
| Rhodamine Phalloidin | Thermo | R415 | IF |
| α-Tubulin Antibody | Sigma | T5168 | IF |
| Lamin B1 Antibody | Abcam | ab16048 | IF |
| Anti-CDX2 Antibody | Abcam | ab76541 | IF |
| GAPDH Antibody | Protein tech | 60004-1-Ig | WB |
| IgG Antibody | Abcam | ab199376 | CO-IP |
| WB, Western blot; IF, immunofluorescence; CO-IP, co-immunoprecipitation; PLA, proximity ligation assay | | | |

**Appendix Table S2: A list of antibodies used in this study.**
