# Supplementary figures and images for: CHK1 controls zygote pronuclear envelope breakdown by regulating F-actin through interacting with MICAL3 (part 1 of 2)
Source: EMBO Rep. 2024 Oct 2;25(11):4876–97. doi: 10.1038/s44319-024-00267-7 (PMC11549291; doi:10.1038/s44319-024-00267-7)

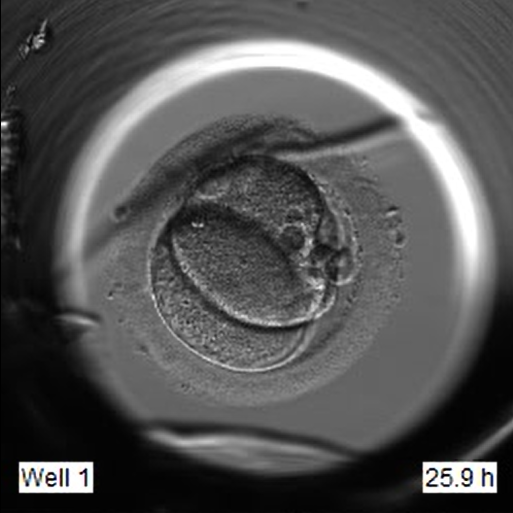

Supplement: Supplementary file 3 — Source data Fig. 1 [file 44319_2024_267_MOESM3_ESM.zip › Figure 1/1A/Em-1/D1 2cell 25.9h.png]

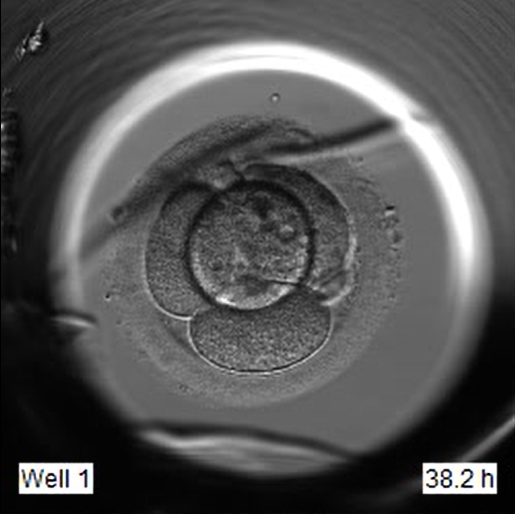

Supplement: Supplementary file 3 — Source data Fig. 1 [file 44319_2024_267_MOESM3_ESM.zip › Figure 1/1A/Em-1/D2 4cell 38.2h.png]

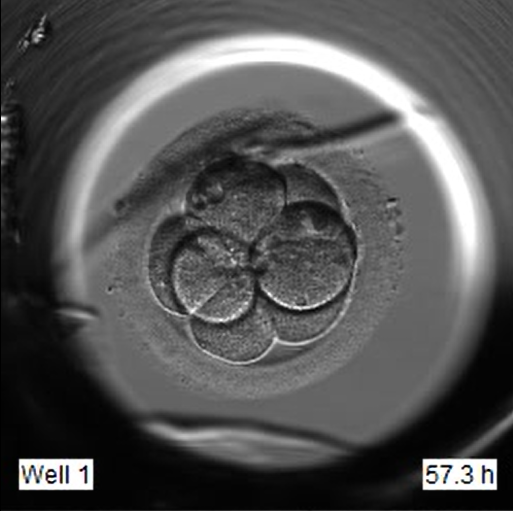

Supplement: Supplementary file 3 — Source data Fig. 1 [file 44319_2024_267_MOESM3_ESM.zip › Figure 1/1A/Em-1/D3 8cell 57.3h.png]

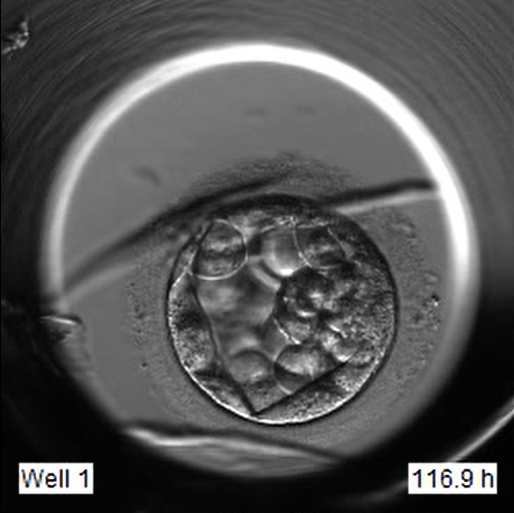

Supplement: Supplementary file 3 — Source data Fig. 1 [file 44319_2024_267_MOESM3_ESM.zip › Figure 1/1A/Em-1/D5 BC 116.9h.png]

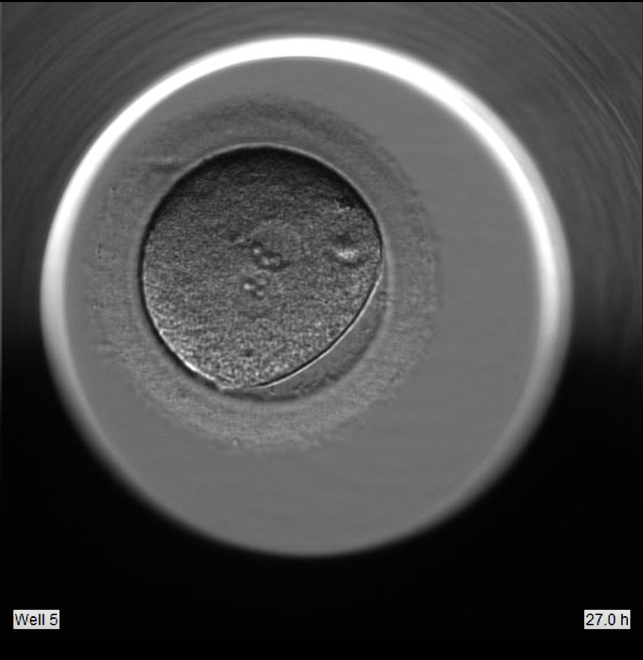

Supplement: Supplementary file 3 — Source data Fig. 1 [file 44319_2024_267_MOESM3_ESM.zip › Figure 1/1A/Em-6/D1 27h.png]

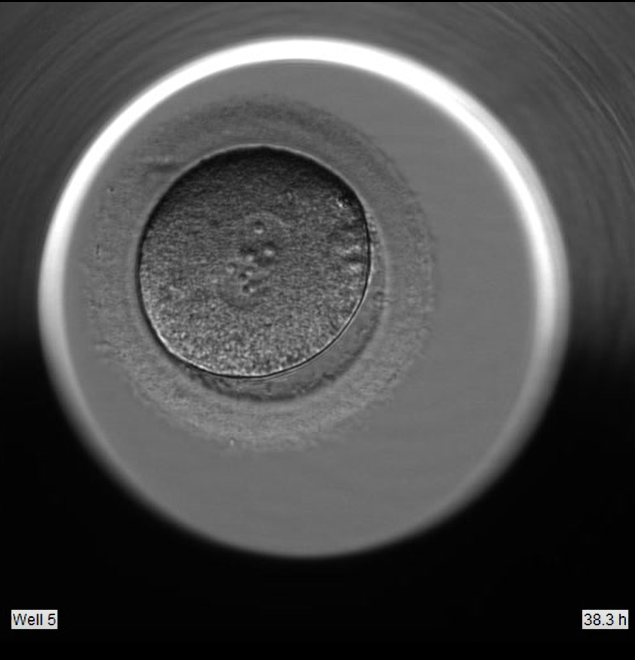

Supplement: Supplementary file 3 — Source data Fig. 1 [file 44319_2024_267_MOESM3_ESM.zip › Figure 1/1A/Em-6/D2 38.3h.png]

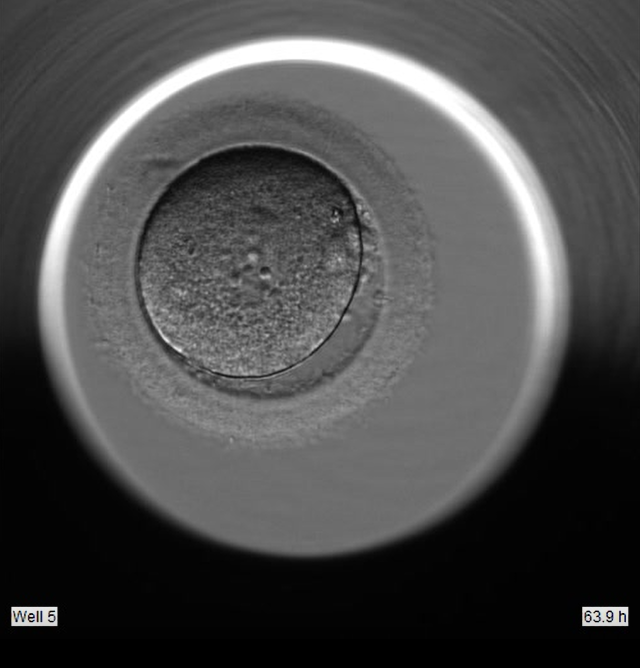

Supplement: Supplementary file 3 — Source data Fig. 1 [file 44319_2024_267_MOESM3_ESM.zip › Figure 1/1A/Em-6/D3 63.9h.png]

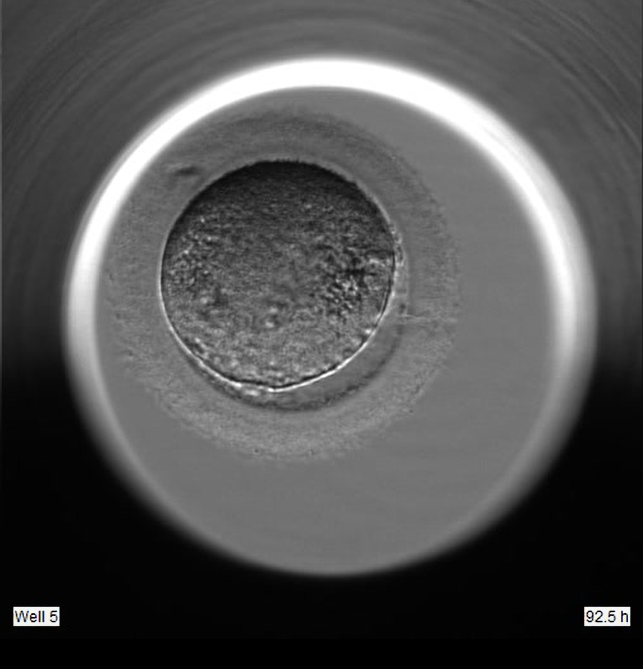

Supplement: Supplementary file 3 — Source data Fig. 1 [file 44319_2024_267_MOESM3_ESM.zip › Figure 1/1A/Em-6/D5 92.5h.png]

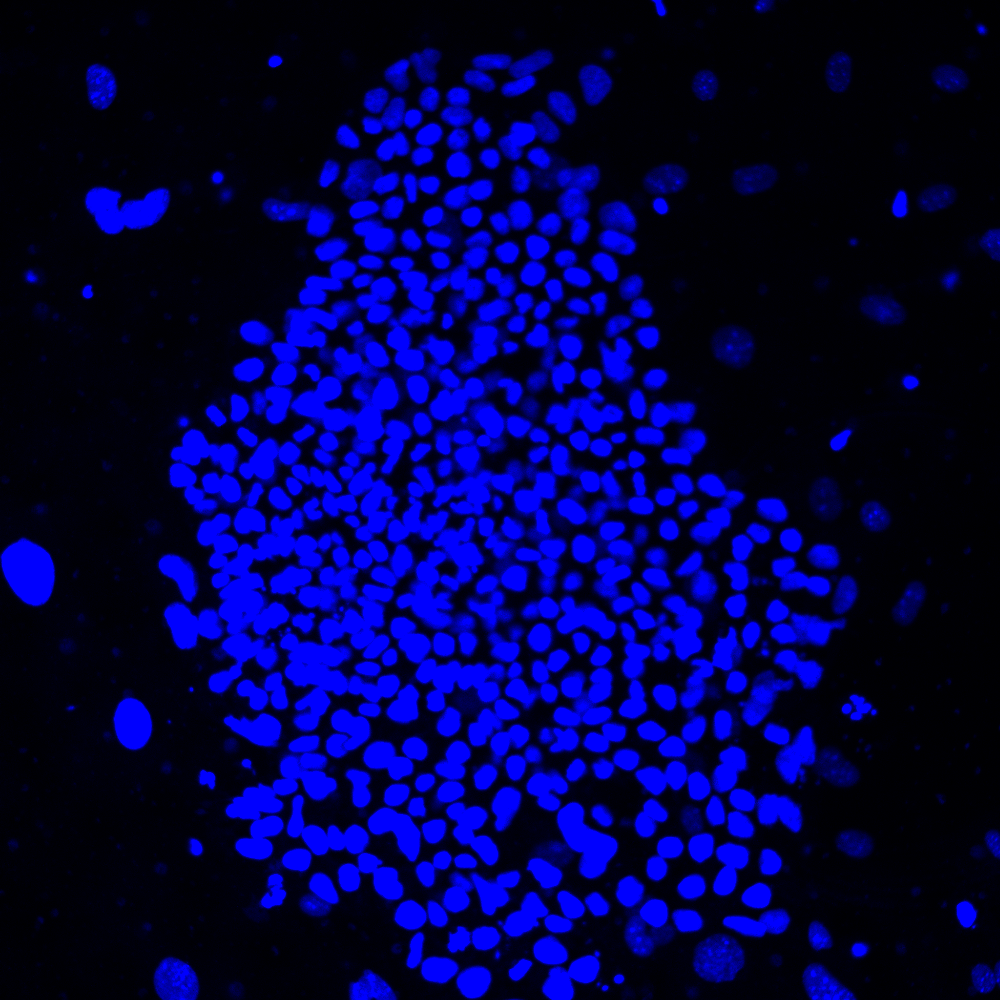

Supplement: Supplementary file 3 — Source data Fig. 1 [file 44319_2024_267_MOESM3_ESM.zip › Figure 1/1B/IF-OCT4-DAPI.tif]

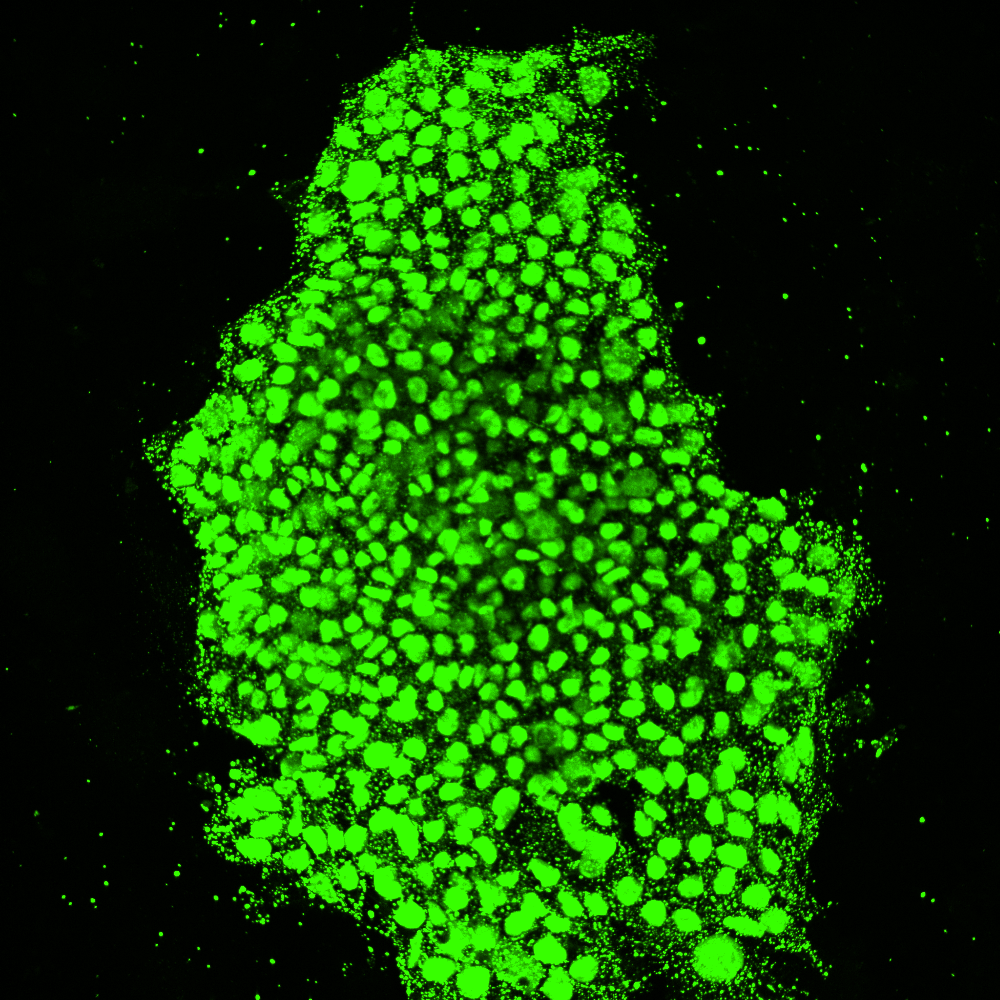

Supplement: Supplementary file 3 — Source data Fig. 1 [file 44319_2024_267_MOESM3_ESM.zip › Figure 1/1B/IF-OCT4-Marker.tif]

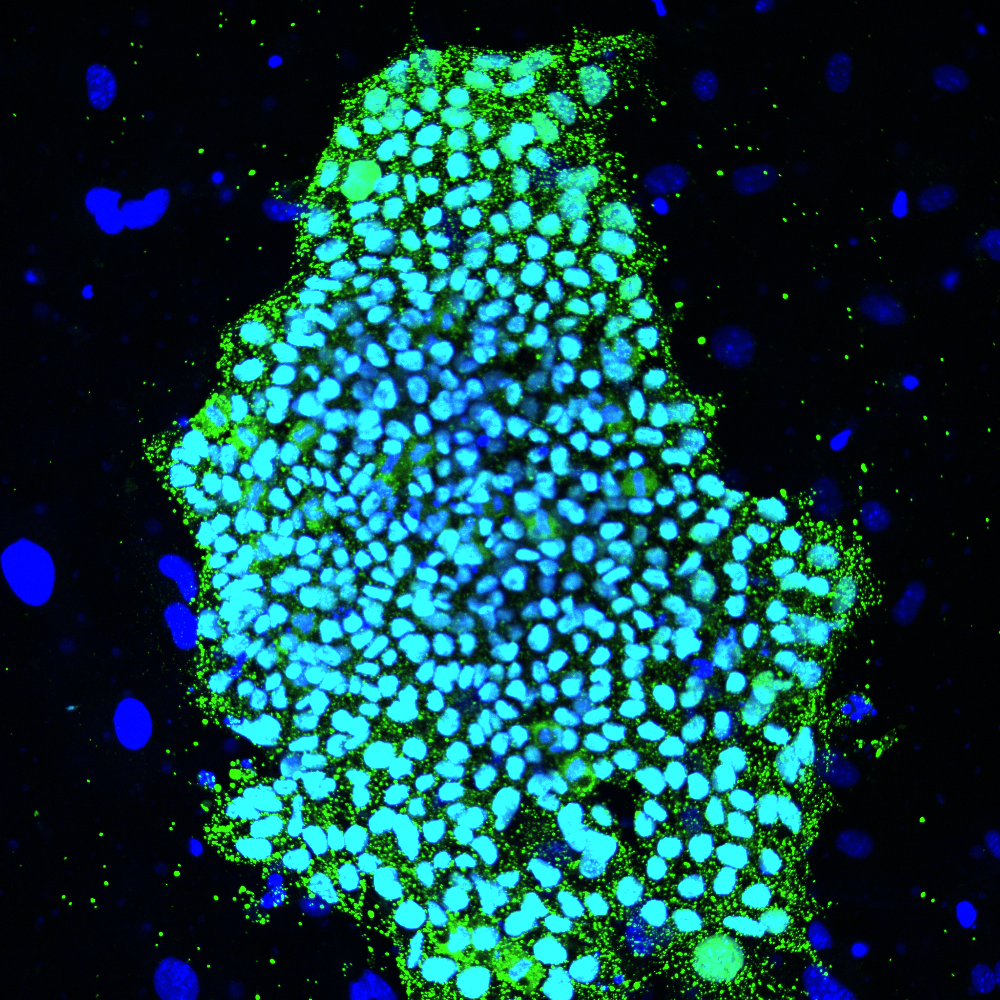

Supplement: Supplementary file 3 — Source data Fig. 1 [file 44319_2024_267_MOESM3_ESM.zip › Figure 1/1B/IF-OCT4-Merge.tif]

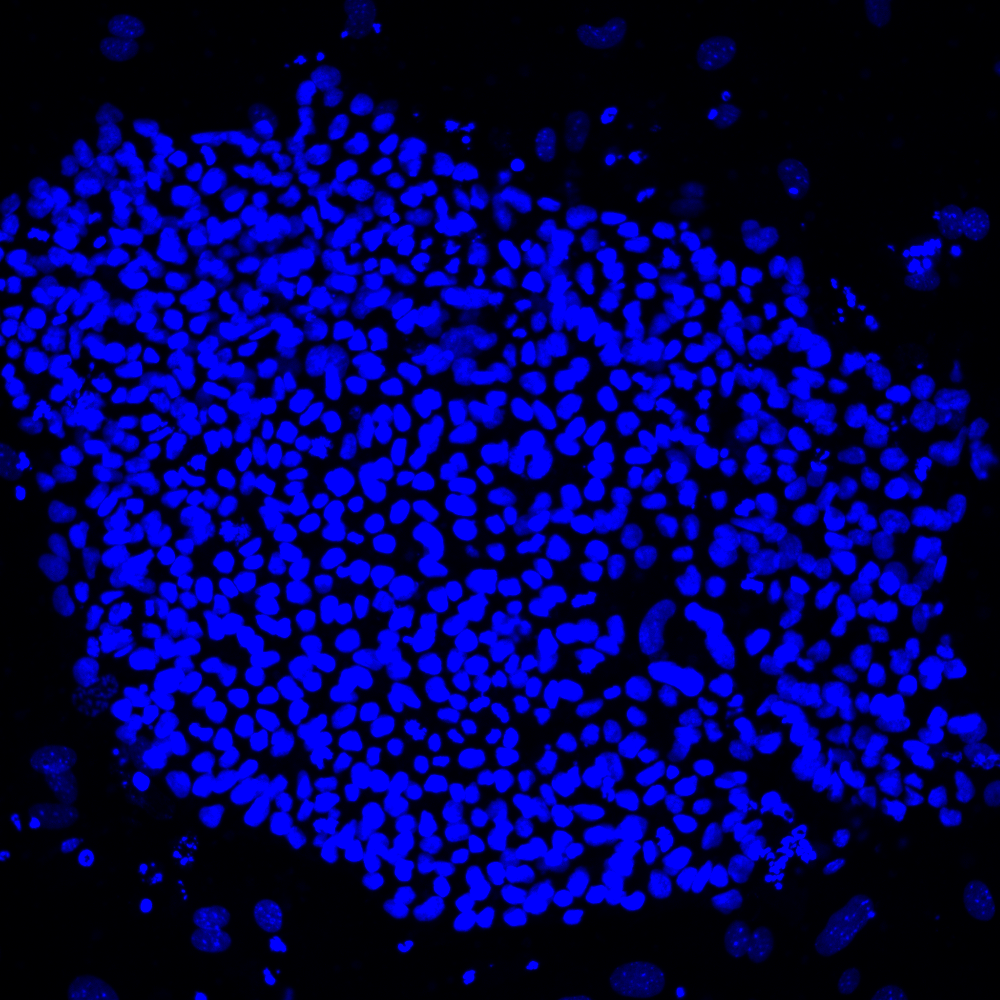

Supplement: Supplementary file 3 — Source data Fig. 1 [file 44319_2024_267_MOESM3_ESM.zip › Figure 1/1B/IF-SOX2-DAPI.tif]

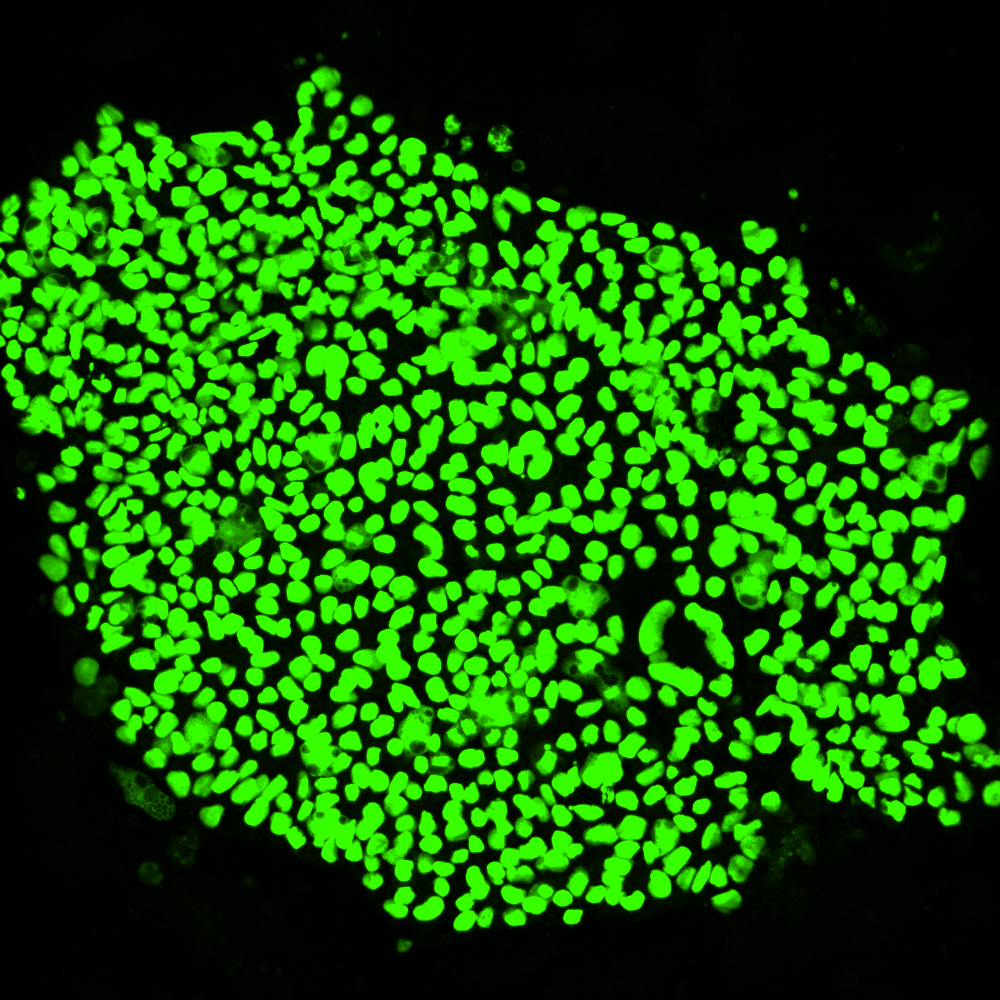

Supplement: Supplementary file 3 — Source data Fig. 1 [file 44319_2024_267_MOESM3_ESM.zip › Figure 1/1B/IF-SOX2-Marker.tif]

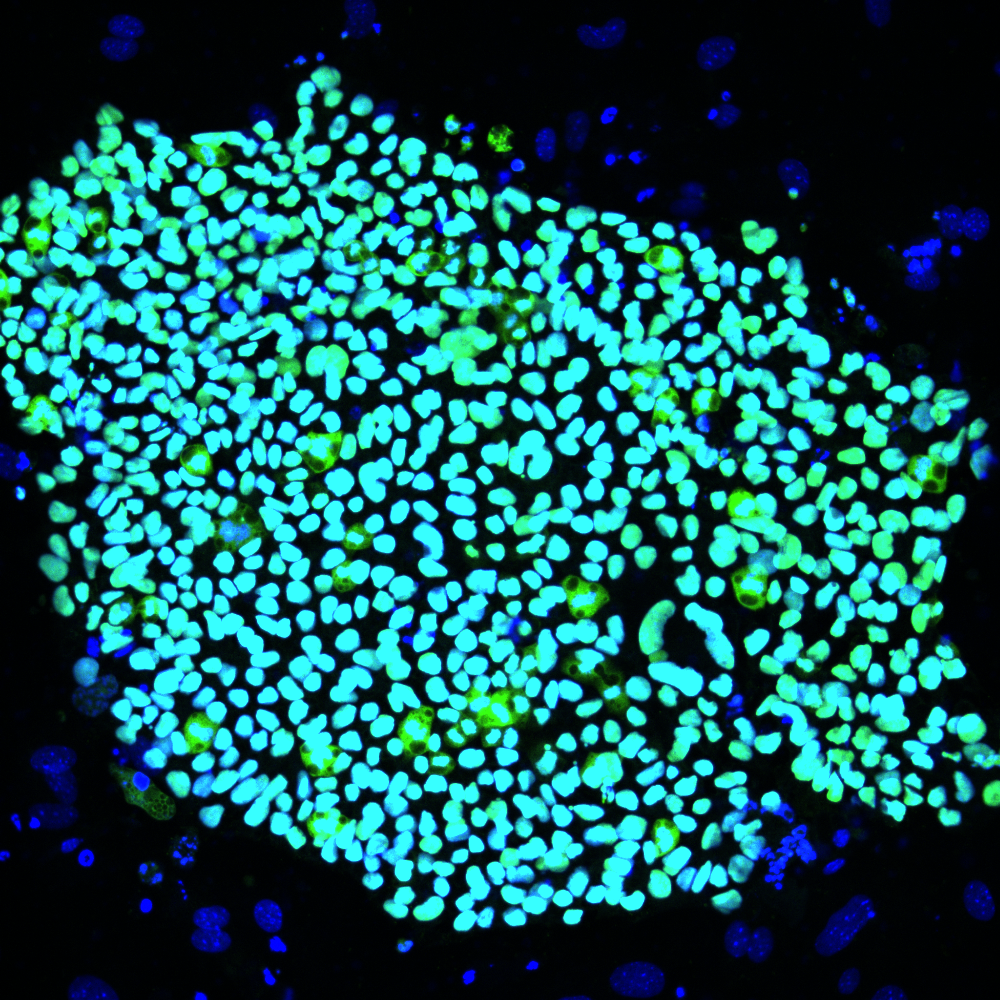

Supplement: Supplementary file 3 — Source data Fig. 1 [file 44319_2024_267_MOESM3_ESM.zip › Figure 1/1B/IF-SOX2-Merge.tif]

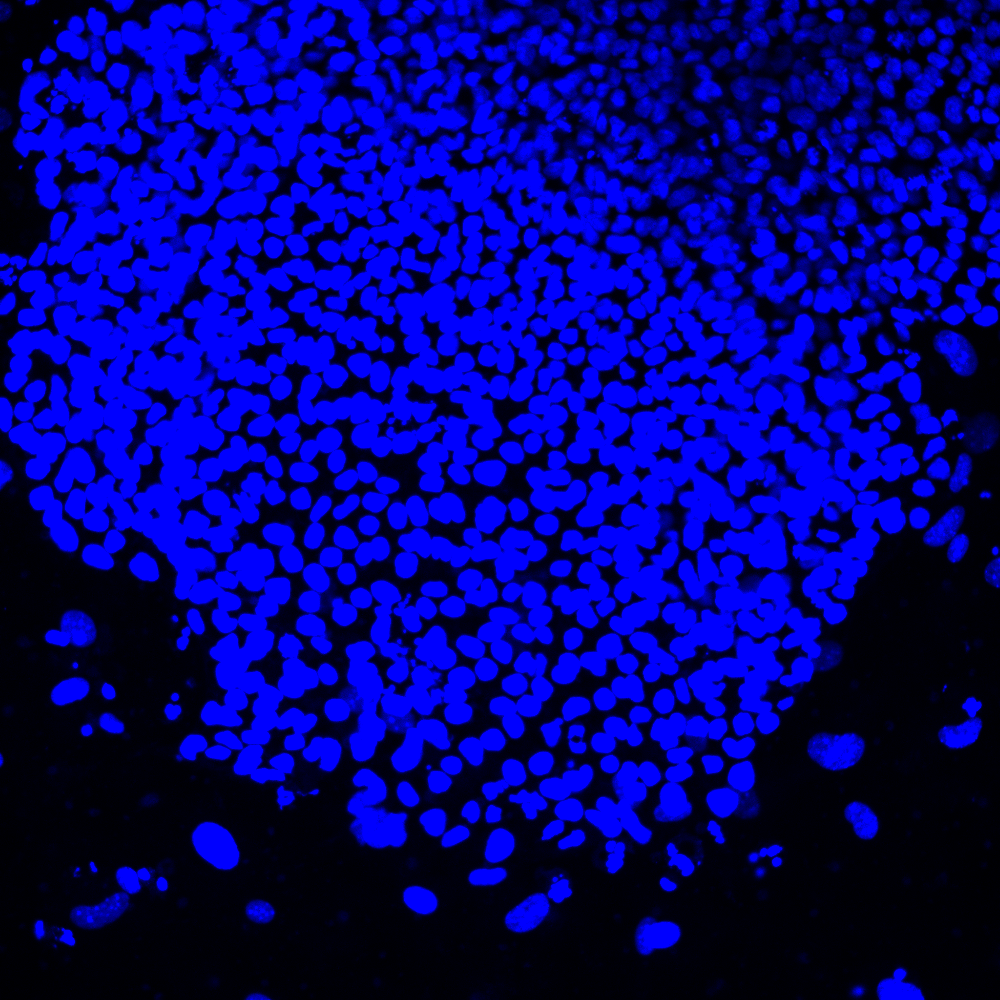

Supplement: Supplementary file 3 — Source data Fig. 1 [file 44319_2024_267_MOESM3_ESM.zip › Figure 1/1B/IF-SSEA4-DAPI.tif]

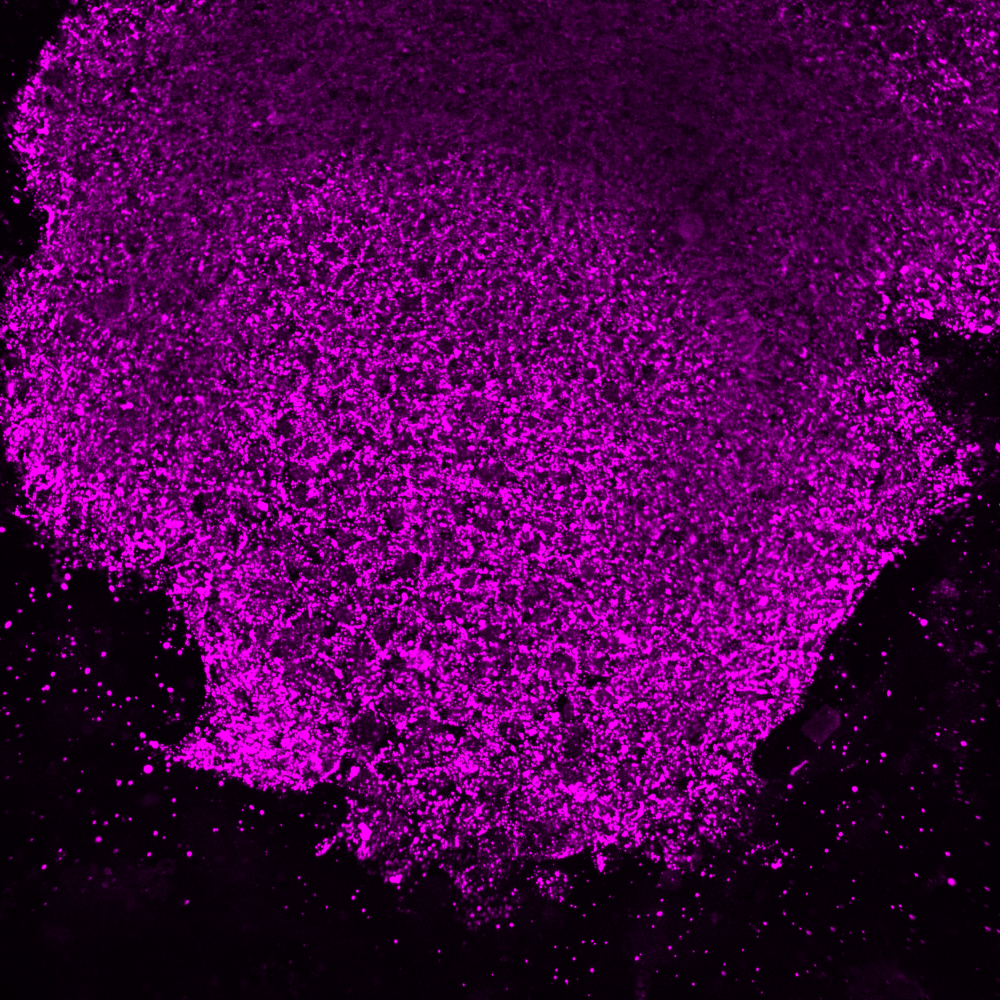

Supplement: Supplementary file 3 — Source data Fig. 1 [file 44319_2024_267_MOESM3_ESM.zip › Figure 1/1B/IF-SSEA4-Marker.png]

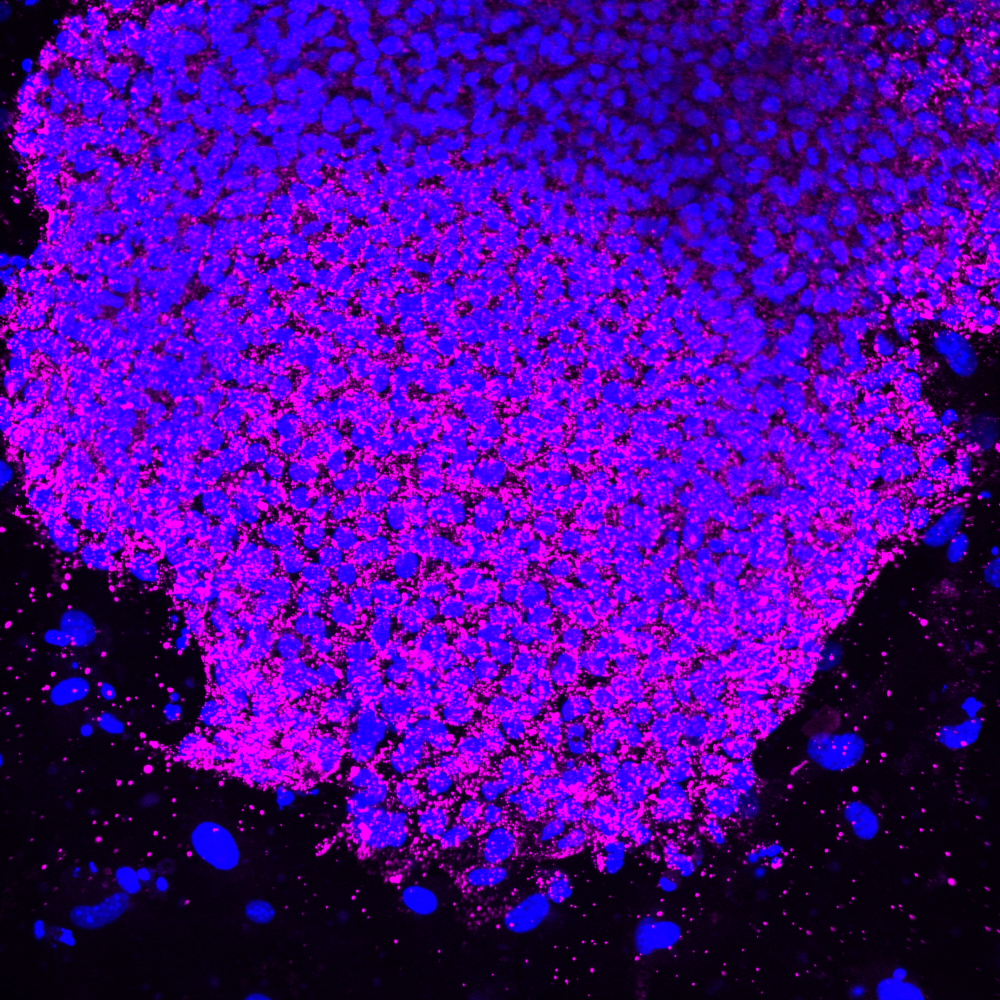

Supplement: Supplementary file 3 — Source data Fig. 1 [file 44319_2024_267_MOESM3_ESM.zip › Figure 1/1B/IF-SSEA4-Merge.png]

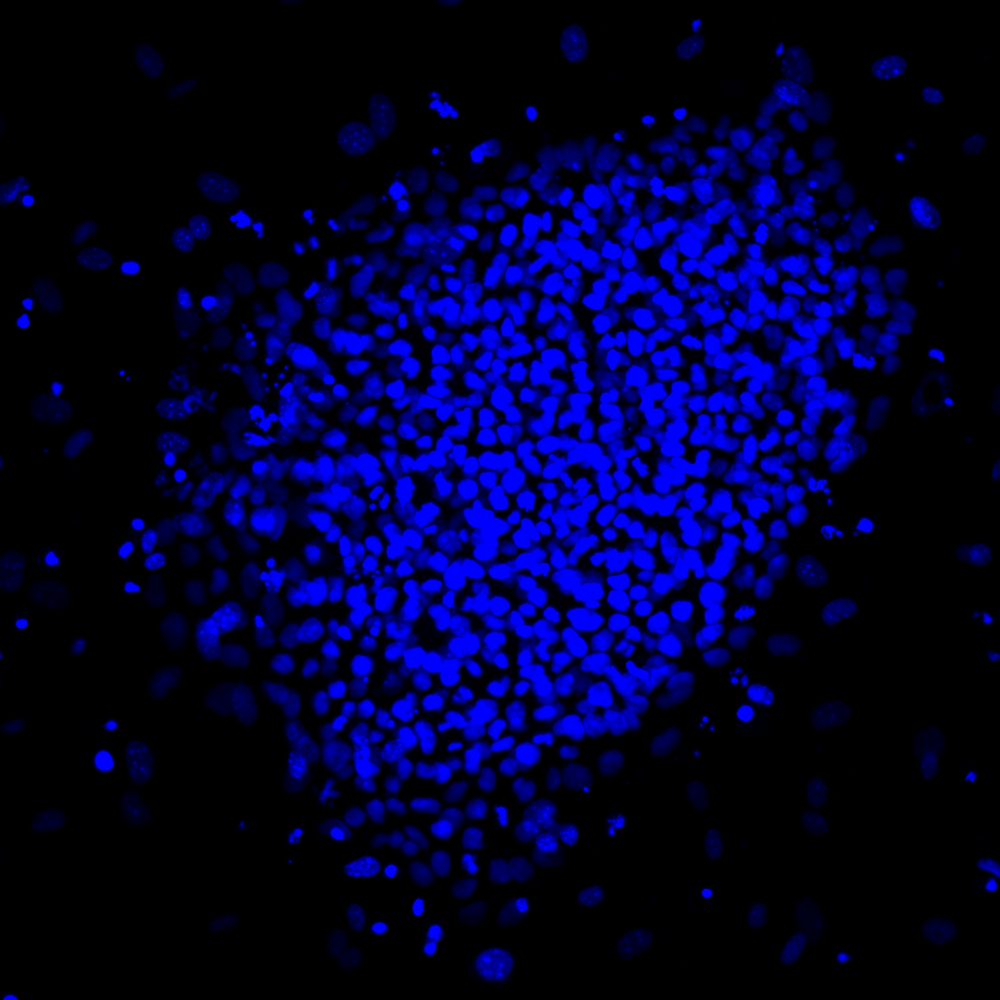

Supplement: Supplementary file 3 — Source data Fig. 1 [file 44319_2024_267_MOESM3_ESM.zip › Figure 1/1B/IF-TRA-1-60-DAPI.tif]

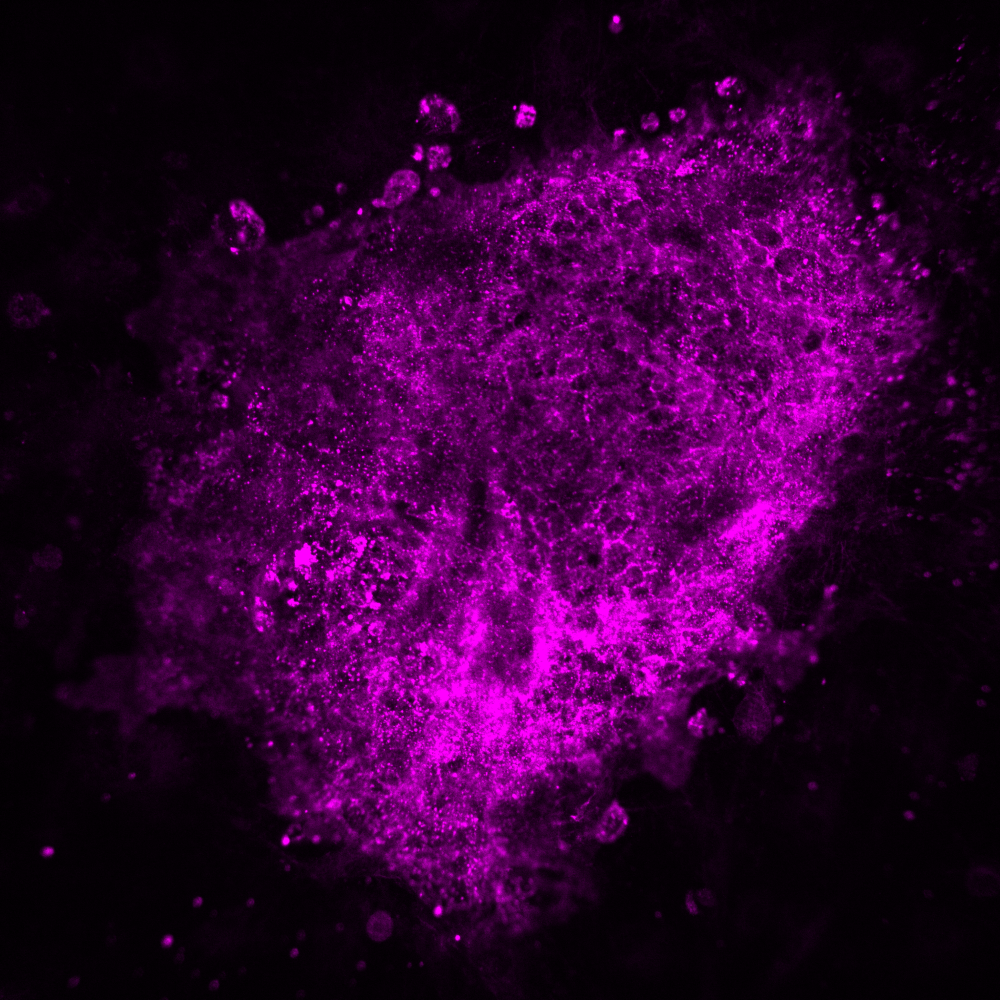

Supplement: Supplementary file 3 — Source data Fig. 1 [file 44319_2024_267_MOESM3_ESM.zip › Figure 1/1B/IF-TRA-1-60-Marker.png]

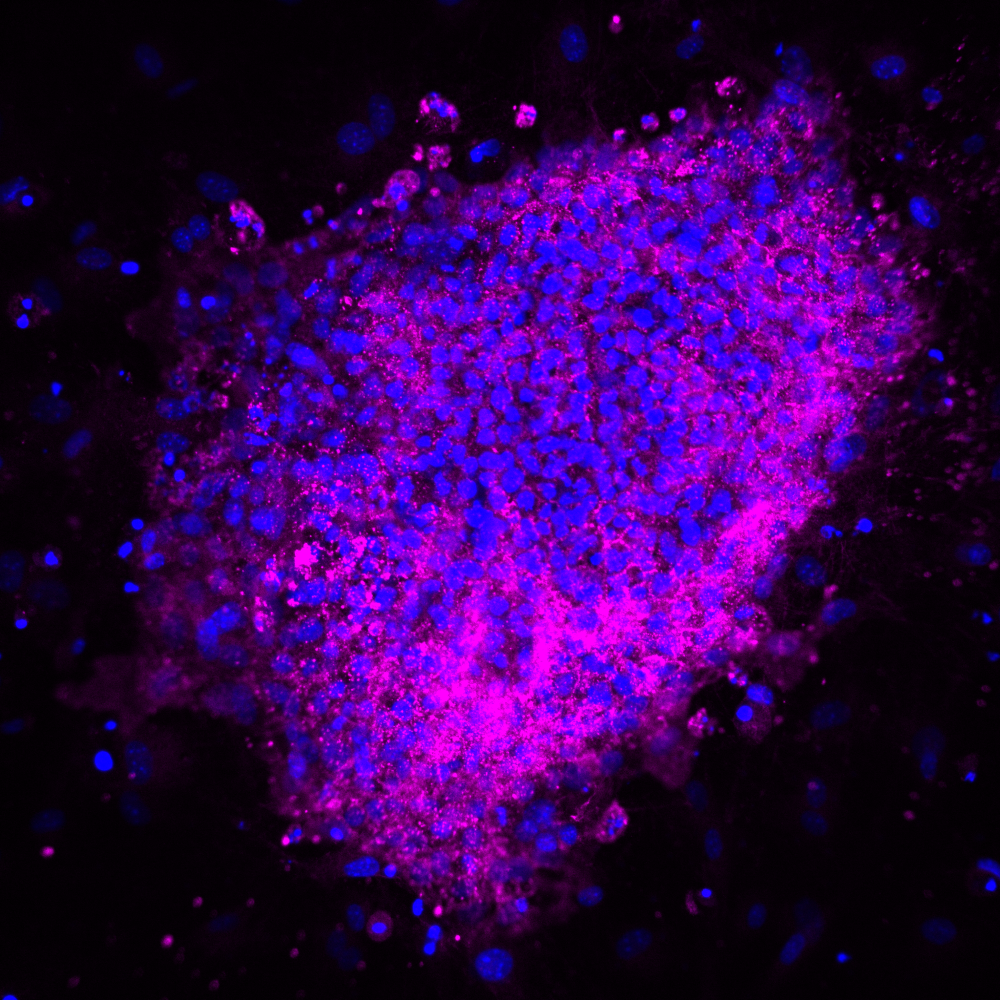

Supplement: Supplementary file 3 — Source data Fig. 1 [file 44319_2024_267_MOESM3_ESM.zip › Figure 1/1B/IF-TRA-1-60-Merge.png]

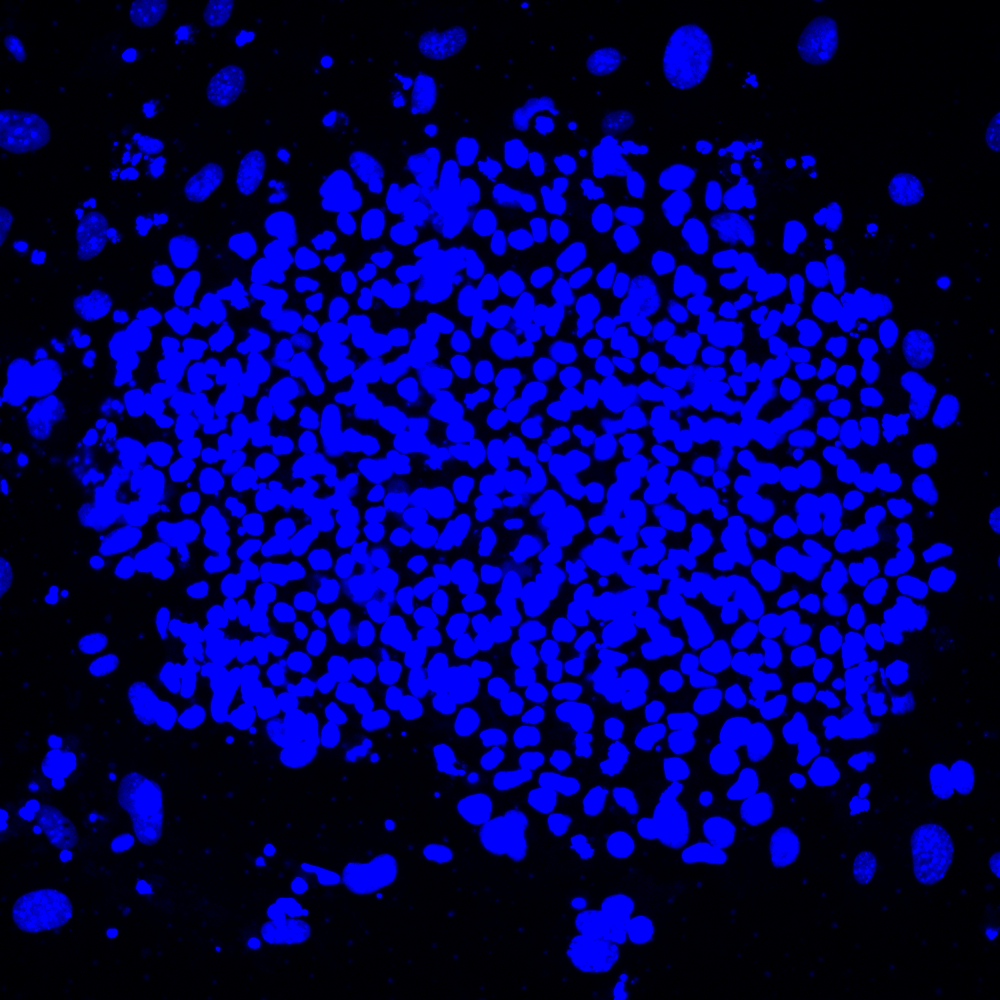

Supplement: Supplementary file 3 — Source data Fig. 1 [file 44319_2024_267_MOESM3_ESM.zip › Figure 1/1B/IF-TRA-1-81-DAPI.tif]

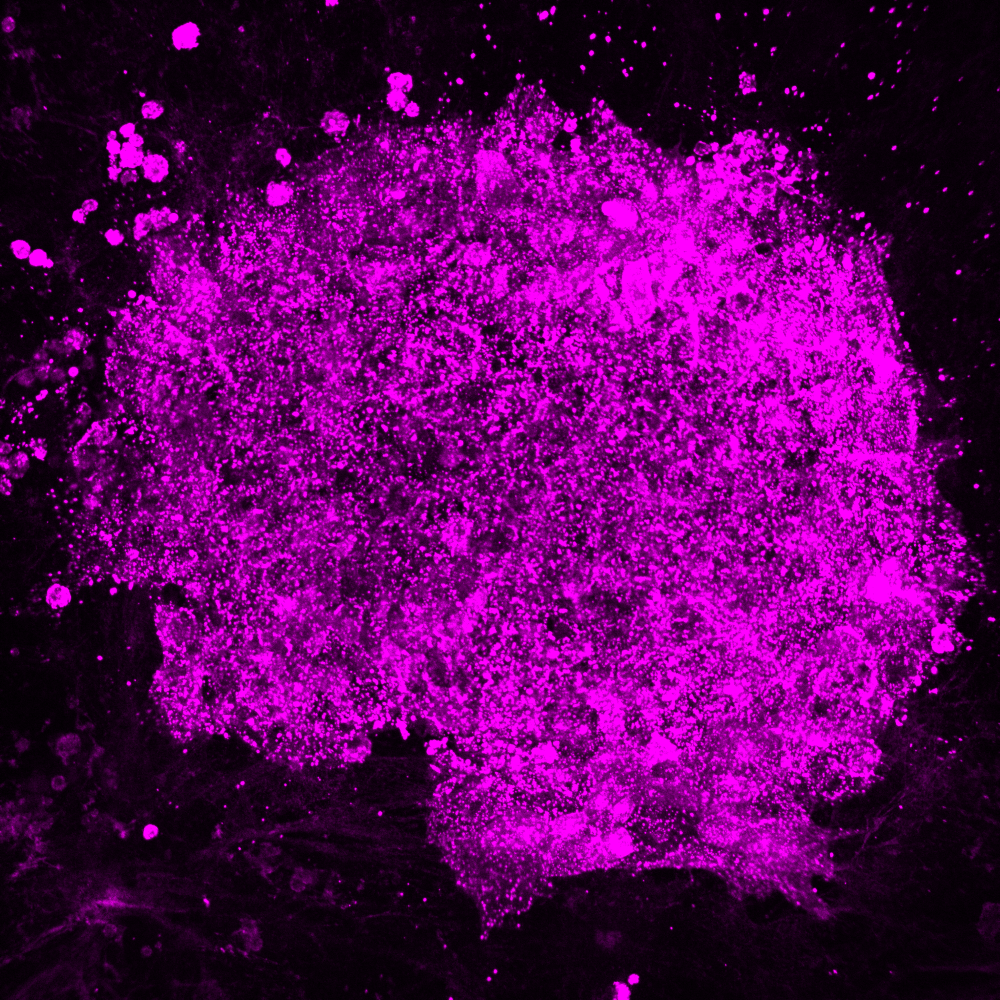

Supplement: Supplementary file 3 — Source data Fig. 1 [file 44319_2024_267_MOESM3_ESM.zip › Figure 1/1B/IF-TRA-1-81-Marker.png]

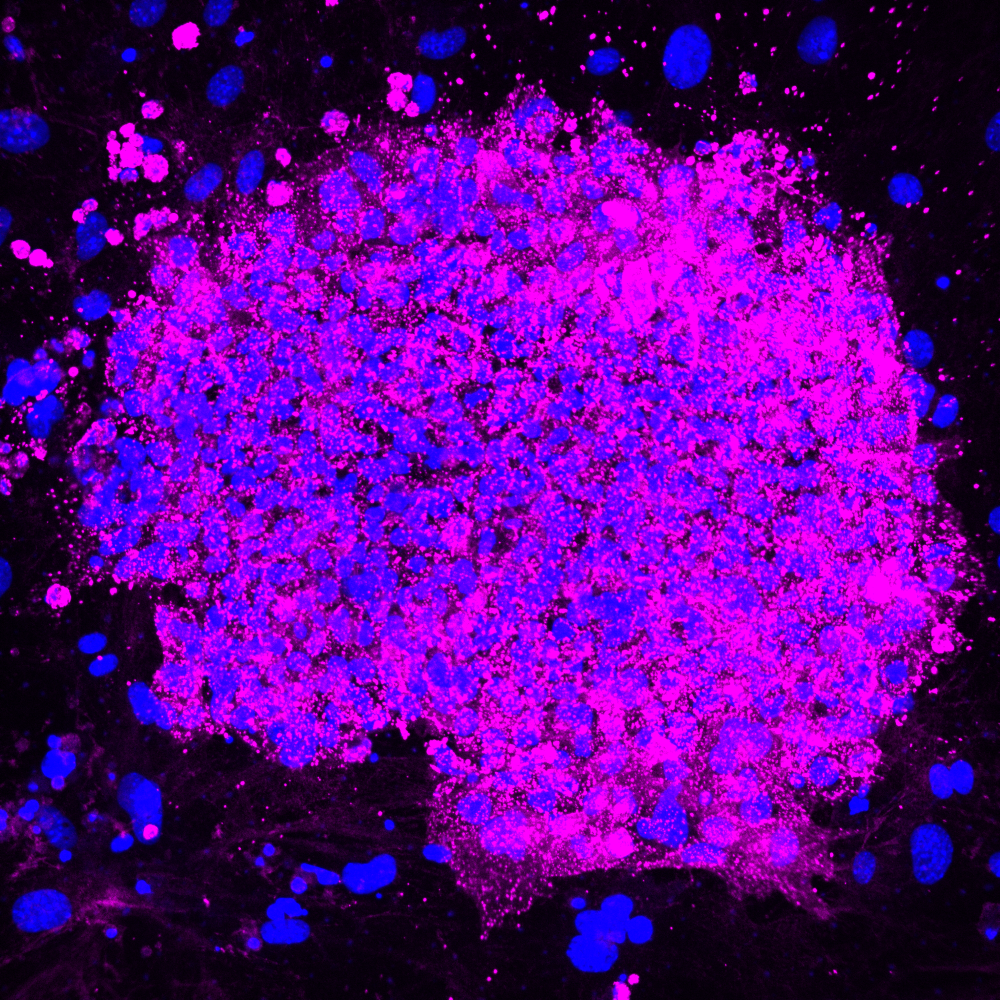

Supplement: Supplementary file 3 — Source data Fig. 1 [file 44319_2024_267_MOESM3_ESM.zip › Figure 1/1B/IF-TRA-1-81-Merge.png]

**ESC_Em-1: 46, XX; Balanced; Euploid**


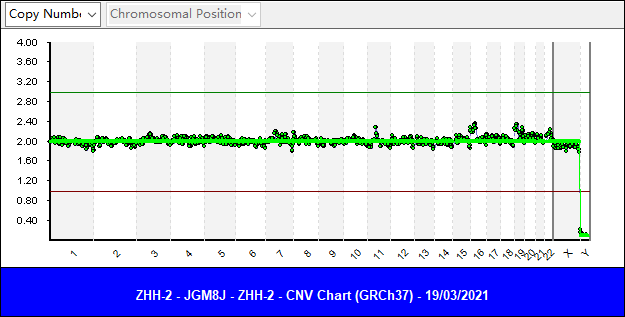

Supplement: Supplementary file 3 — Source data Fig. 1 [file 44319_2024_267_MOESM3_ESM.zip › Figure 1/1C/CNV Result for ESC-Em-1.docx]

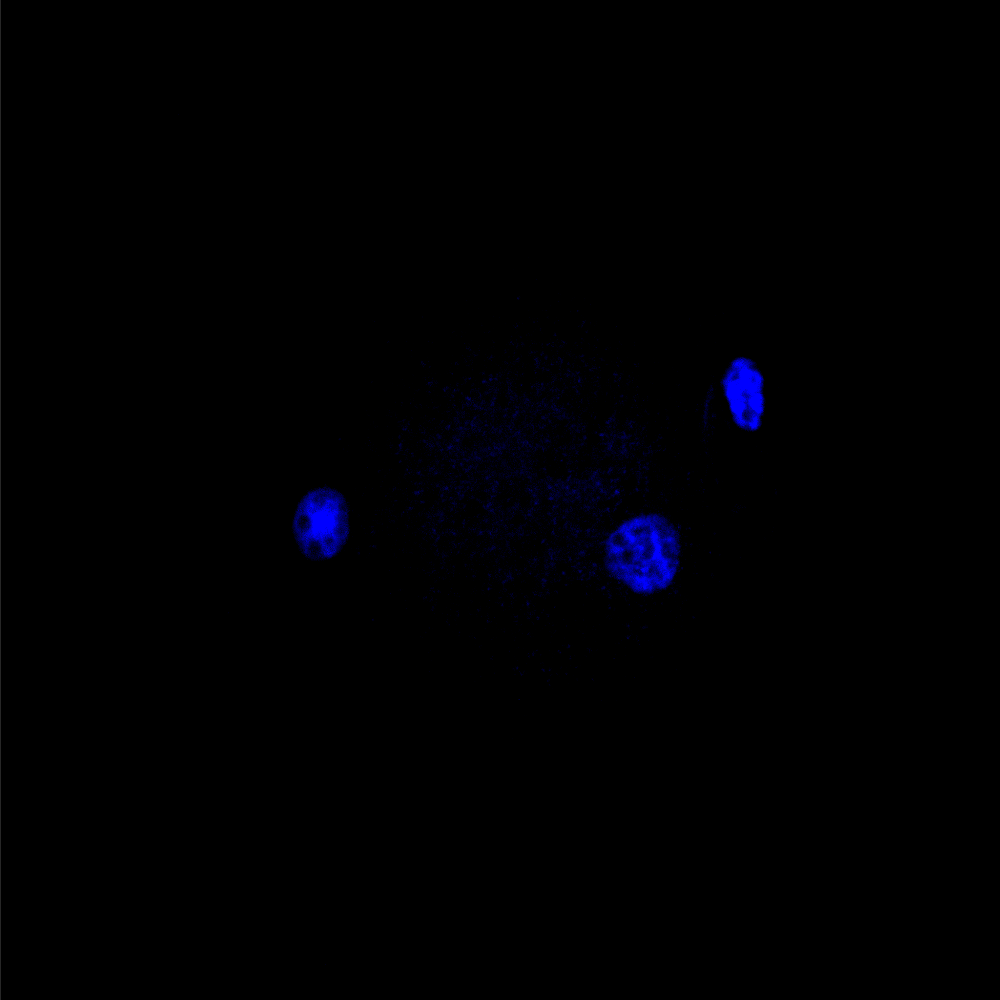

Supplement: Supplementary file 4 — Source data Fig. 2 [file 44319_2024_267_MOESM4_ESM.zip › Figure 2/2A/IF-1-DAPI.tif]

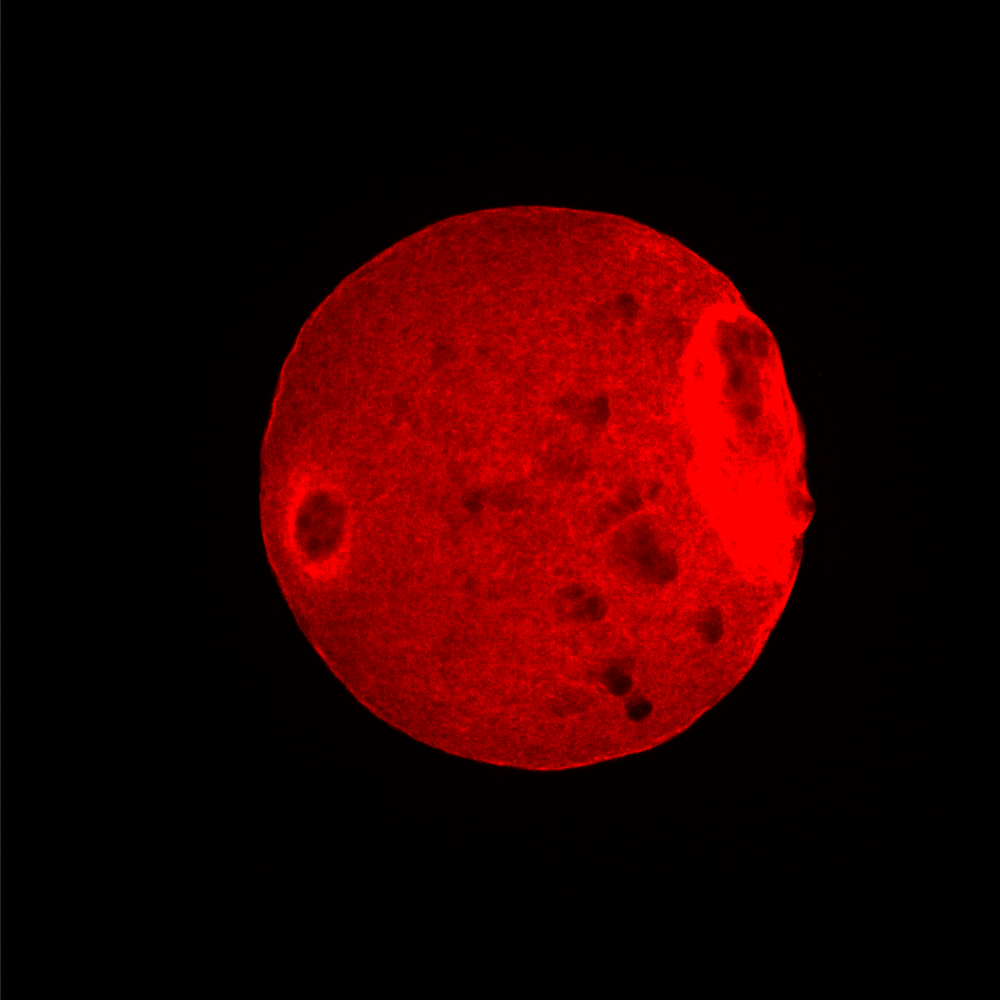

Supplement: Supplementary file 4 — Source data Fig. 2 [file 44319_2024_267_MOESM4_ESM.zip › Figure 2/2A/IF-1-Factin.tif]

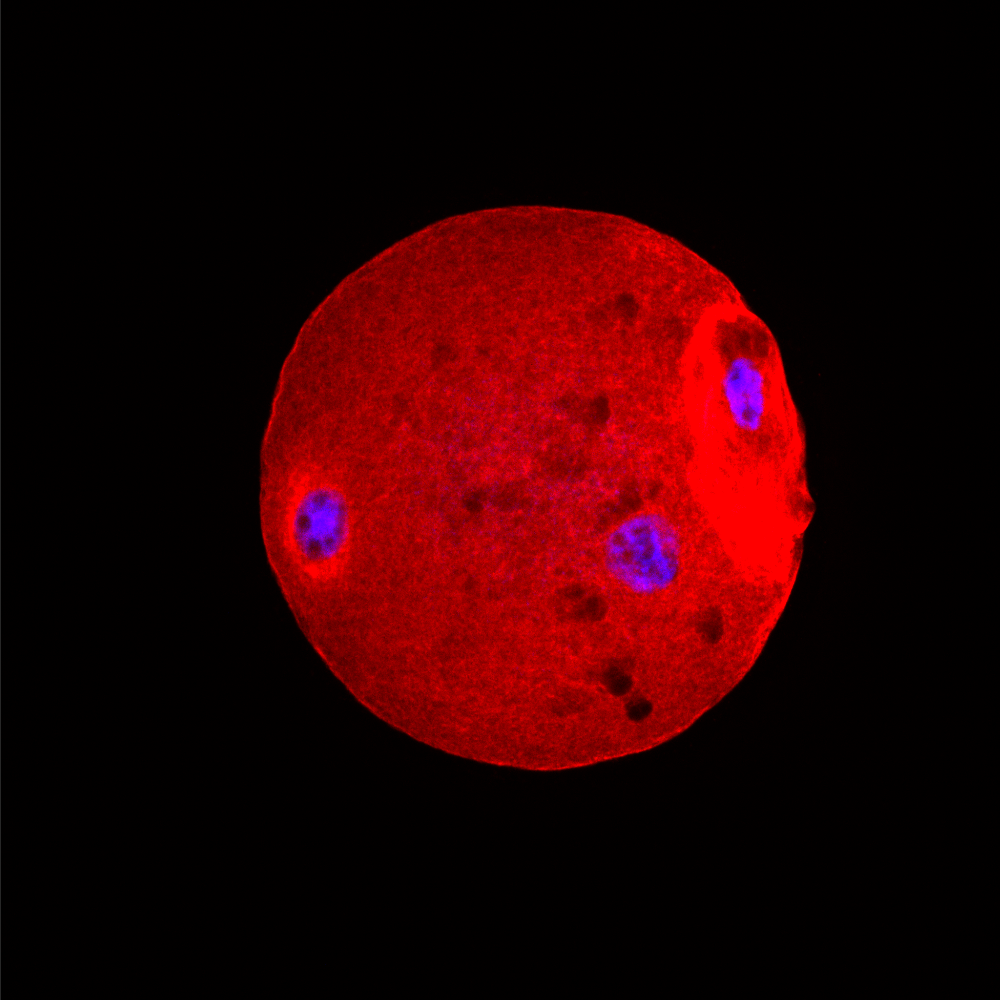

Supplement: Supplementary file 4 — Source data Fig. 2 [file 44319_2024_267_MOESM4_ESM.zip › Figure 2/2A/IF-1-Merge.tif]

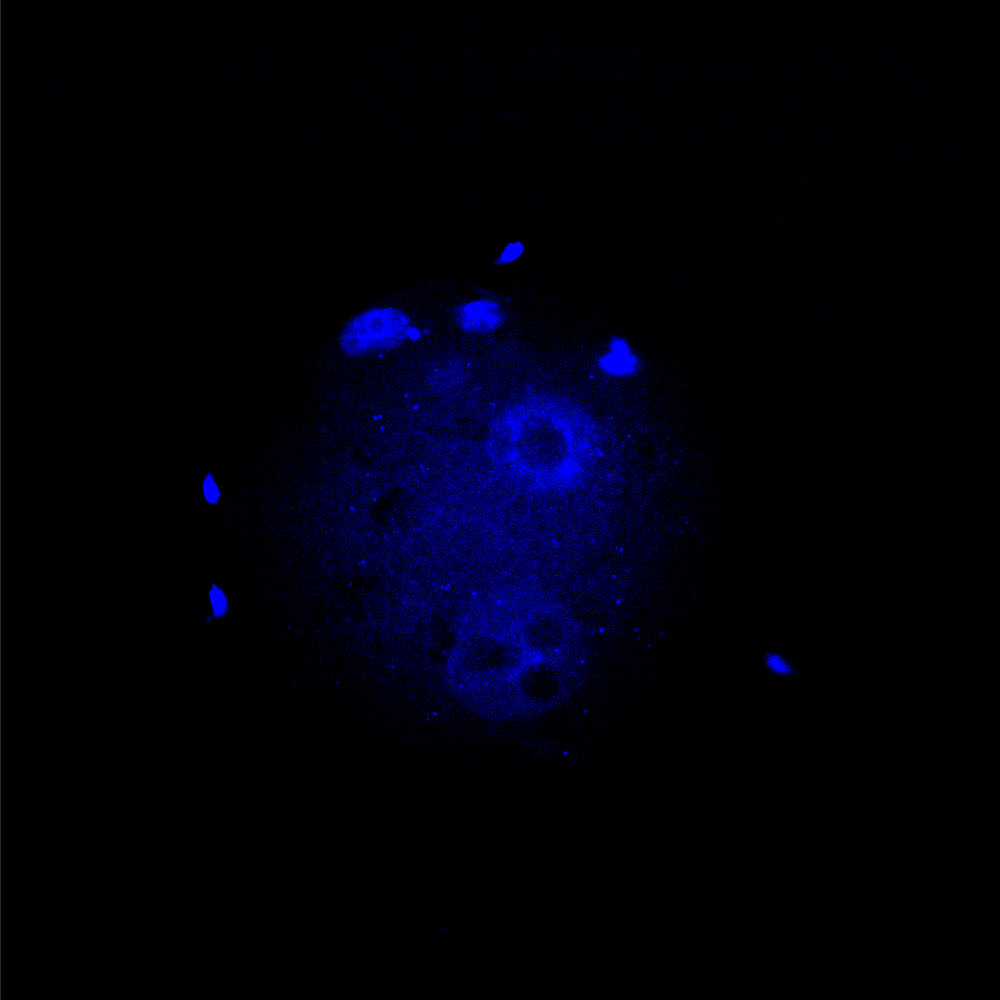

Supplement: Supplementary file 4 — Source data Fig. 2 [file 44319_2024_267_MOESM4_ESM.zip › Figure 2/2A/IF-2-DAPI.tif]

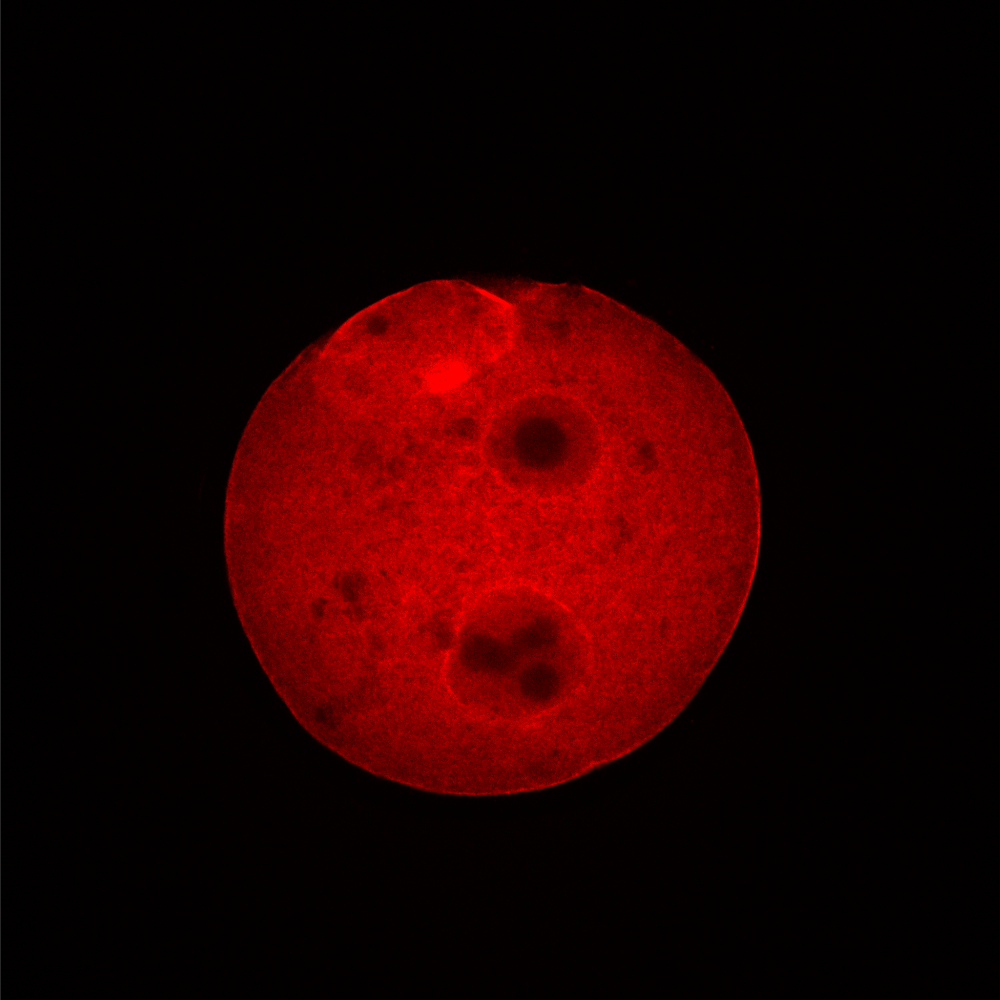

Supplement: Supplementary file 4 — Source data Fig. 2 [file 44319_2024_267_MOESM4_ESM.zip › Figure 2/2A/IF-2-Factin.tif]

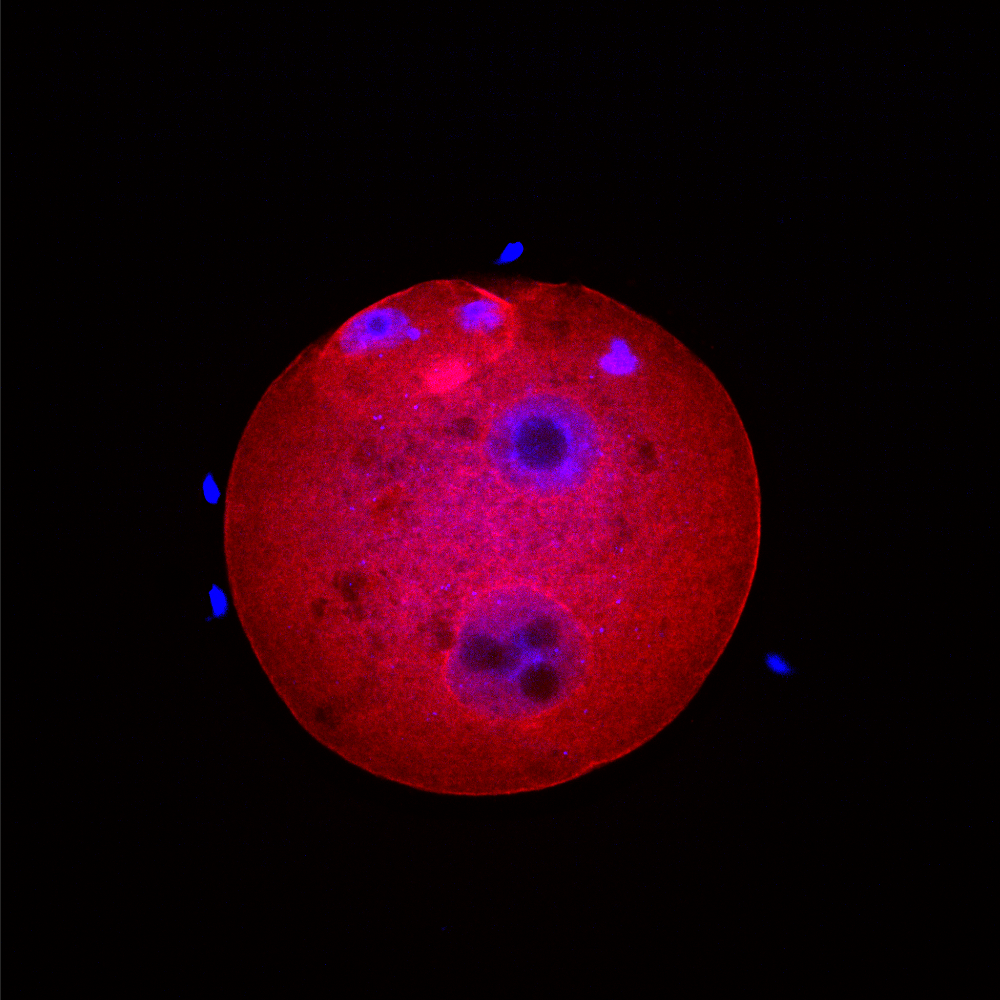

Supplement: Supplementary file 4 — Source data Fig. 2 [file 44319_2024_267_MOESM4_ESM.zip › Figure 2/2A/IF-2-Merge.tif]

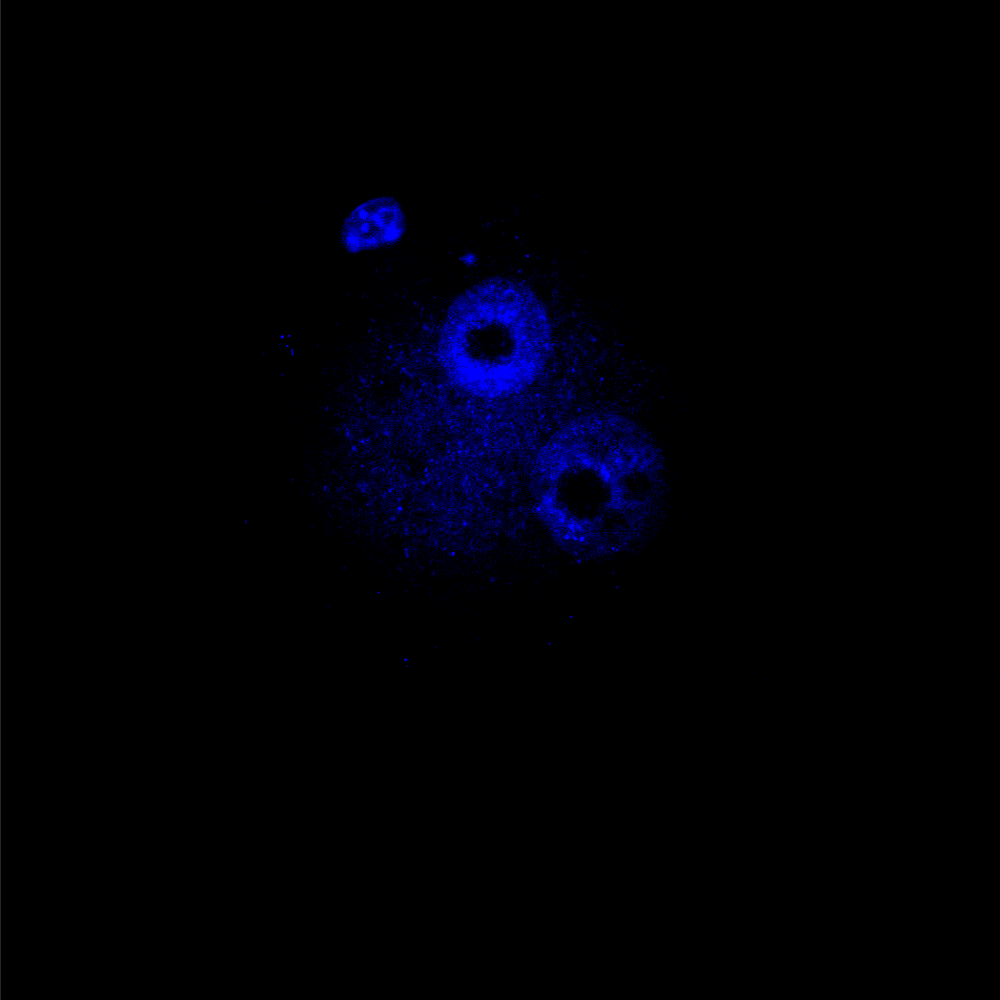

Supplement: Supplementary file 4 — Source data Fig. 2 [file 44319_2024_267_MOESM4_ESM.zip › Figure 2/2A/IF-3-DAPI.tif]

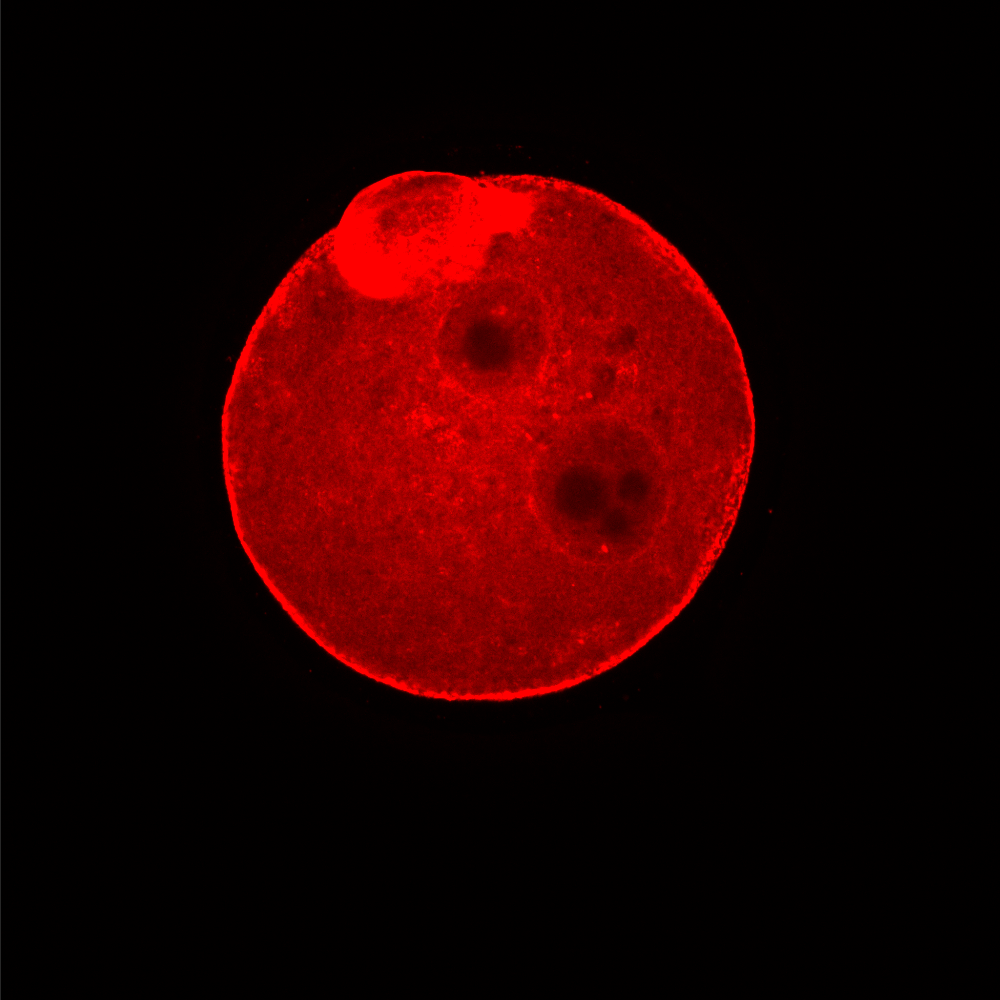

Supplement: Supplementary file 4 — Source data Fig. 2 [file 44319_2024_267_MOESM4_ESM.zip › Figure 2/2A/IF-3-Factin.tif]

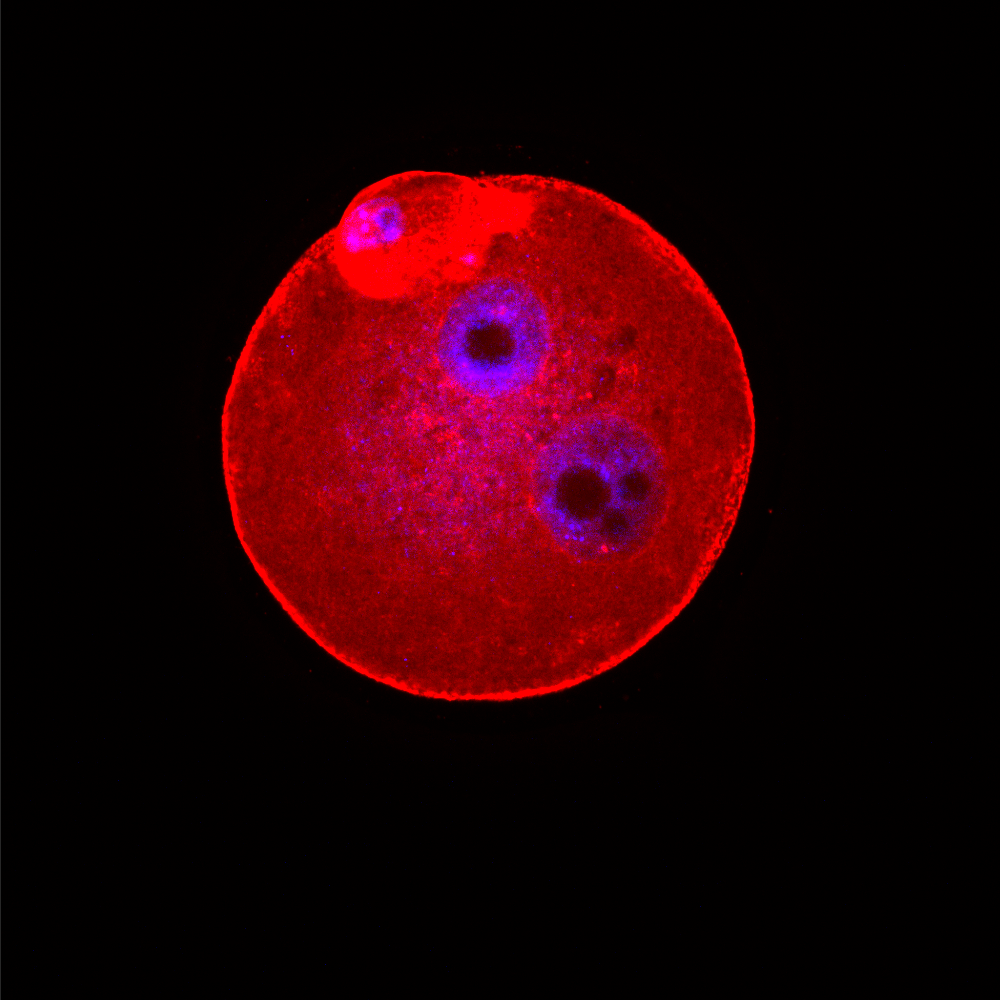

Supplement: Supplementary file 4 — Source data Fig. 2 [file 44319_2024_267_MOESM4_ESM.zip › Figure 2/2A/IF-3-Merge.tif]

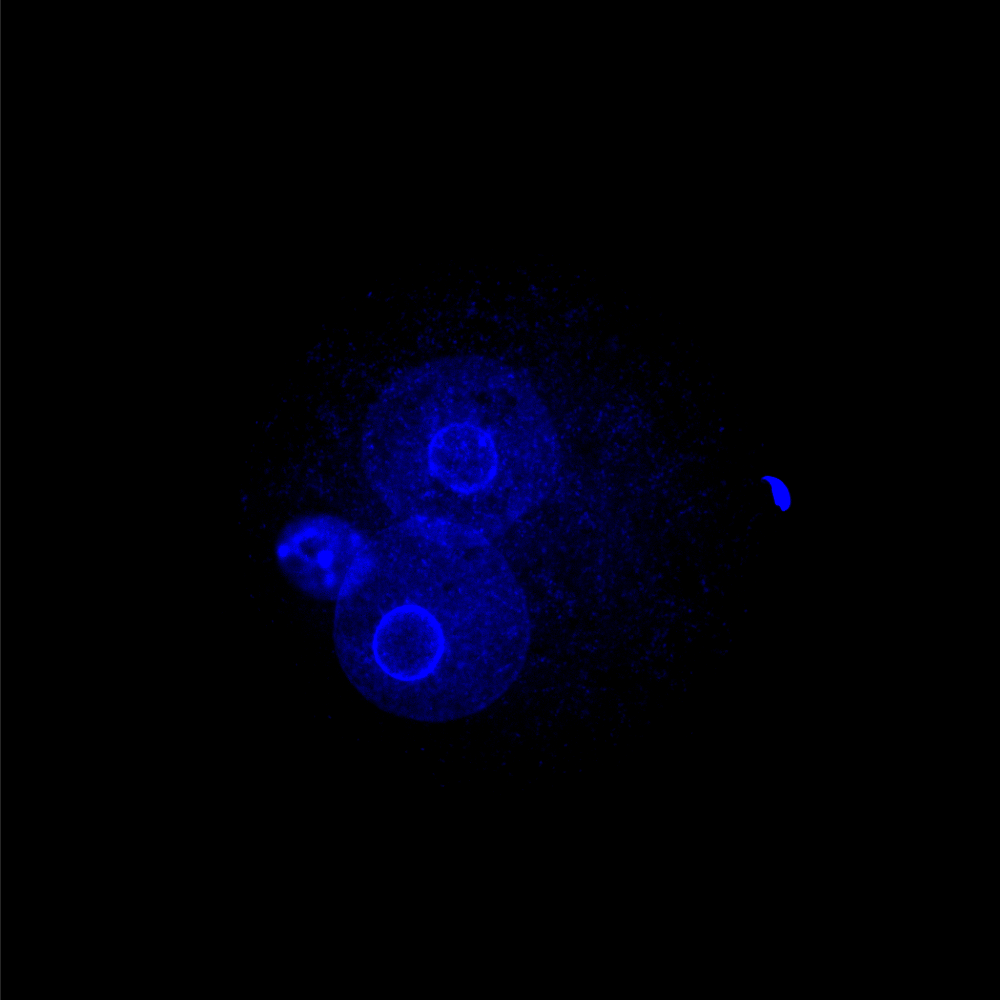

Supplement: Supplementary file 4 — Source data Fig. 2 [file 44319_2024_267_MOESM4_ESM.zip › Figure 2/2A/IF-4-DAPI.tif]

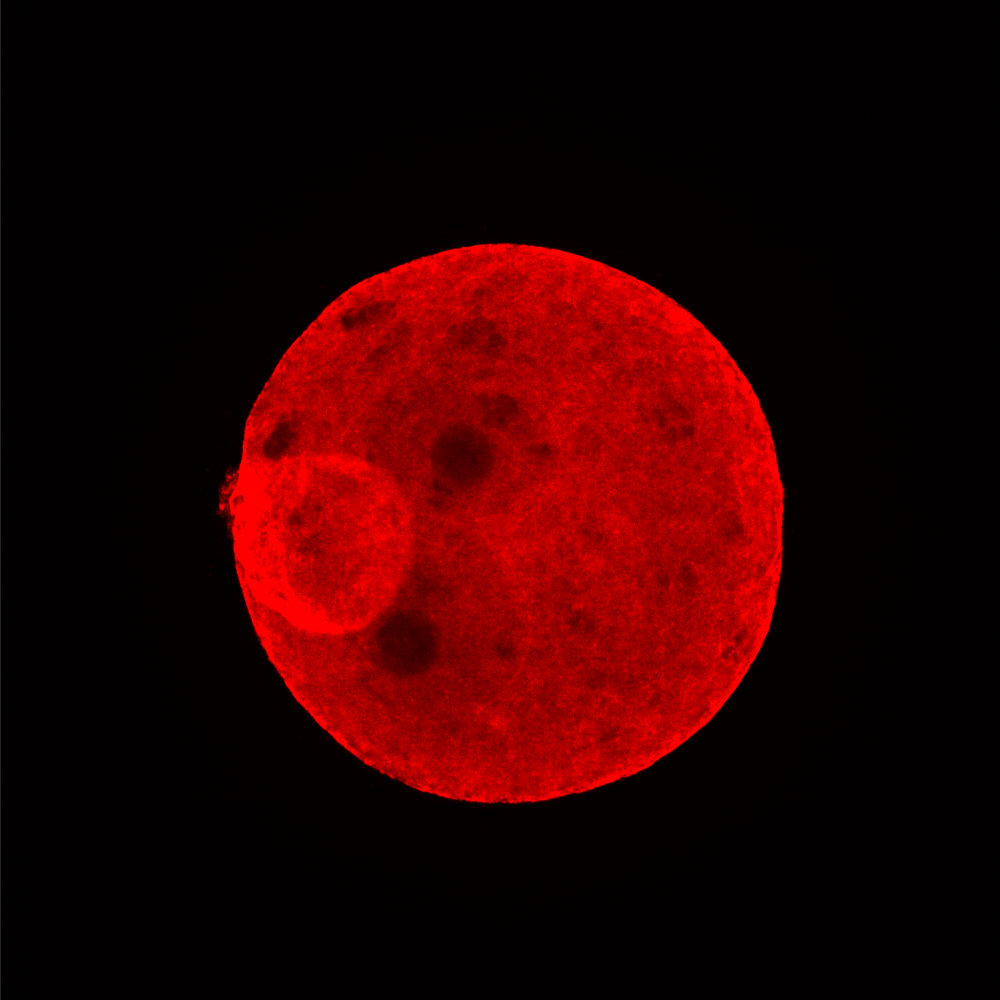

Supplement: Supplementary file 4 — Source data Fig. 2 [file 44319_2024_267_MOESM4_ESM.zip › Figure 2/2A/IF-4-Factin.tif]

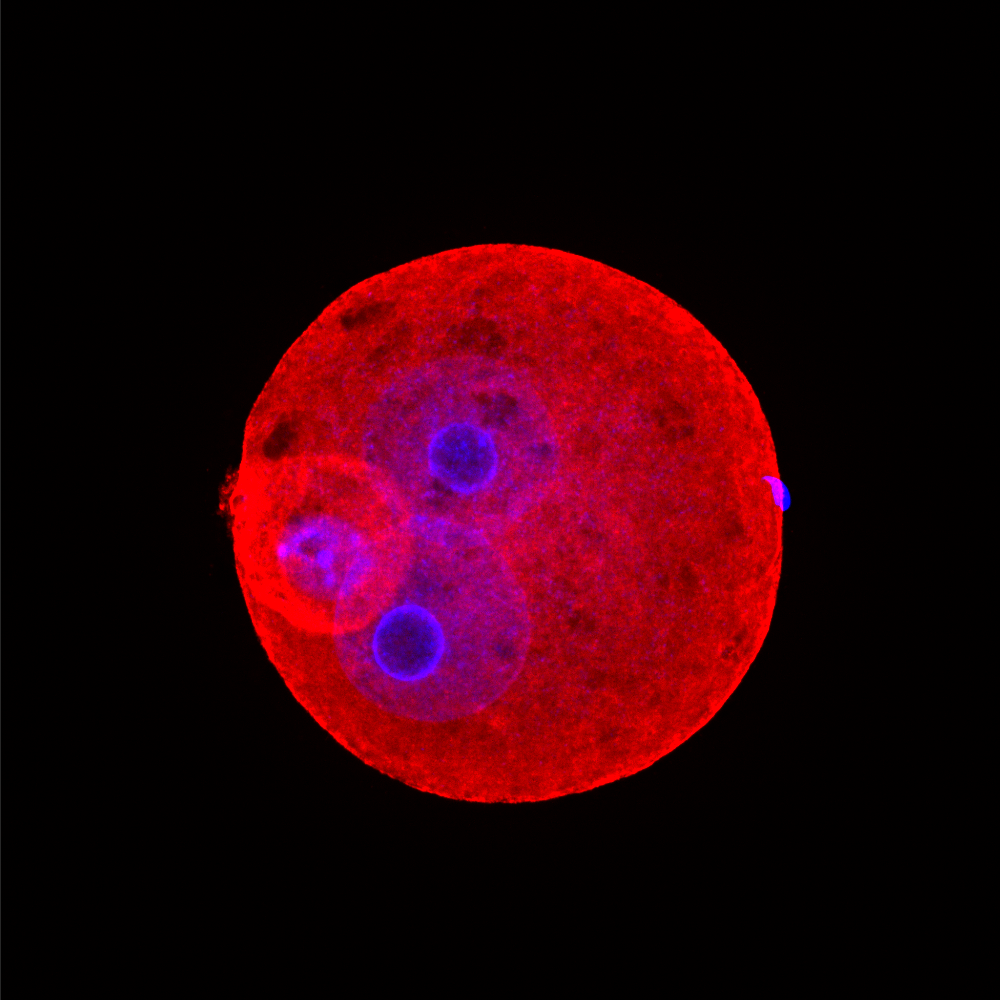

Supplement: Supplementary file 4 — Source data Fig. 2 [file 44319_2024_267_MOESM4_ESM.zip › Figure 2/2A/IF-4-Merge.tif]

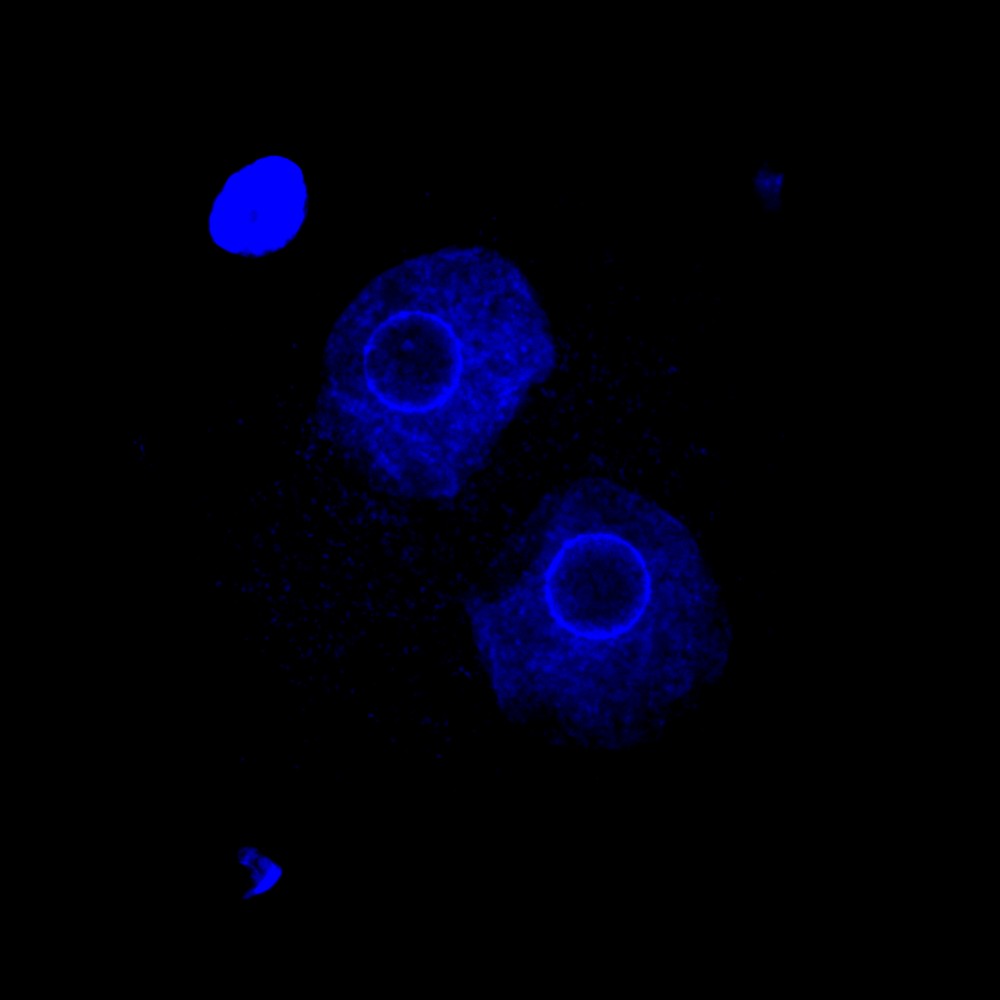

Supplement: Supplementary file 4 — Source data Fig. 2 [file 44319_2024_267_MOESM4_ESM.zip › Figure 2/2C/IF-F441-1-DAPI.png]

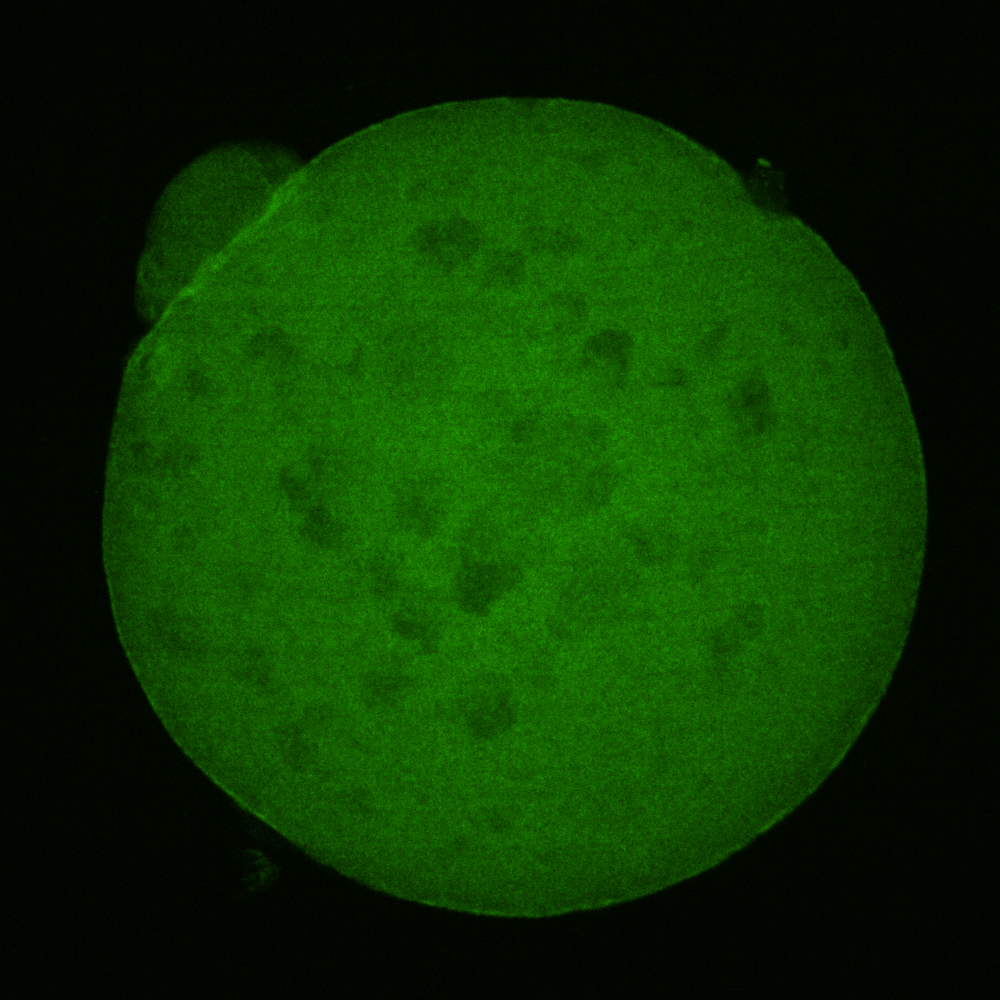

Supplement: Supplementary file 4 — Source data Fig. 2 [file 44319_2024_267_MOESM4_ESM.zip › Figure 2/2C/IF-F441-1-EGFP.png]

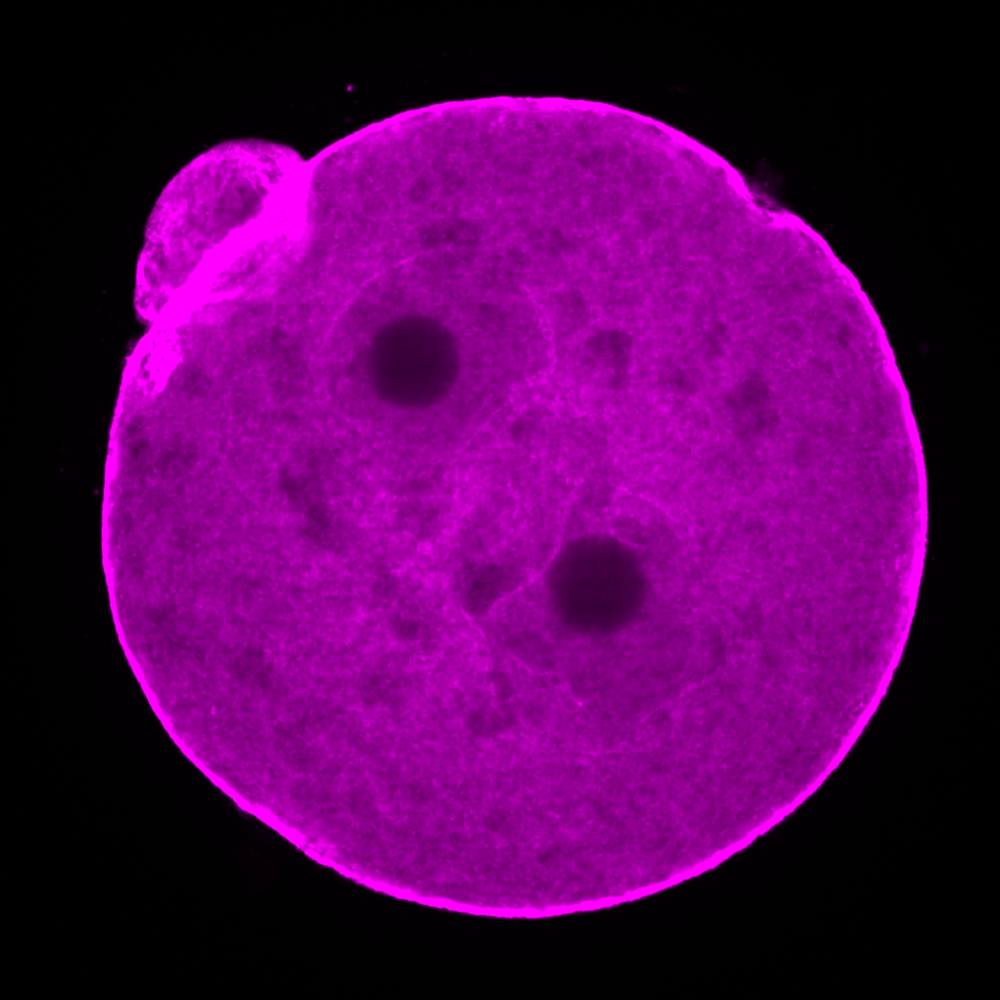

Supplement: Supplementary file 4 — Source data Fig. 2 [file 44319_2024_267_MOESM4_ESM.zip › Figure 2/2C/IF-F441-1-Factin.png]

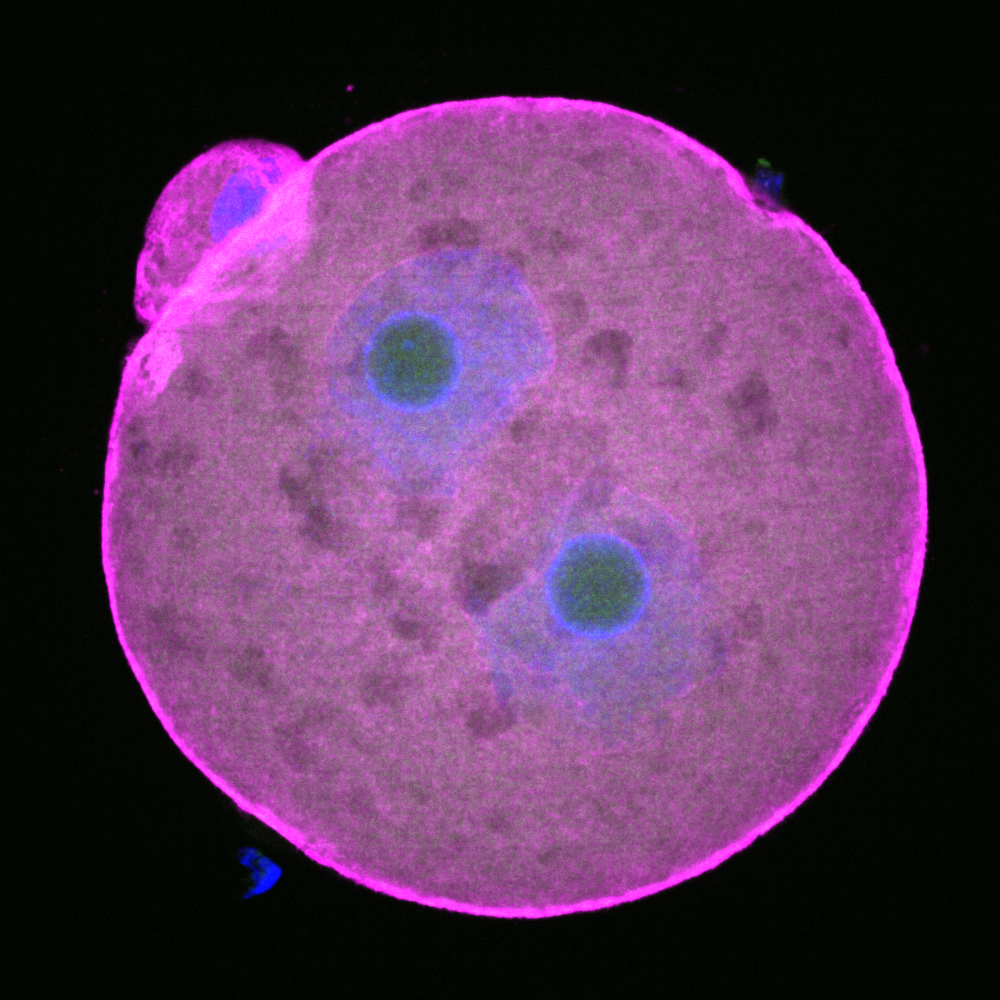

Supplement: Supplementary file 4 — Source data Fig. 2 [file 44319_2024_267_MOESM4_ESM.zip › Figure 2/2C/IF-F441-1-Merge.png]

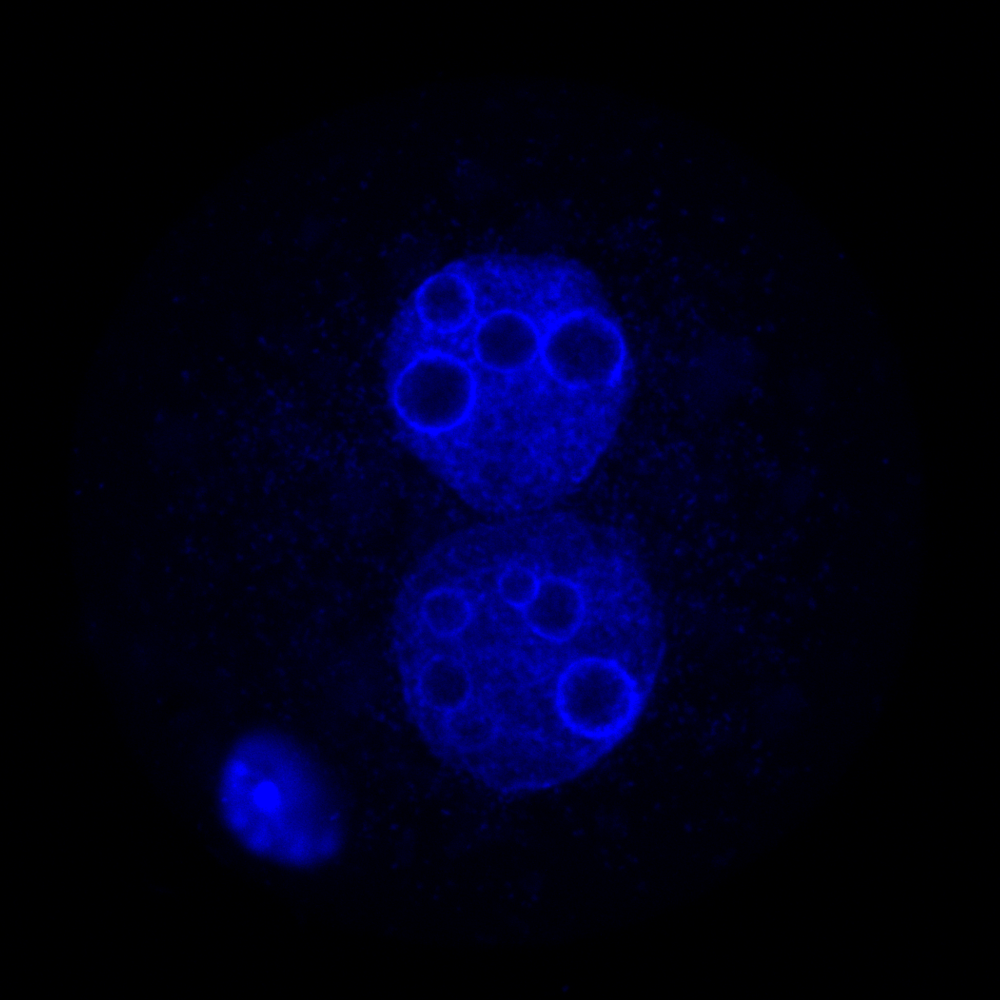

Supplement: Supplementary file 4 — Source data Fig. 2 [file 44319_2024_267_MOESM4_ESM.zip › Figure 2/2C/IF-F441-2-DAPI.png]

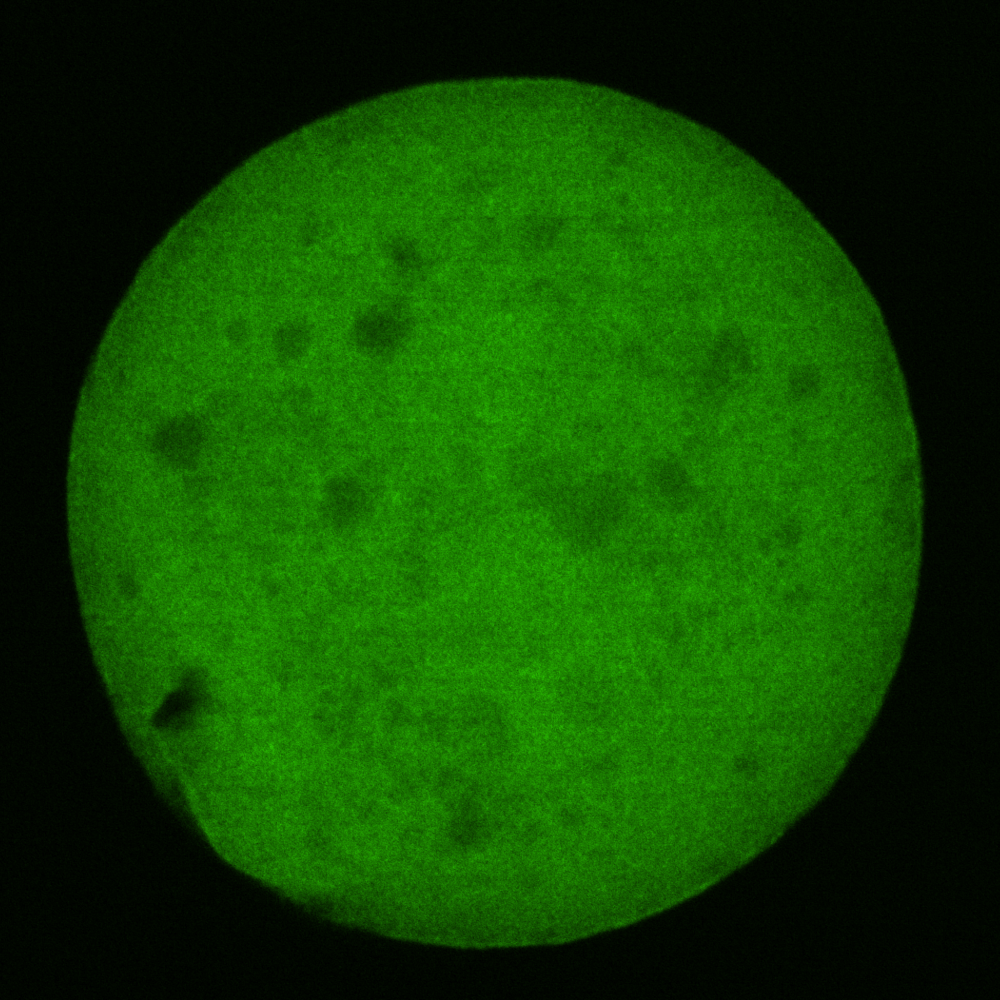

Supplement: Supplementary file 4 — Source data Fig. 2 [file 44319_2024_267_MOESM4_ESM.zip › Figure 2/2C/IF-F441-2-EGFP.png]

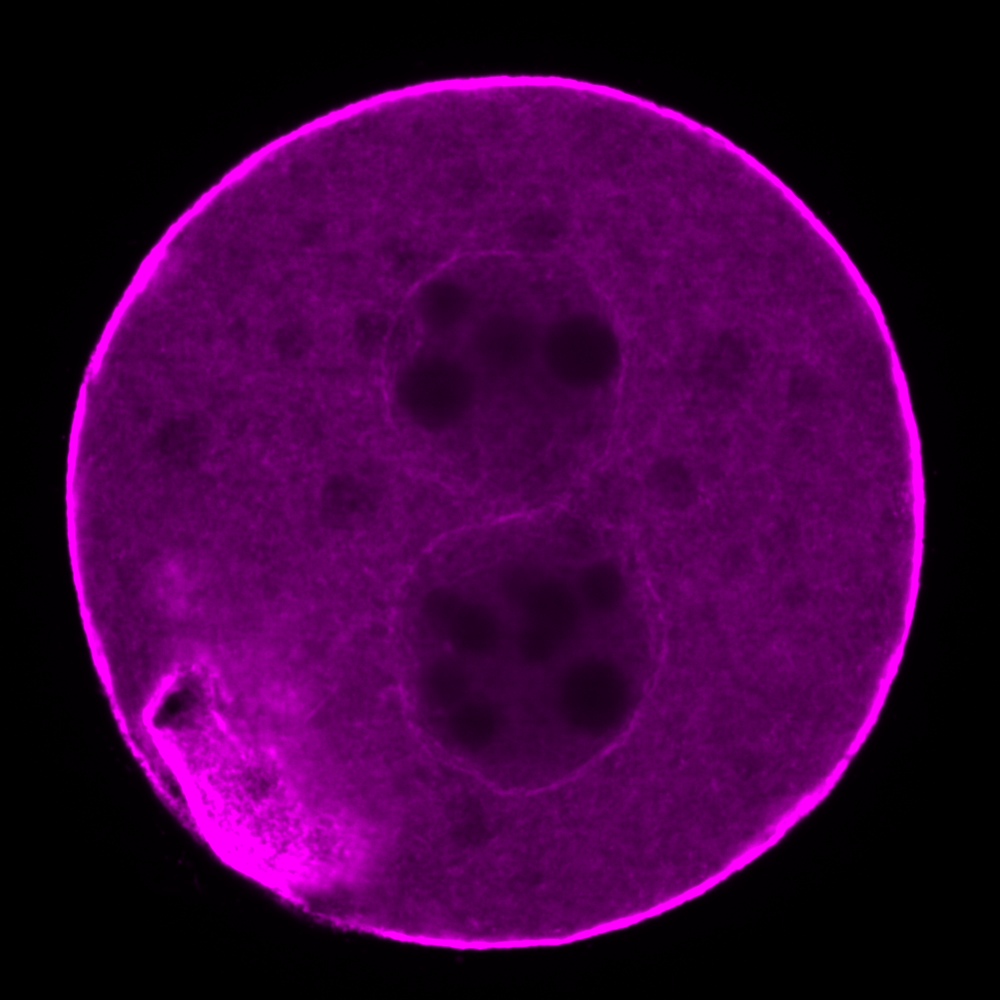

Supplement: Supplementary file 4 — Source data Fig. 2 [file 44319_2024_267_MOESM4_ESM.zip › Figure 2/2C/IF-F441-2-Factin.png]

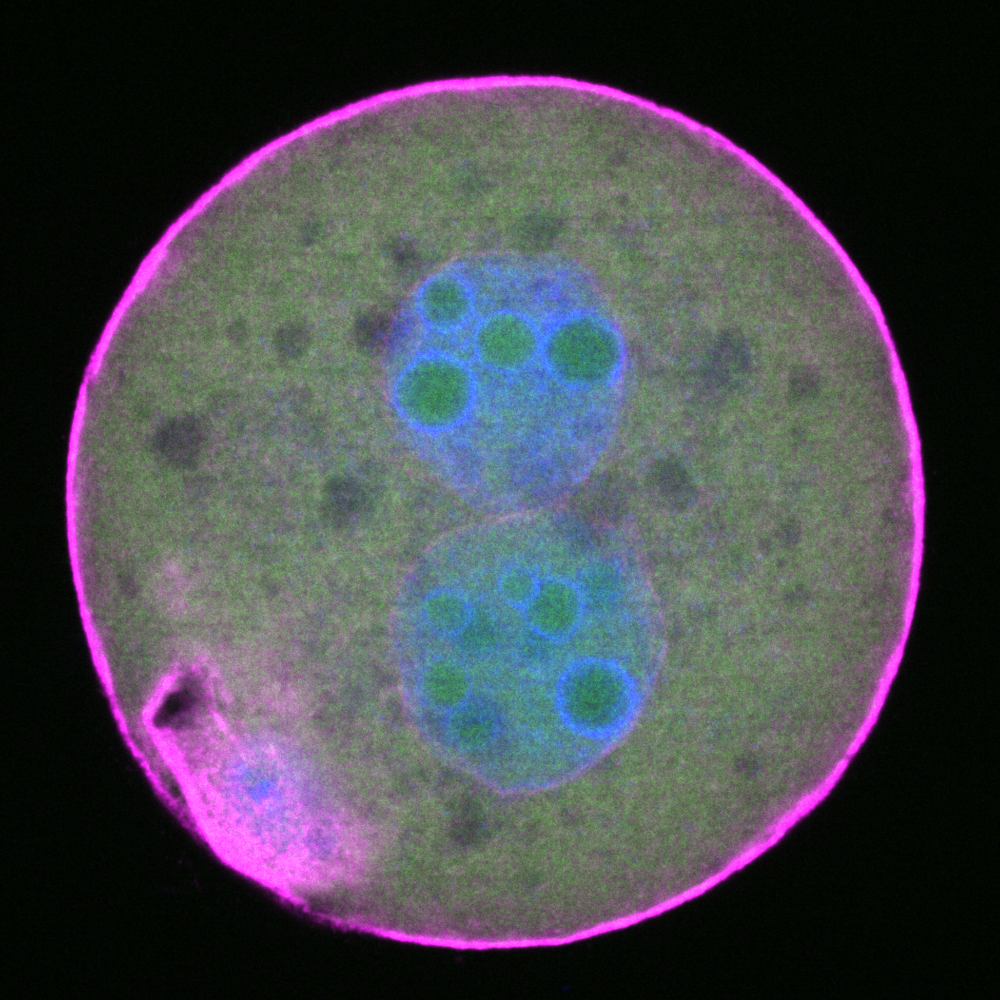

Supplement: Supplementary file 4 — Source data Fig. 2 [file 44319_2024_267_MOESM4_ESM.zip › Figure 2/2C/IF-F441-2-Merge.png]

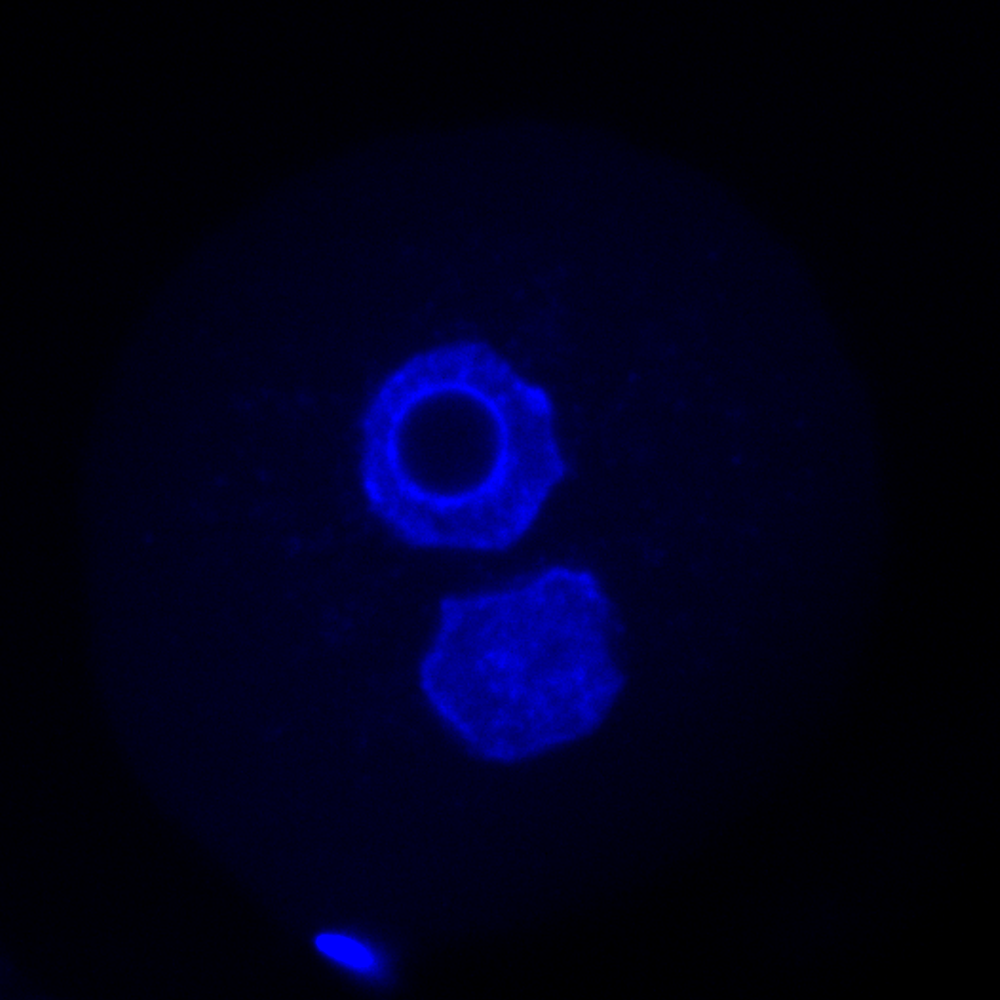

Supplement: Supplementary file 4 — Source data Fig. 2 [file 44319_2024_267_MOESM4_ESM.zip › Figure 2/2C/IF-WT-1-DAPI.png]

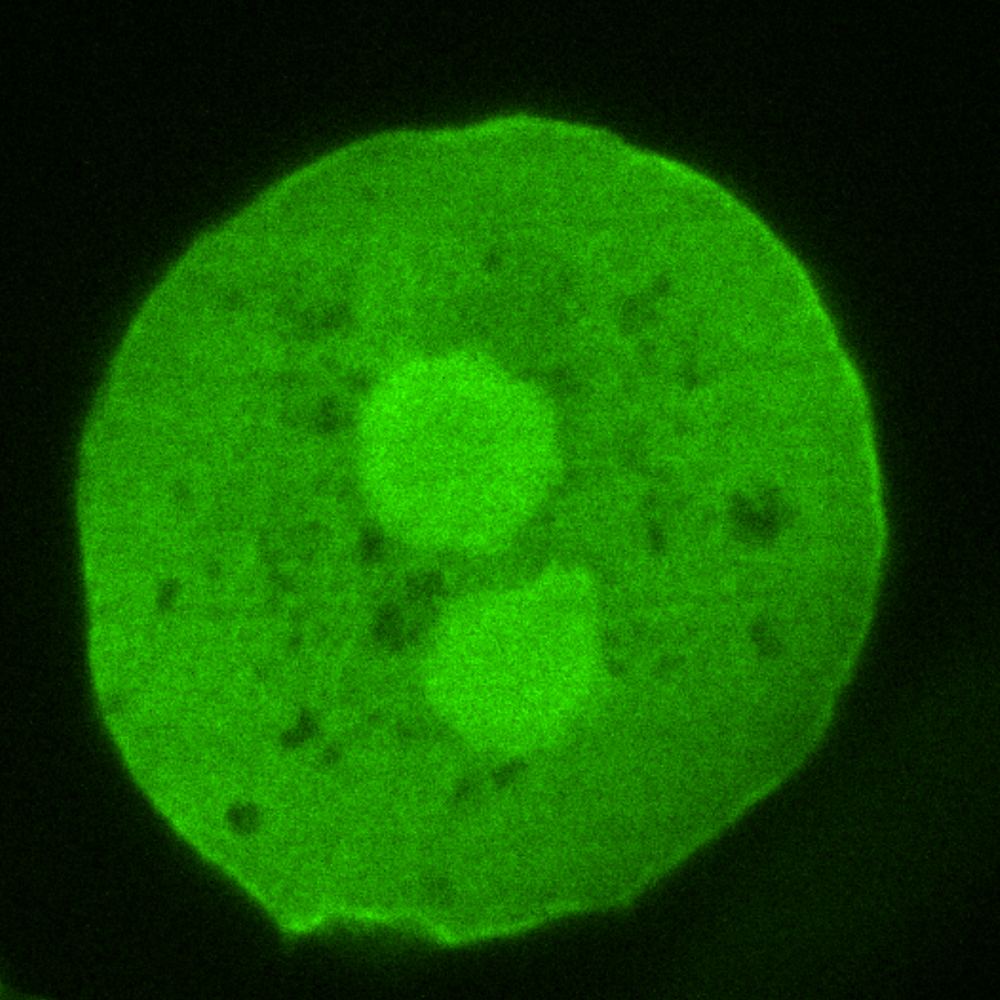

Supplement: Supplementary file 4 — Source data Fig. 2 [file 44319_2024_267_MOESM4_ESM.zip › Figure 2/2C/IF-WT-1-EGFP.png]

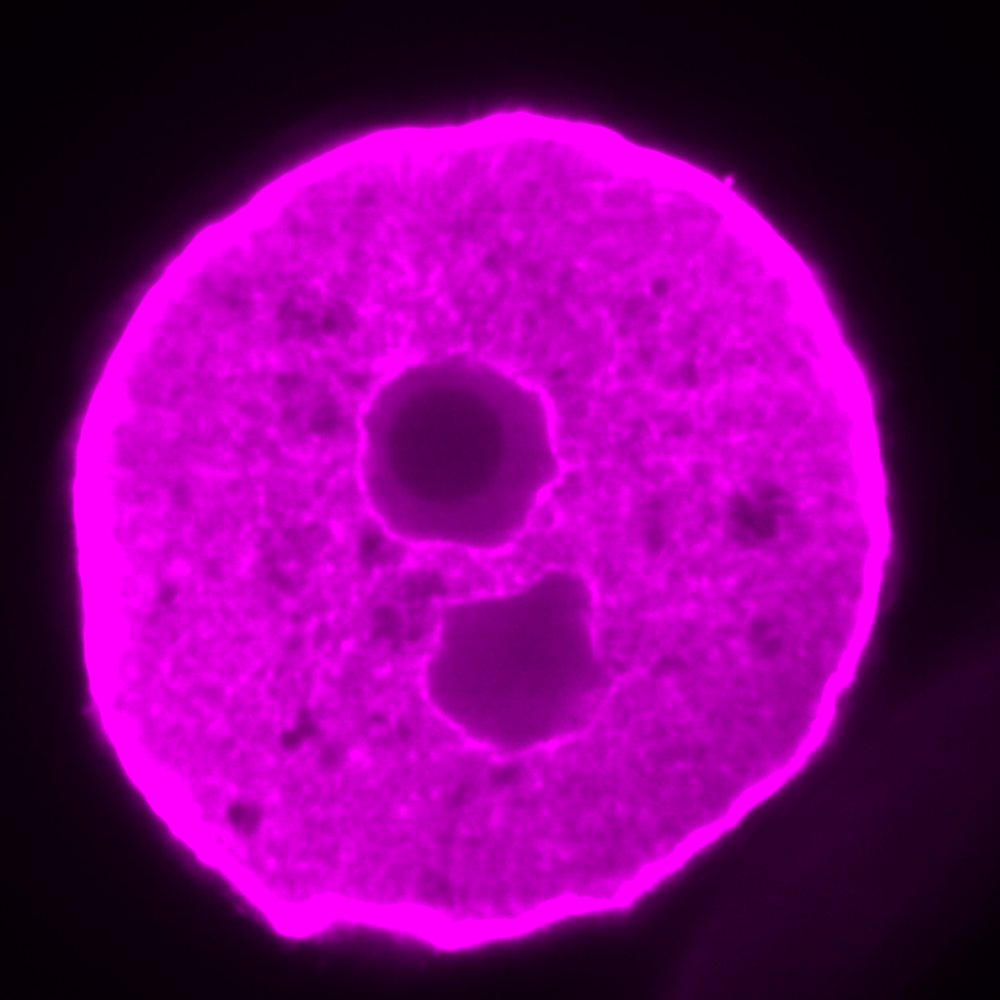

Supplement: Supplementary file 4 — Source data Fig. 2 [file 44319_2024_267_MOESM4_ESM.zip › Figure 2/2C/IF-WT-1-Factin.png]

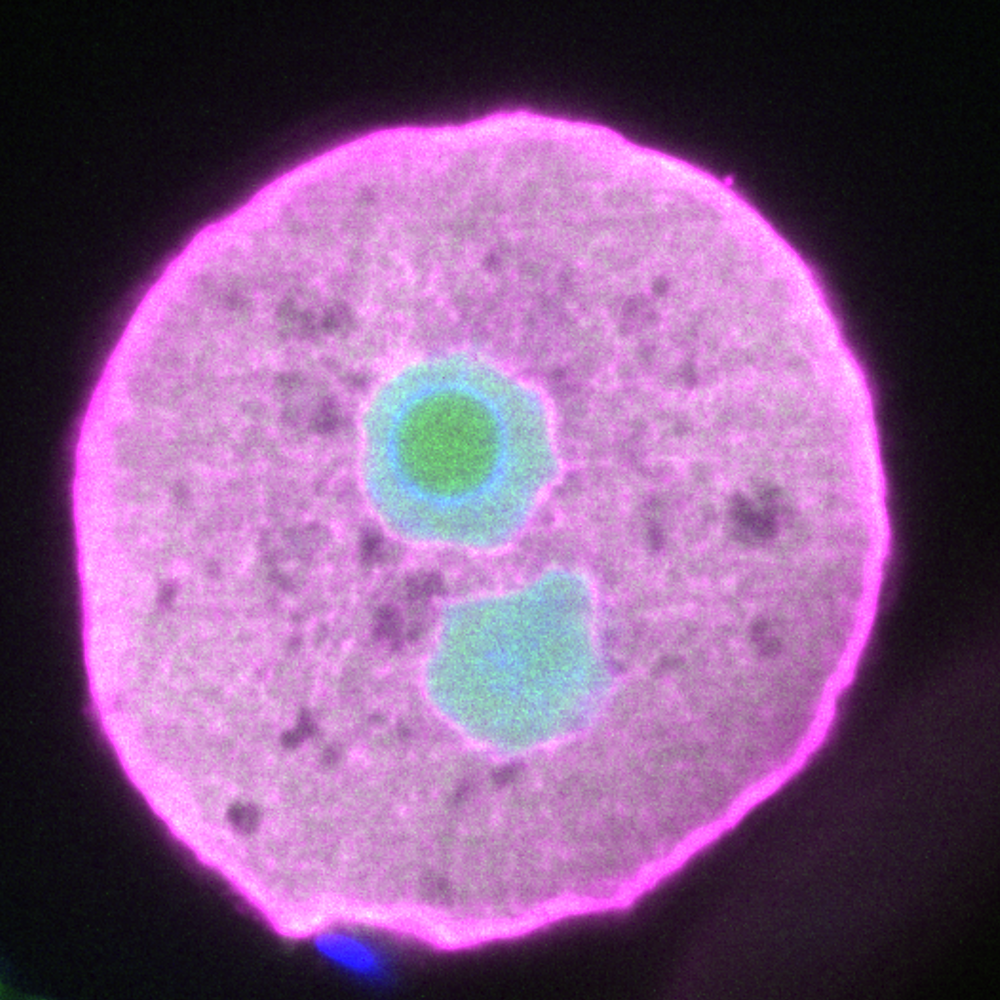

Supplement: Supplementary file 4 — Source data Fig. 2 [file 44319_2024_267_MOESM4_ESM.zip › Figure 2/2C/IF-WT-1-Merge.png]

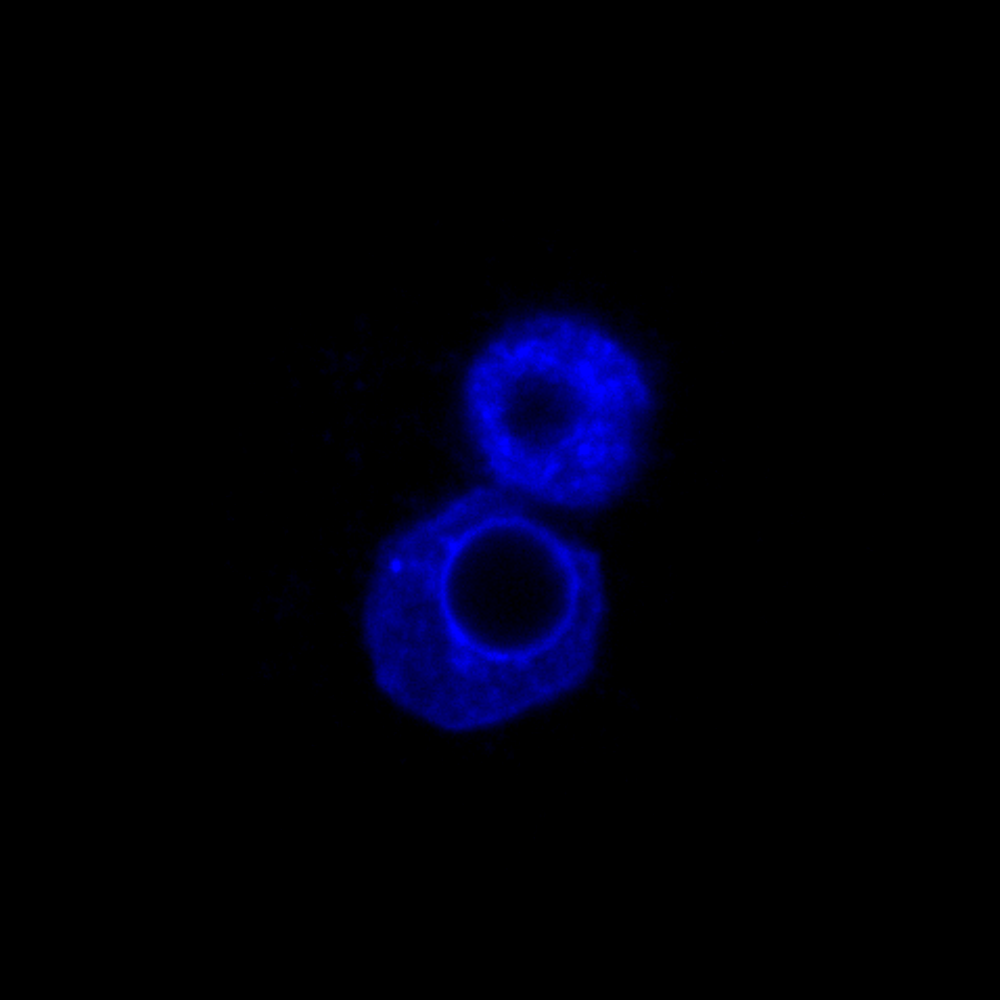

Supplement: Supplementary file 4 — Source data Fig. 2 [file 44319_2024_267_MOESM4_ESM.zip › Figure 2/2C/IF-WT-2-DAPI.png]

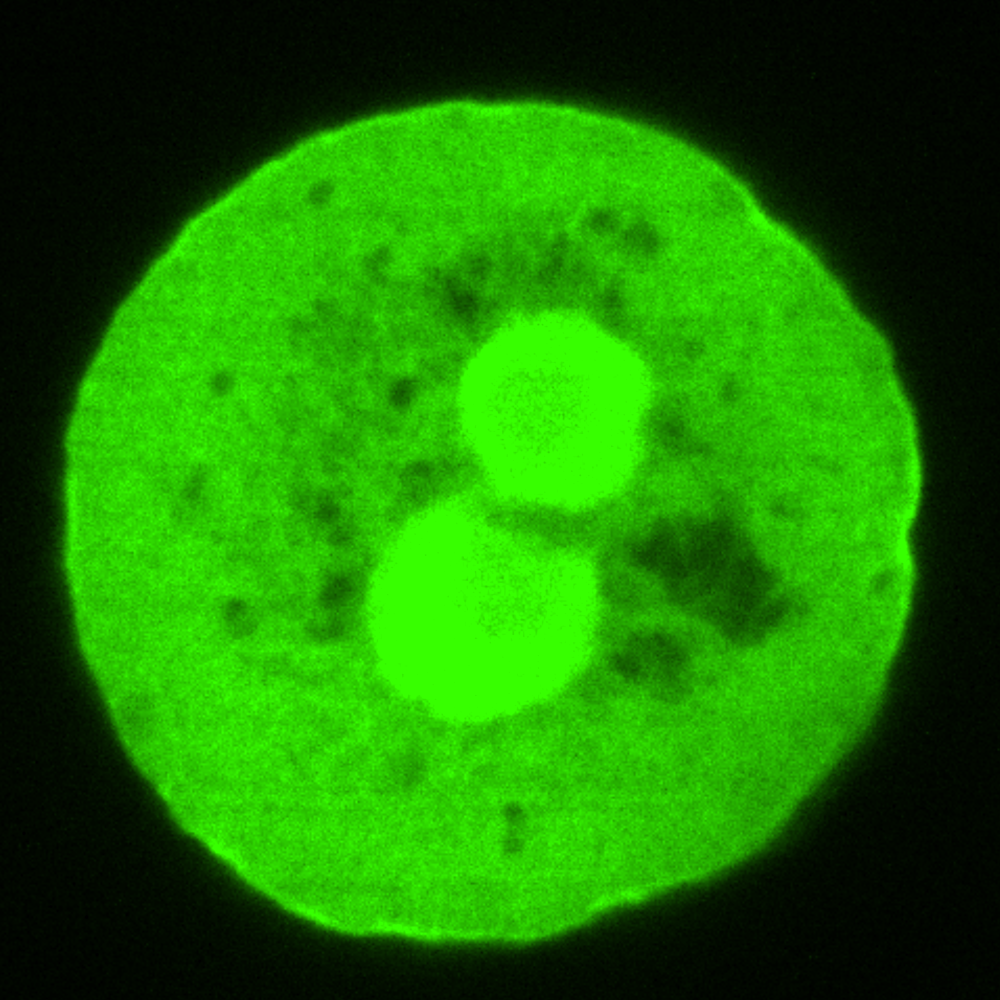

Supplement: Supplementary file 4 — Source data Fig. 2 [file 44319_2024_267_MOESM4_ESM.zip › Figure 2/2C/IF-WT-2-EGFP.png]

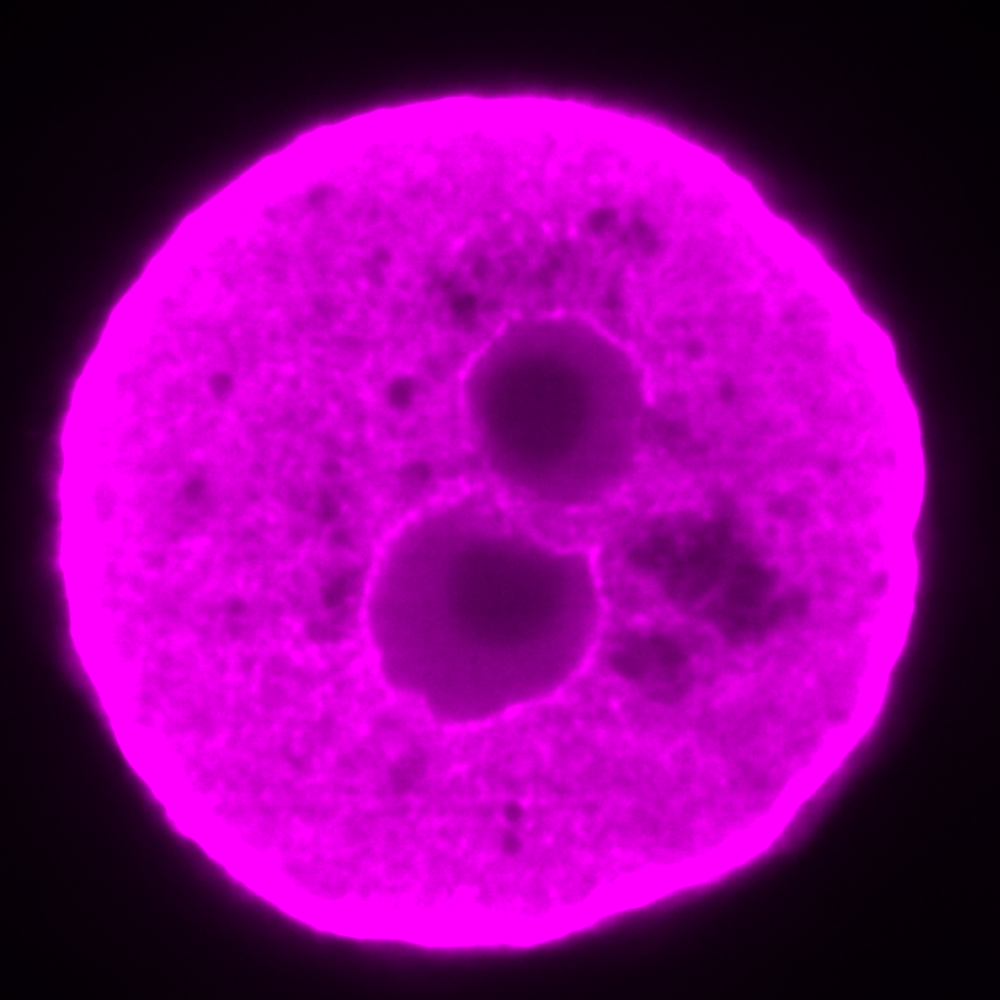

Supplement: Supplementary file 4 — Source data Fig. 2 [file 44319_2024_267_MOESM4_ESM.zip › Figure 2/2C/IF-WT-2-Factin.png]

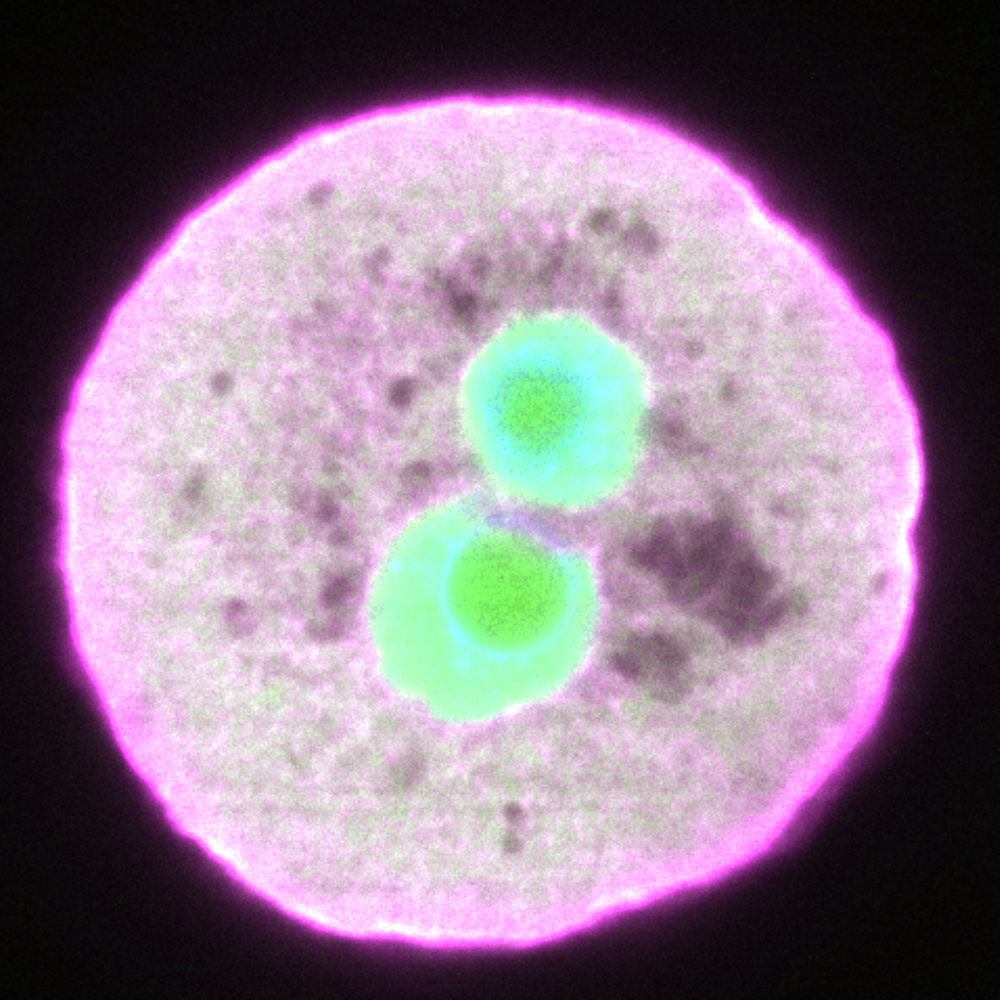

Supplement: Supplementary file 4 — Source data Fig. 2 [file 44319_2024_267_MOESM4_ESM.zip › Figure 2/2C/IF-WT-2-Merge.png]

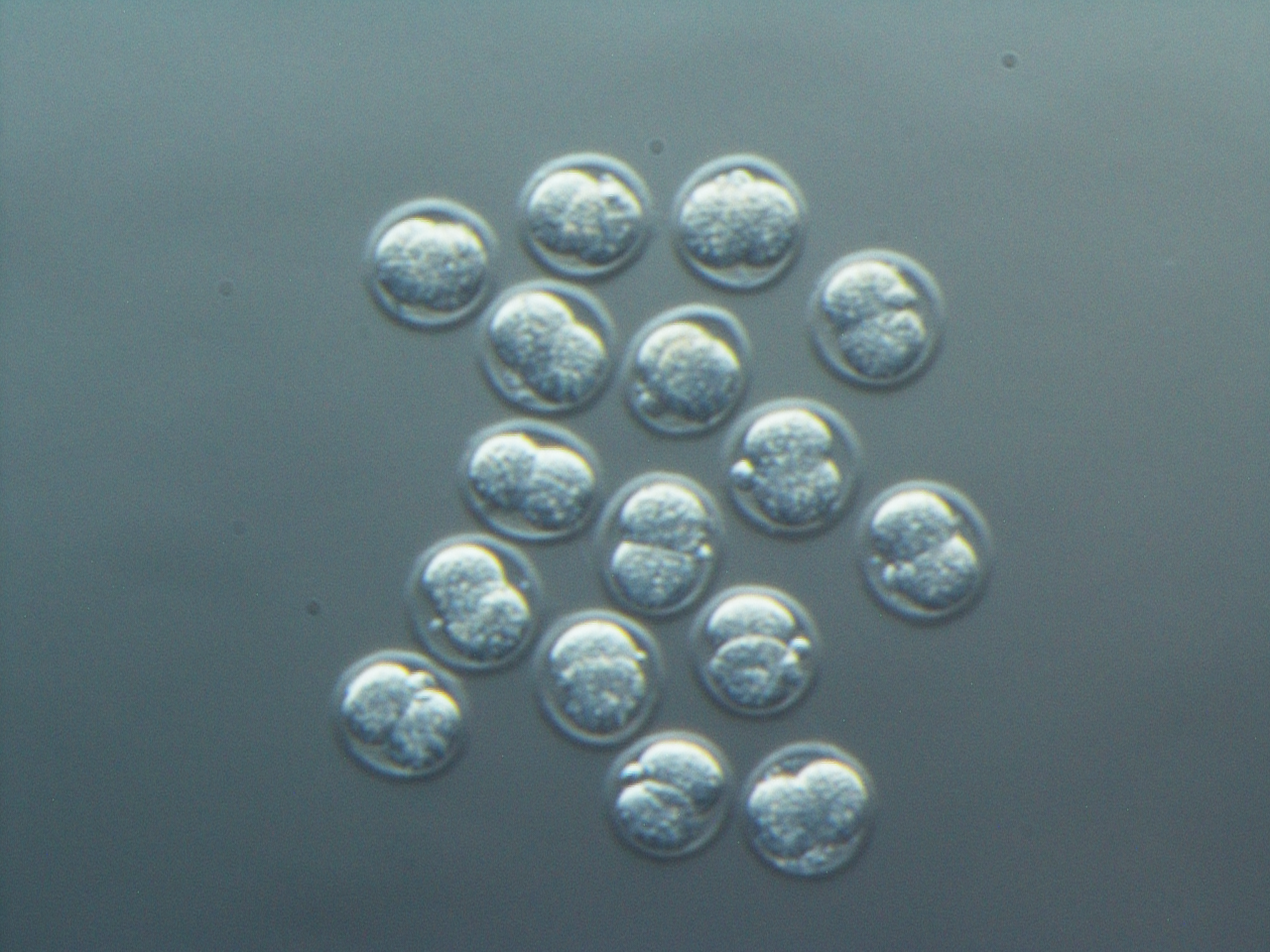

Supplement: Supplementary file 5 — Source data Fig. 3 [file 44319_2024_267_MOESM5_ESM.zip › Figure 3/3B/Control.tif]

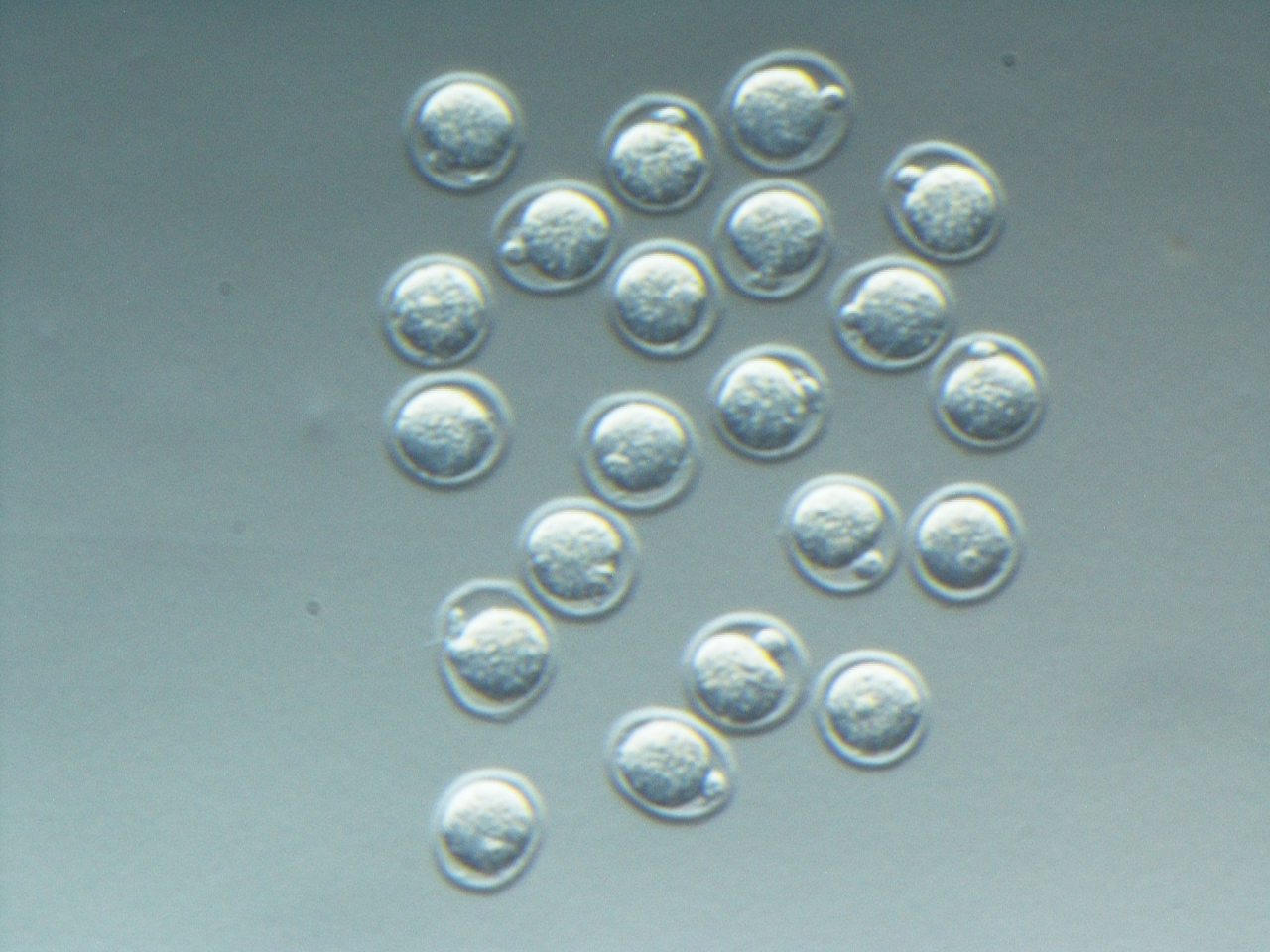

Supplement: Supplementary file 5 — Source data Fig. 3 [file 44319_2024_267_MOESM5_ESM.zip › Figure 3/3B/Cytochalasin B.tif]

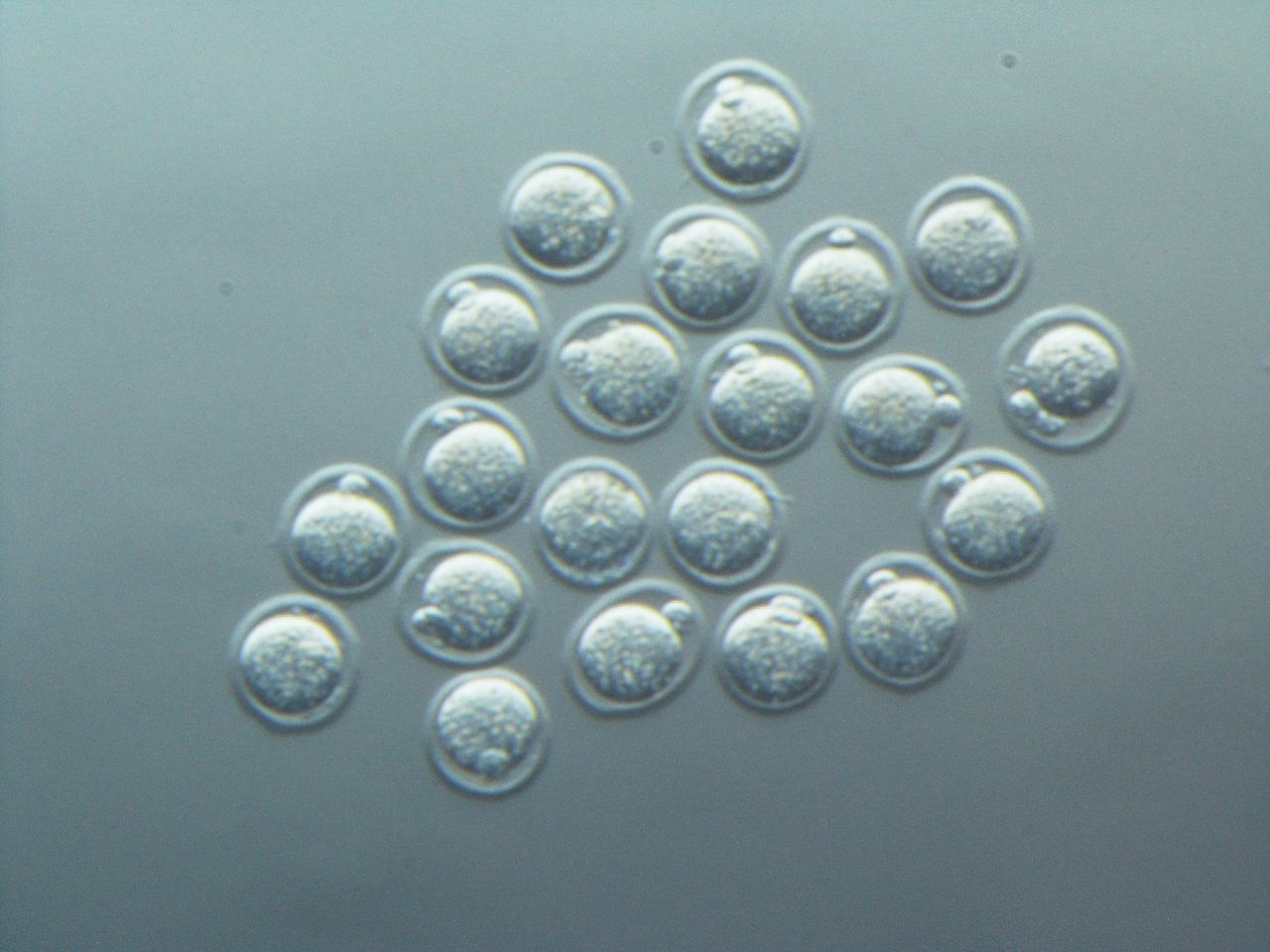

Supplement: Supplementary file 5 — Source data Fig. 3 [file 44319_2024_267_MOESM5_ESM.zip › Figure 3/3B/Nocodazole&Cytochalasin B.tif]

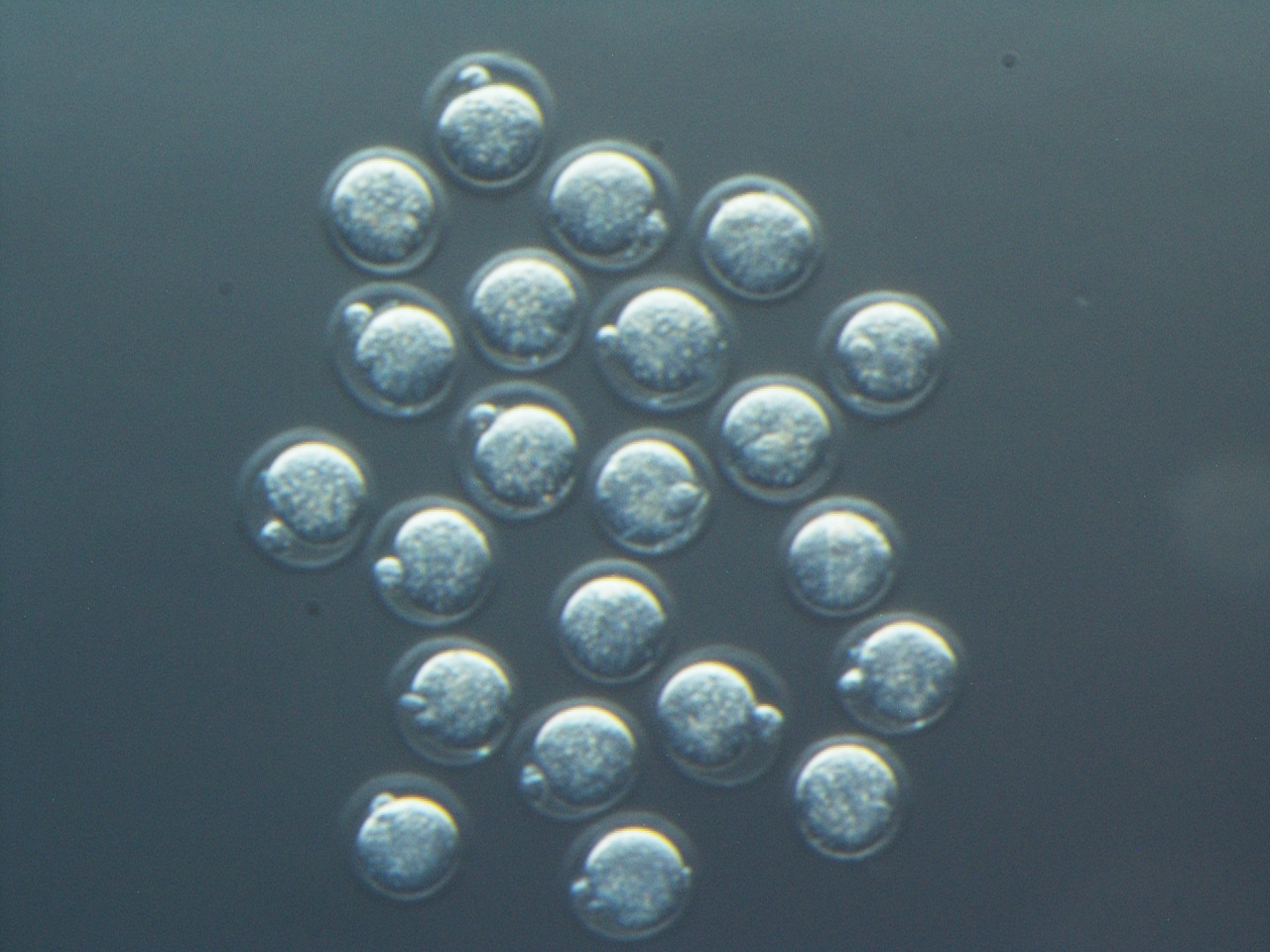

Supplement: Supplementary file 5 — Source data Fig. 3 [file 44319_2024_267_MOESM5_ESM.zip › Figure 3/3B/Nocodazole.tif]

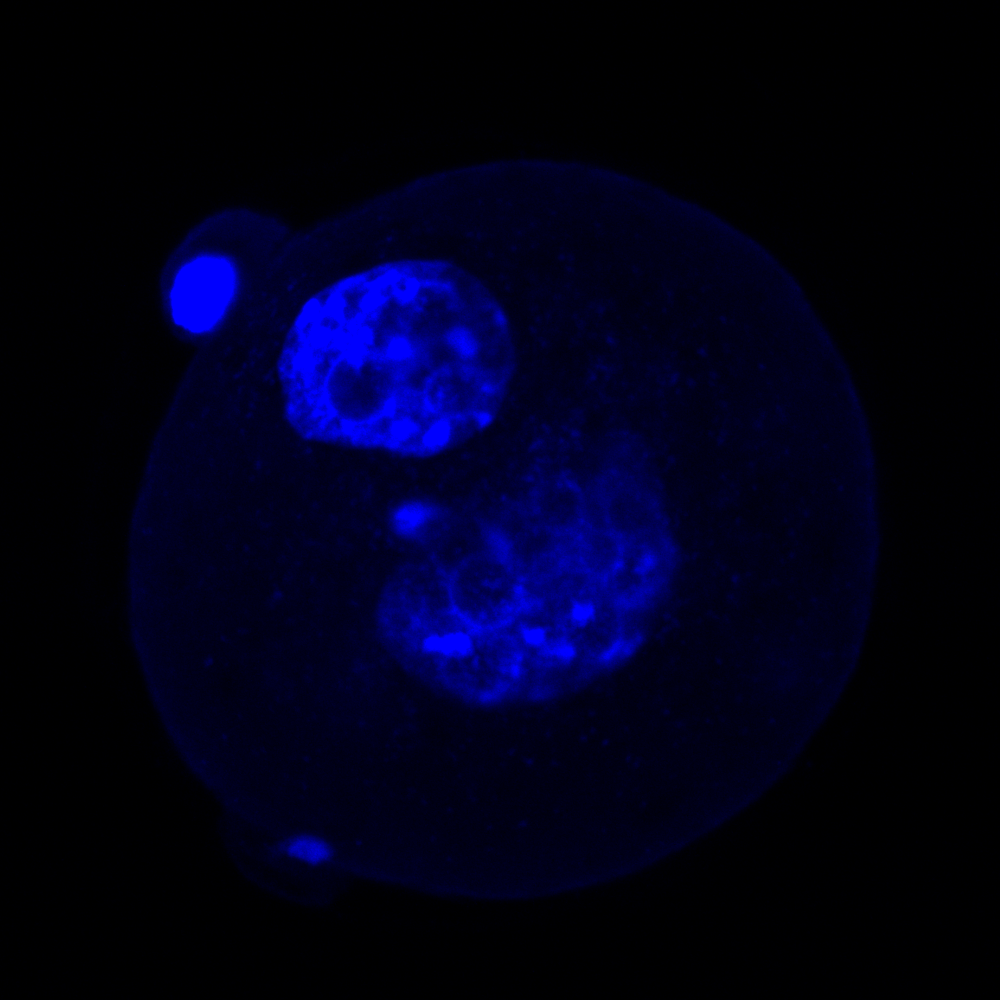

Supplement: Supplementary file 5 — Source data Fig. 3 [file 44319_2024_267_MOESM5_ESM.zip › Figure 3/3D/IF-CB-DAPI.png]

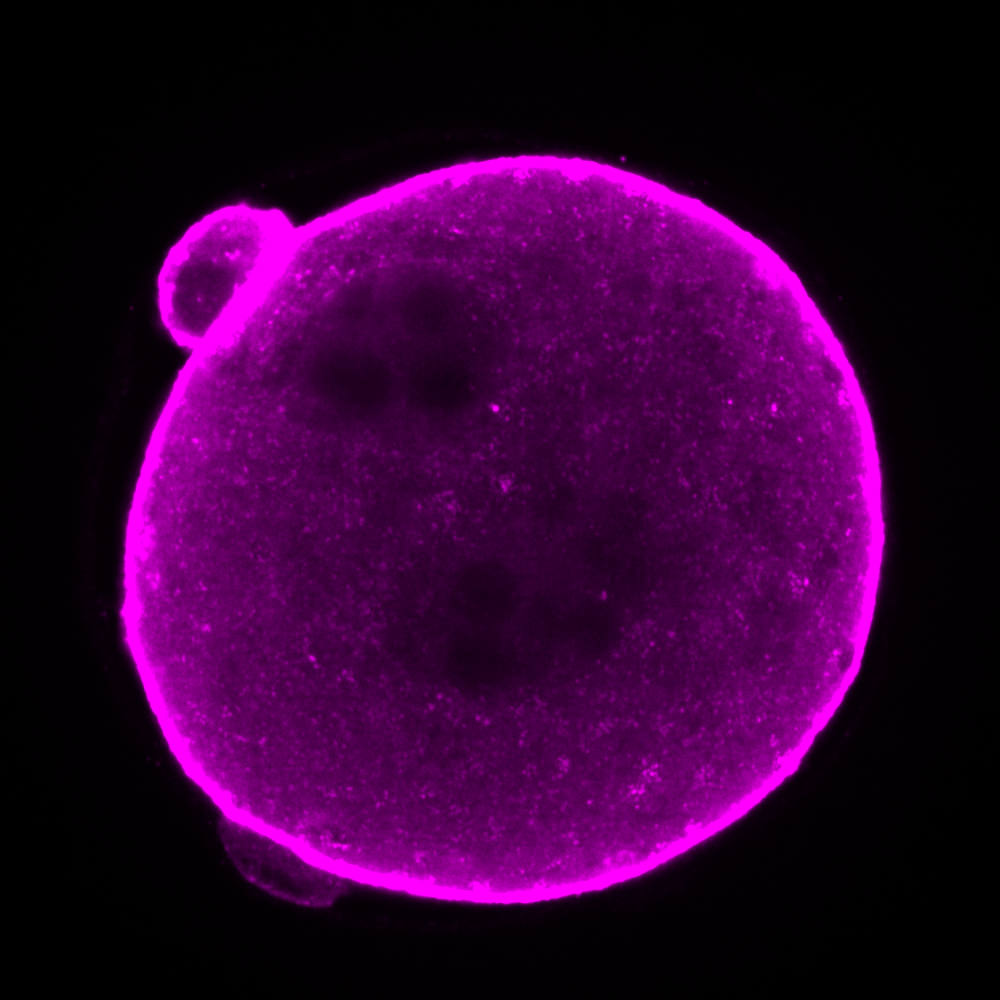

Supplement: Supplementary file 5 — Source data Fig. 3 [file 44319_2024_267_MOESM5_ESM.zip › Figure 3/3D/IF-CB-Factin.png]

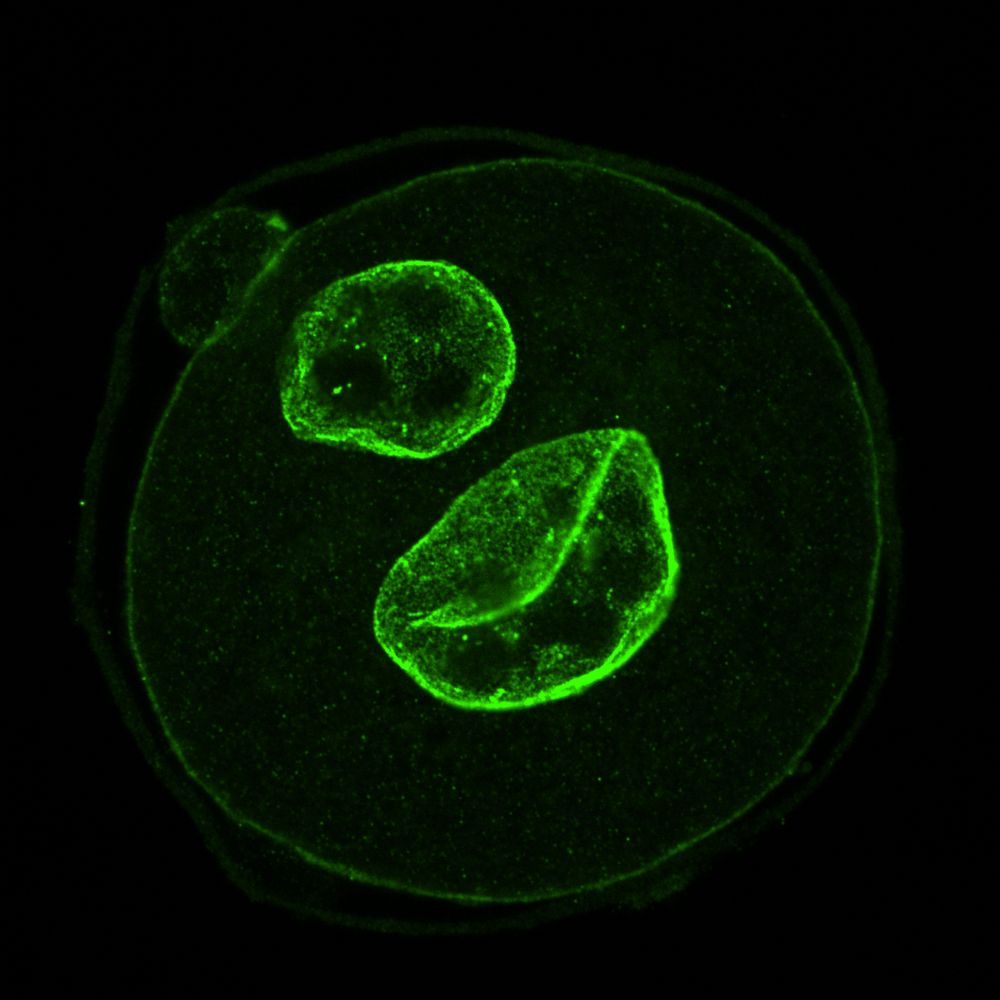

Supplement: Supplementary file 5 — Source data Fig. 3 [file 44319_2024_267_MOESM5_ESM.zip › Figure 3/3D/IF-CB-LaminB1.png]

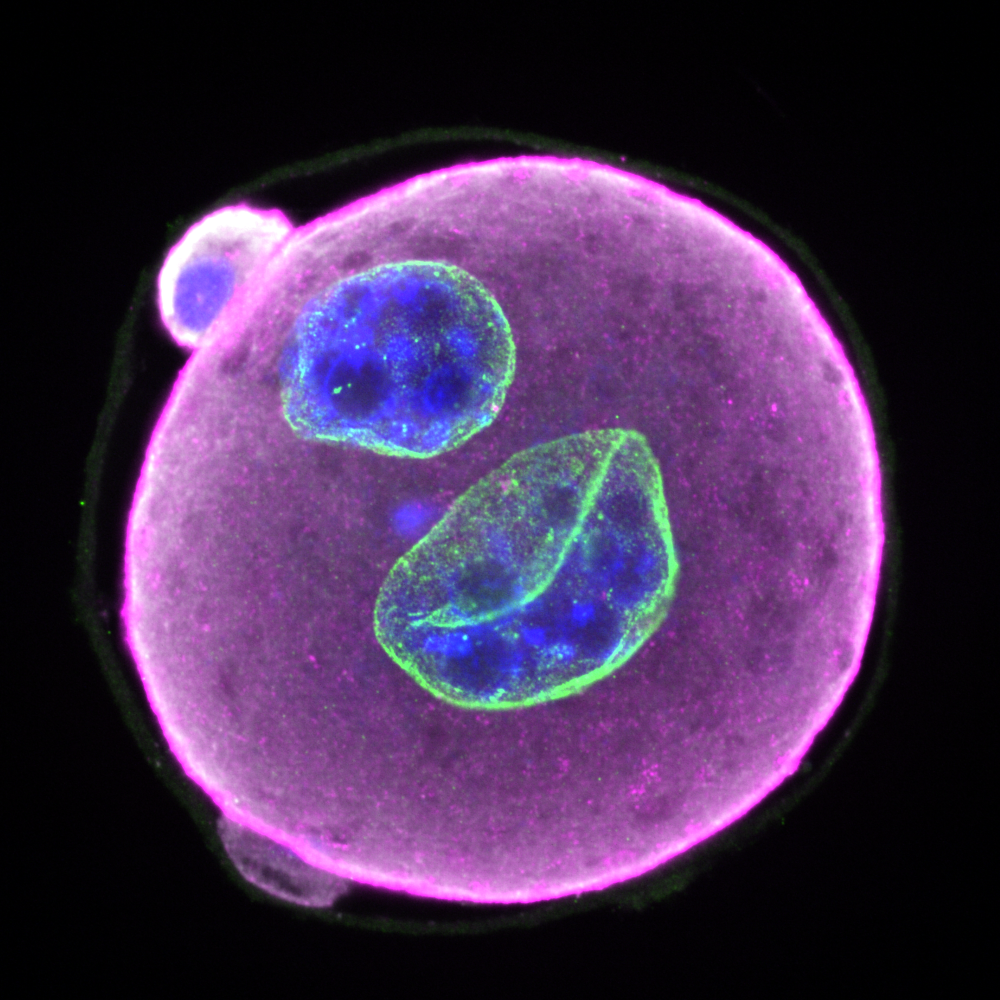

Supplement: Supplementary file 5 — Source data Fig. 3 [file 44319_2024_267_MOESM5_ESM.zip › Figure 3/3D/IF-CB-Merge.png]

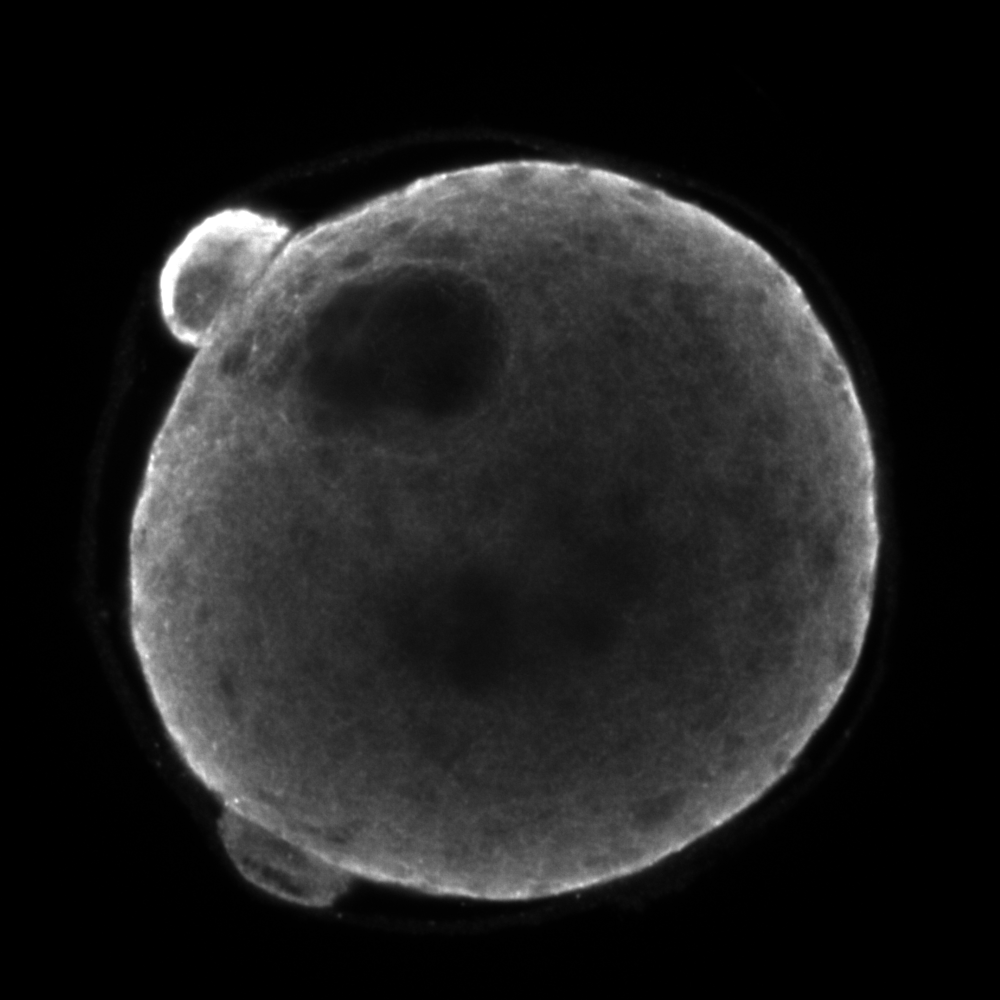

Supplement: Supplementary file 5 — Source data Fig. 3 [file 44319_2024_267_MOESM5_ESM.zip › Figure 3/3D/IF-CB-αTubulin.png]

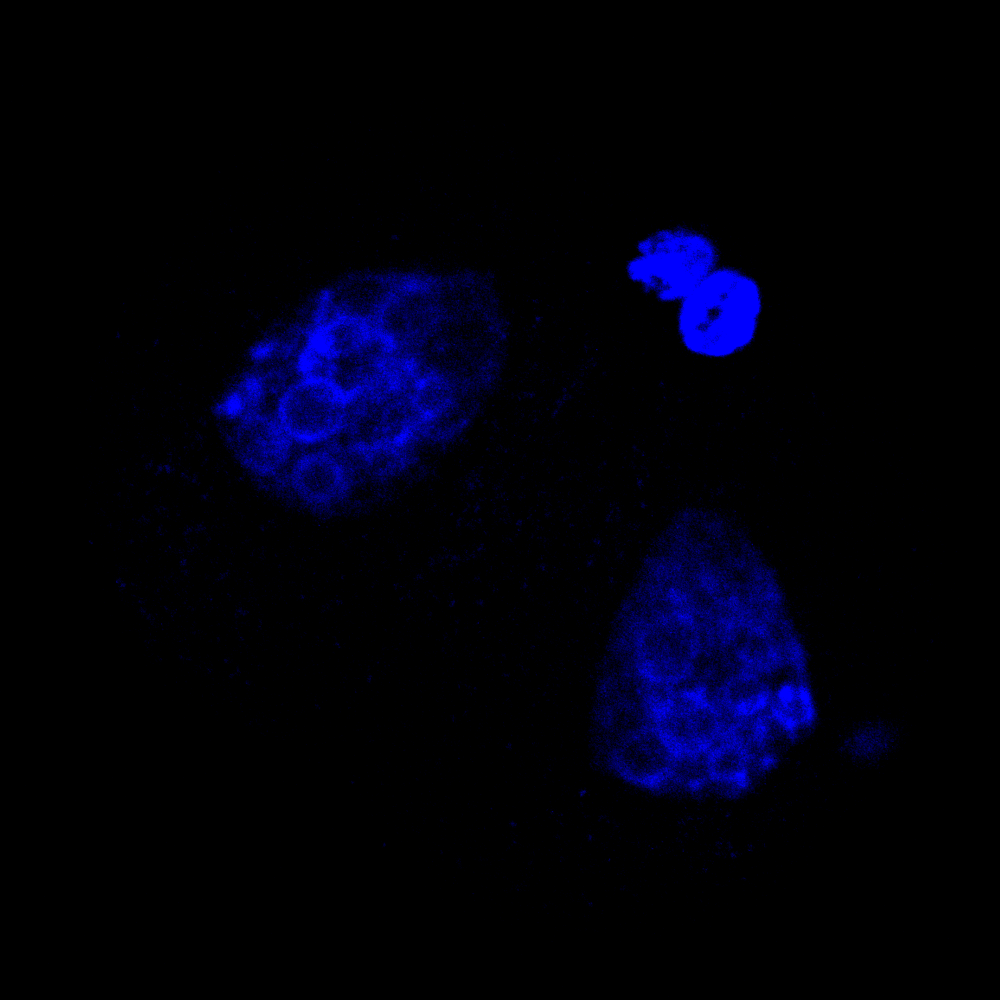

Supplement: Supplementary file 5 — Source data Fig. 3 [file 44319_2024_267_MOESM5_ESM.zip › Figure 3/3D/IF-Control-DAPI.png]

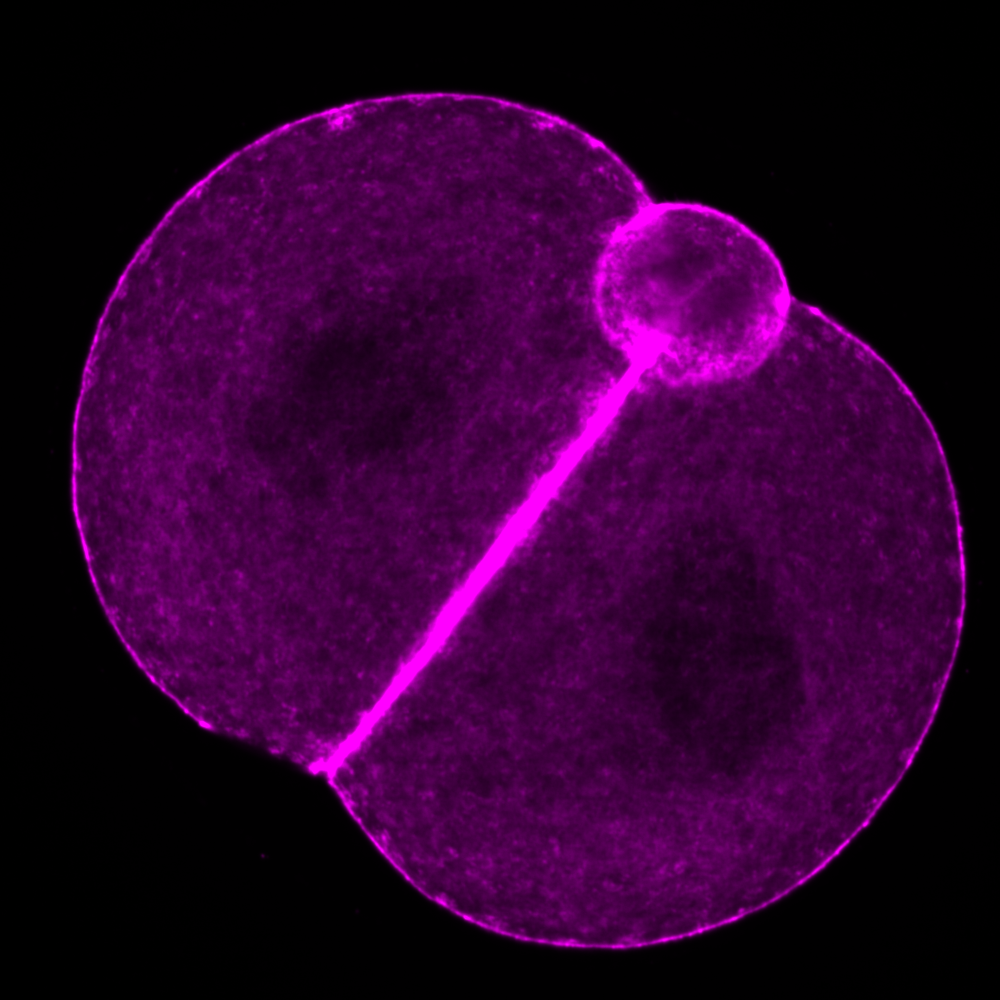

Supplement: Supplementary file 5 — Source data Fig. 3 [file 44319_2024_267_MOESM5_ESM.zip › Figure 3/3D/IF-Control-Factin.png]

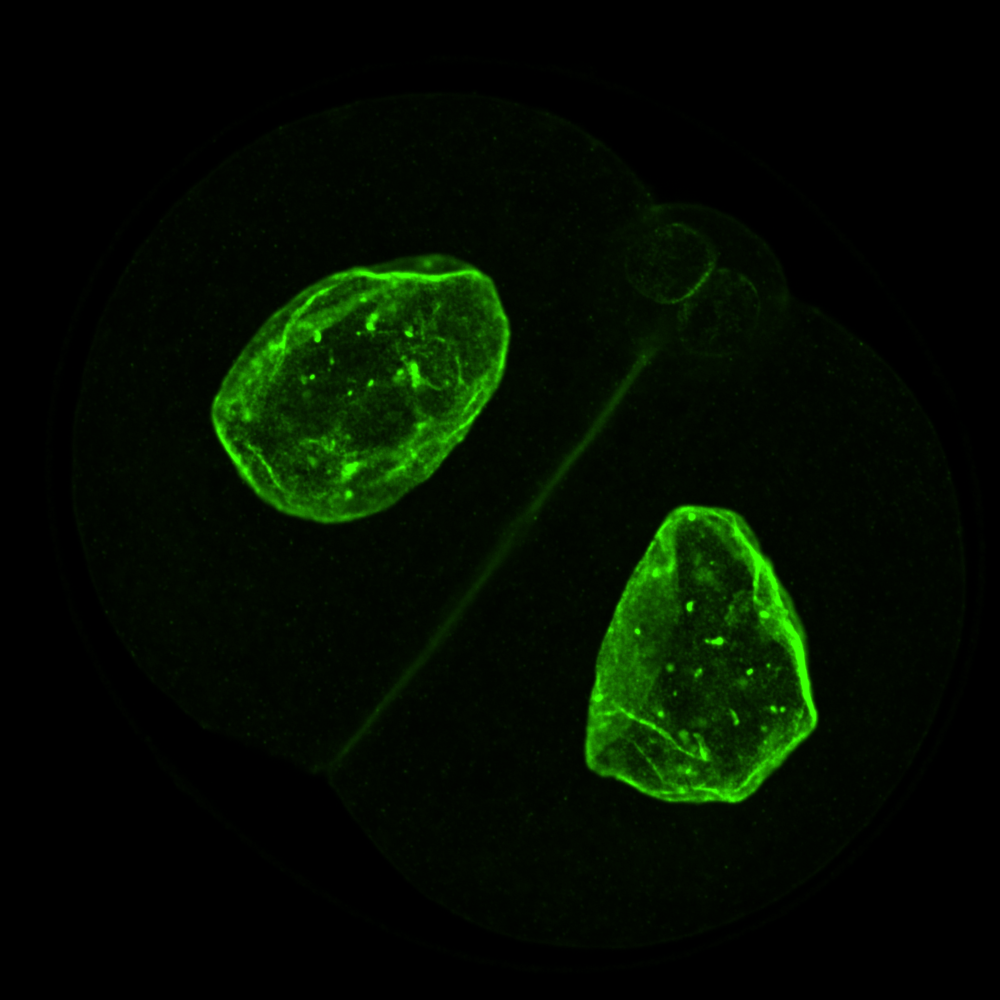

Supplement: Supplementary file 5 — Source data Fig. 3 [file 44319_2024_267_MOESM5_ESM.zip › Figure 3/3D/IF-Control-LaminB1.png]

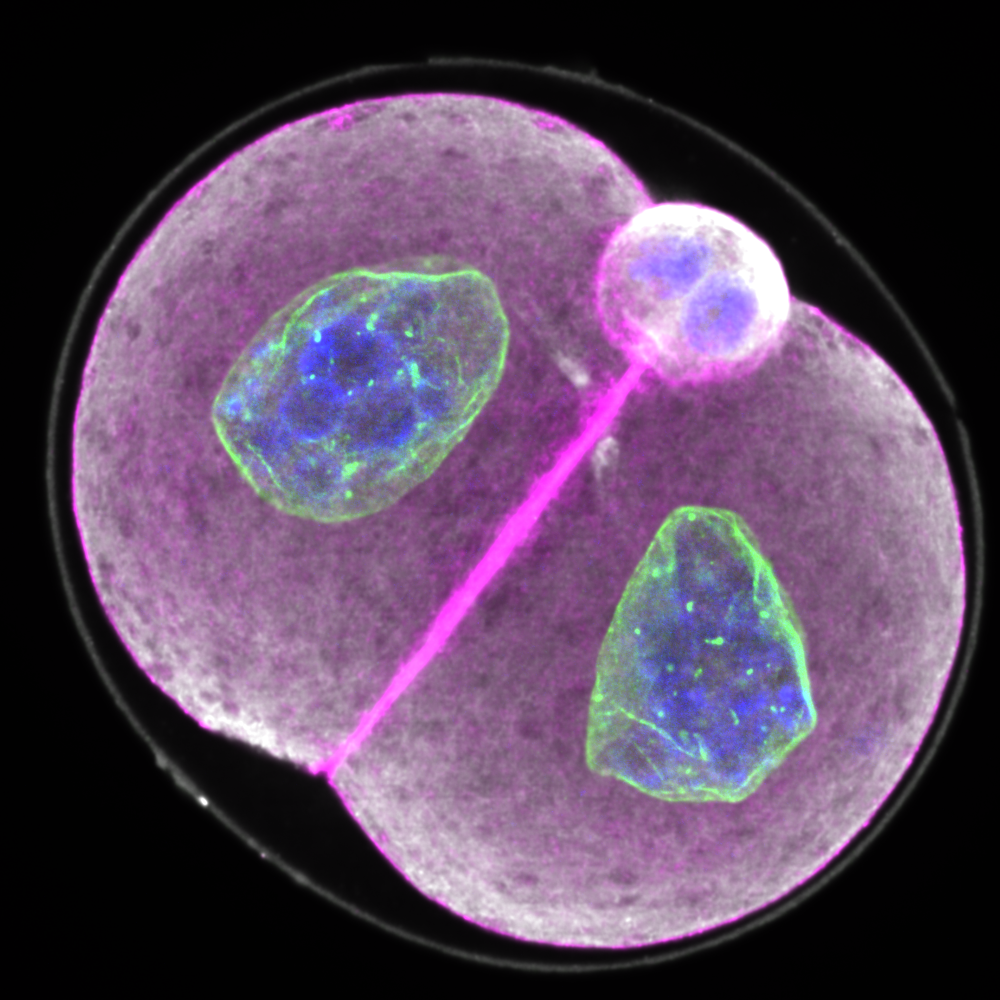

Supplement: Supplementary file 5 — Source data Fig. 3 [file 44319_2024_267_MOESM5_ESM.zip › Figure 3/3D/IF-Control-Merge.png]

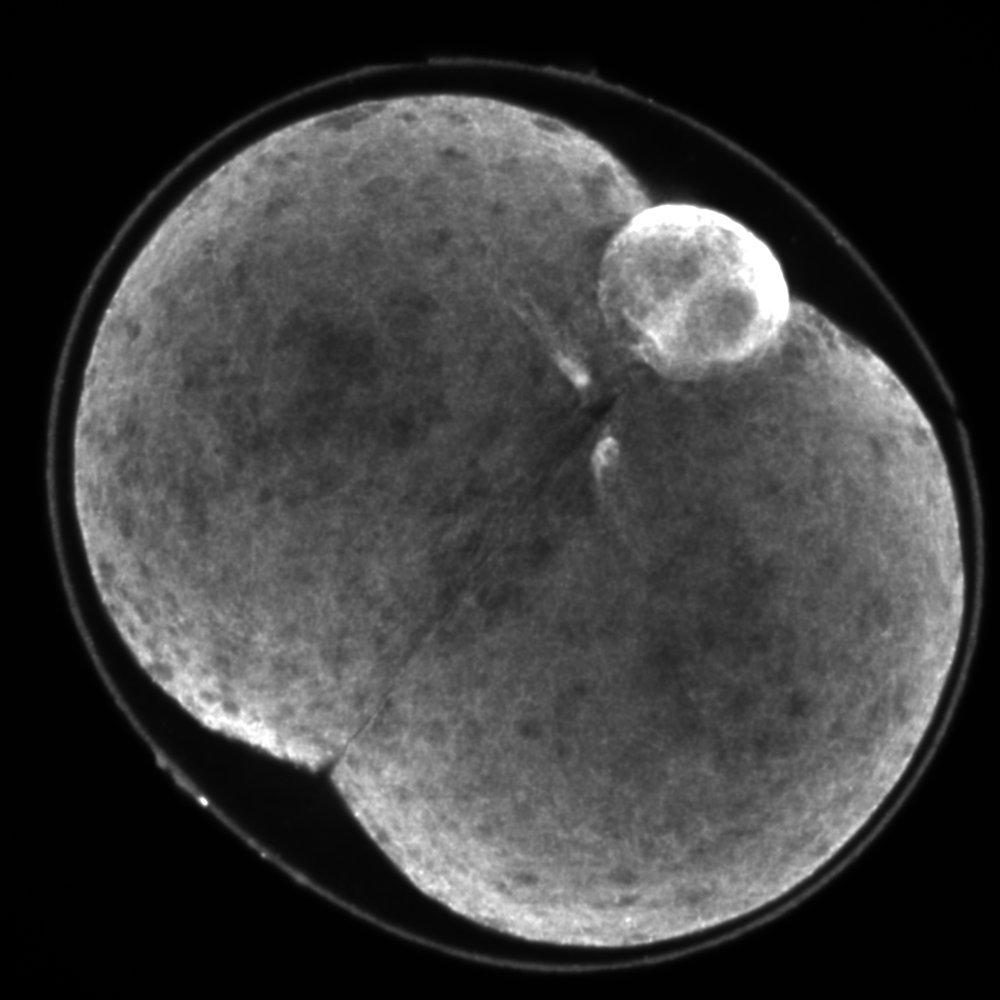

Supplement: Supplementary file 5 — Source data Fig. 3 [file 44319_2024_267_MOESM5_ESM.zip › Figure 3/3D/IF-Control-αTubulin.png]

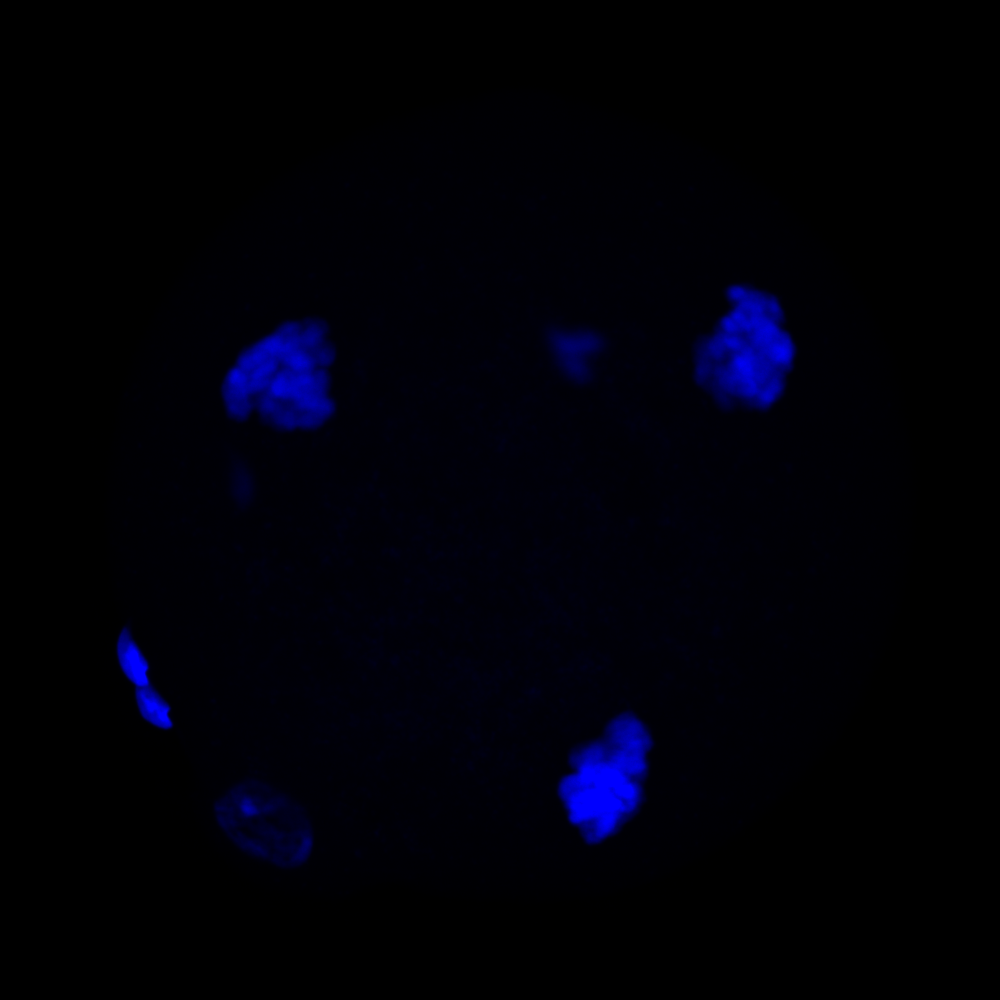

Supplement: Supplementary file 5 — Source data Fig. 3 [file 44319_2024_267_MOESM5_ESM.zip › Figure 3/3D/IF-Noco&CB-DAPI.png]

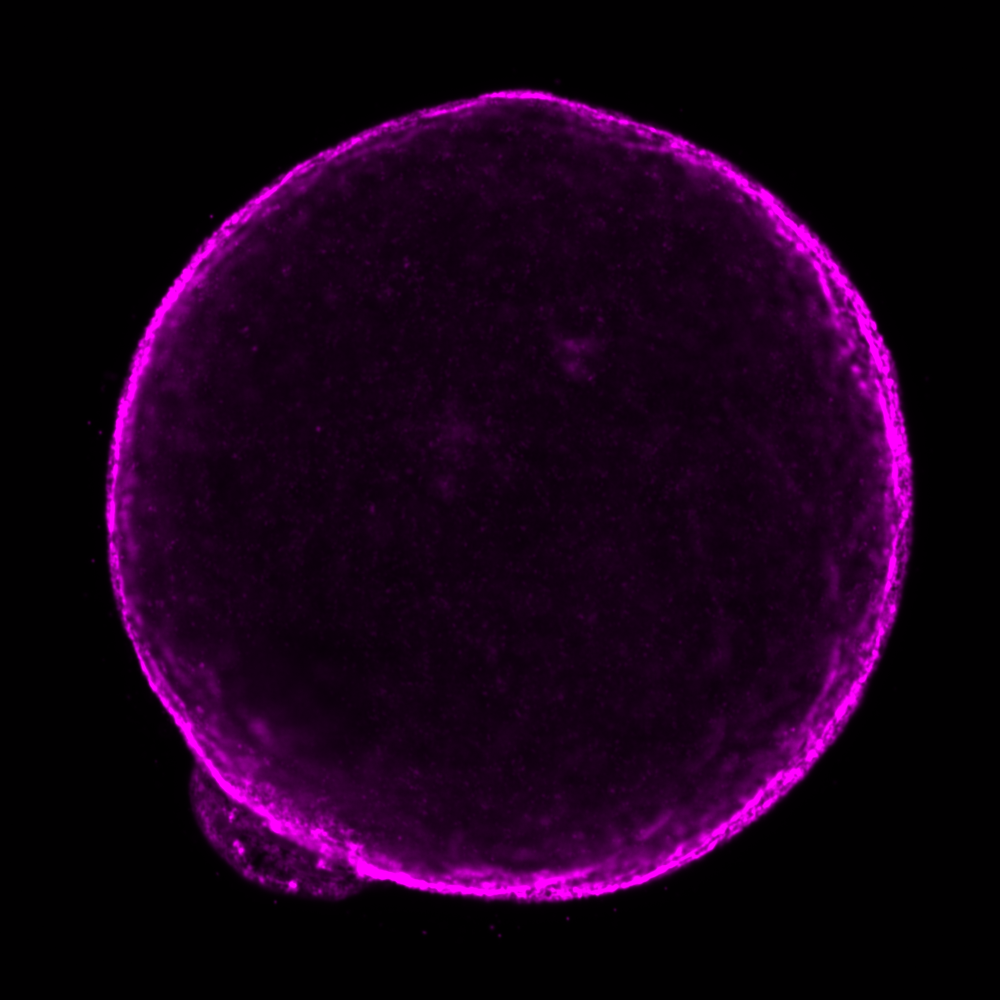

Supplement: Supplementary file 5 — Source data Fig. 3 [file 44319_2024_267_MOESM5_ESM.zip › Figure 3/3D/IF-Noco&CB-Factin.png]

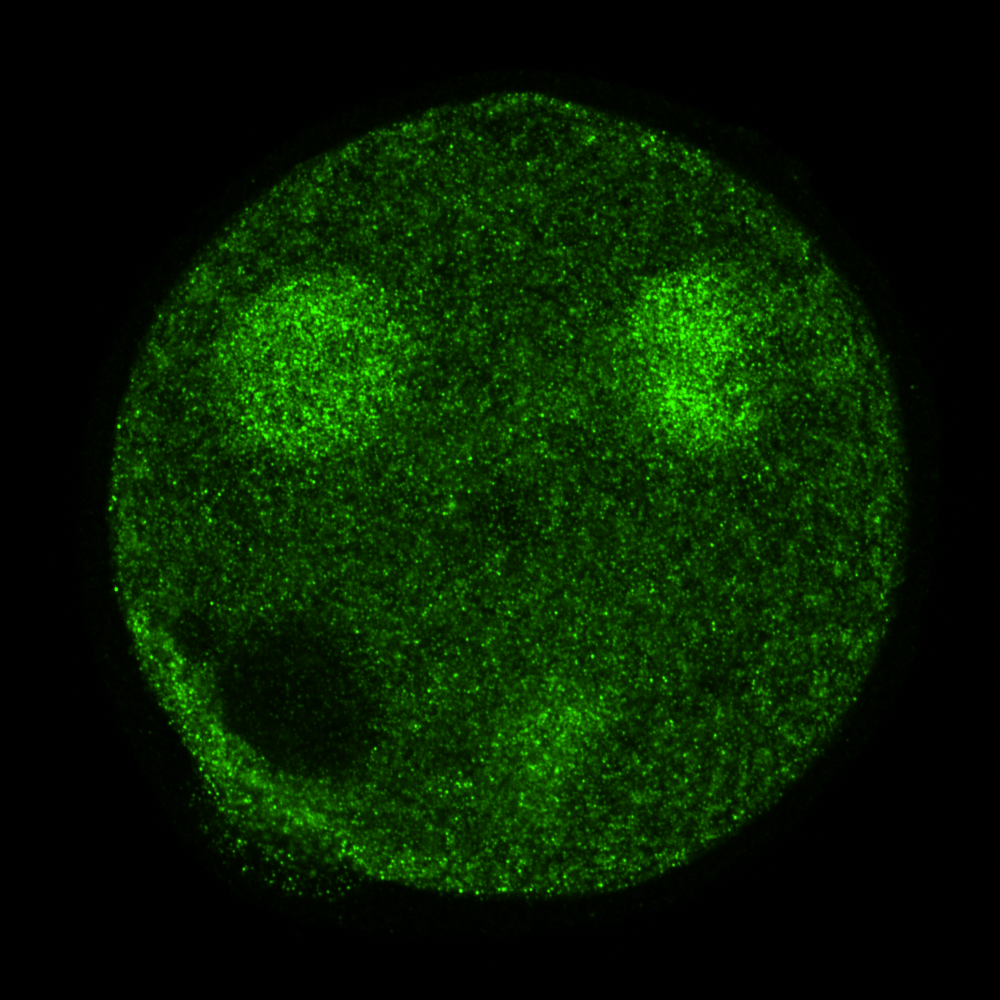

Supplement: Supplementary file 5 — Source data Fig. 3 [file 44319_2024_267_MOESM5_ESM.zip › Figure 3/3D/IF-Noco&CB-LaminB1.png]

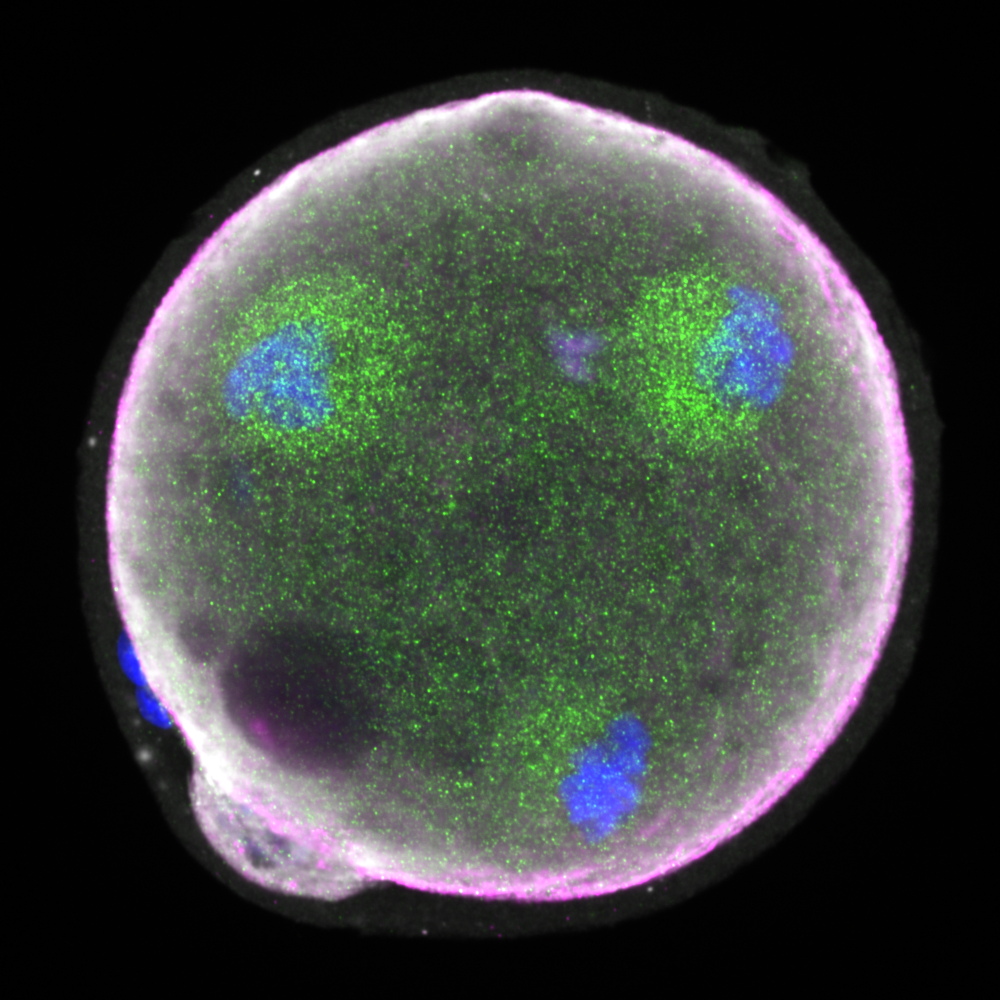

Supplement: Supplementary file 5 — Source data Fig. 3 [file 44319_2024_267_MOESM5_ESM.zip › Figure 3/3D/IF-Noco&CB-Merge.png]

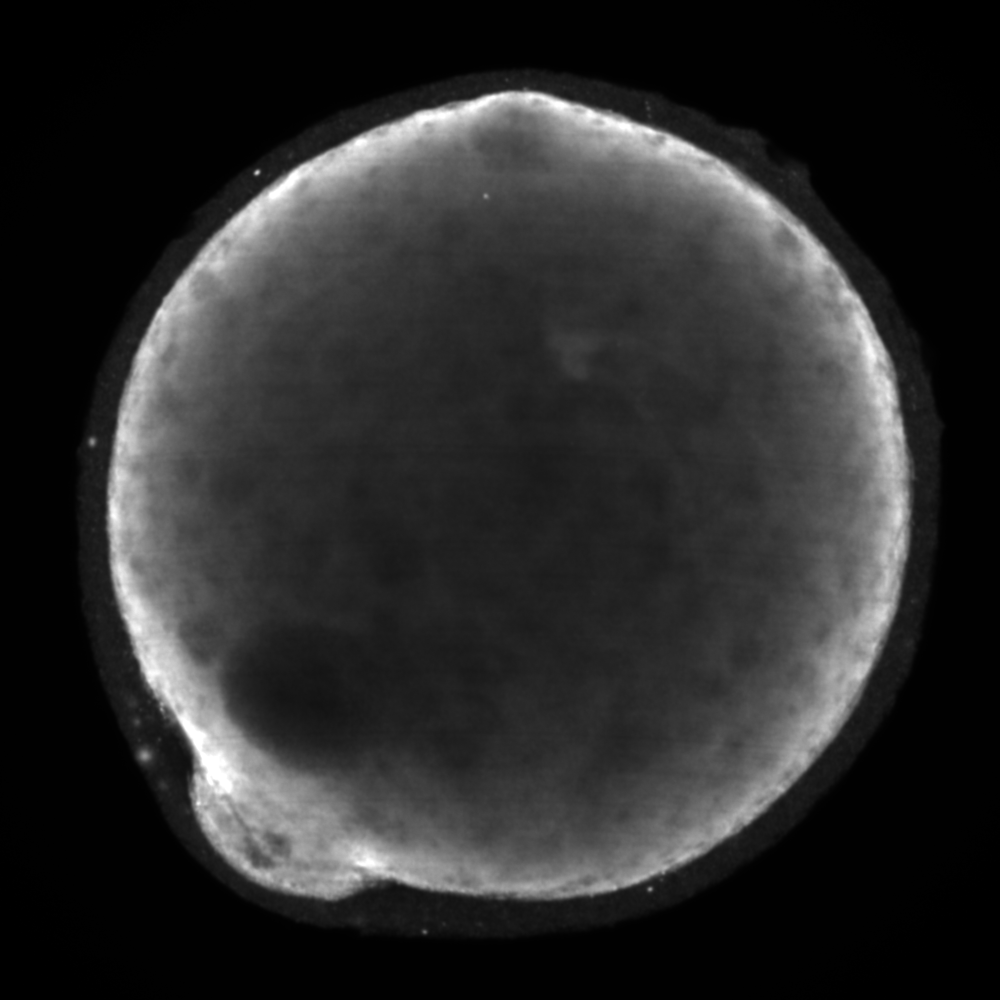

Supplement: Supplementary file 5 — Source data Fig. 3 [file 44319_2024_267_MOESM5_ESM.zip › Figure 3/3D/IF-Noco&CB-αTubulin.png]

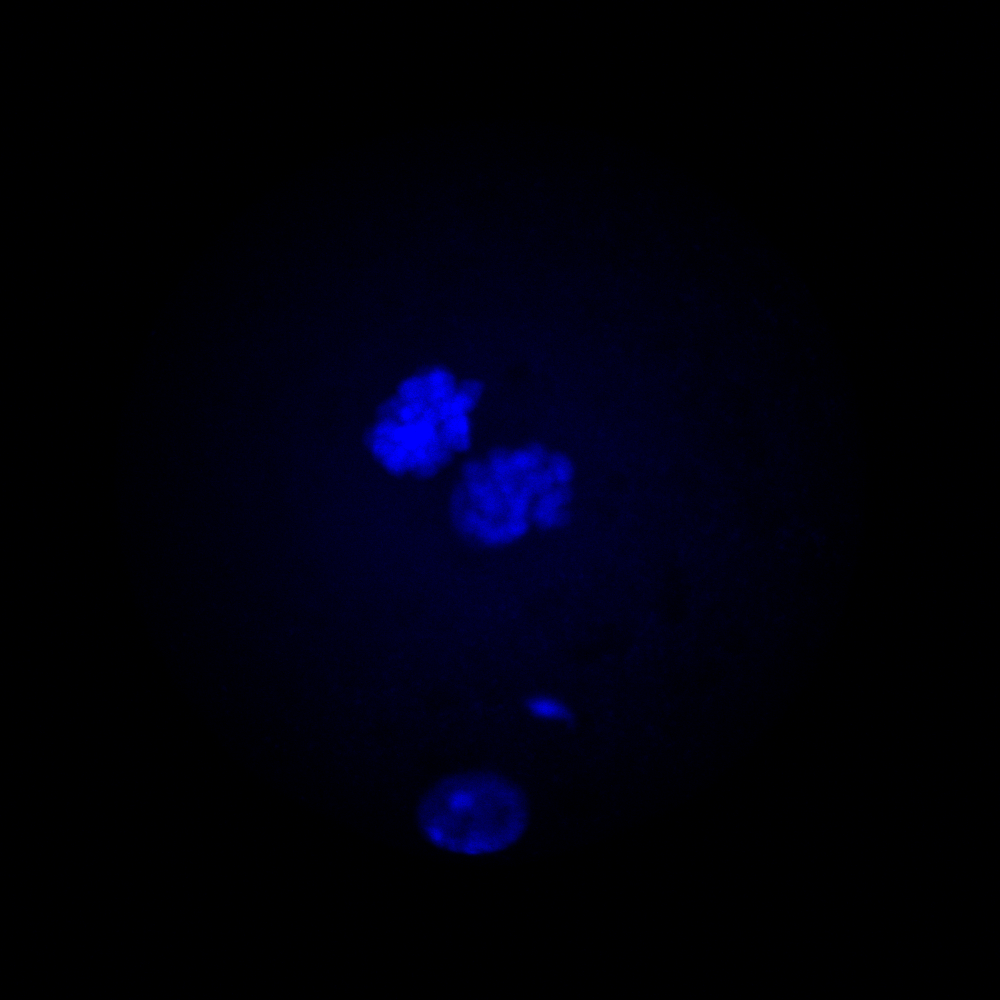

Supplement: Supplementary file 5 — Source data Fig. 3 [file 44319_2024_267_MOESM5_ESM.zip › Figure 3/3D/IF-Noco-DAPI.png]

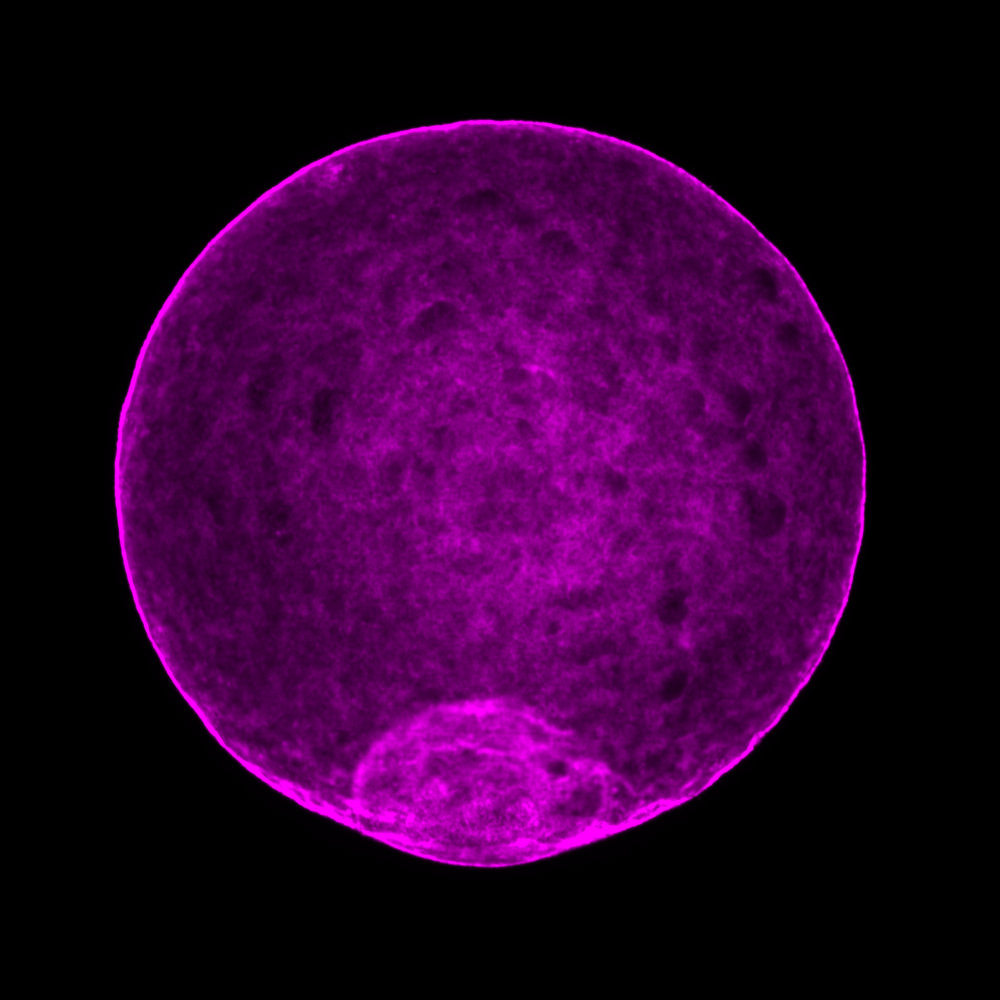

Supplement: Supplementary file 5 — Source data Fig. 3 [file 44319_2024_267_MOESM5_ESM.zip › Figure 3/3D/IF-Noco-Factin.png]

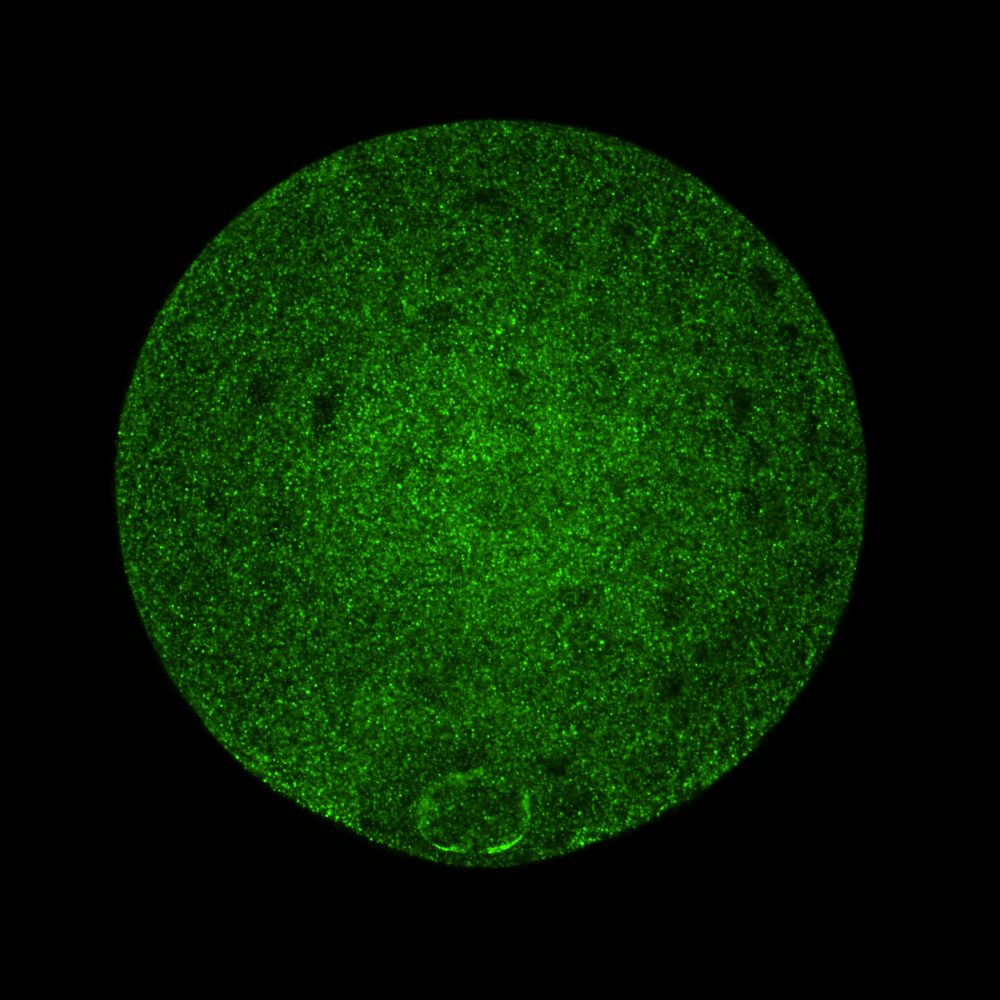

Supplement: Supplementary file 5 — Source data Fig. 3 [file 44319_2024_267_MOESM5_ESM.zip › Figure 3/3D/IF-Noco-LaminB1.png]

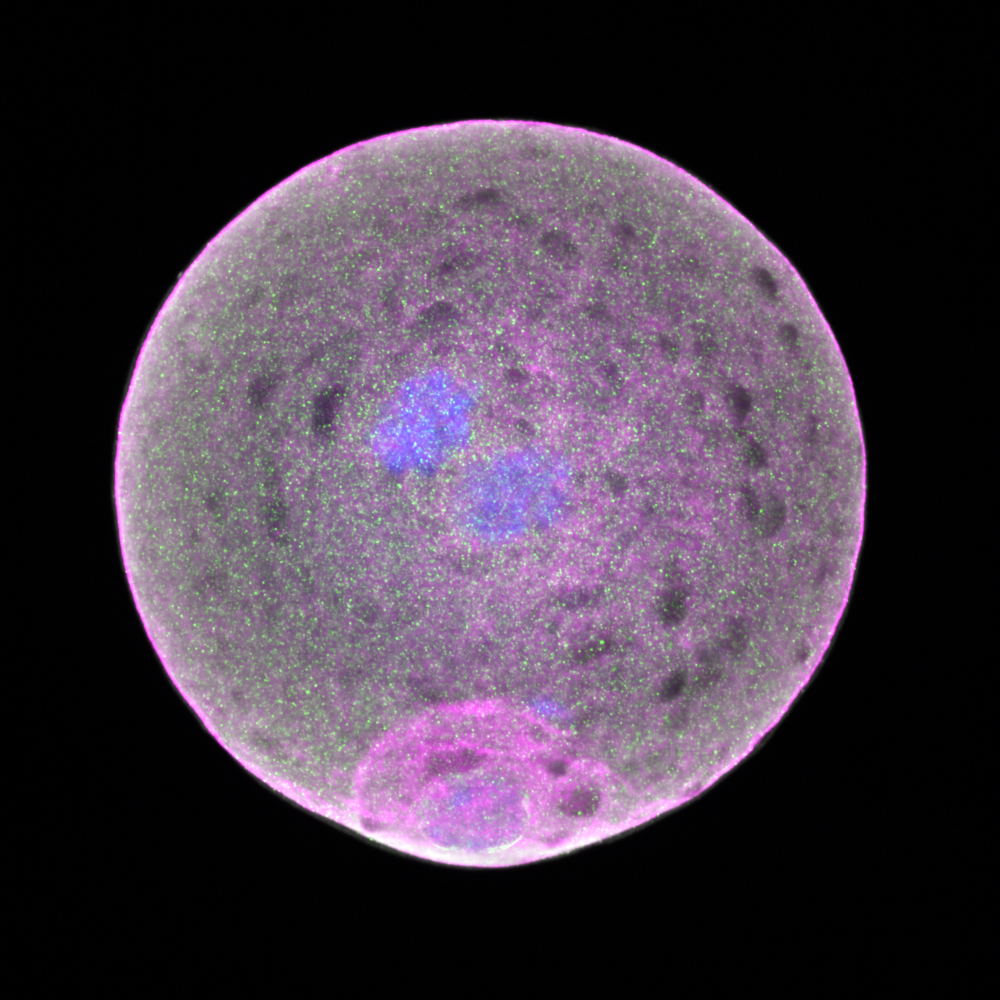

Supplement: Supplementary file 5 — Source data Fig. 3 [file 44319_2024_267_MOESM5_ESM.zip › Figure 3/3D/IF-Noco-Merge.png]

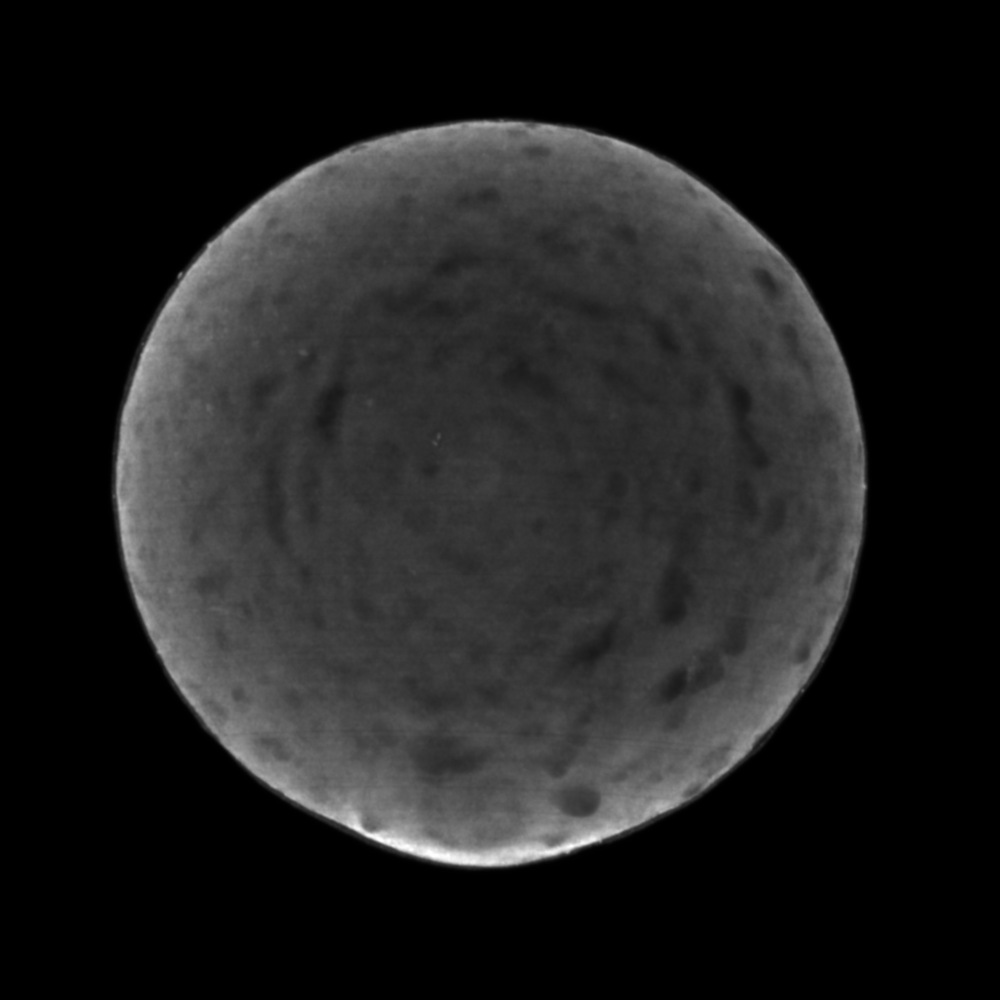

Supplement: Supplementary file 5 — Source data Fig. 3 [file 44319_2024_267_MOESM5_ESM.zip › Figure 3/3D/IF-Noco-αTubulin.png]

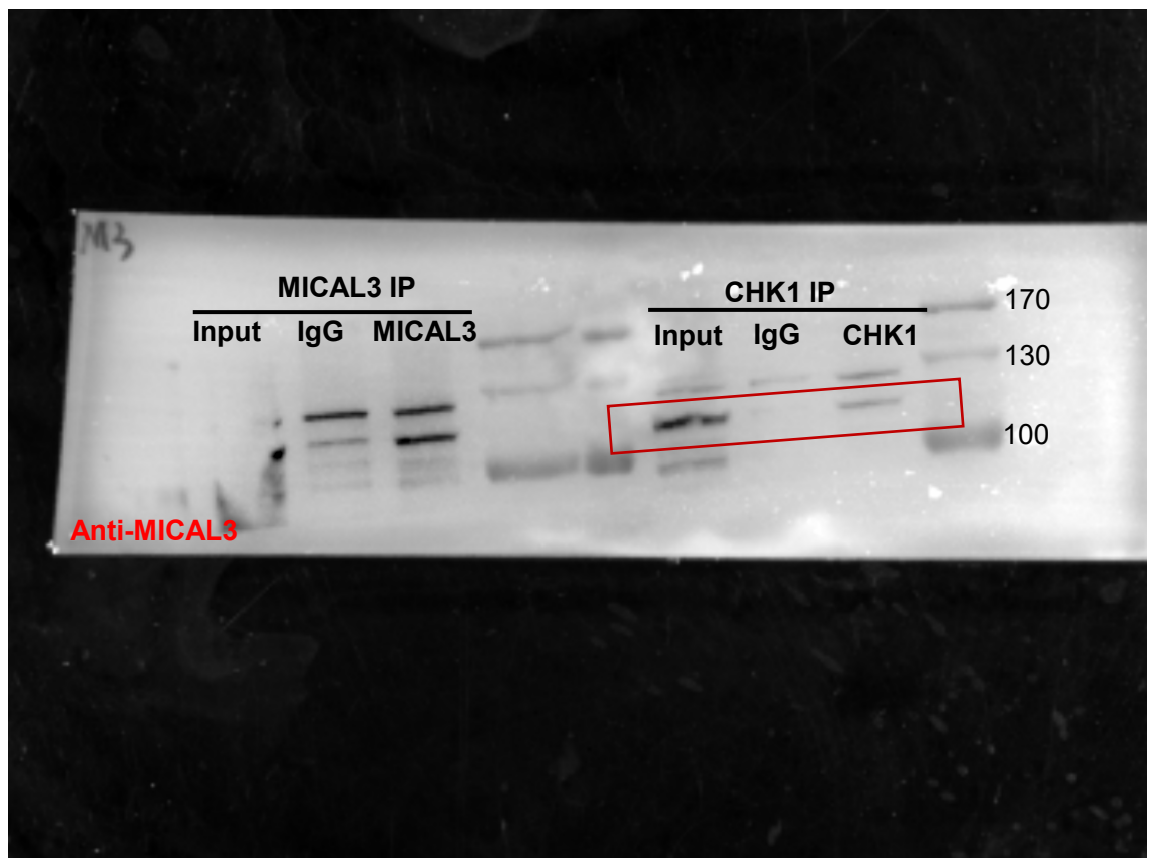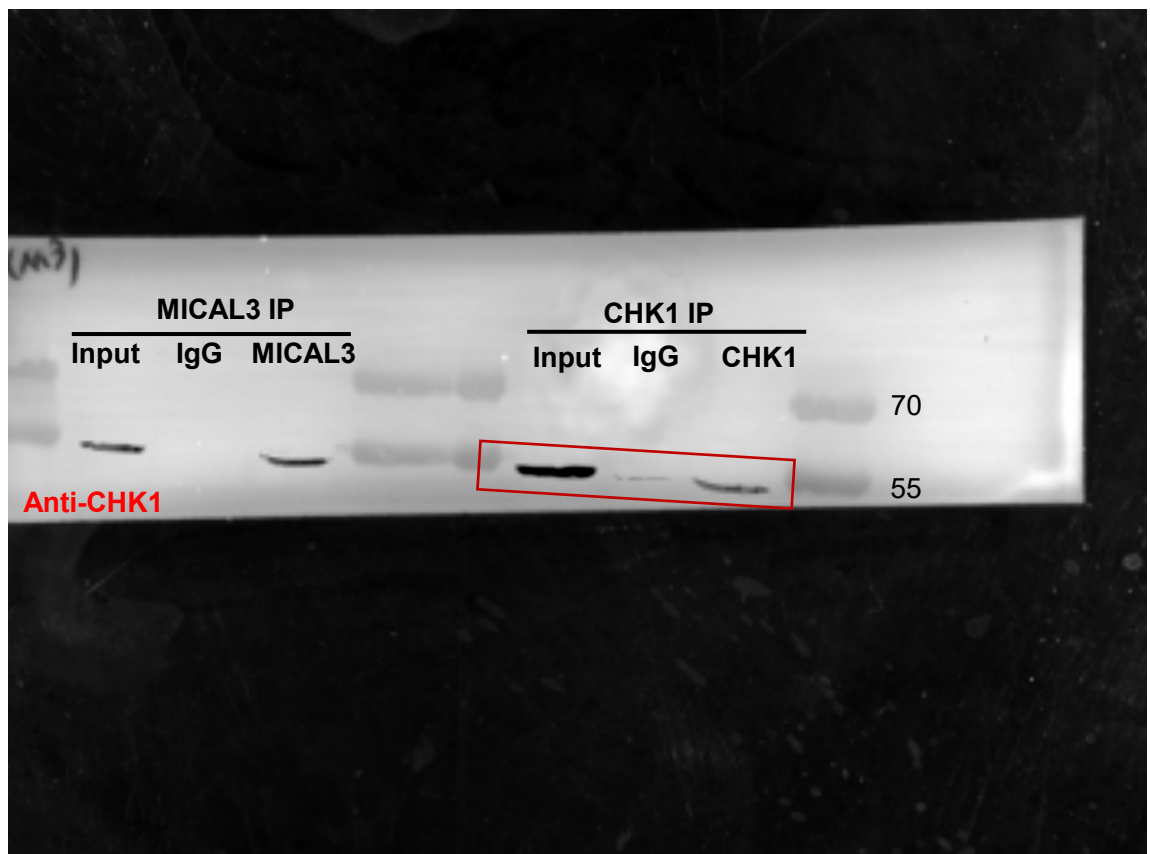

Supplement: Supplementary file 6 — Source data Fig. 4 [file 44319_2024_267_MOESM6_ESM.zip › Figure 4/4B/4B.pdf]

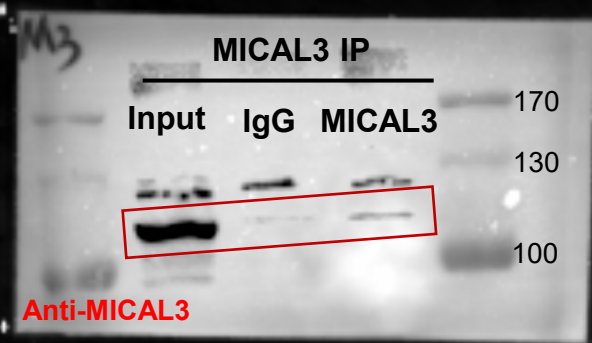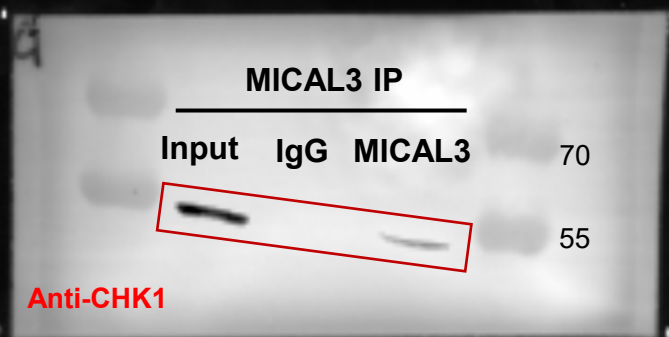

Supplement: Supplementary file 6 — Source data Fig. 4 [file 44319_2024_267_MOESM6_ESM.zip › Figure 4/4C/4C.pdf]

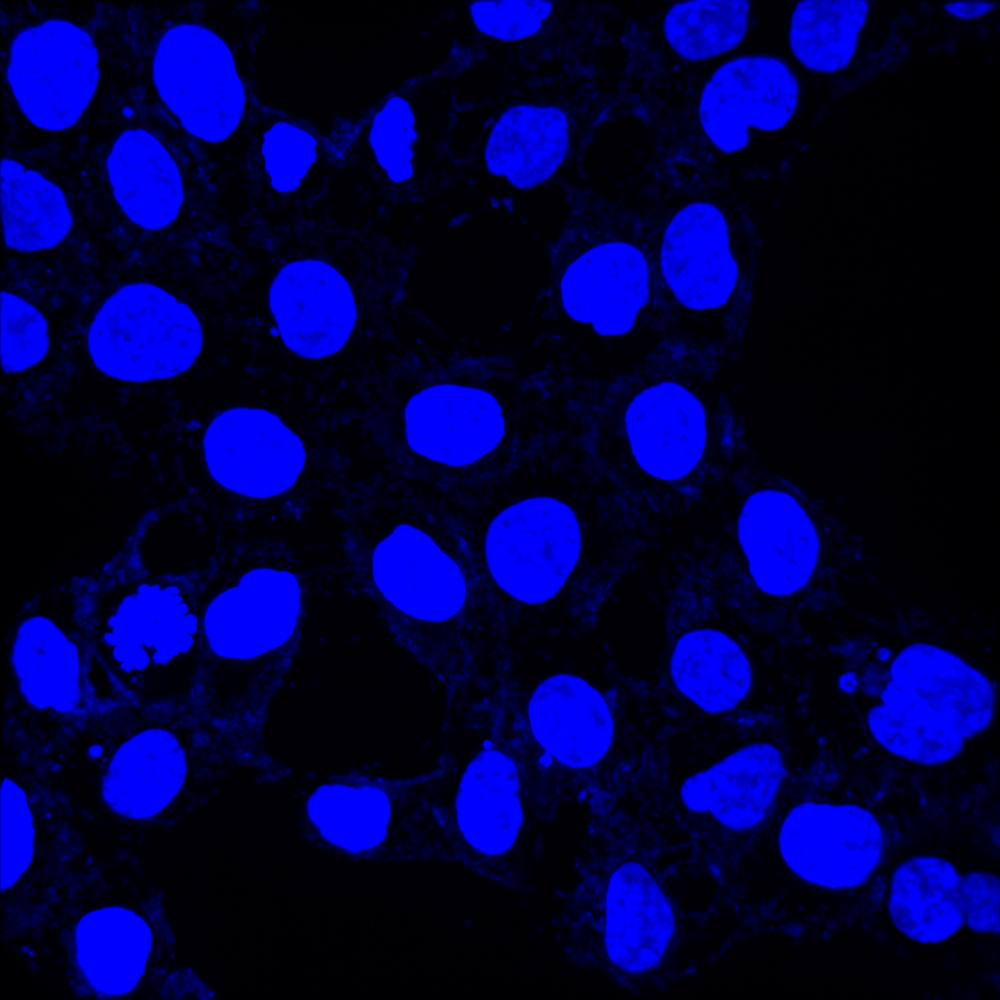

Supplement: Supplementary file 6 — Source data Fig. 4 [file 44319_2024_267_MOESM6_ESM.zip › Figure 4/4D/IF-NC-DAPI.tif]

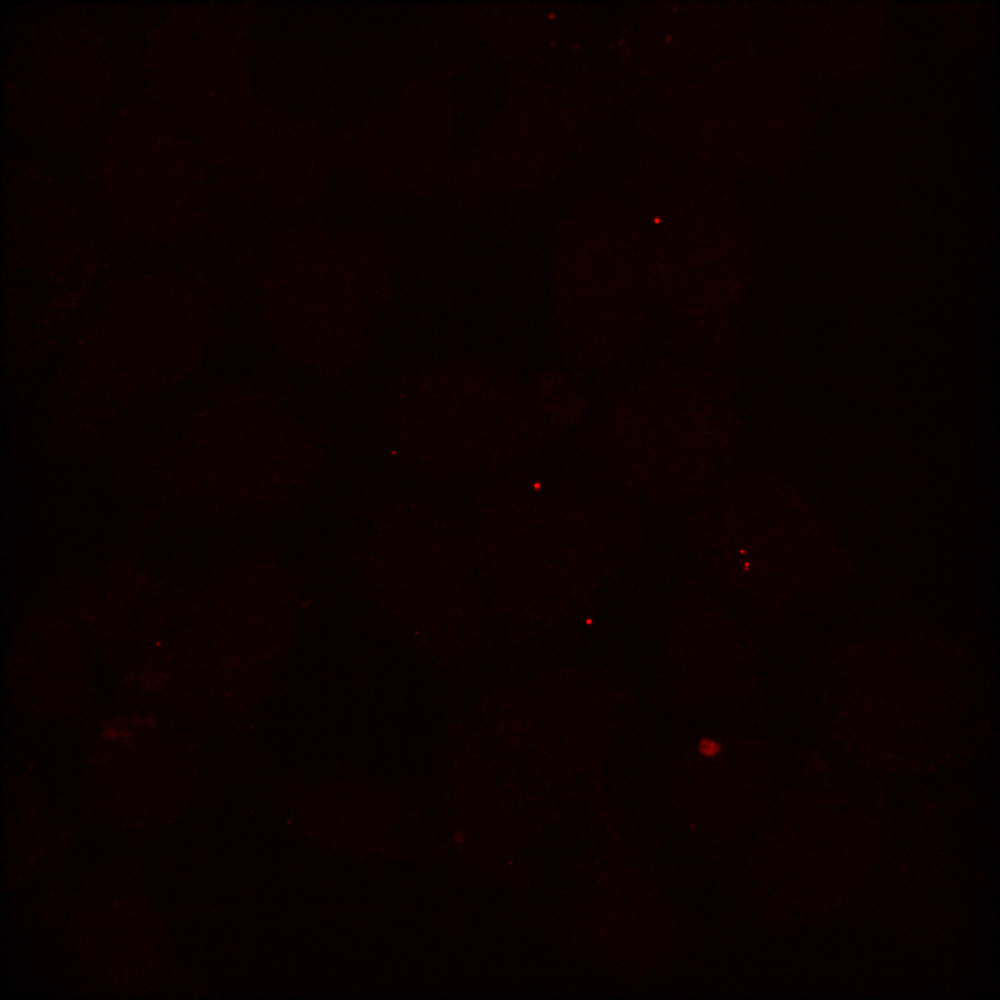

Supplement: Supplementary file 6 — Source data Fig. 4 [file 44319_2024_267_MOESM6_ESM.zip › Figure 4/4D/IF-NC-Red.tif]

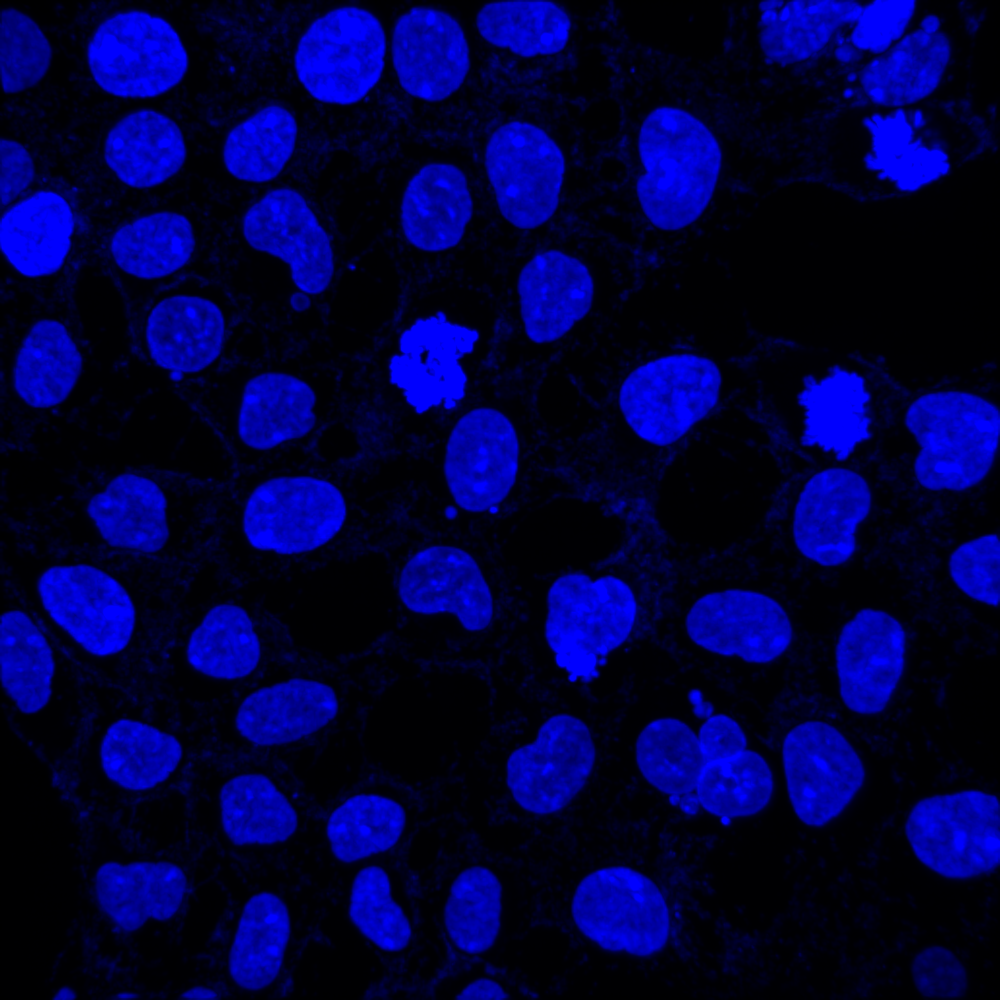

Supplement: Supplementary file 6 — Source data Fig. 4 [file 44319_2024_267_MOESM6_ESM.zip › Figure 4/4D/IF-PLA-DAPI.tif]

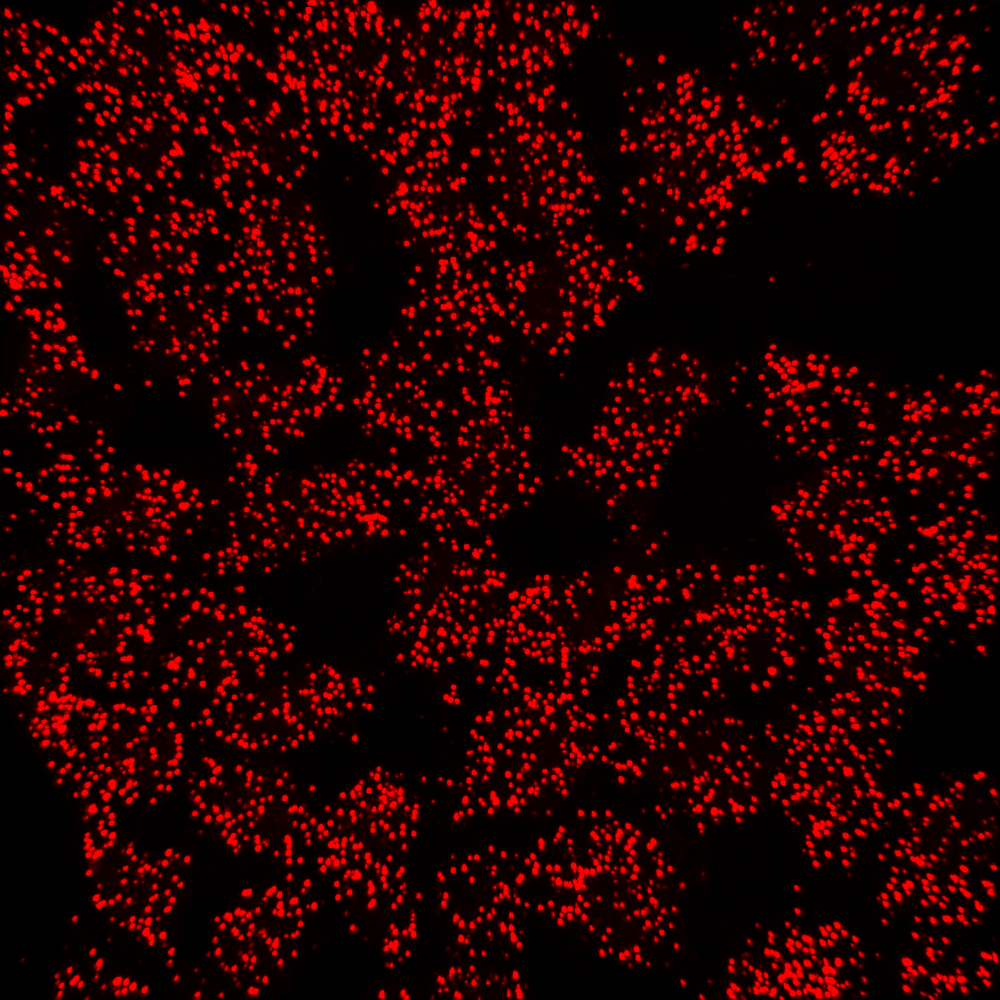

Supplement: Supplementary file 6 — Source data Fig. 4 [file 44319_2024_267_MOESM6_ESM.zip › Figure 4/4D/IF-PLA-Red.tif]

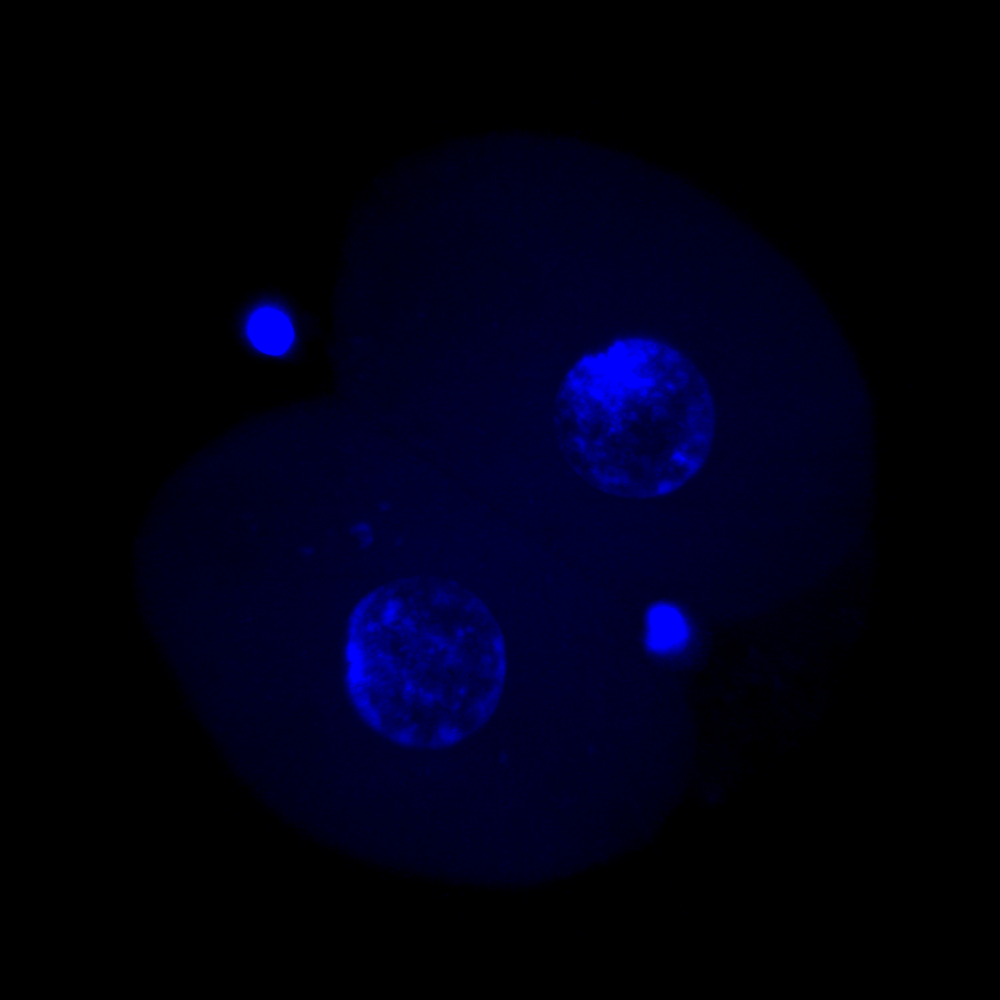

Supplement: Supplementary file 6 — Source data Fig. 4 [file 44319_2024_267_MOESM6_ESM.zip › Figure 4/4F/IF-2cell-DAPI.tif]

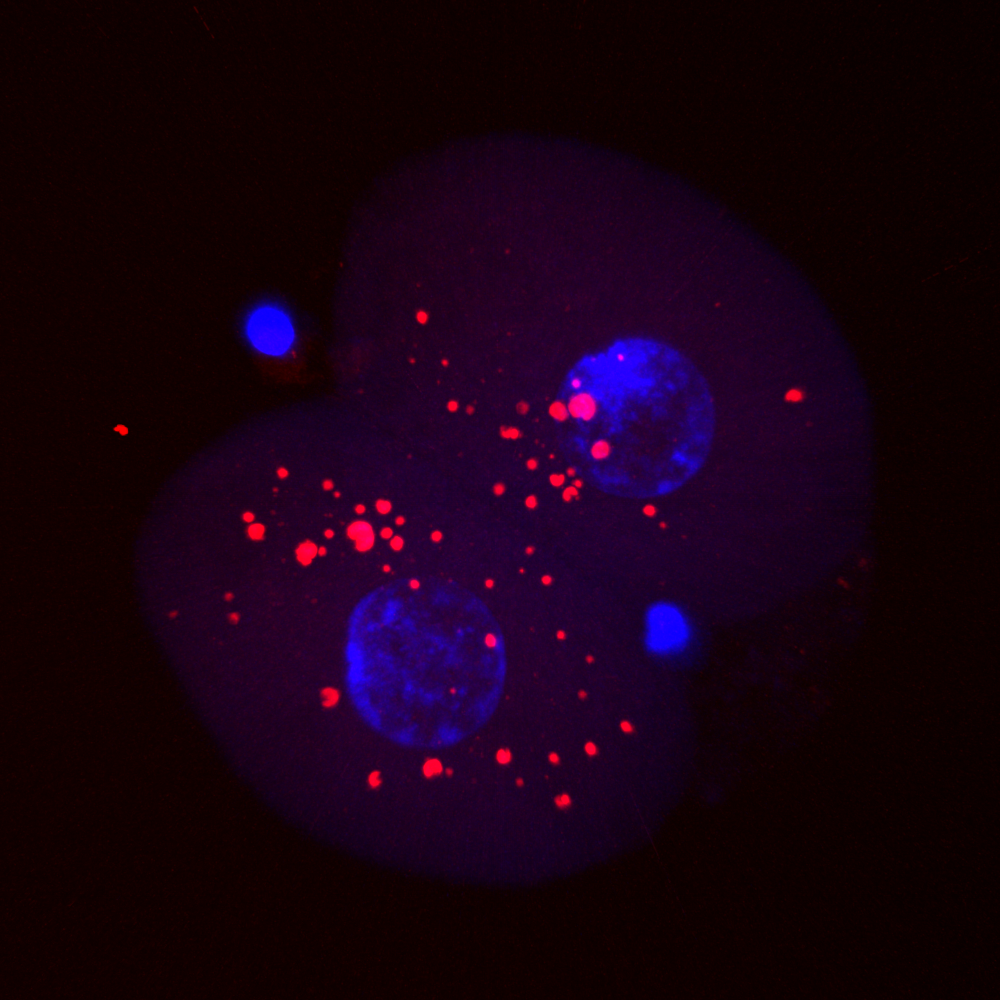

Supplement: Supplementary file 6 — Source data Fig. 4 [file 44319_2024_267_MOESM6_ESM.zip › Figure 4/4F/IF-2cell-Merge.tif]

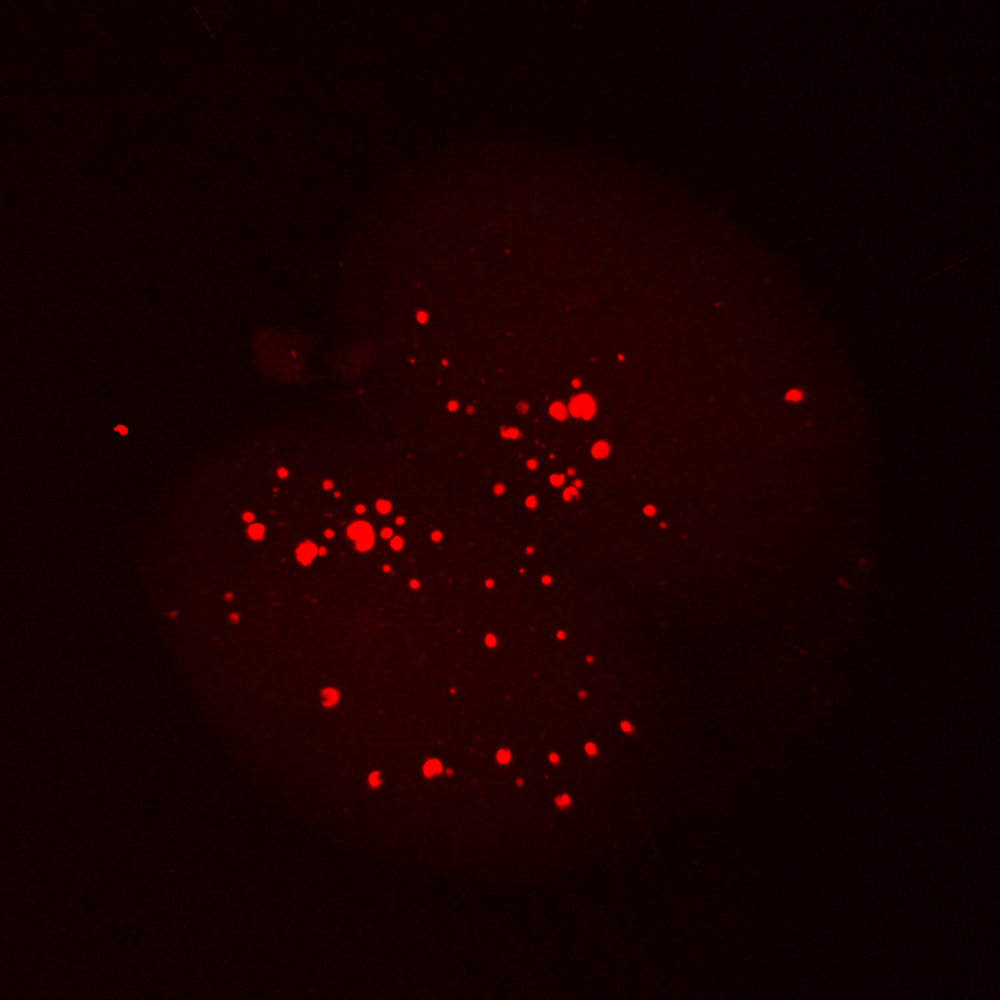

Supplement: Supplementary file 6 — Source data Fig. 4 [file 44319_2024_267_MOESM6_ESM.zip › Figure 4/4F/IF-2cell-PLA.tif]

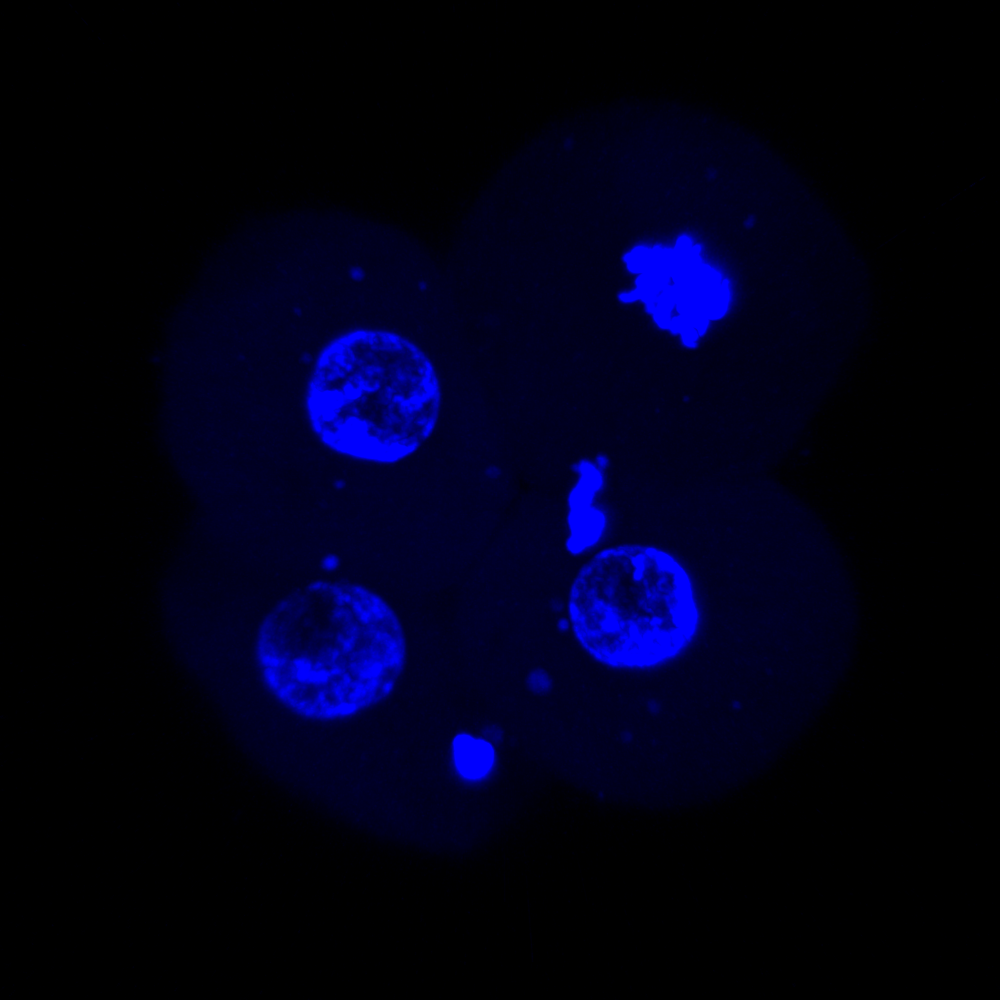

Supplement: Supplementary file 6 — Source data Fig. 4 [file 44319_2024_267_MOESM6_ESM.zip › Figure 4/4F/IF-4cell-DAPI.tif]

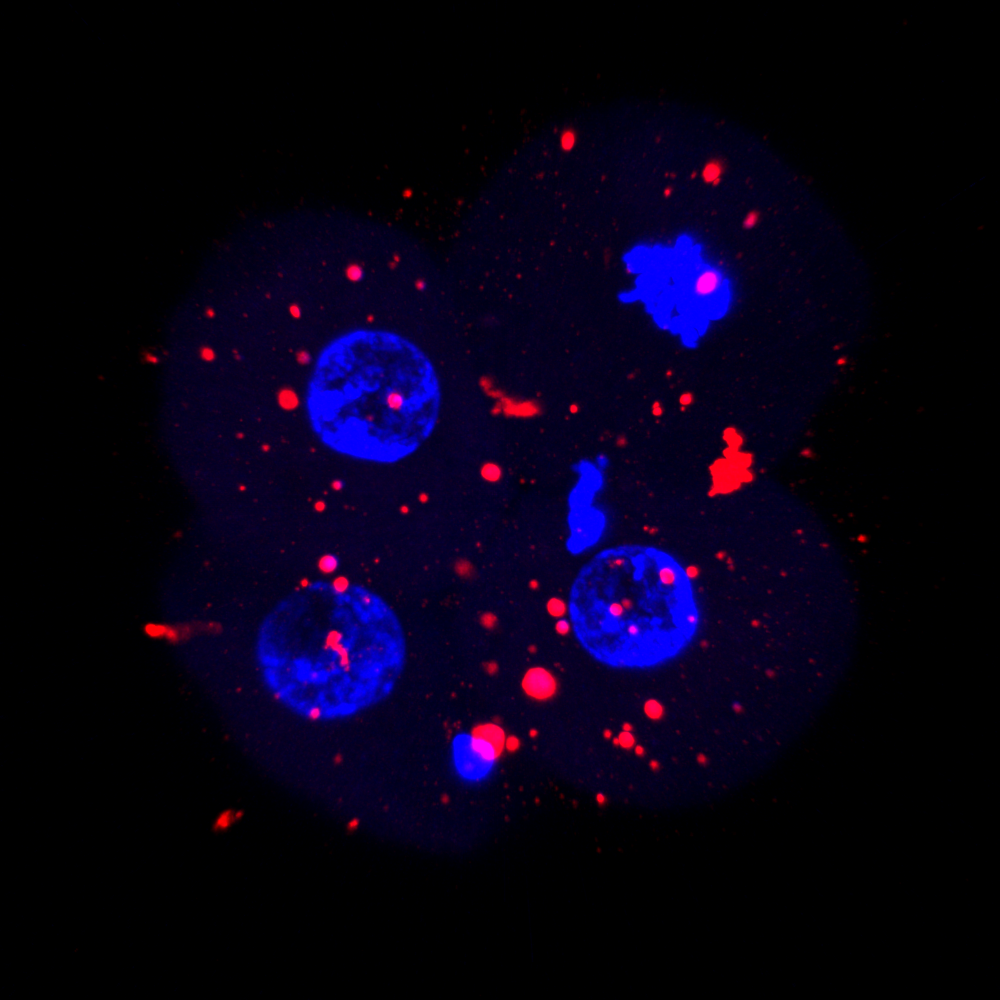

Supplement: Supplementary file 6 — Source data Fig. 4 [file 44319_2024_267_MOESM6_ESM.zip › Figure 4/4F/IF-4cell-Merge.tif]

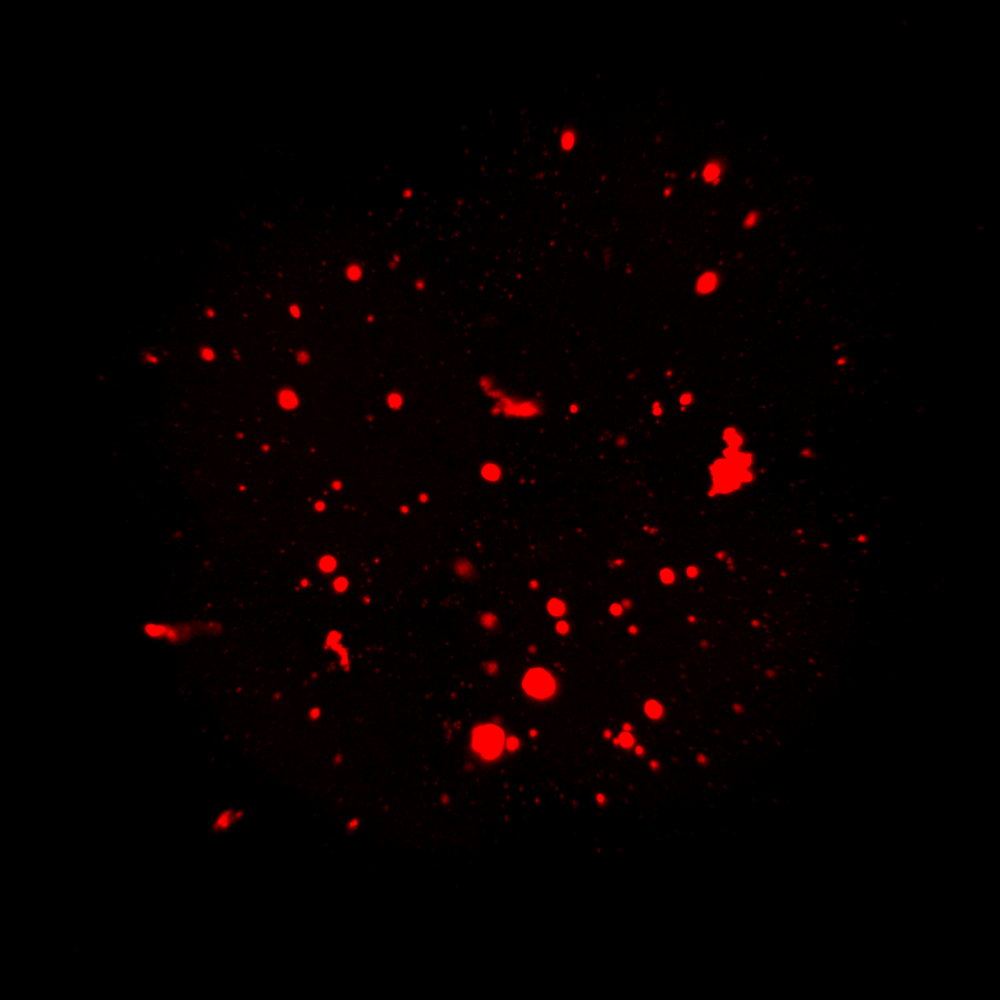

Supplement: Supplementary file 6 — Source data Fig. 4 [file 44319_2024_267_MOESM6_ESM.zip › Figure 4/4F/IF-4cell-PLA.tif]

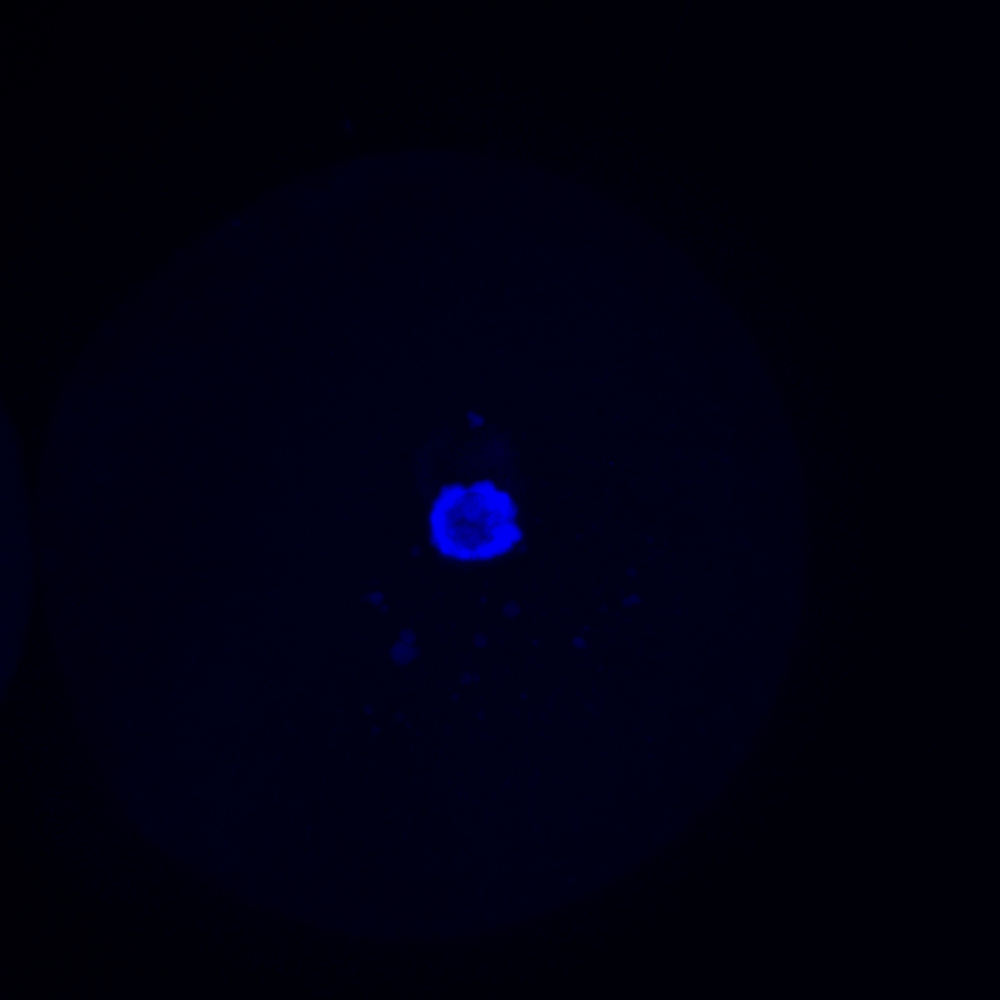

Supplement: Supplementary file 6 — Source data Fig. 4 [file 44319_2024_267_MOESM6_ESM.zip › Figure 4/4F/IF-GV-DAPI.tif]

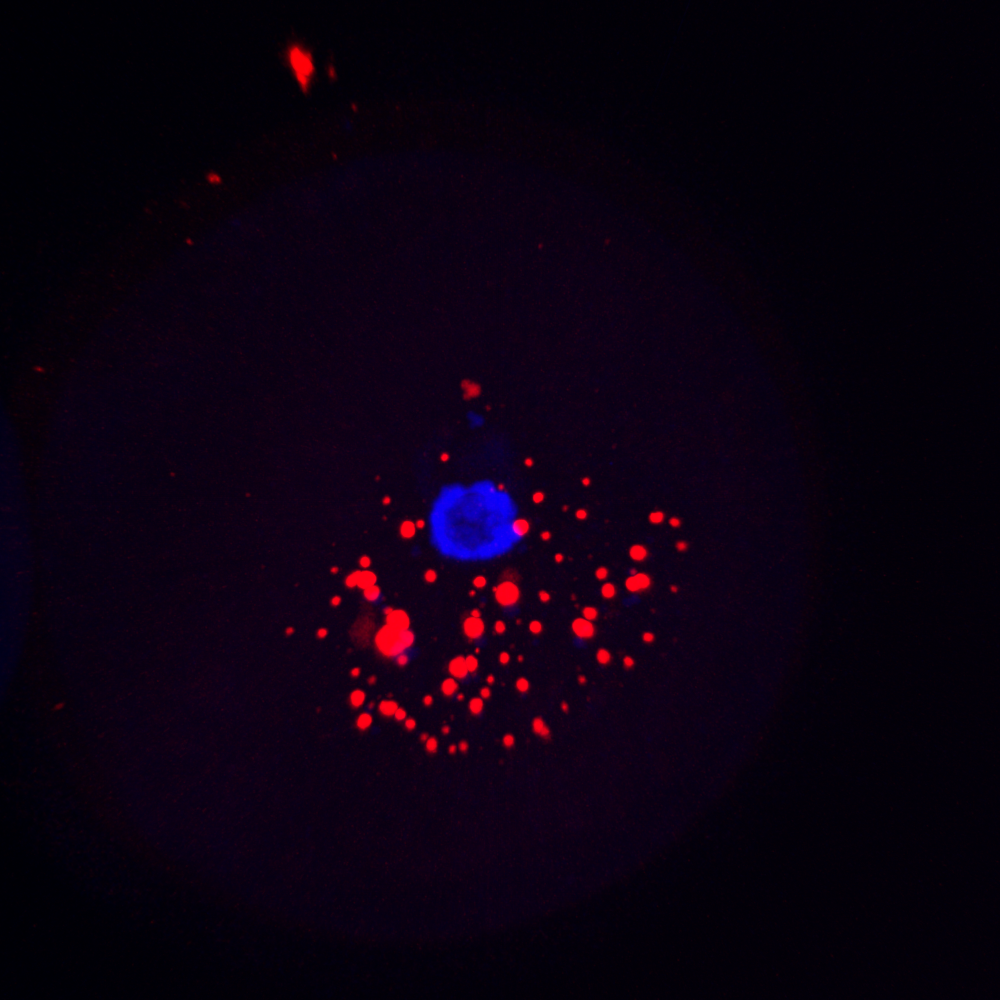

Supplement: Supplementary file 6 — Source data Fig. 4 [file 44319_2024_267_MOESM6_ESM.zip › Figure 4/4F/IF-GV-Merge.tif]

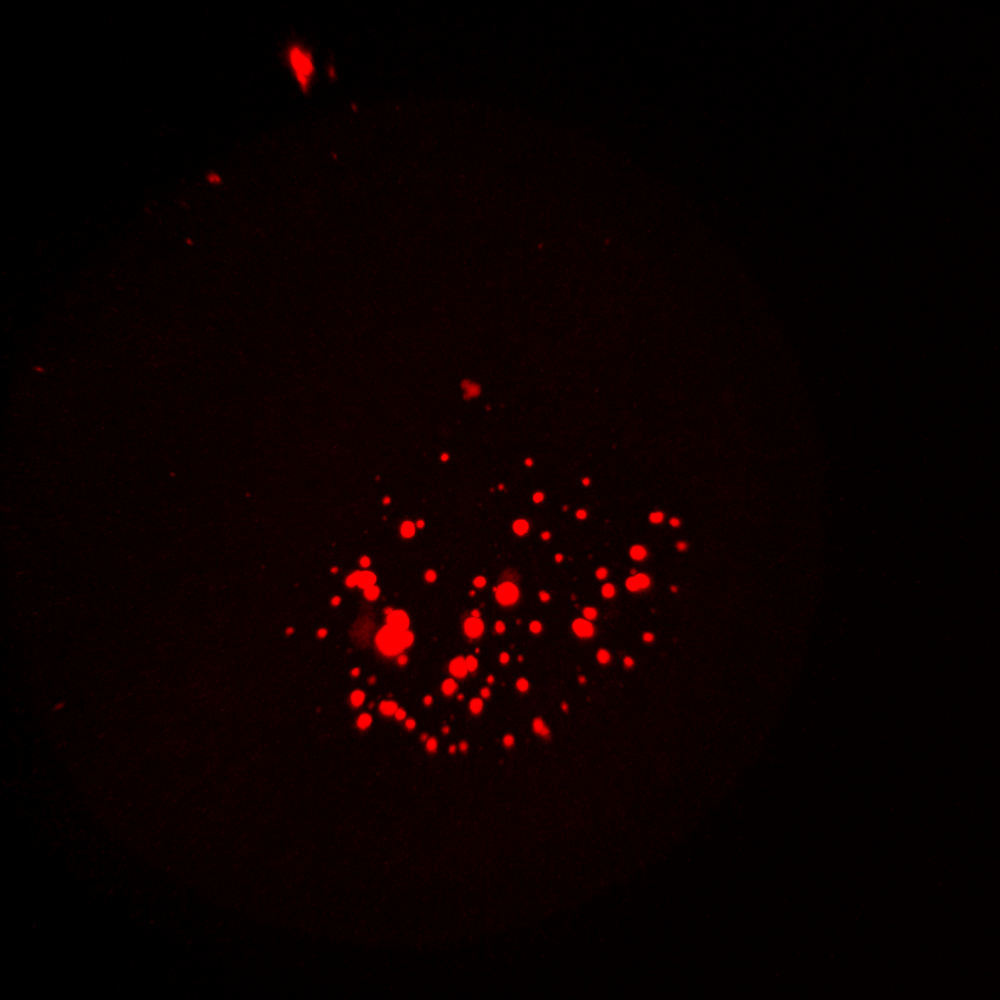

Supplement: Supplementary file 6 — Source data Fig. 4 [file 44319_2024_267_MOESM6_ESM.zip › Figure 4/4F/IF-GV-PLA.tif]

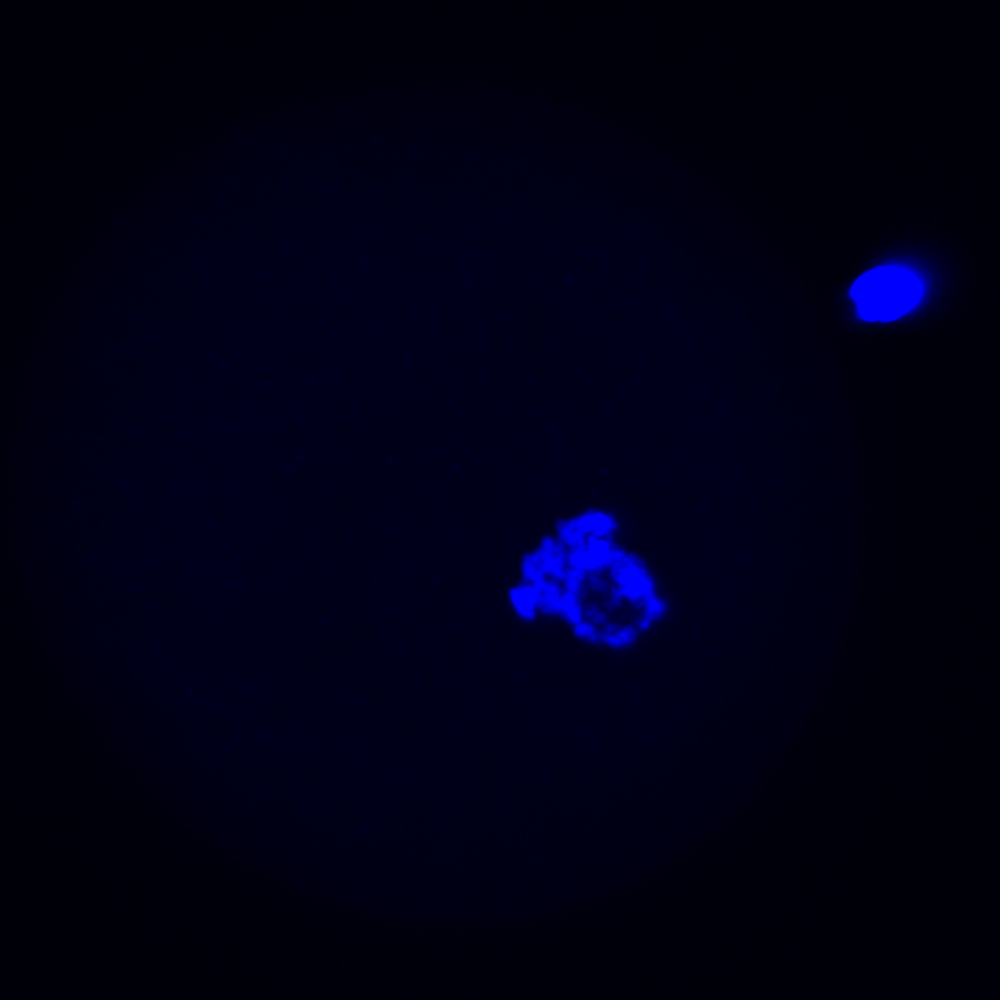

Supplement: Supplementary file 6 — Source data Fig. 4 [file 44319_2024_267_MOESM6_ESM.zip › Figure 4/4G/IF-GV-DAPI.tif]

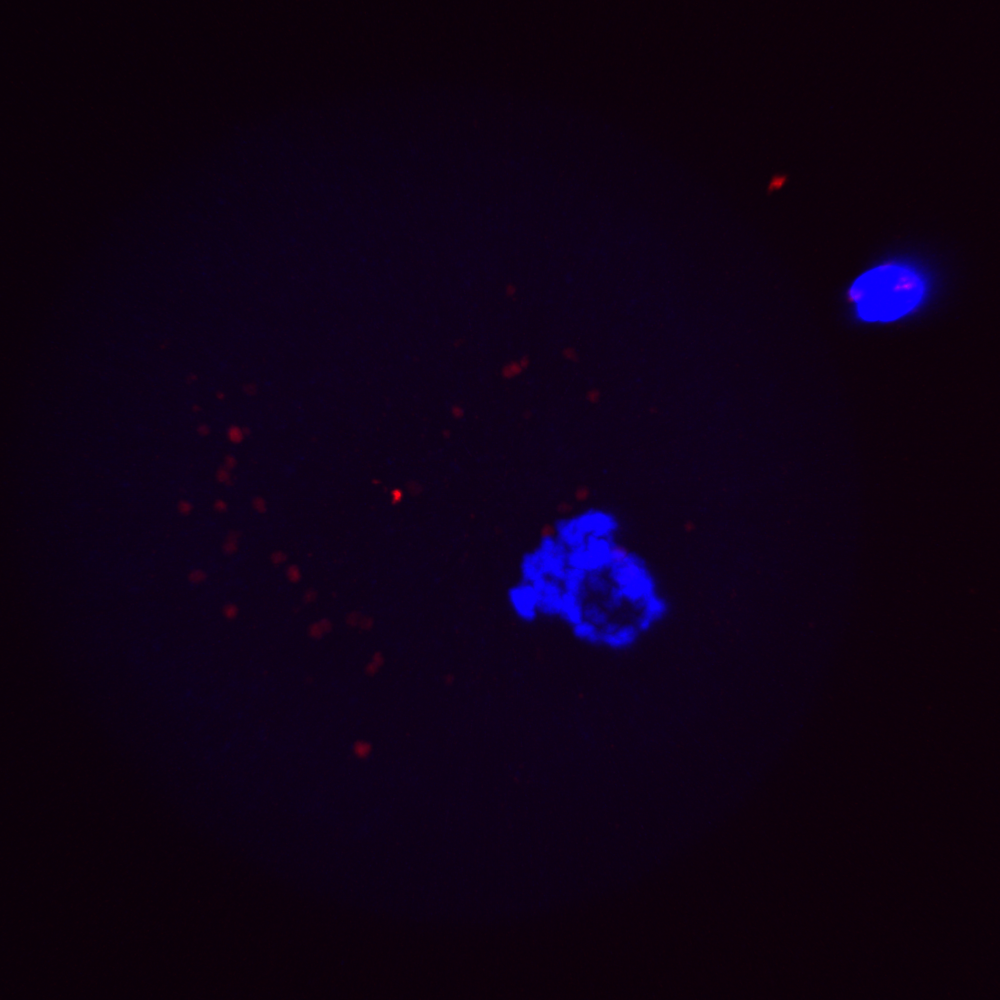

Supplement: Supplementary file 6 — Source data Fig. 4 [file 44319_2024_267_MOESM6_ESM.zip › Figure 4/4G/IF-GV-Merge.tif]

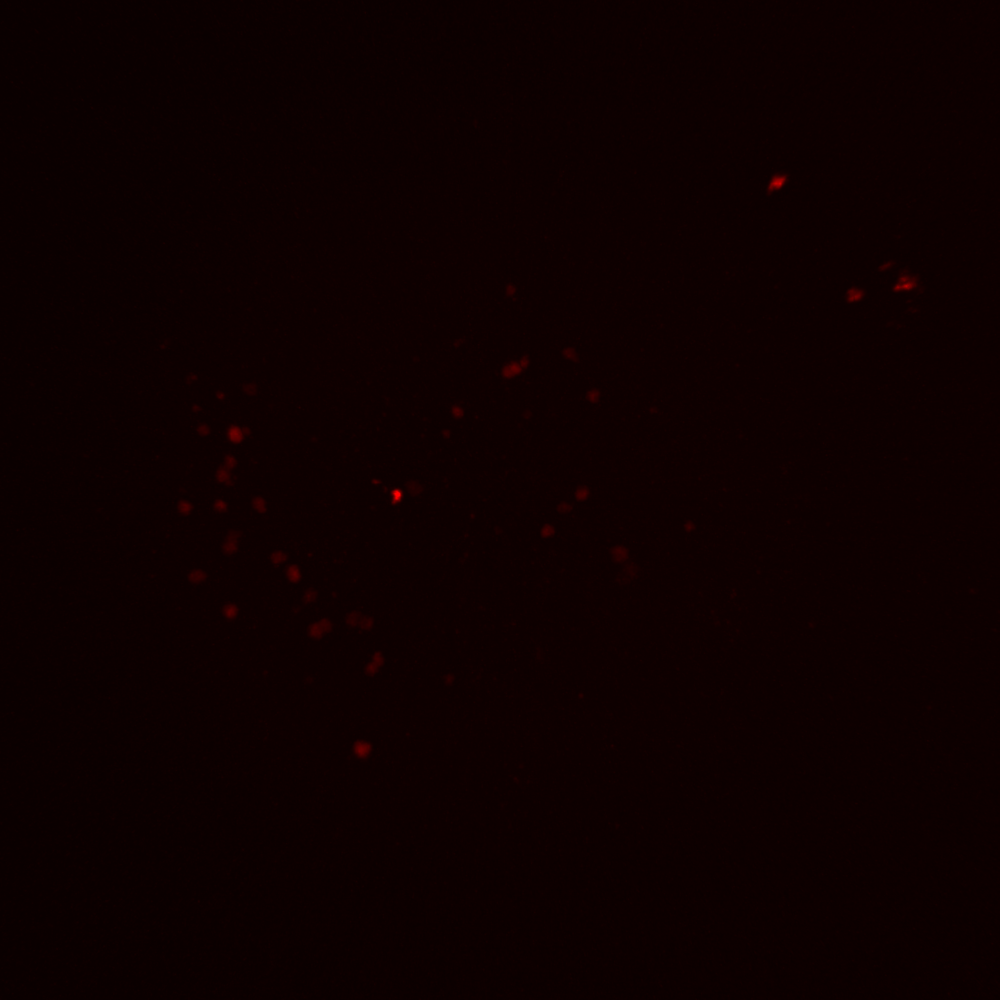

Supplement: Supplementary file 6 — Source data Fig. 4 [file 44319_2024_267_MOESM6_ESM.zip › Figure 4/4G/IF-GV-NC.tif]

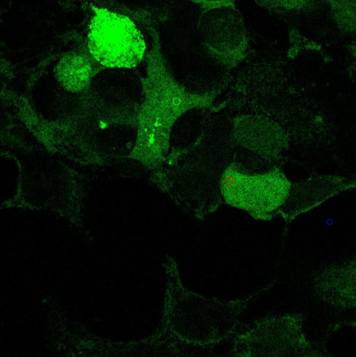

Supplement: Supplementary file 7 — Source data Fig. 5 [file 44319_2024_267_MOESM7_ESM.zip › Figure 5/5B/1-266-EGFP-after bleaching.png]

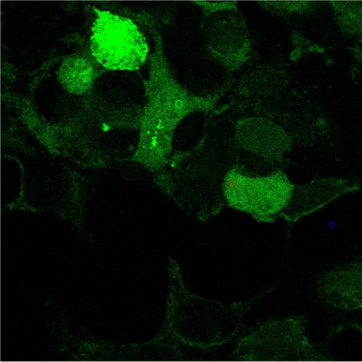

Supplement: Supplementary file 7 — Source data Fig. 5 [file 44319_2024_267_MOESM7_ESM.zip › Figure 5/5B/1-266-EGFP-before bleaching.png]

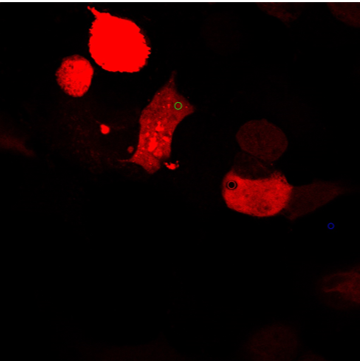

Supplement: Supplementary file 7 — Source data Fig. 5 [file 44319_2024_267_MOESM7_ESM.zip › Figure 5/5B/1-266-mCherry-after bleaching.png]

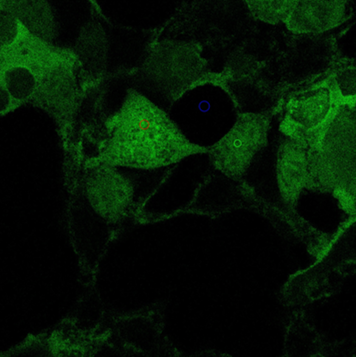

Supplement: Supplementary file 7 — Source data Fig. 5 [file 44319_2024_267_MOESM7_ESM.zip › Figure 5/5B/F441fs16-EGFP-after bleaching.png]

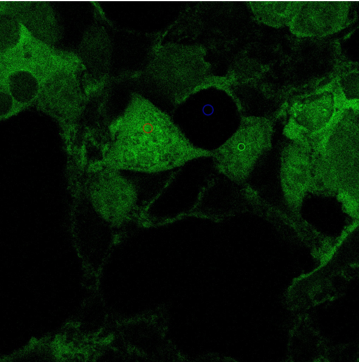

Supplement: Supplementary file 7 — Source data Fig. 5 [file 44319_2024_267_MOESM7_ESM.zip › Figure 5/5B/F441fs16-EGFP-before bleaching.jpg.png]

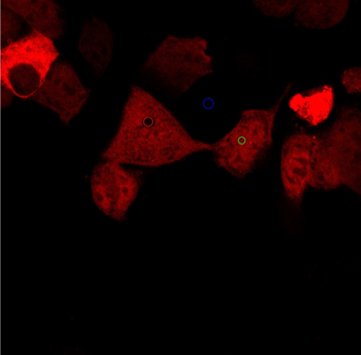

Supplement: Supplementary file 7 — Source data Fig. 5 [file 44319_2024_267_MOESM7_ESM.zip › Figure 5/5B/F441fs16-mCherry-after bleaching.png]
