# Supplementary figures and images for: CHK1 controls zygote pronuclear envelope breakdown by regulating F-actin through interacting with MICAL3 (part 2 of 2)
Source: EMBO Rep. 2024 Oct 2;25(11):4876–97. doi: 10.1038/s44319-024-00267-7 (PMC11549291; doi:10.1038/s44319-024-00267-7)

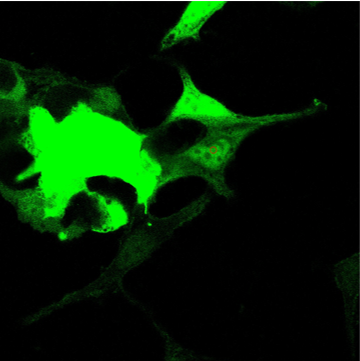

Supplement: Supplementary file 7 — Source data Fig. 5 [file 44319_2024_267_MOESM7_ESM.zip › Figure 5/5B/R379Q-EGFP-after bleaching.png]

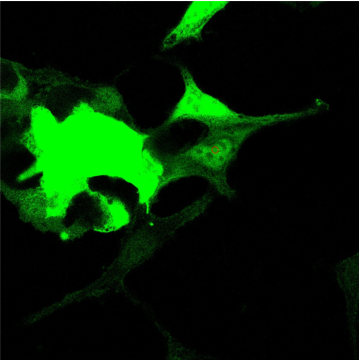

Supplement: Supplementary file 7 — Source data Fig. 5 [file 44319_2024_267_MOESM7_ESM.zip › Figure 5/5B/R379Q-EGFP-before bleaching.png]

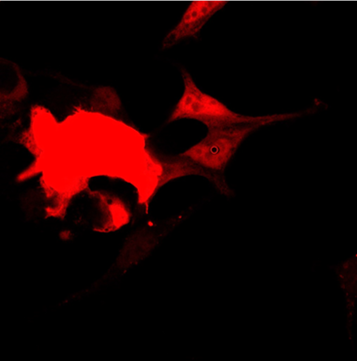

Supplement: Supplementary file 7 — Source data Fig. 5 [file 44319_2024_267_MOESM7_ESM.zip › Figure 5/5B/R379Q-mCherry-after bleaching.png]

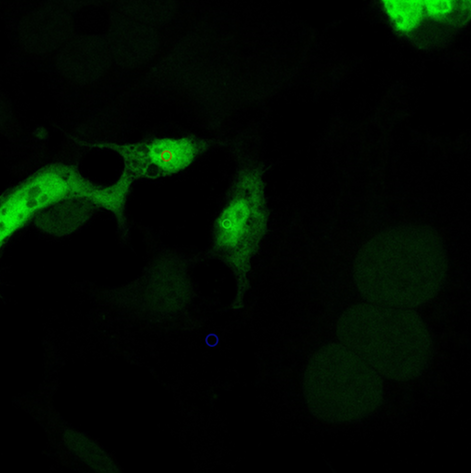

Supplement: Supplementary file 7 — Source data Fig. 5 [file 44319_2024_267_MOESM7_ESM.zip › Figure 5/5B/WT-EGFP-after bleaching.png]

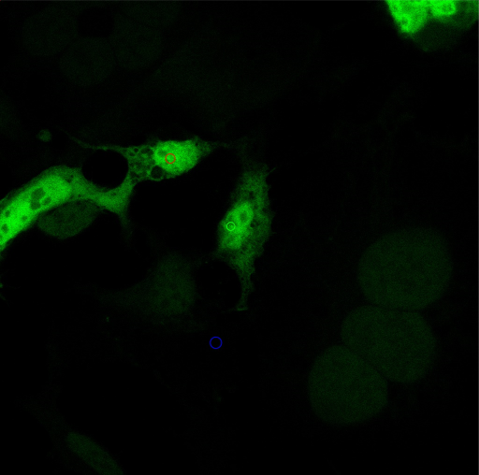

Supplement: Supplementary file 7 — Source data Fig. 5 [file 44319_2024_267_MOESM7_ESM.zip › Figure 5/5B/WT-EGFP-before bleaching.png]

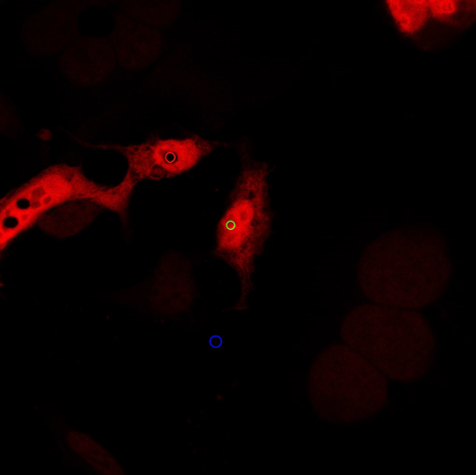

Supplement: Supplementary file 7 — Source data Fig. 5 [file 44319_2024_267_MOESM7_ESM.zip › Figure 5/5B/WT-mCherry-after bleaching.png]

IgG WT 1-266 F441fs\*16 R379Q 1-266 F441fs\*16

PF477736

IP

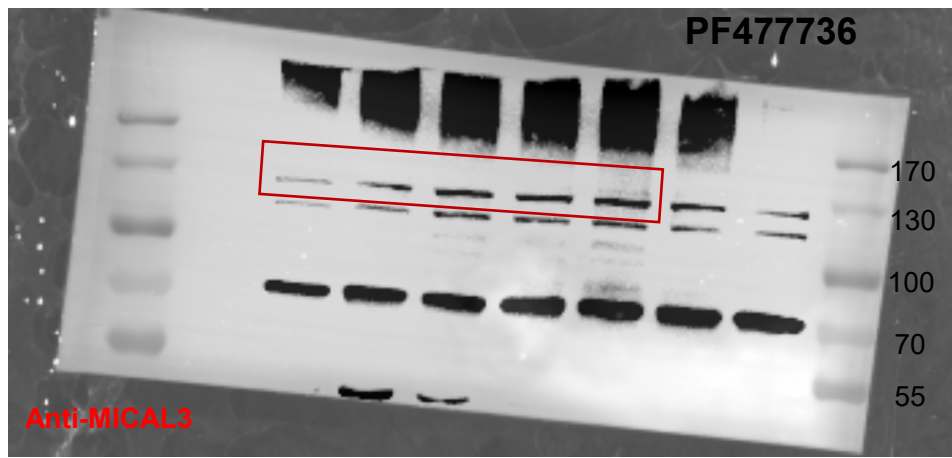

Anti-MICAL3

Input

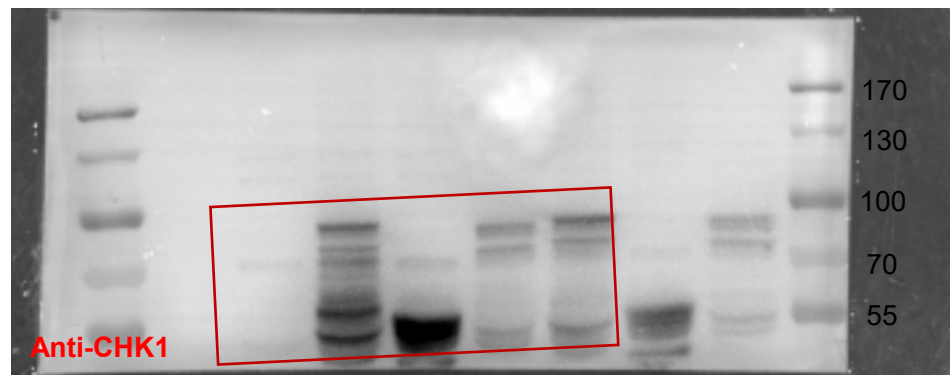

Anti-CHK1

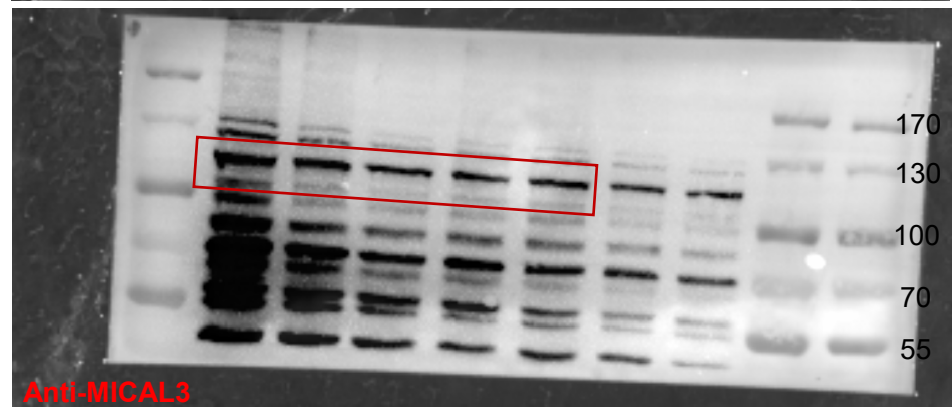

Anti-MICAL3

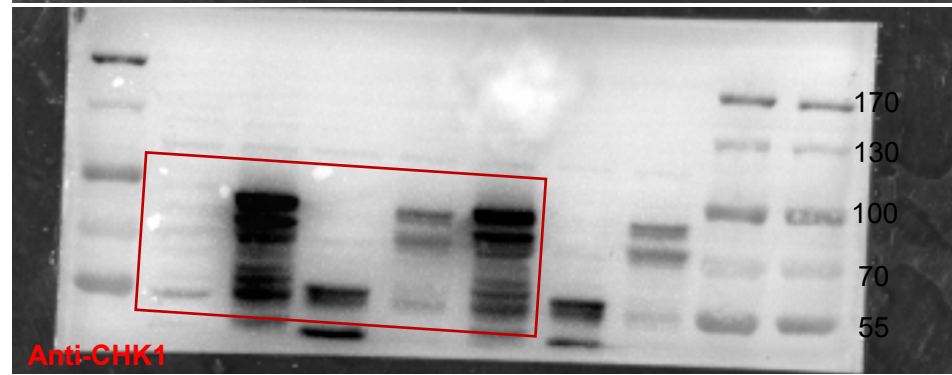

Anti-CHK1

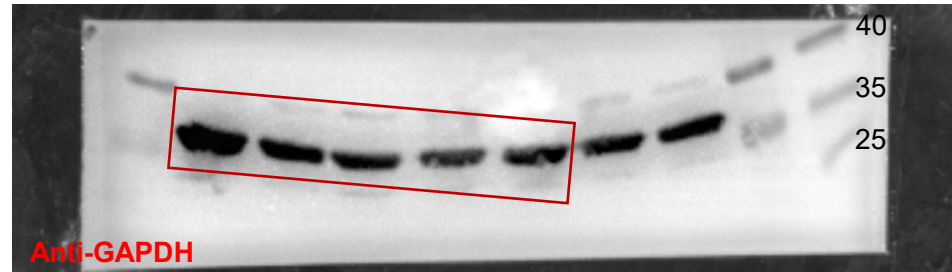

Anti-GAPDH

Supplement: Supplementary file 7 — Source data Fig. 5 [file 44319_2024_267_MOESM7_ESM.zip › Figure 5/5D/5D.pdf]

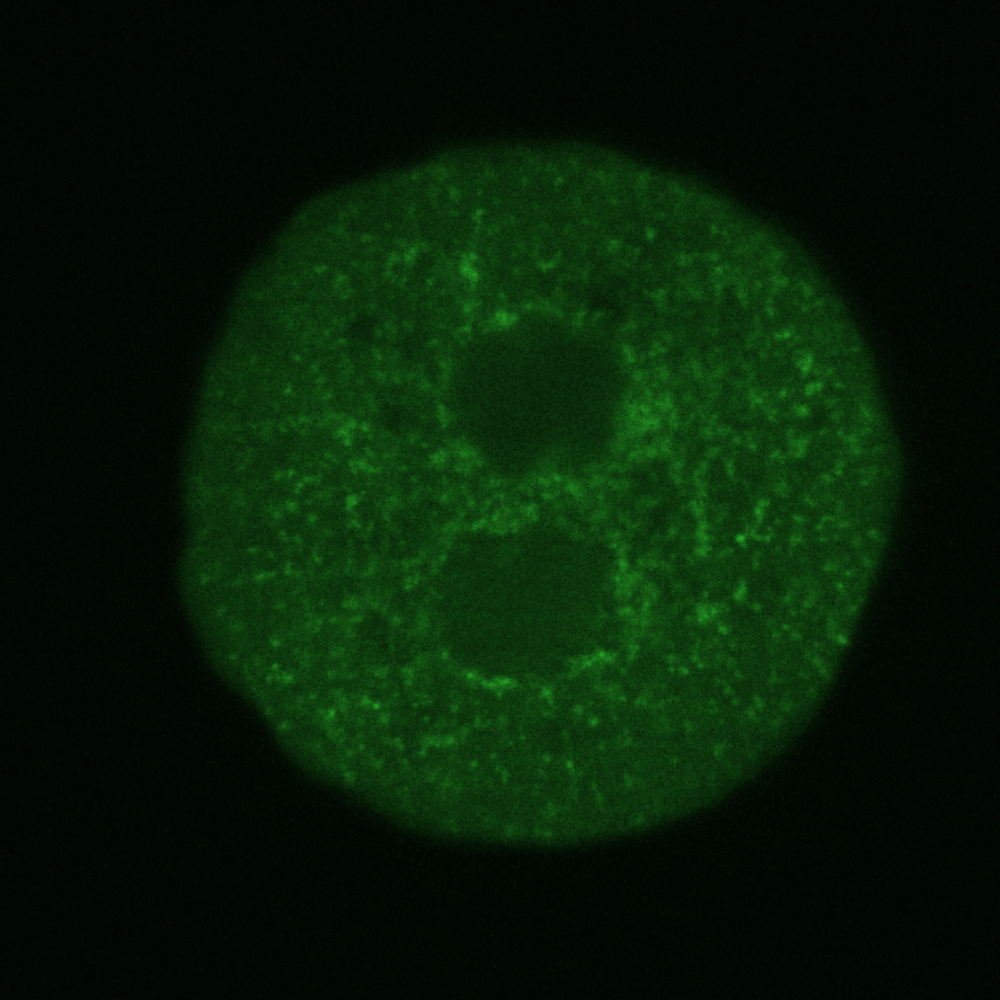

Supplement: Supplementary file 7 — Source data Fig. 5 [file 44319_2024_267_MOESM7_ESM.zip › Figure 5/5G/H2O2-F441-Left.tif]

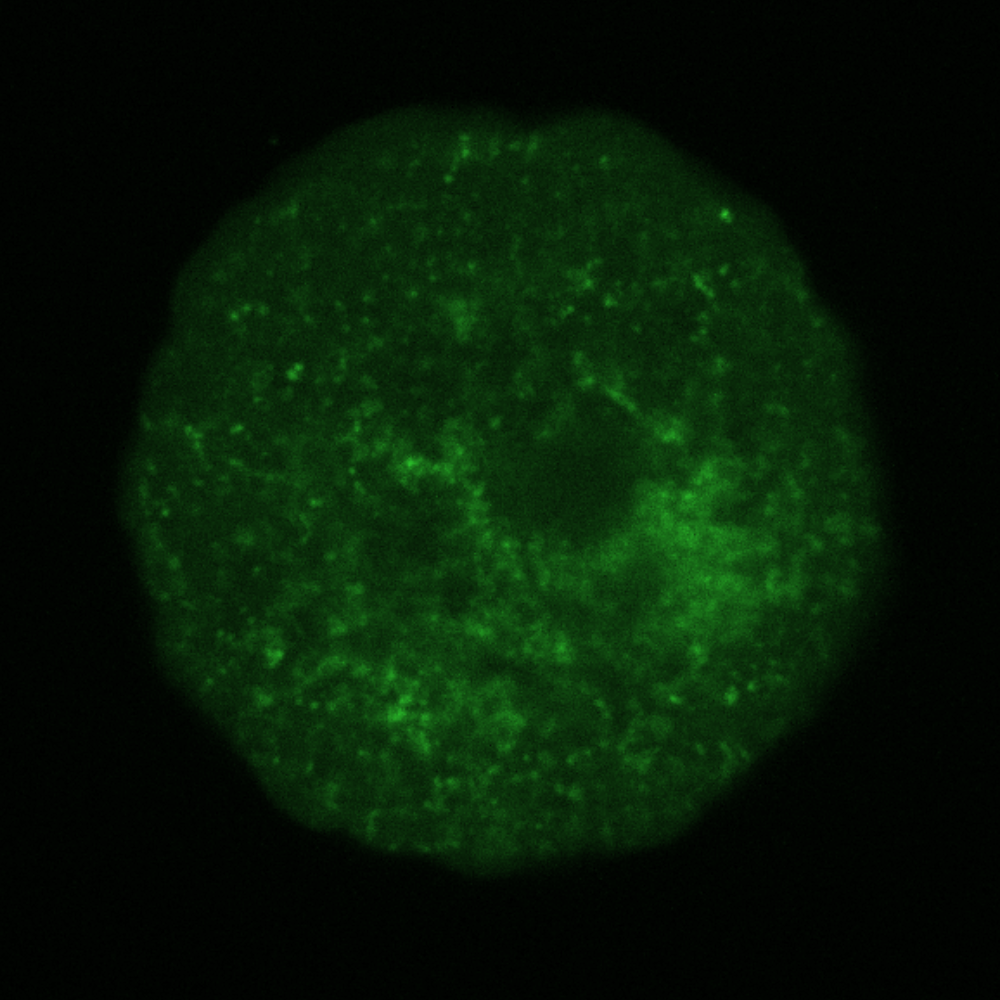

Supplement: Supplementary file 7 — Source data Fig. 5 [file 44319_2024_267_MOESM7_ESM.zip › Figure 5/5G/H2O2-F441-Right.tif]

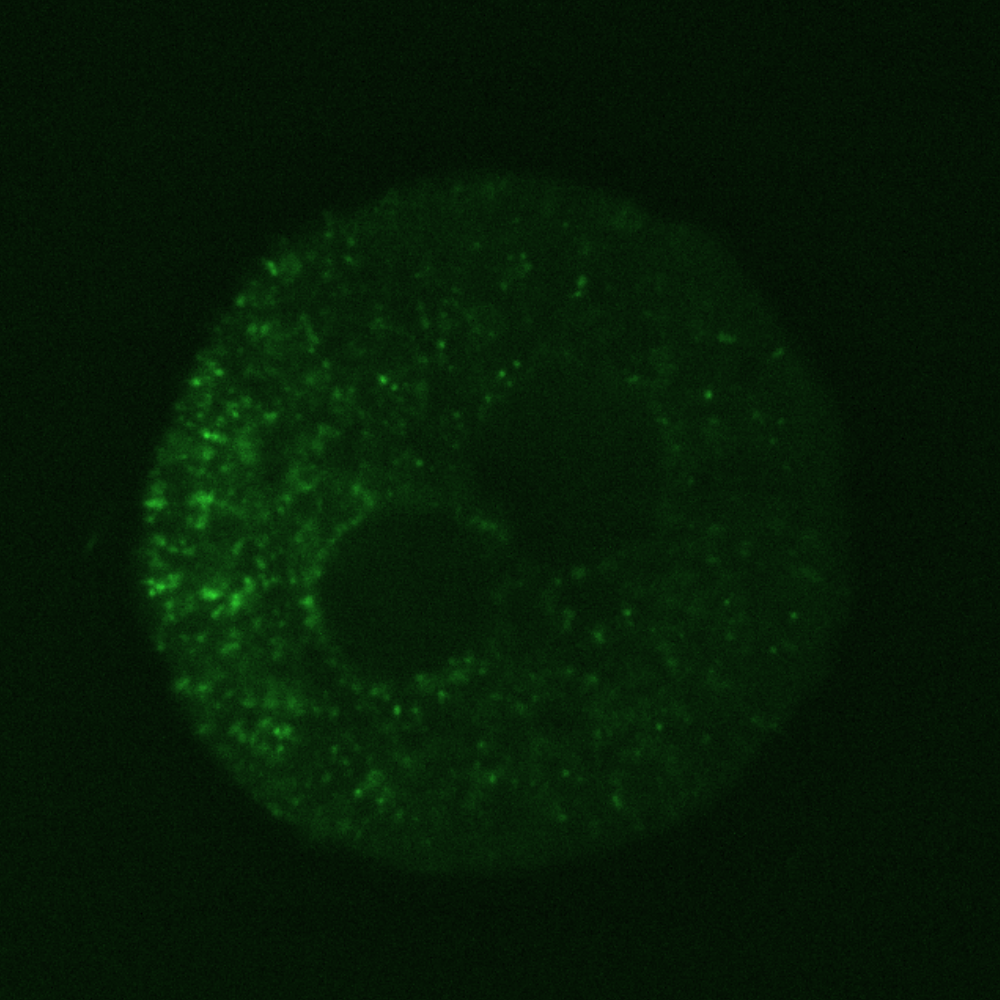

Supplement: Supplementary file 7 — Source data Fig. 5 [file 44319_2024_267_MOESM7_ESM.zip › Figure 5/5G/H2O2-WT-Left.tif]

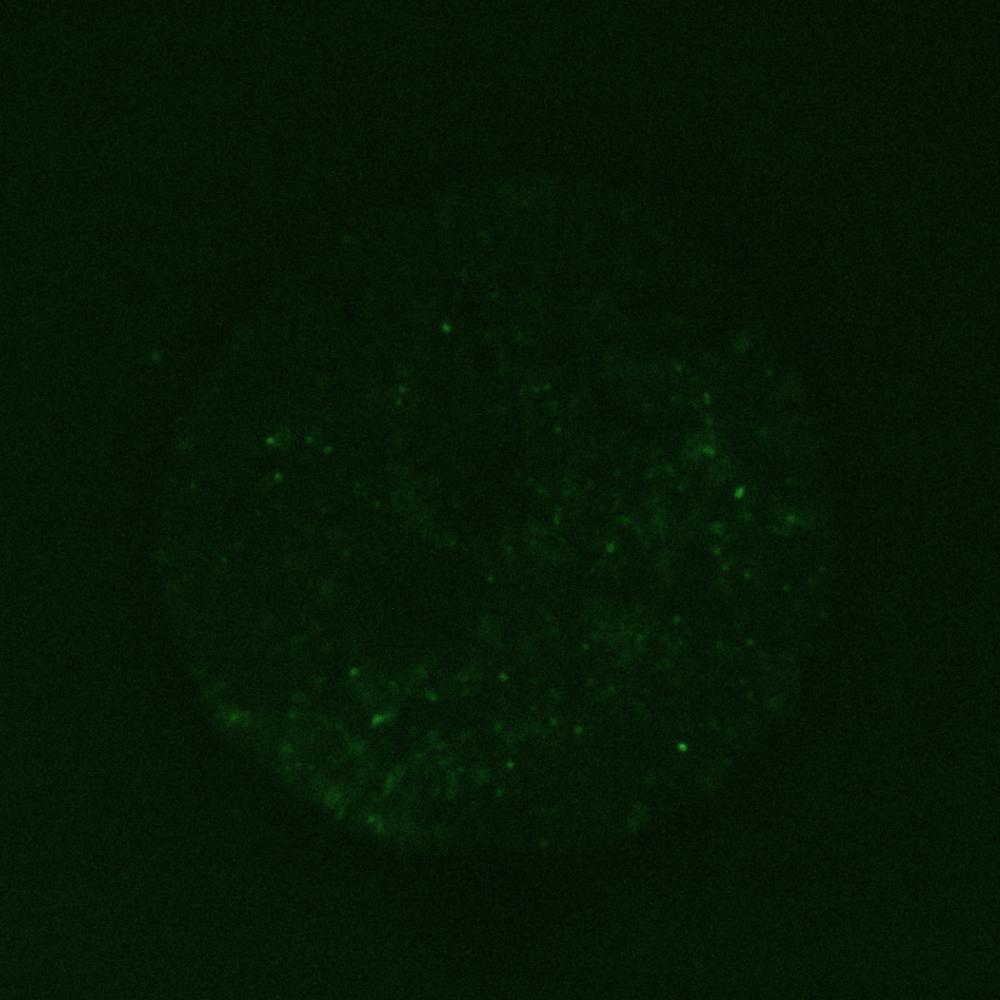

Supplement: Supplementary file 7 — Source data Fig. 5 [file 44319_2024_267_MOESM7_ESM.zip › Figure 5/5G/H2O2-WT-Right.tif]

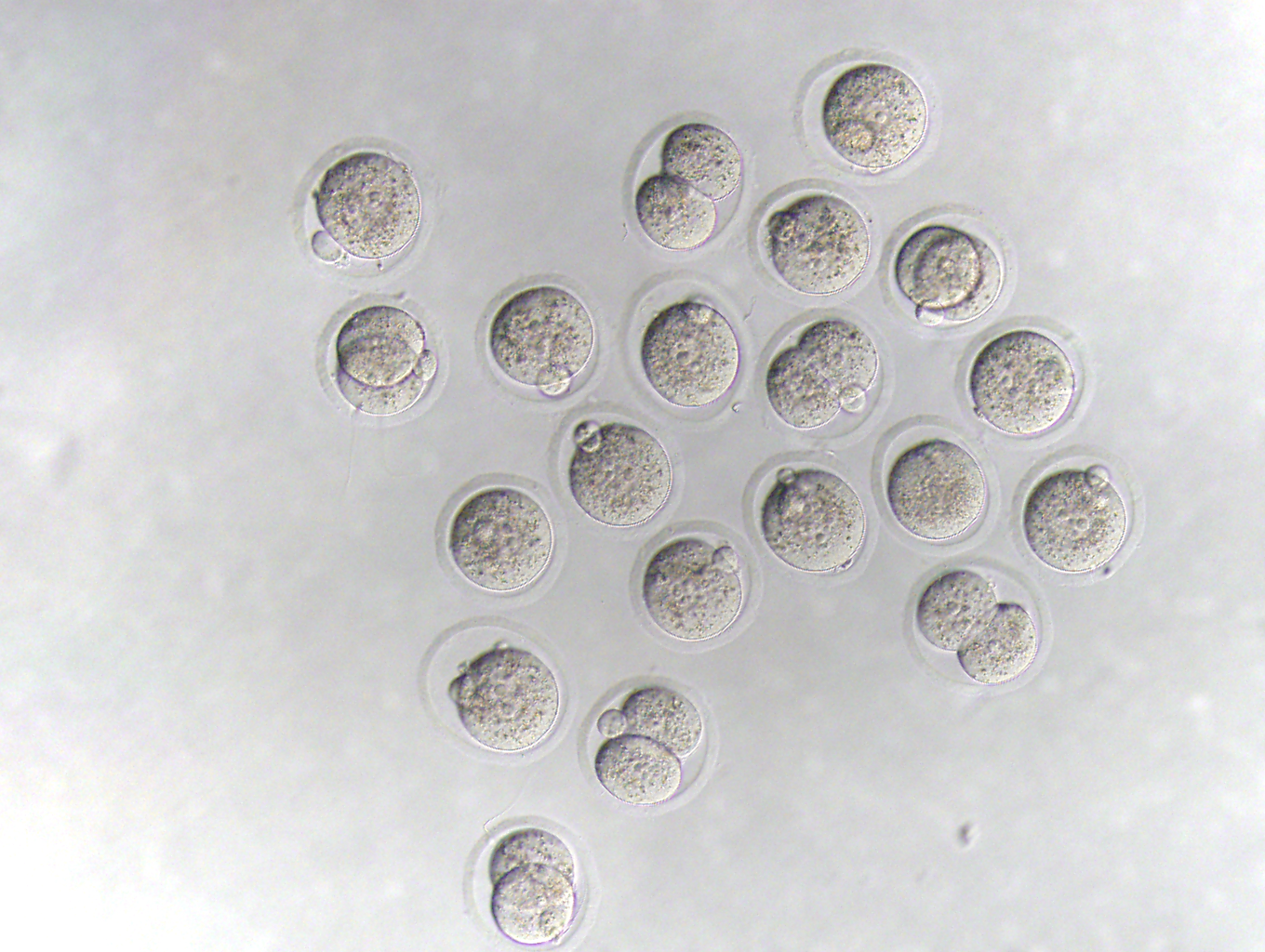

Supplement: Supplementary file 8 — Source data Fig. 6 [file 44319_2024_267_MOESM8_ESM.zip › Figure 6/6B/100uM_EGCG-Brightfield.tif]

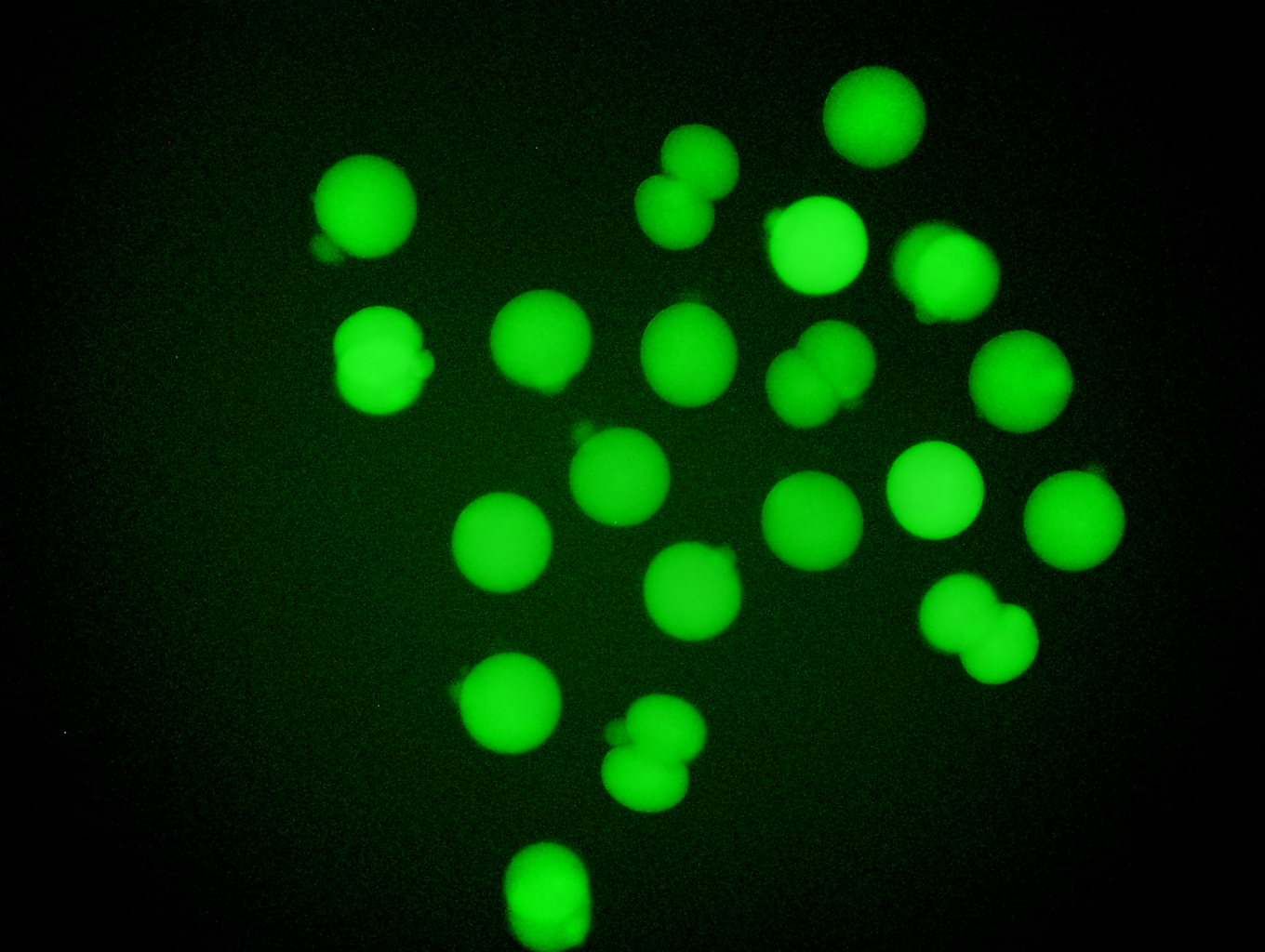

Supplement: Supplementary file 8 — Source data Fig. 6 [file 44319_2024_267_MOESM8_ESM.zip › Figure 6/6B/100uM_EGCG-EGFP.tif]

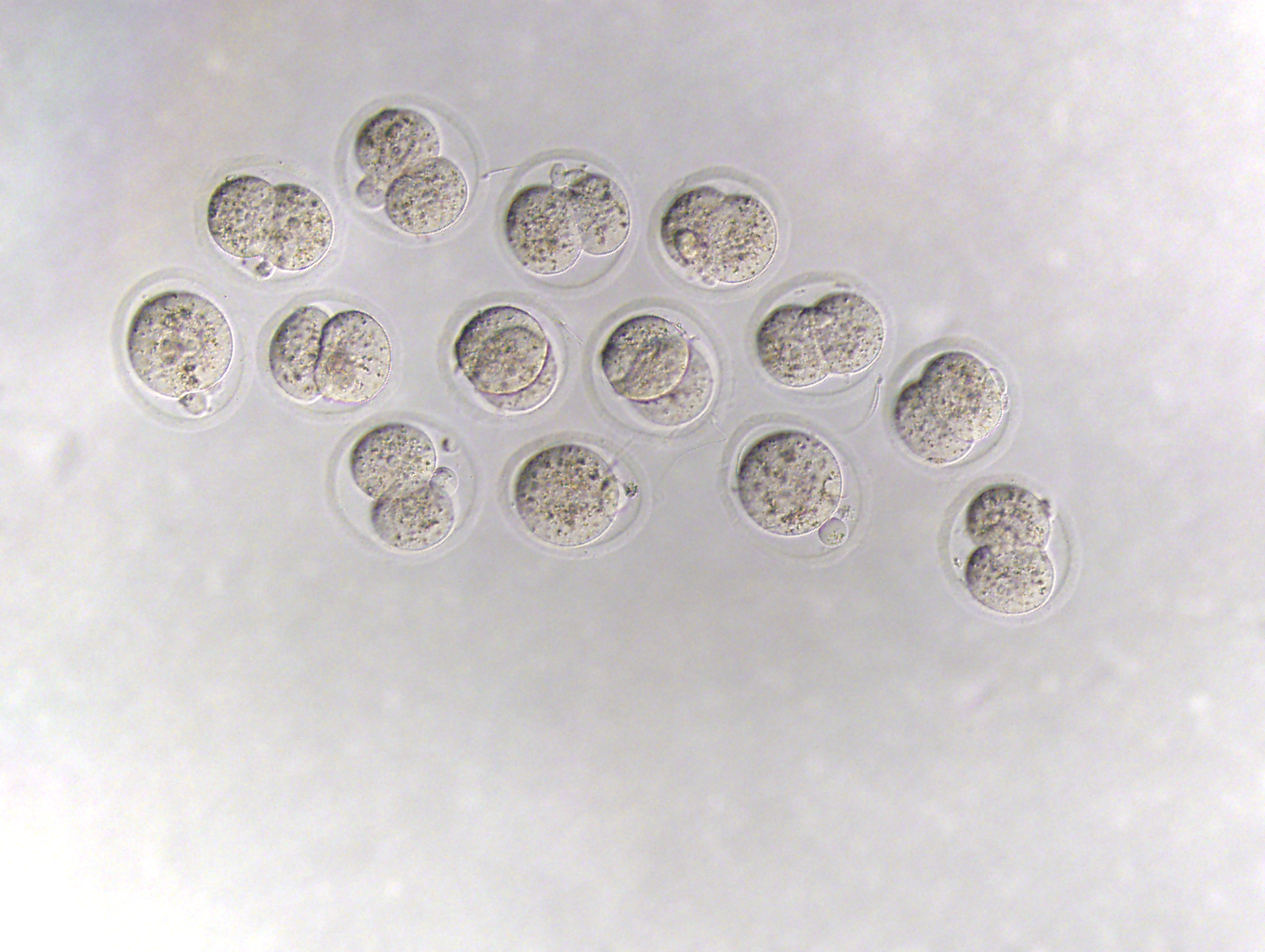

Supplement: Supplementary file 8 — Source data Fig. 6 [file 44319_2024_267_MOESM8_ESM.zip › Figure 6/6B/10uM_EGCG-Brightfield.tif]

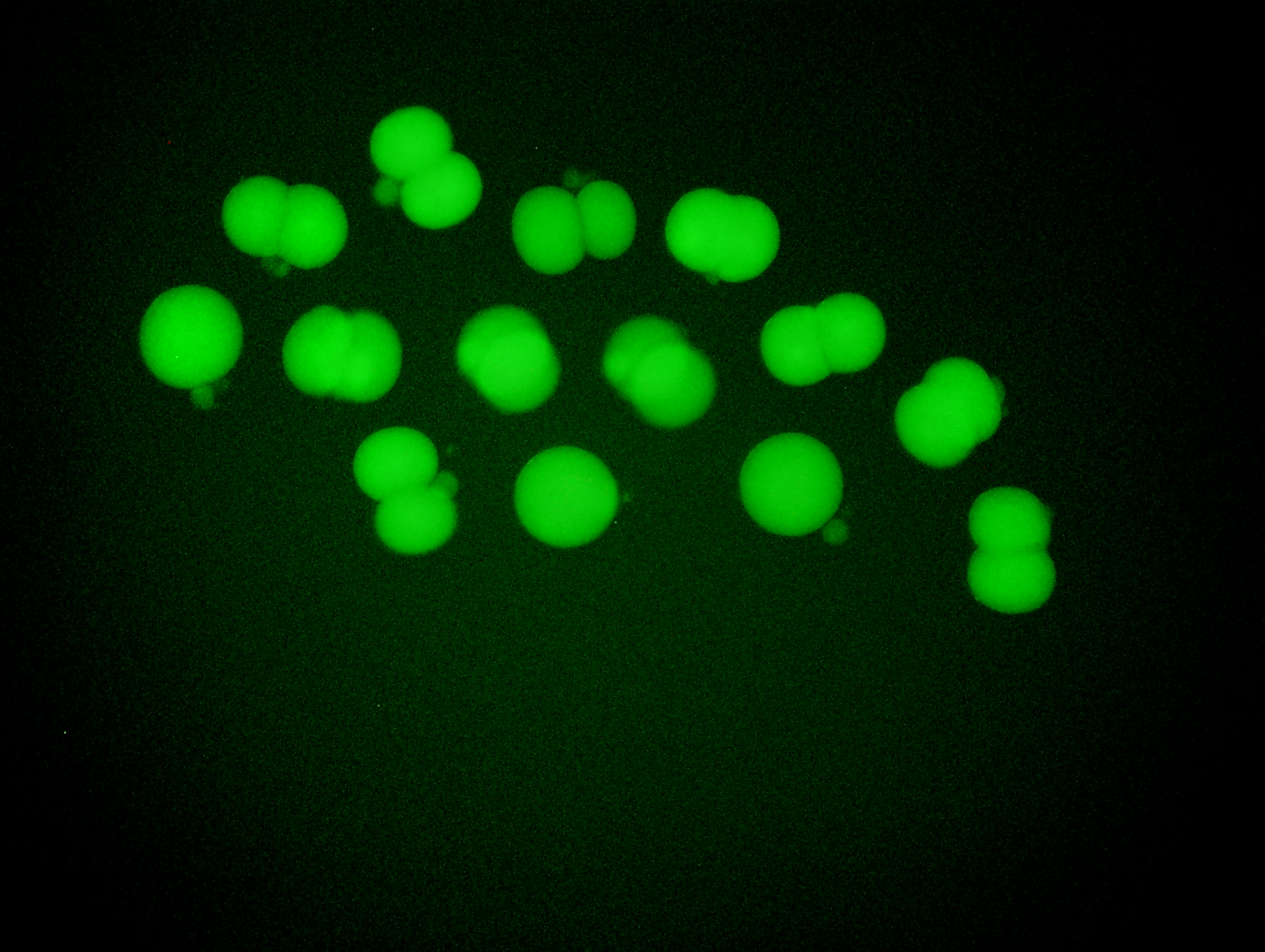

Supplement: Supplementary file 8 — Source data Fig. 6 [file 44319_2024_267_MOESM8_ESM.zip › Figure 6/6B/10uM_EGCG-EGFP.tif]

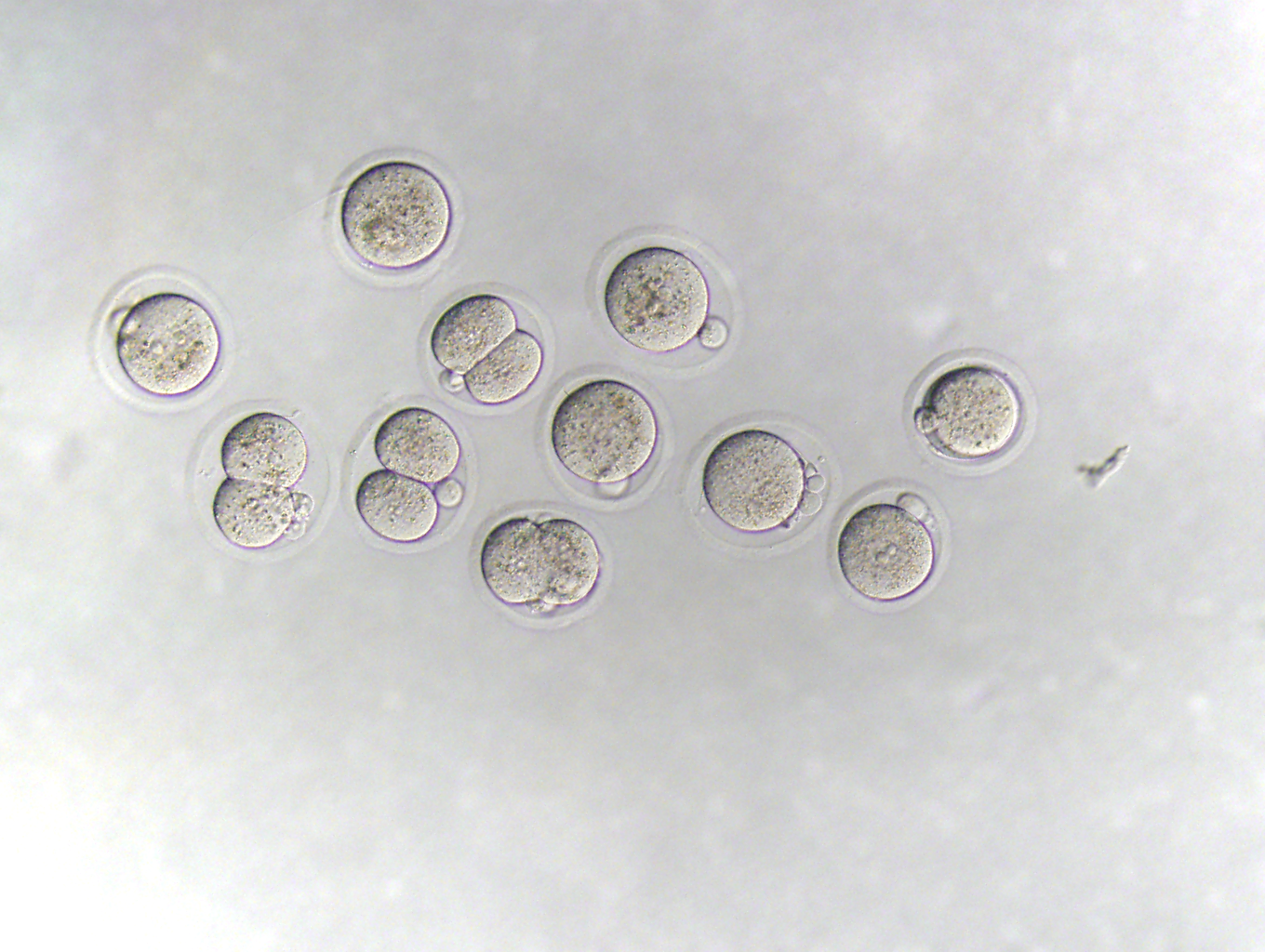

Supplement: Supplementary file 8 — Source data Fig. 6 [file 44319_2024_267_MOESM8_ESM.zip › Figure 6/6B/1uM_EGCG-Brightfield.tif]

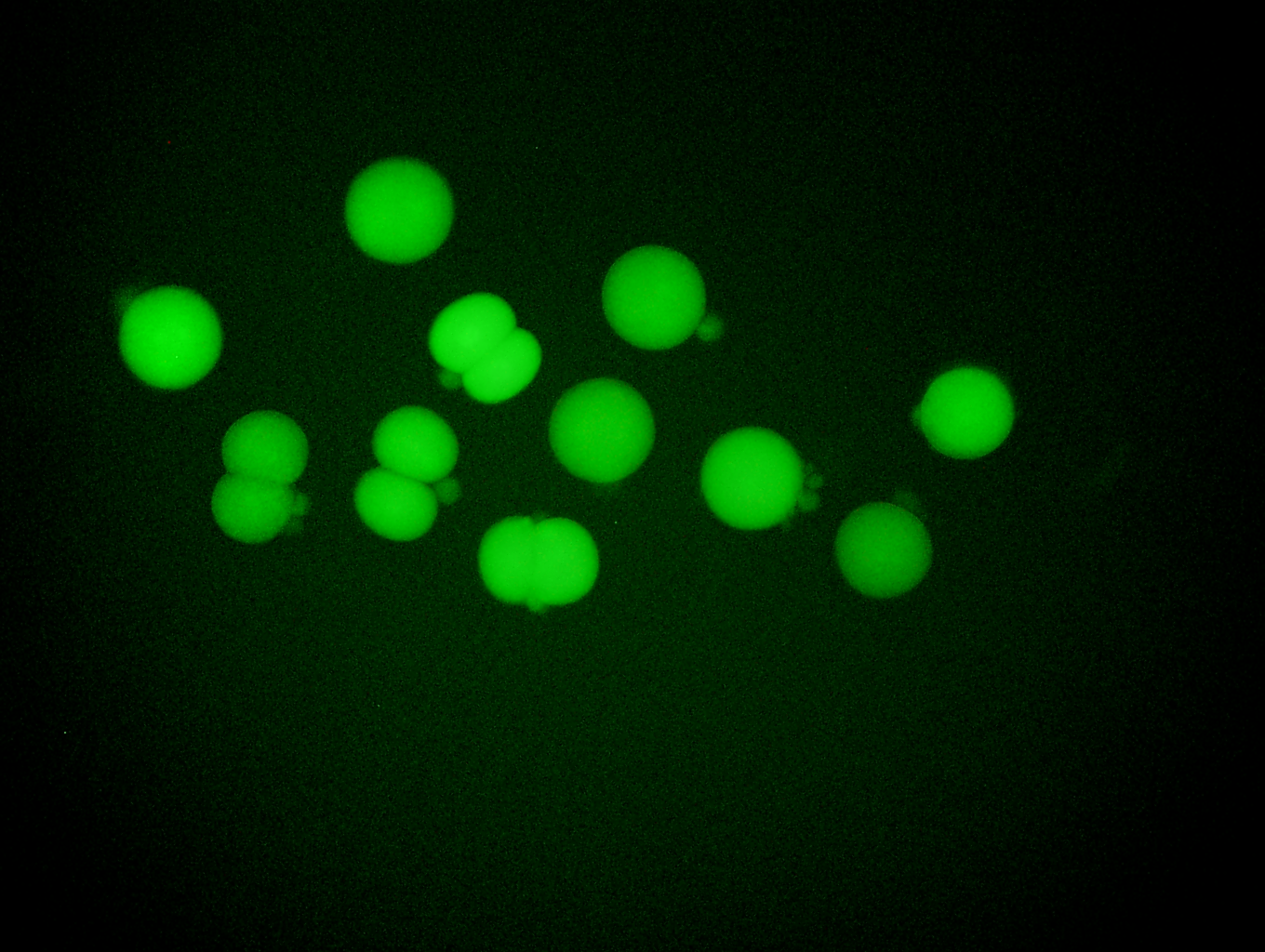

Supplement: Supplementary file 8 — Source data Fig. 6 [file 44319_2024_267_MOESM8_ESM.zip › Figure 6/6B/1uM_EGCG-EGFP.tif]

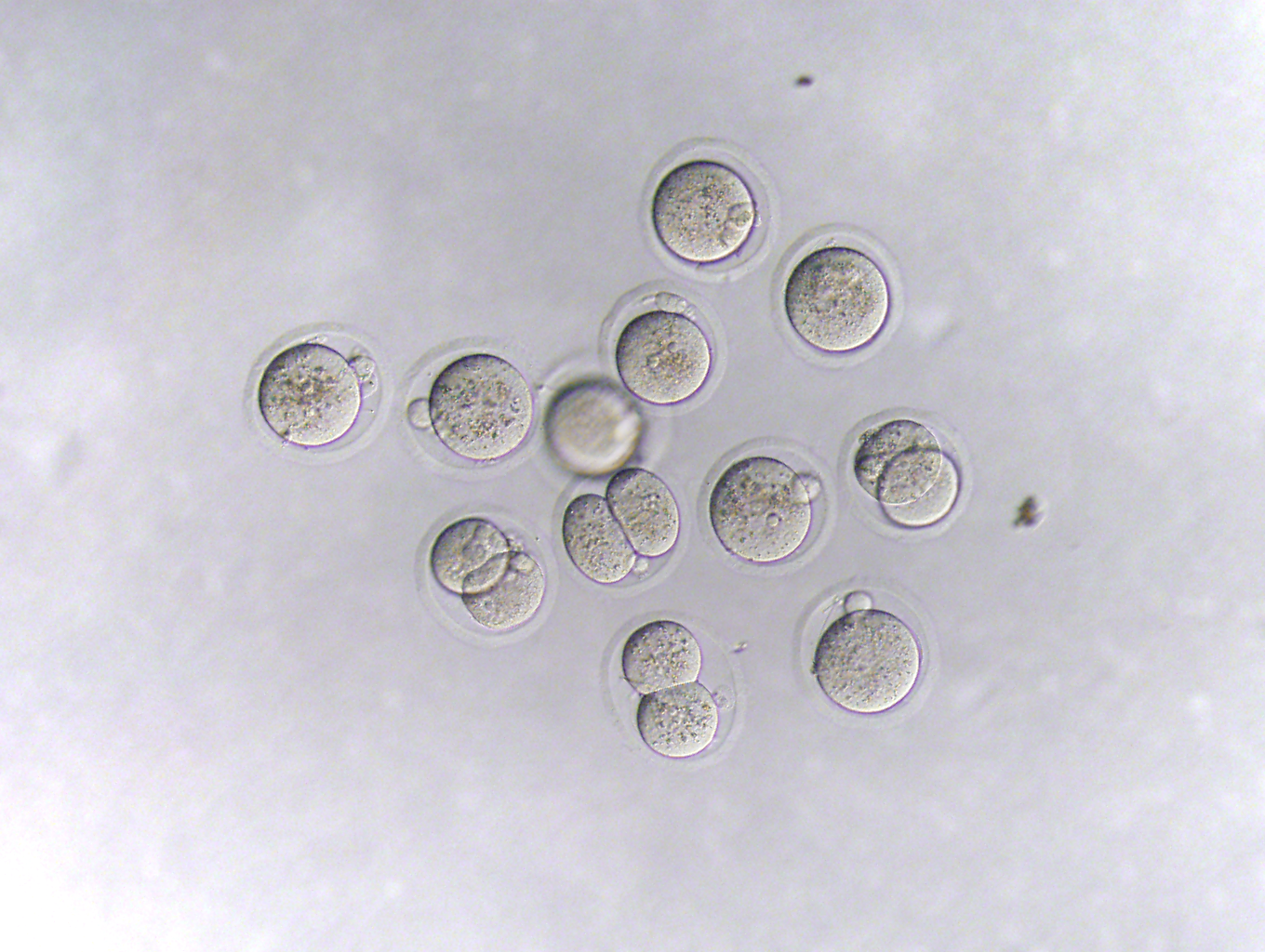

Supplement: Supplementary file 8 — Source data Fig. 6 [file 44319_2024_267_MOESM8_ESM.zip › Figure 6/6B/DMSO-Brightfield.tif]

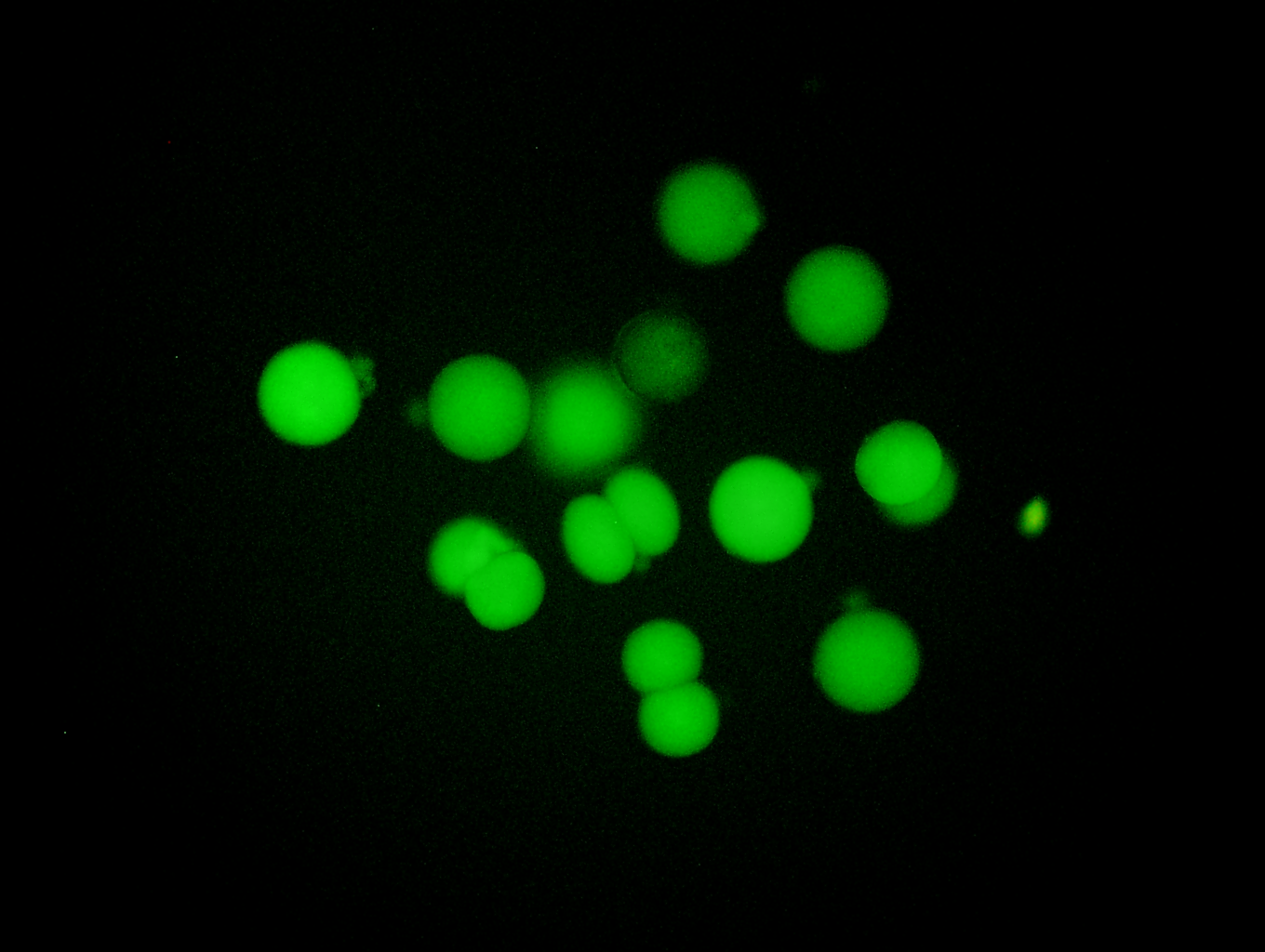

Supplement: Supplementary file 8 — Source data Fig. 6 [file 44319_2024_267_MOESM8_ESM.zip › Figure 6/6B/DMSO-EGFP.tif]

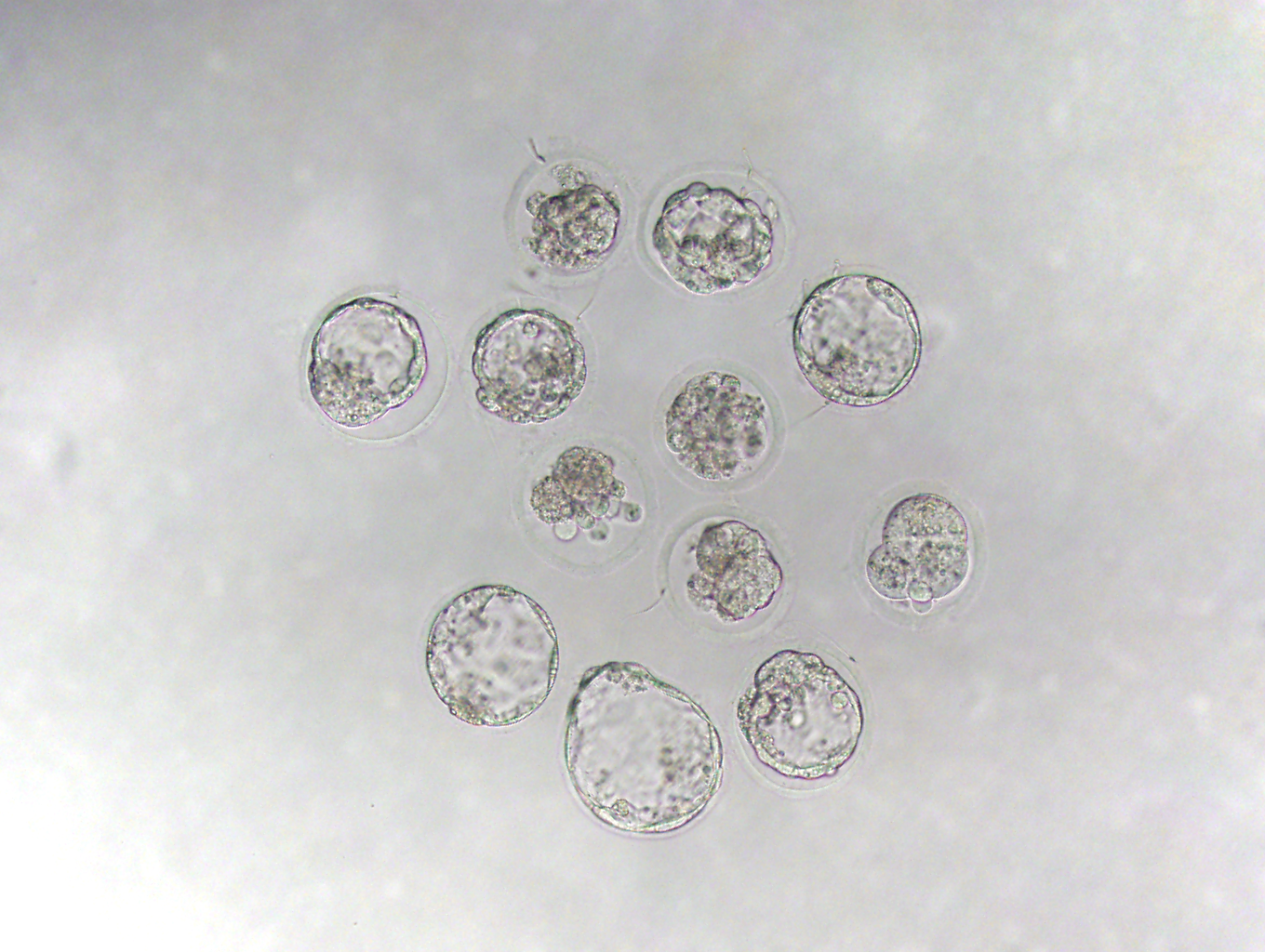

Supplement: Supplementary file 8 — Source data Fig. 6 [file 44319_2024_267_MOESM8_ESM.zip › Figure 6/6C/100uM_Brightfield.tif]

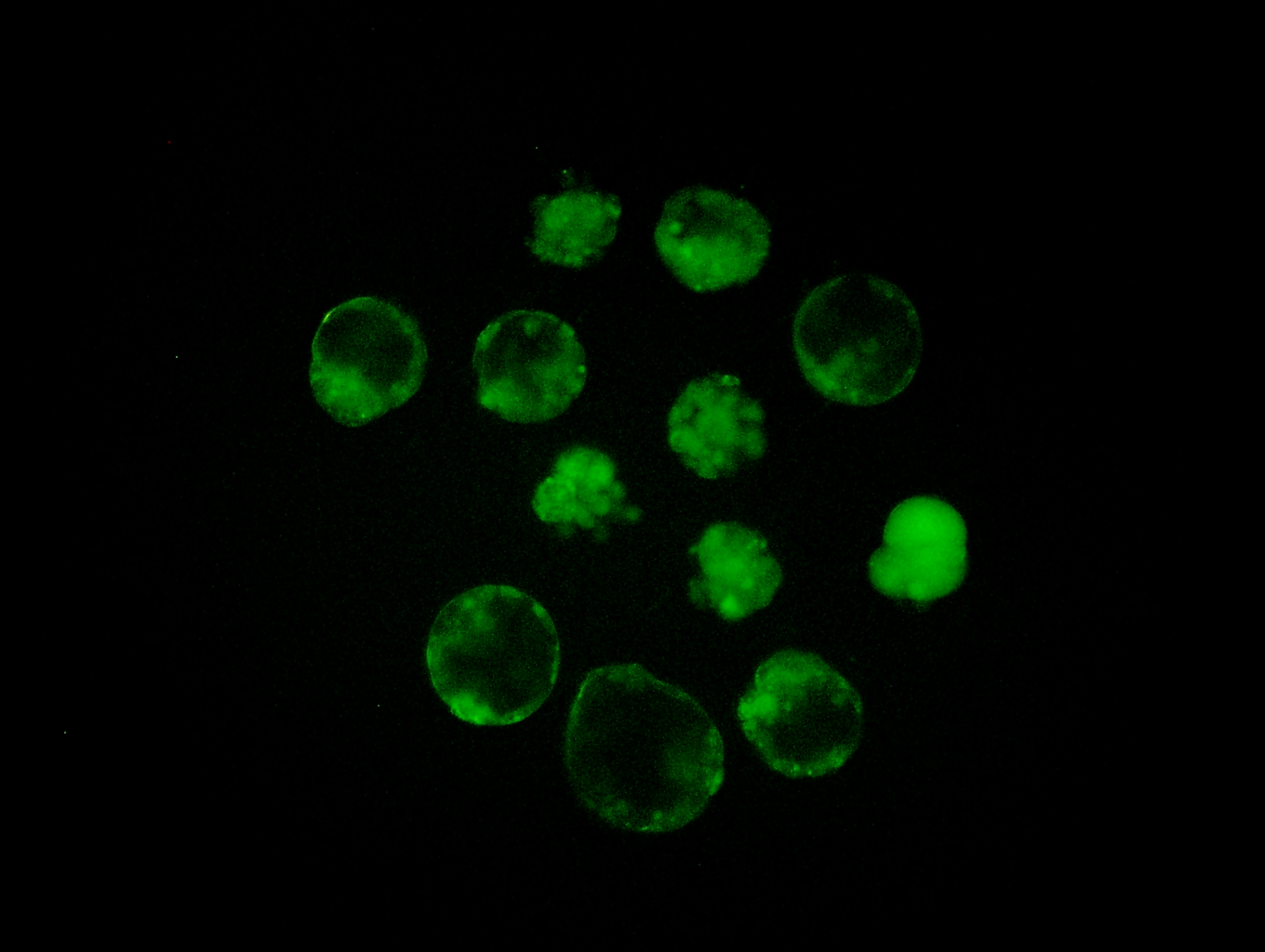

Supplement: Supplementary file 8 — Source data Fig. 6 [file 44319_2024_267_MOESM8_ESM.zip › Figure 6/6C/100uM_EGCG-EGFP.tif]

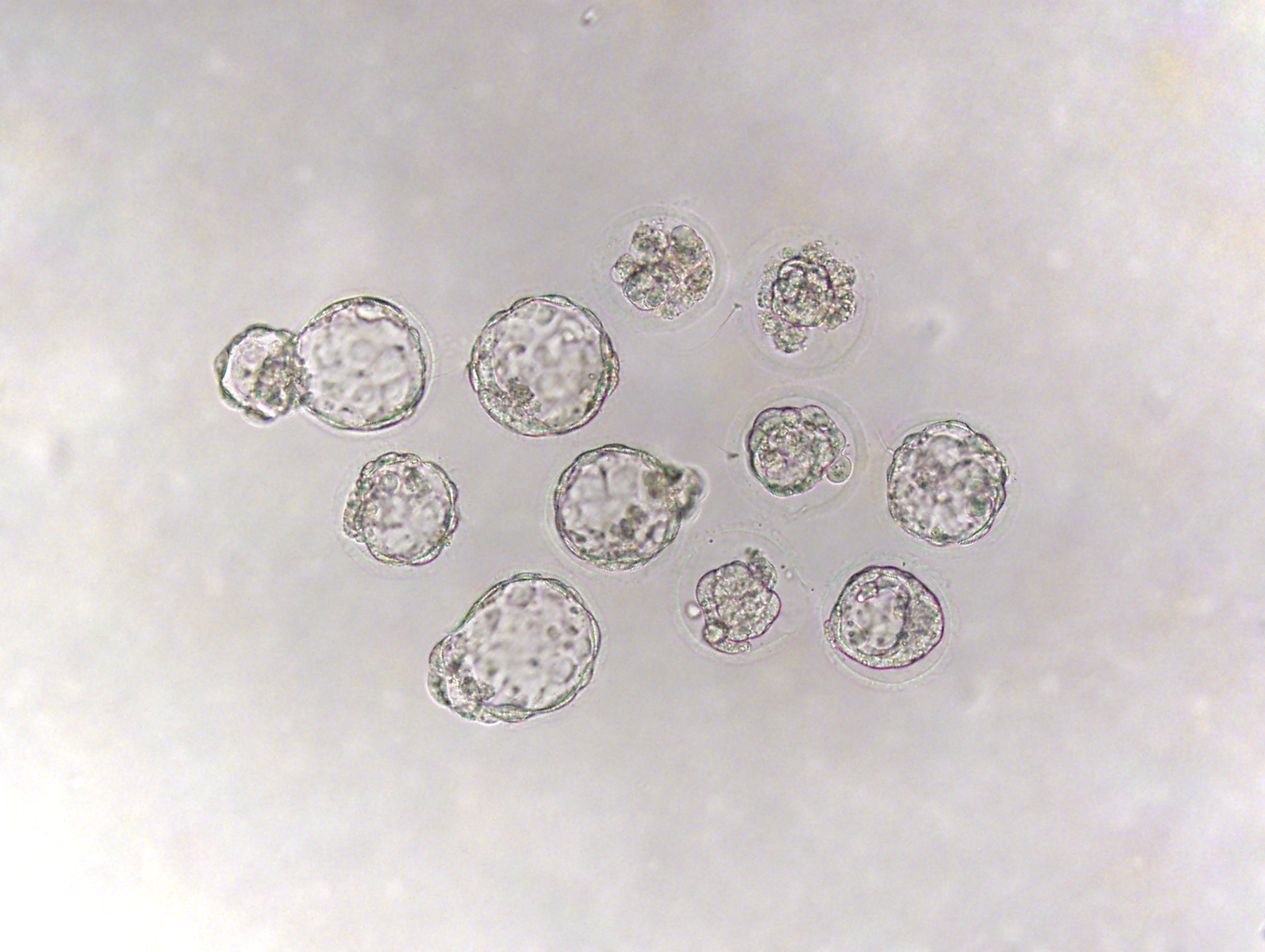

Supplement: Supplementary file 8 — Source data Fig. 6 [file 44319_2024_267_MOESM8_ESM.zip › Figure 6/6C/10uM_EGCG-Brightfield.tif]

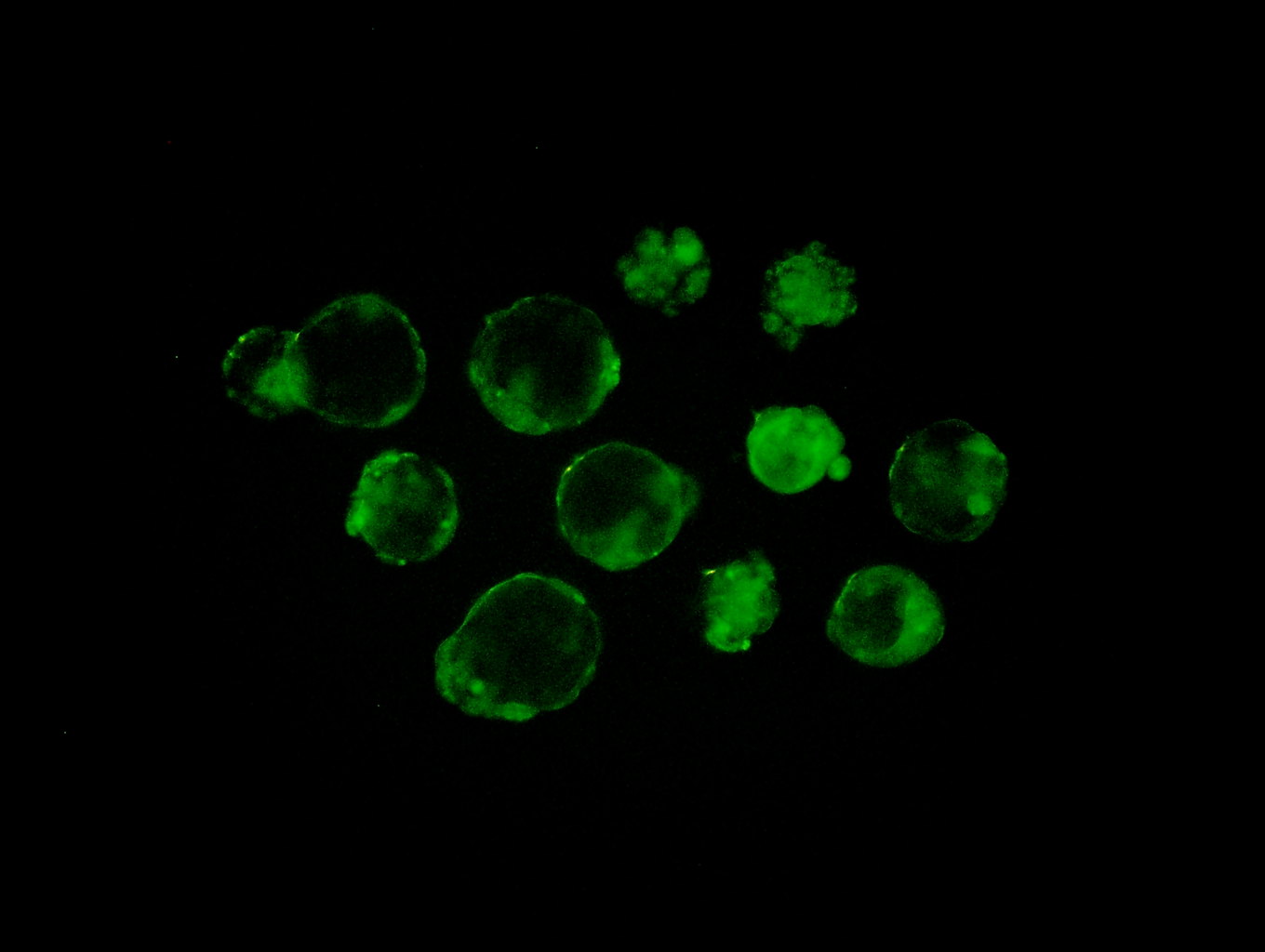

Supplement: Supplementary file 8 — Source data Fig. 6 [file 44319_2024_267_MOESM8_ESM.zip › Figure 6/6C/10uM_EGCG-EGFP.tif]

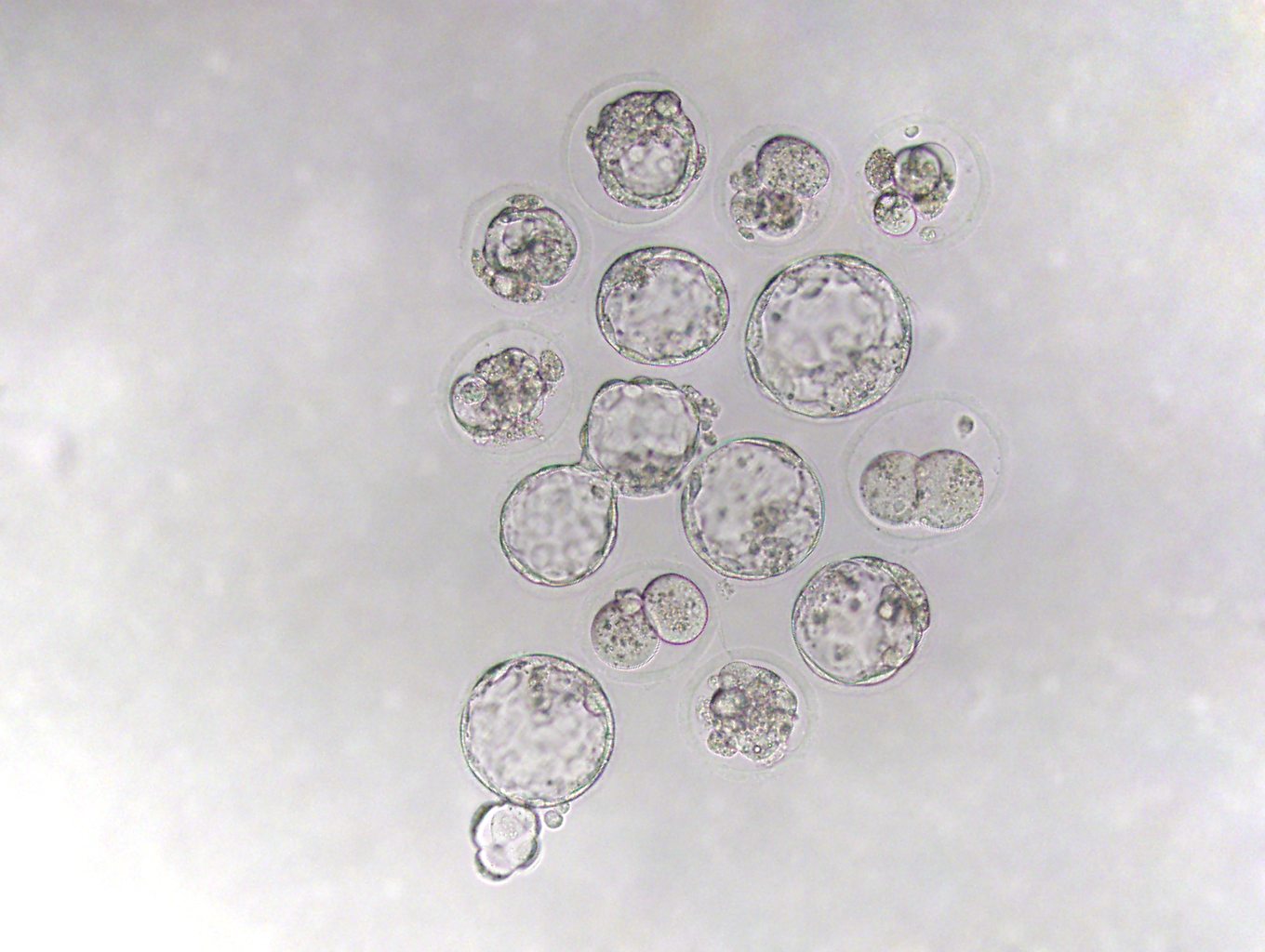

Supplement: Supplementary file 8 — Source data Fig. 6 [file 44319_2024_267_MOESM8_ESM.zip › Figure 6/6C/1uM_EGCG-Brightfield.tif]

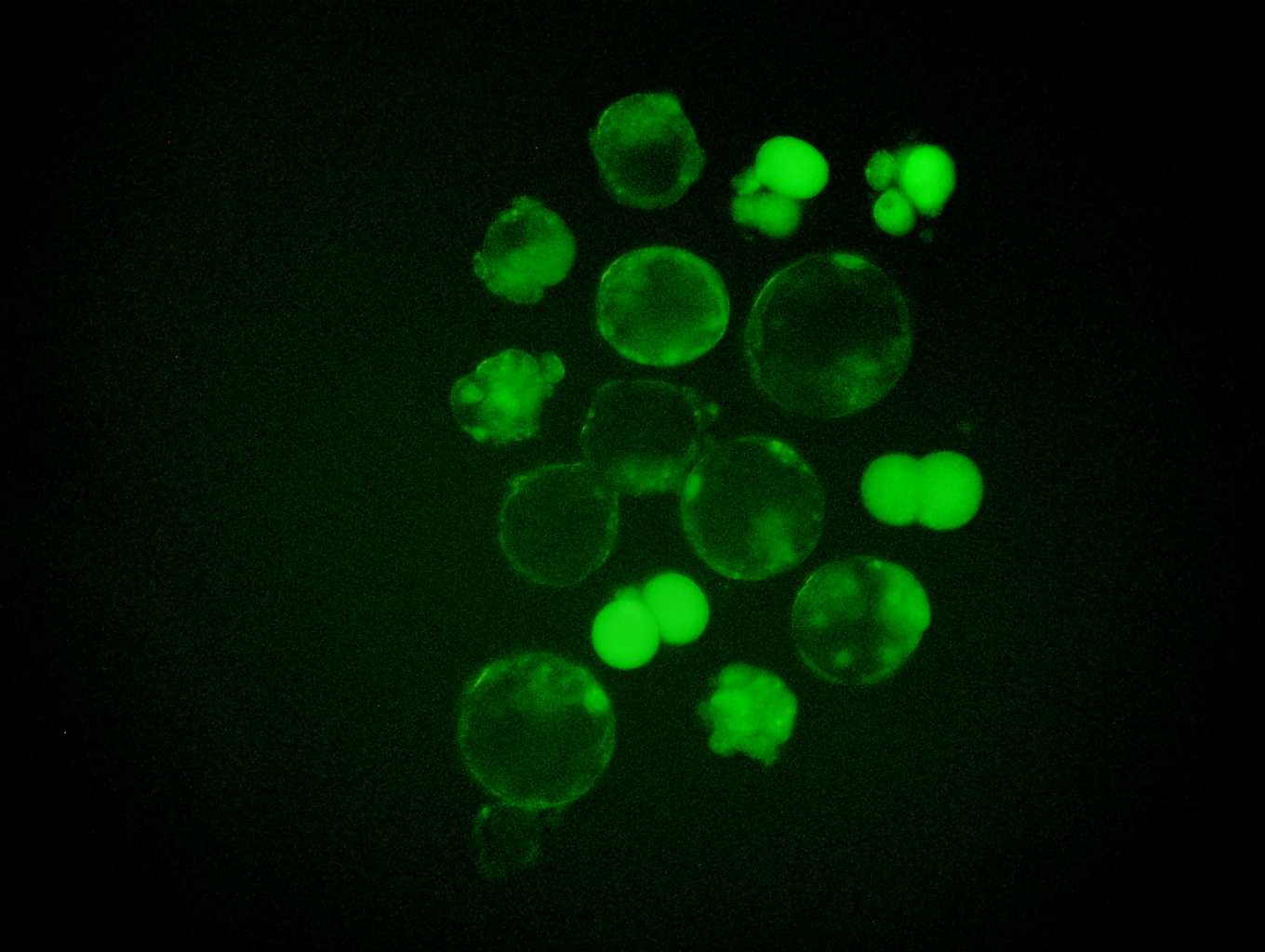

Supplement: Supplementary file 8 — Source data Fig. 6 [file 44319_2024_267_MOESM8_ESM.zip › Figure 6/6C/1uM_EGCG-EGFP.tif]

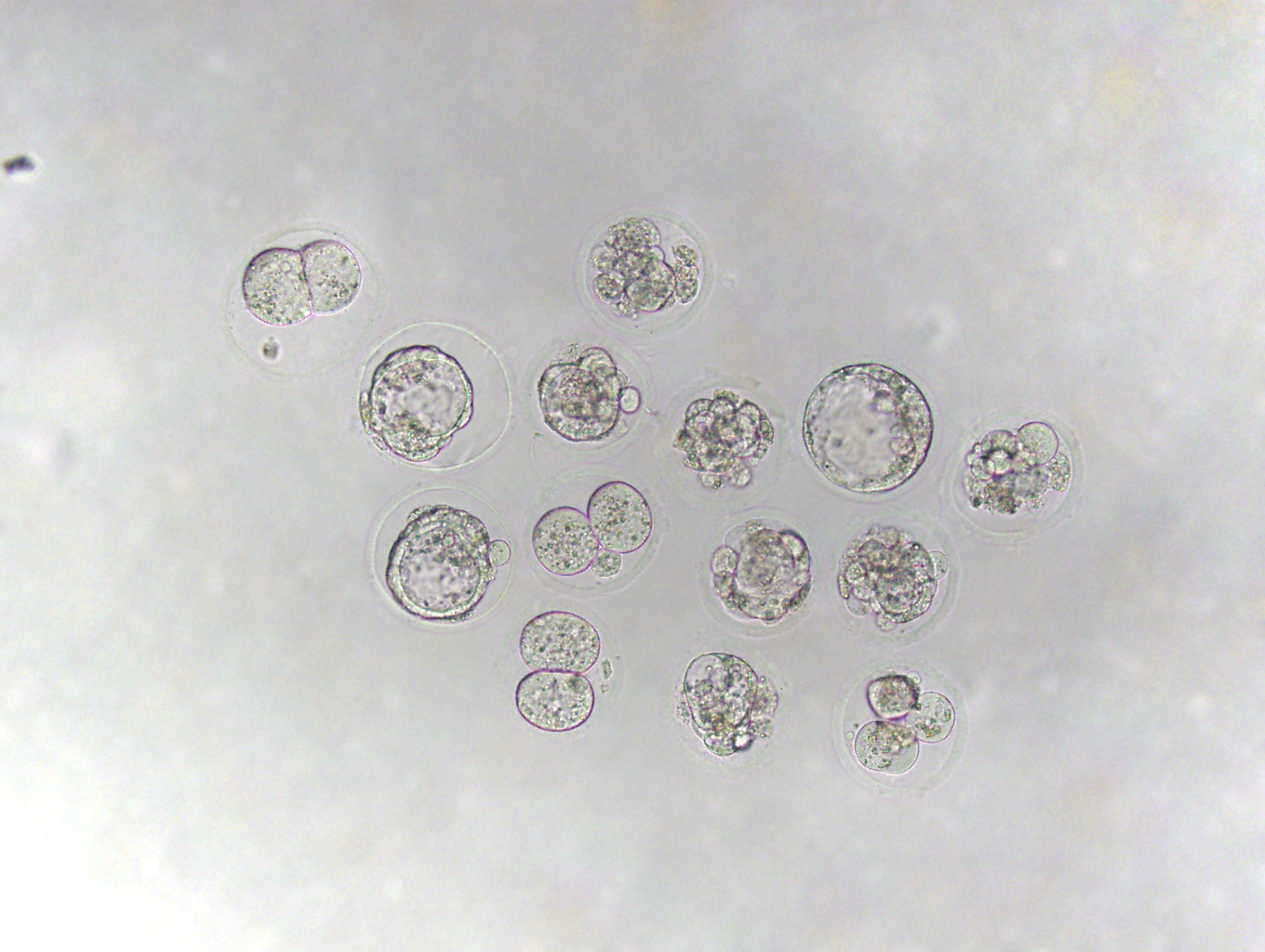

Supplement: Supplementary file 8 — Source data Fig. 6 [file 44319_2024_267_MOESM8_ESM.zip › Figure 6/6C/DMSO-Brightfield.tif]

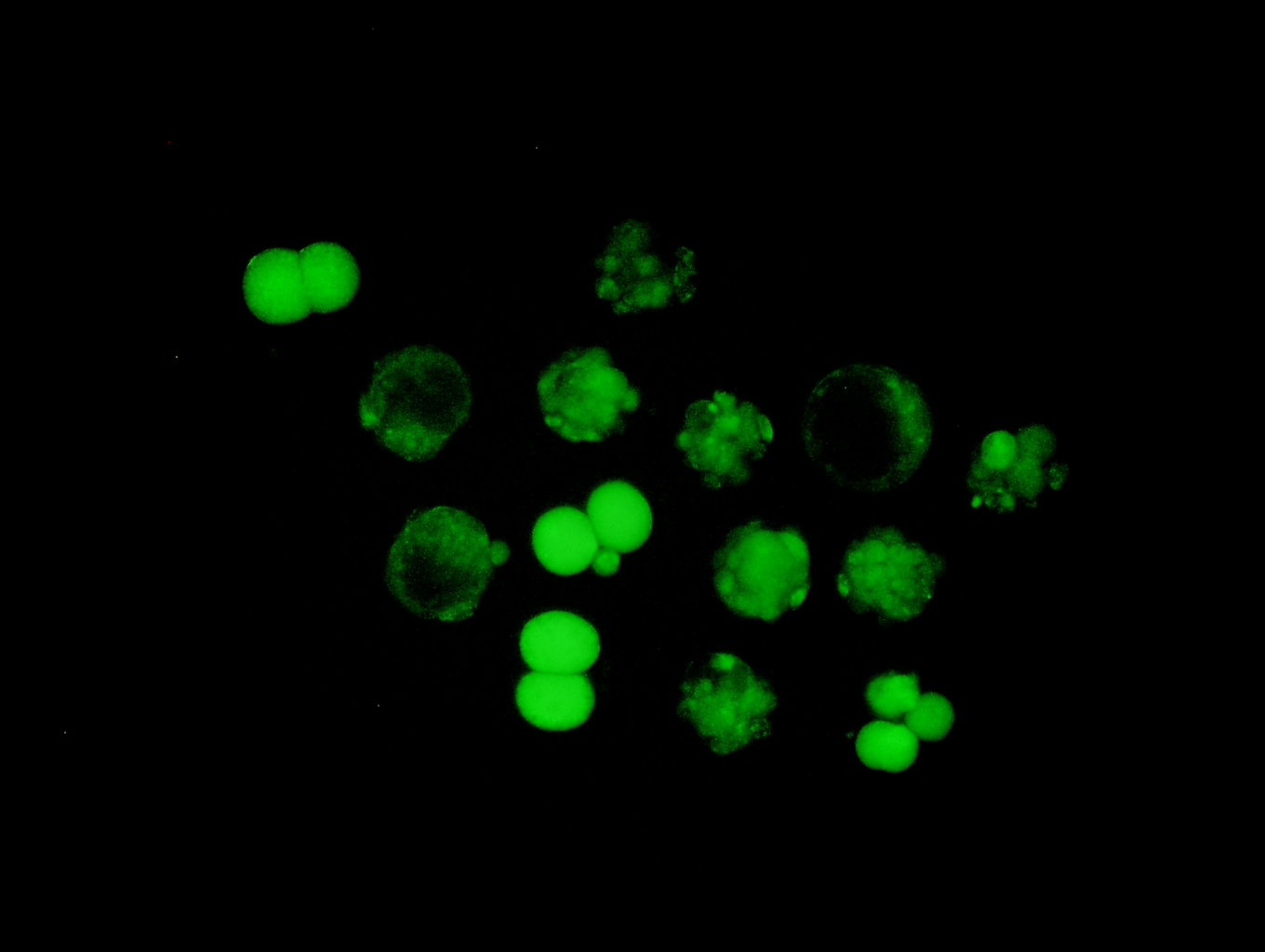

Supplement: Supplementary file 8 — Source data Fig. 6 [file 44319_2024_267_MOESM8_ESM.zip › Figure 6/6C/DMSO-EGFP.tif]

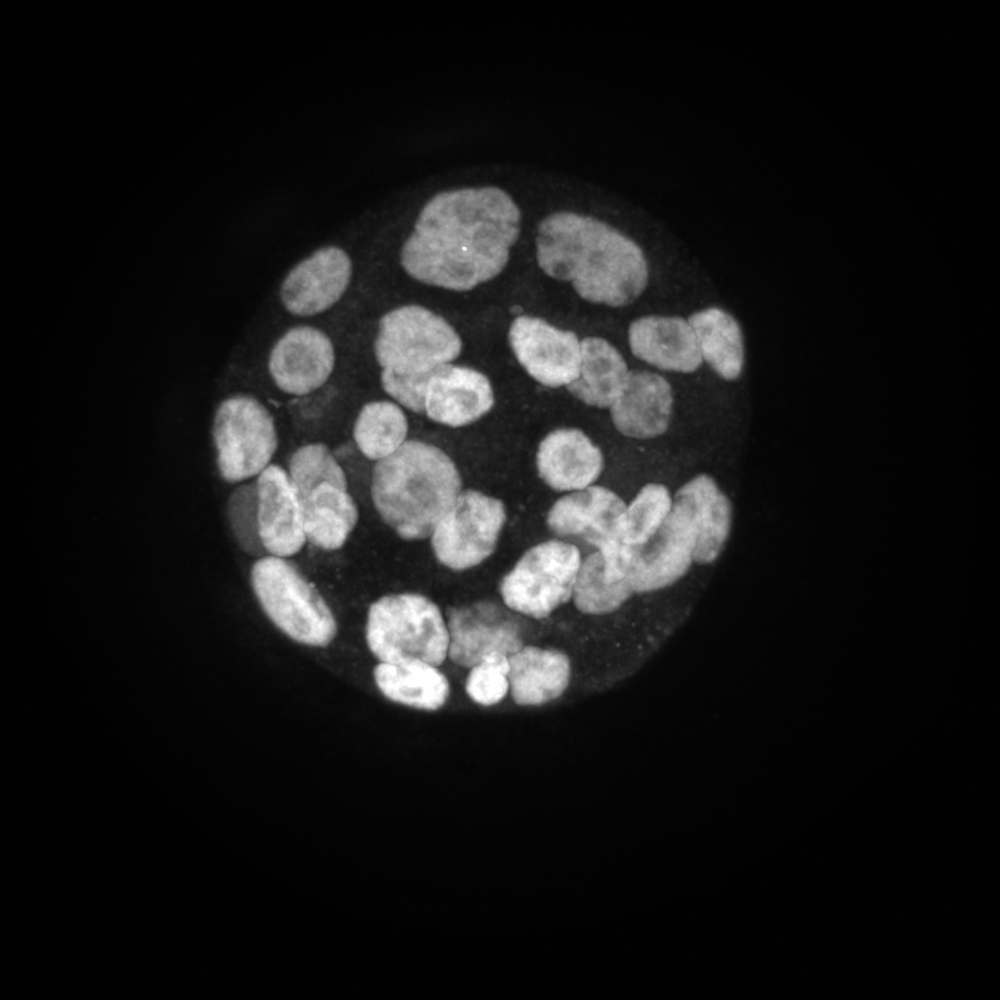

Supplement: Supplementary file 8 — Source data Fig. 6 [file 44319_2024_267_MOESM8_ESM.zip › Figure 6/6F/IF-F441-Cdx2.tif]

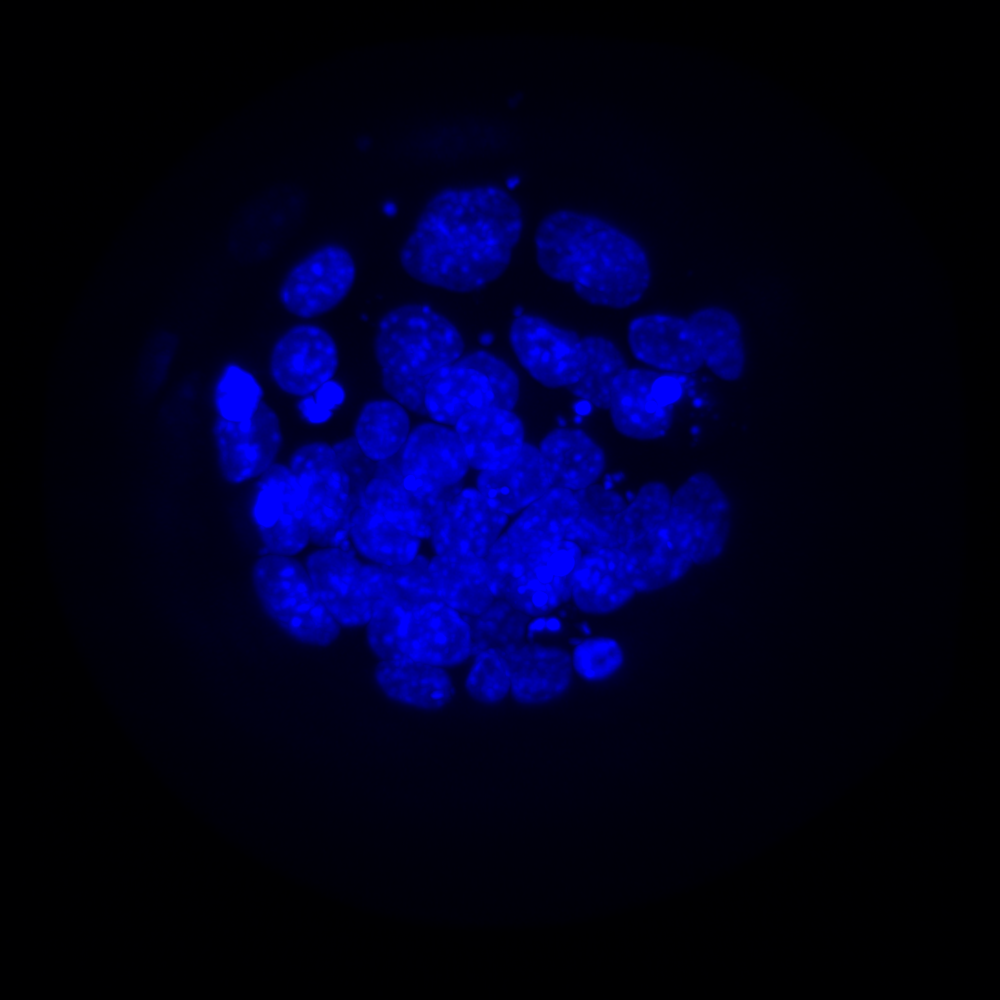

Supplement: Supplementary file 8 — Source data Fig. 6 [file 44319_2024_267_MOESM8_ESM.zip › Figure 6/6F/IF-F441-DAPI.tif]

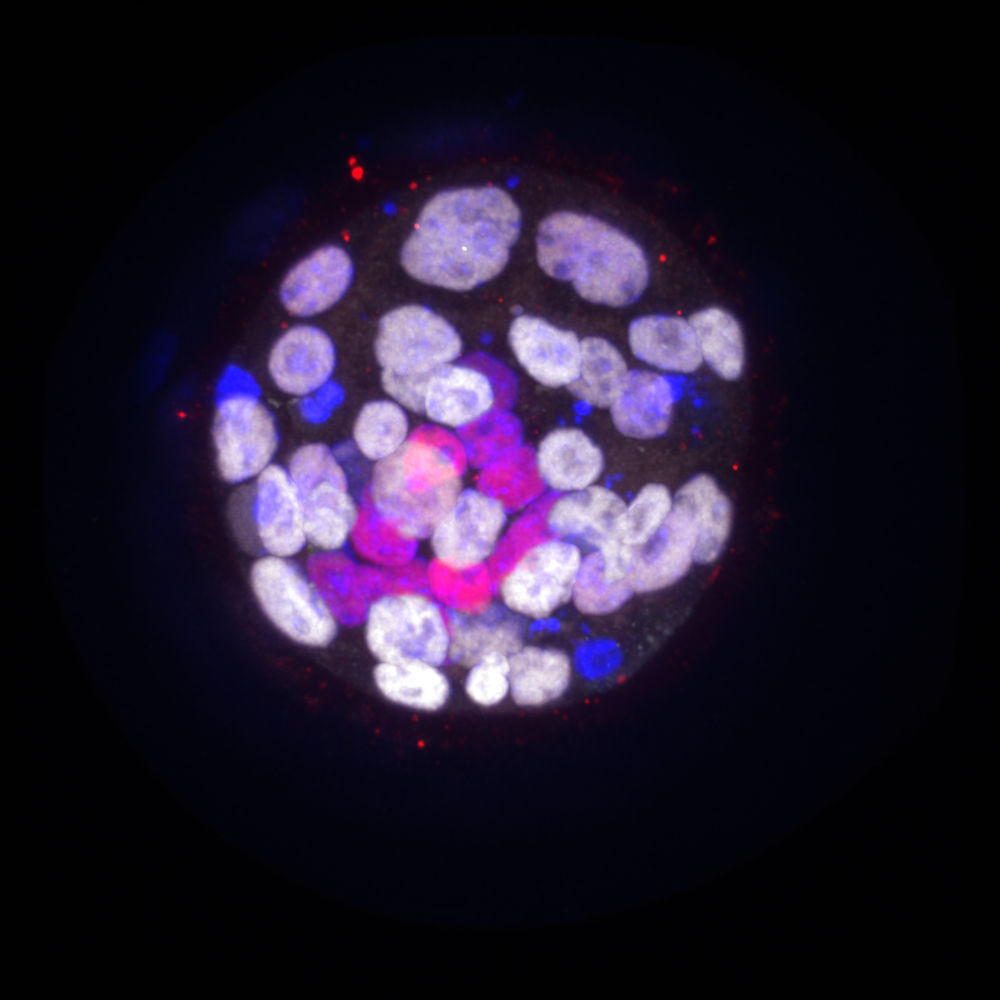

Supplement: Supplementary file 8 — Source data Fig. 6 [file 44319_2024_267_MOESM8_ESM.zip › Figure 6/6F/IF-F441-Merge.tif]

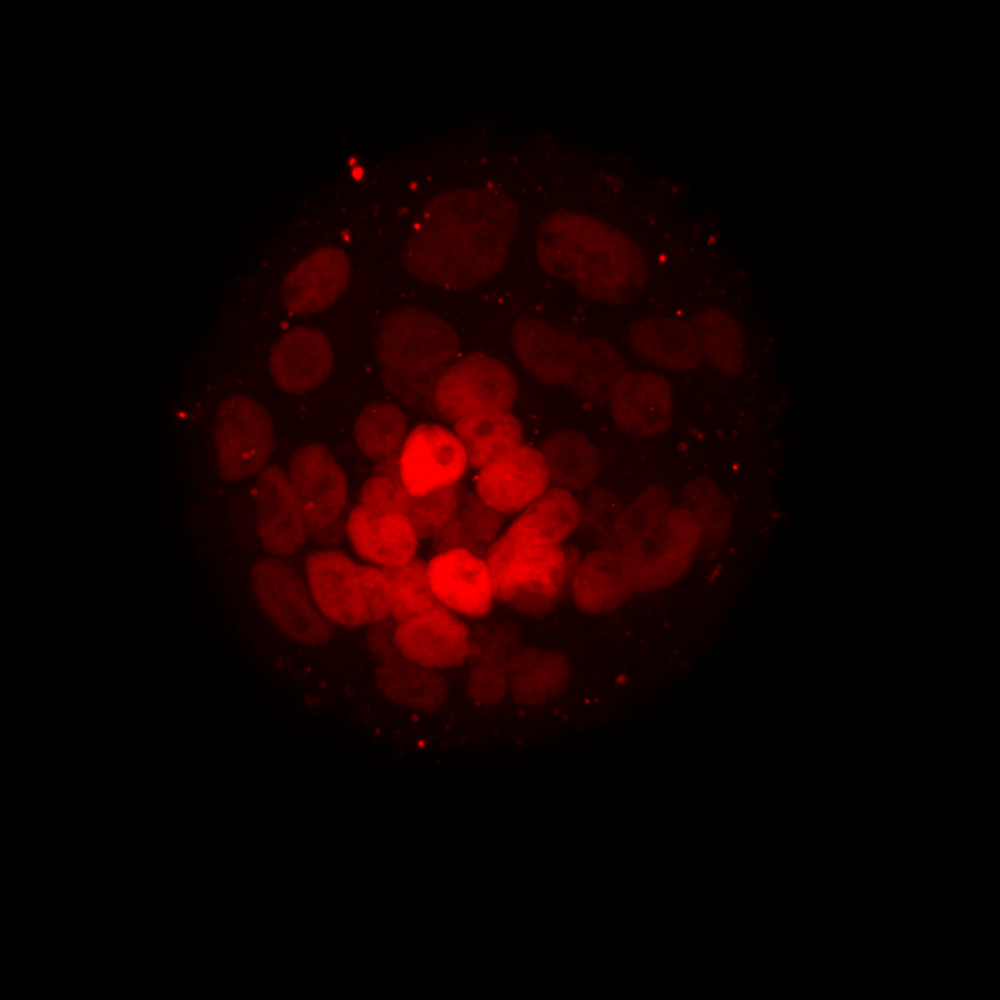

Supplement: Supplementary file 8 — Source data Fig. 6 [file 44319_2024_267_MOESM8_ESM.zip › Figure 6/6F/IF-F441-Oct3 4.tif]

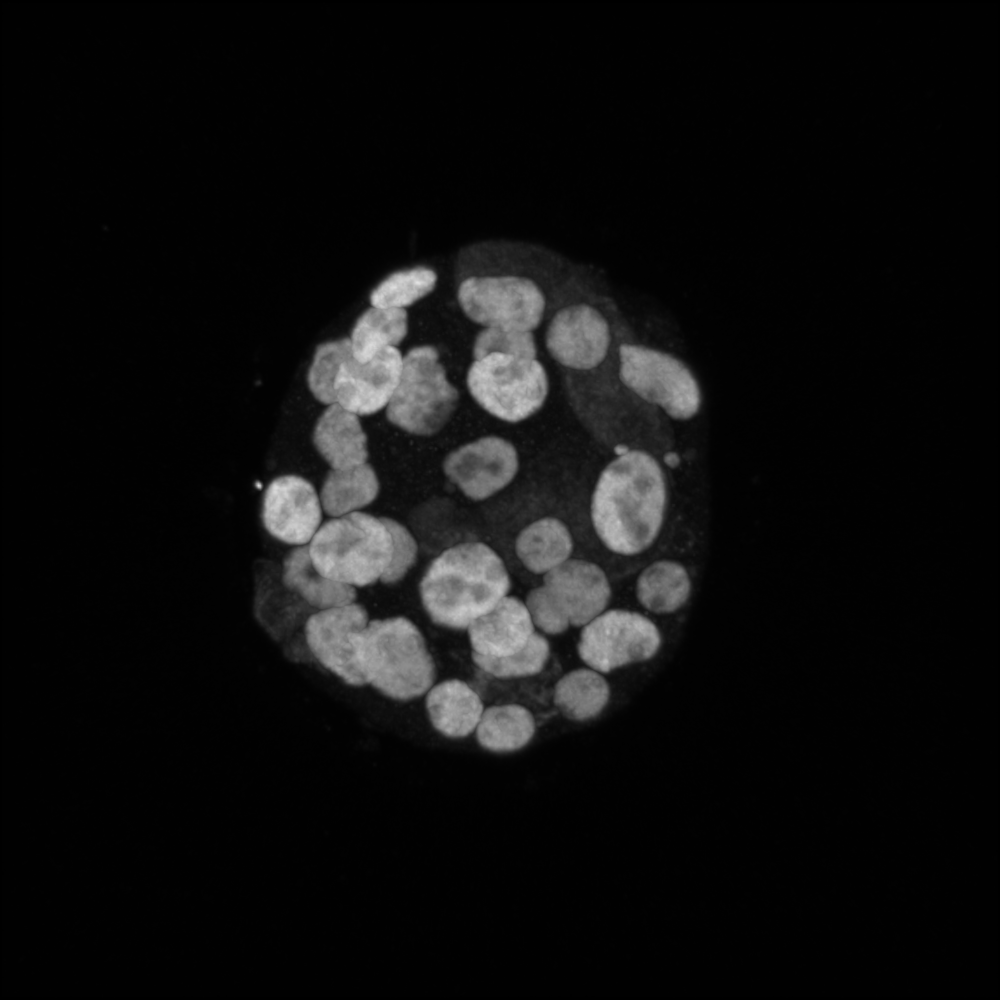

Supplement: Supplementary file 8 — Source data Fig. 6 [file 44319_2024_267_MOESM8_ESM.zip › Figure 6/6F/IF-WT-Cdx2.tif]

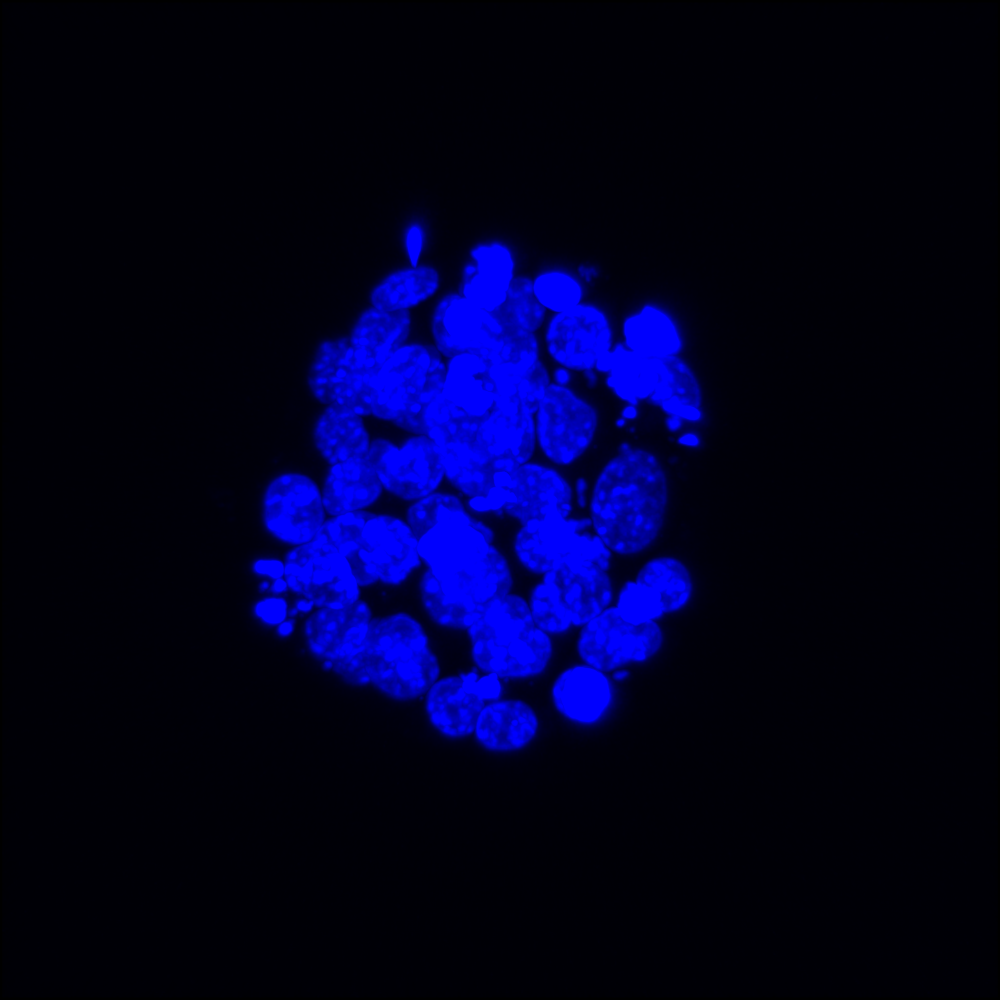

Supplement: Supplementary file 8 — Source data Fig. 6 [file 44319_2024_267_MOESM8_ESM.zip › Figure 6/6F/IF-WT-DAPI.tif]

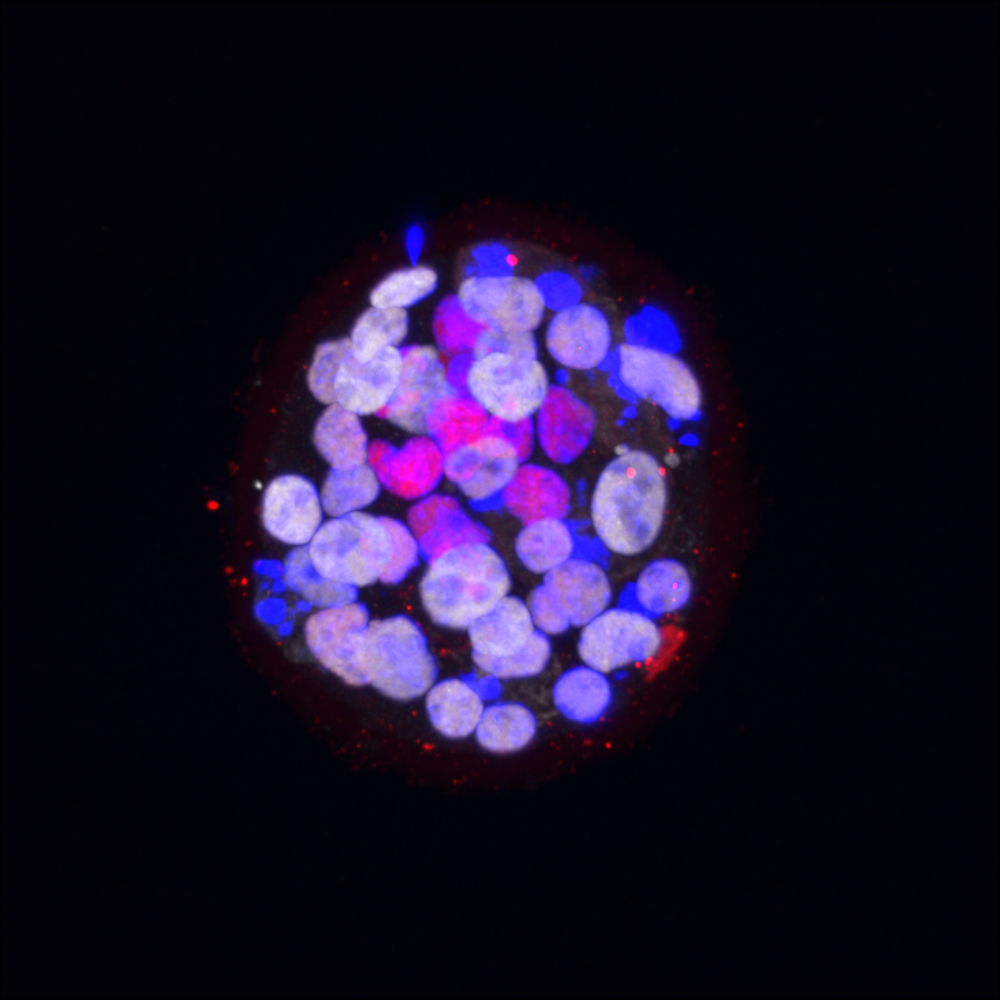

Supplement: Supplementary file 8 — Source data Fig. 6 [file 44319_2024_267_MOESM8_ESM.zip › Figure 6/6F/IF-WT-Merge.tif]

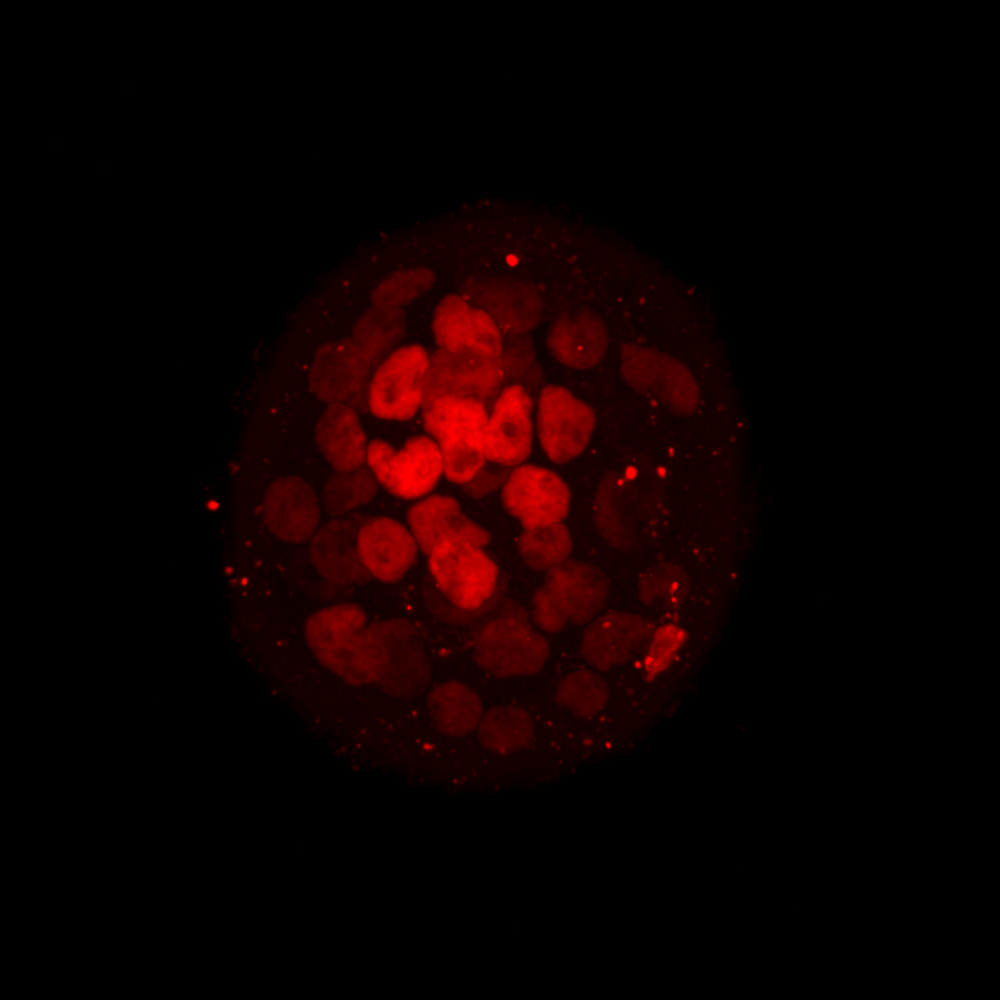

Supplement: Supplementary file 8 — Source data Fig. 6 [file 44319_2024_267_MOESM8_ESM.zip › Figure 6/6F/IF-WT-Oct3 4.tif]

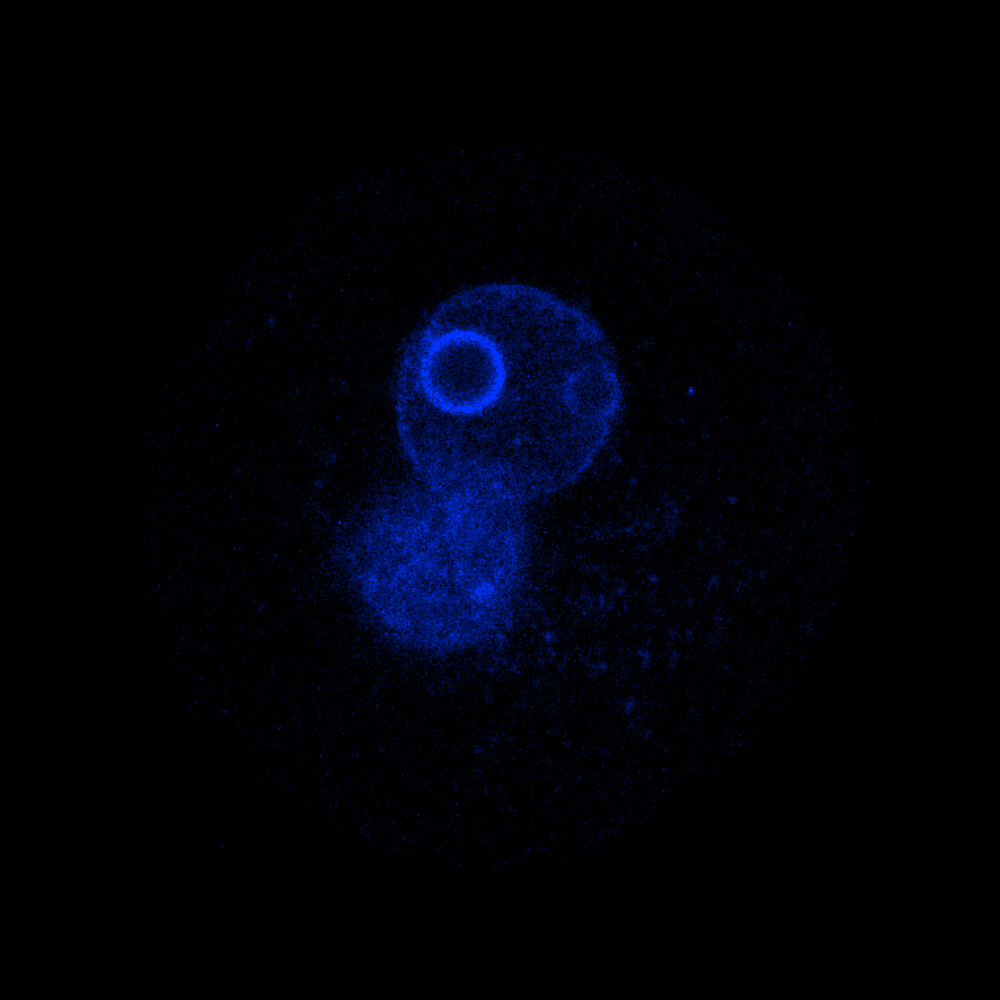

Supplement: Supplementary file 8 — Source data Fig. 6 [file 44319_2024_267_MOESM8_ESM.zip › Figure 6/6I/IF-DMSO-DAPI.tif]

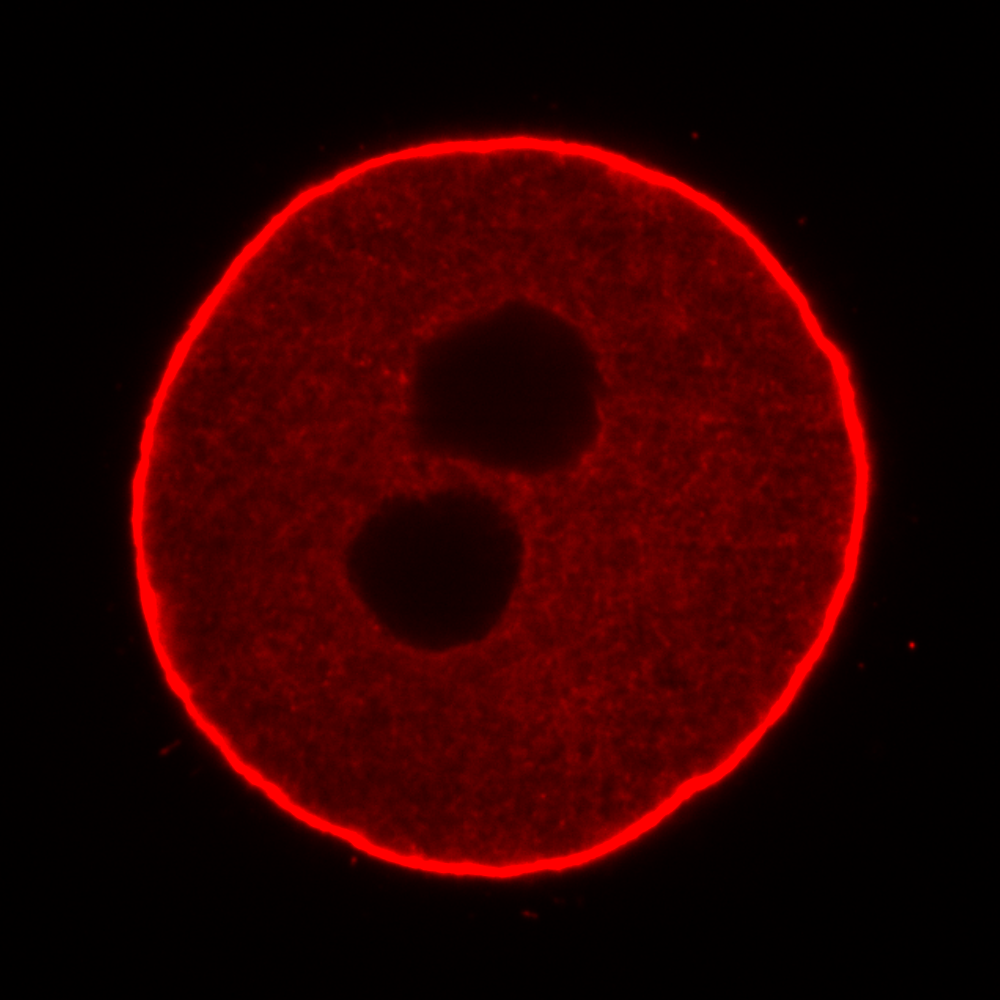

Supplement: Supplementary file 8 — Source data Fig. 6 [file 44319_2024_267_MOESM8_ESM.zip › Figure 6/6I/IF-DMSO-Factin.tif]

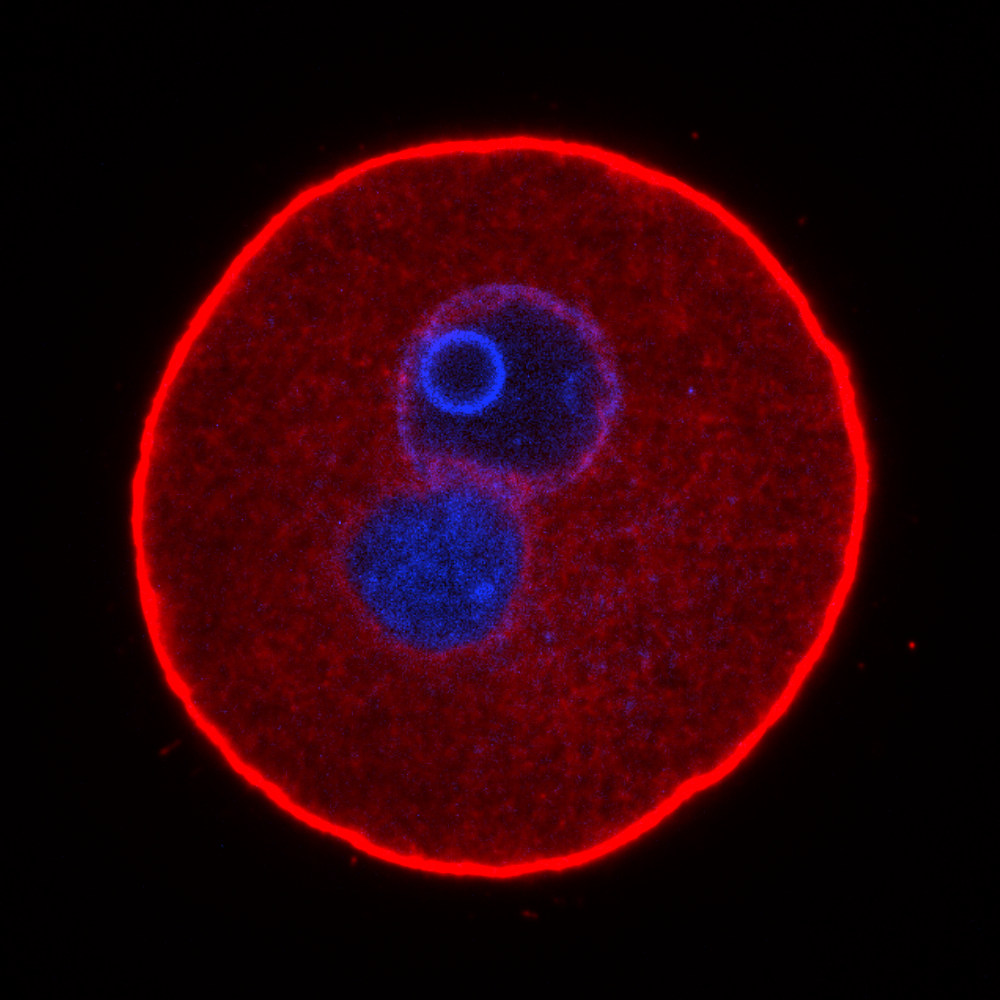

Supplement: Supplementary file 8 — Source data Fig. 6 [file 44319_2024_267_MOESM8_ESM.zip › Figure 6/6I/IF-DMSO-Merge.tif]

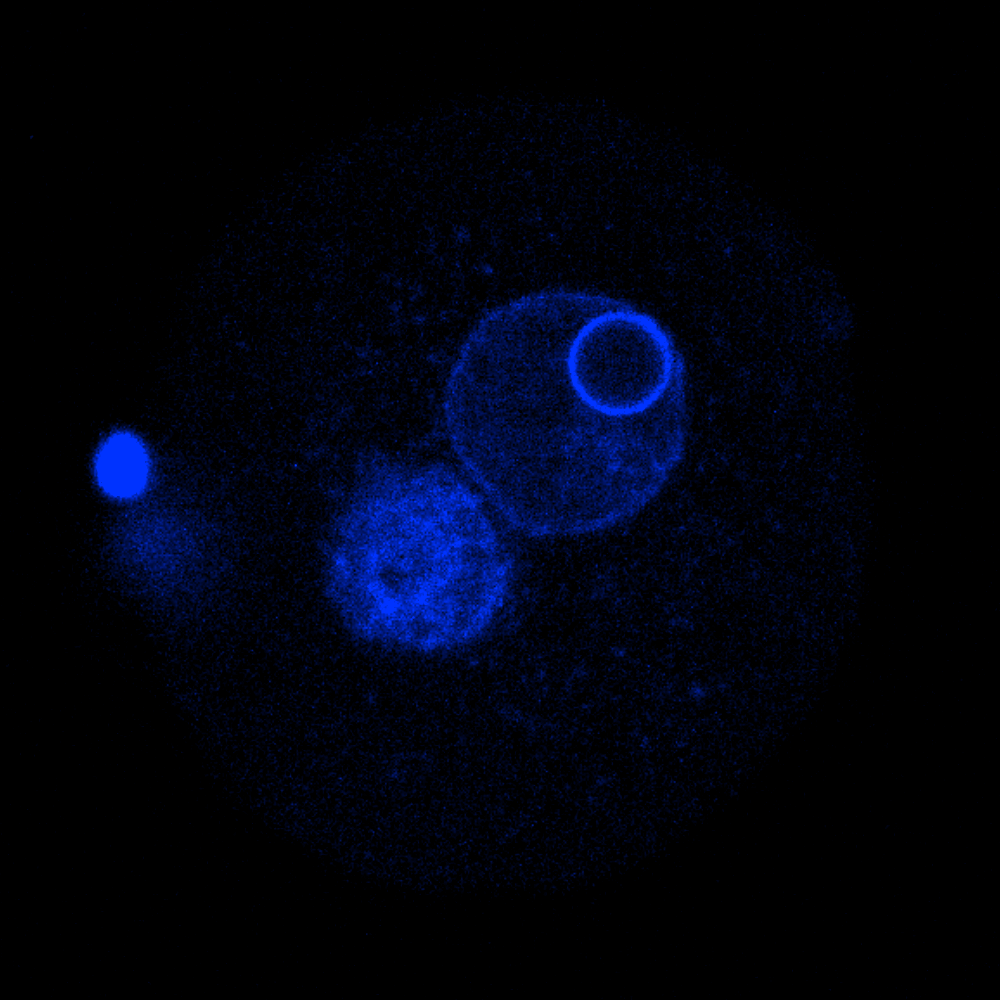

Supplement: Supplementary file 8 — Source data Fig. 6 [file 44319_2024_267_MOESM8_ESM.zip › Figure 6/6I/IF-EGCG-DAPI.tif]

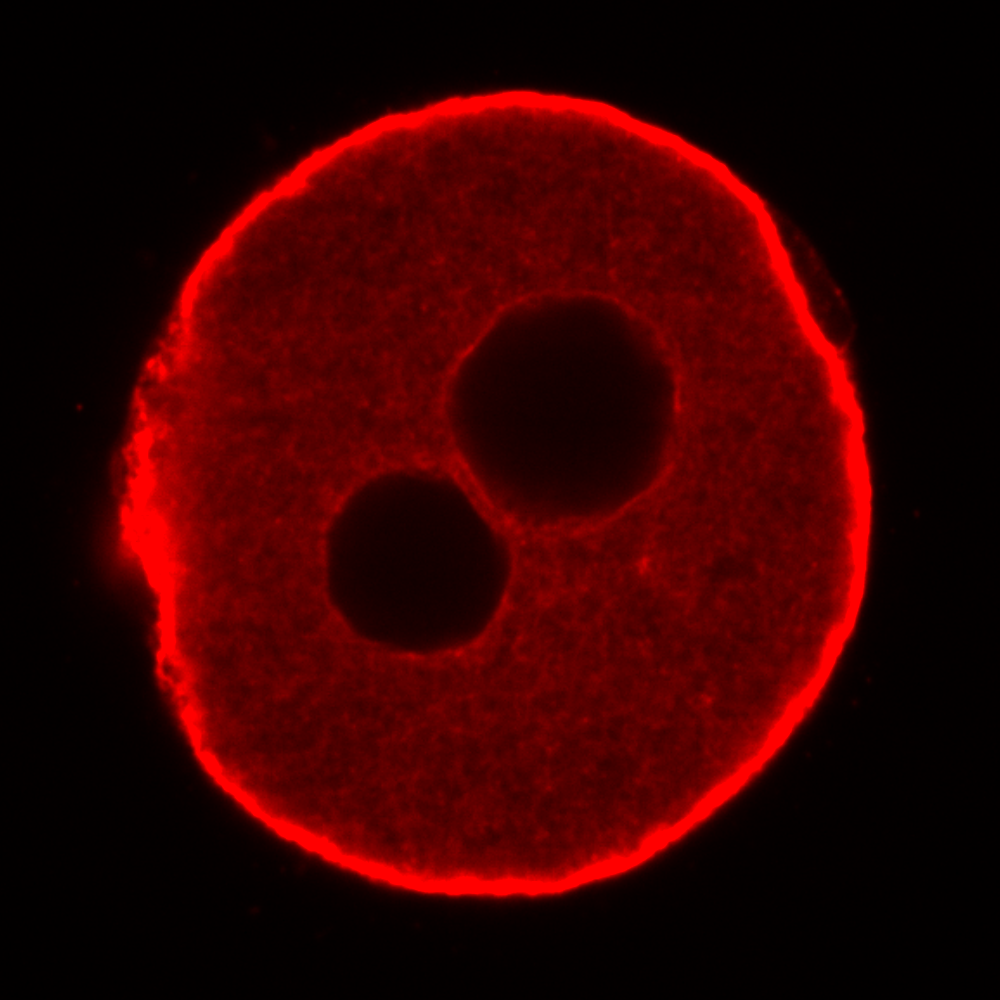

Supplement: Supplementary file 8 — Source data Fig. 6 [file 44319_2024_267_MOESM8_ESM.zip › Figure 6/6I/IF-EGCG-Factin.tif]

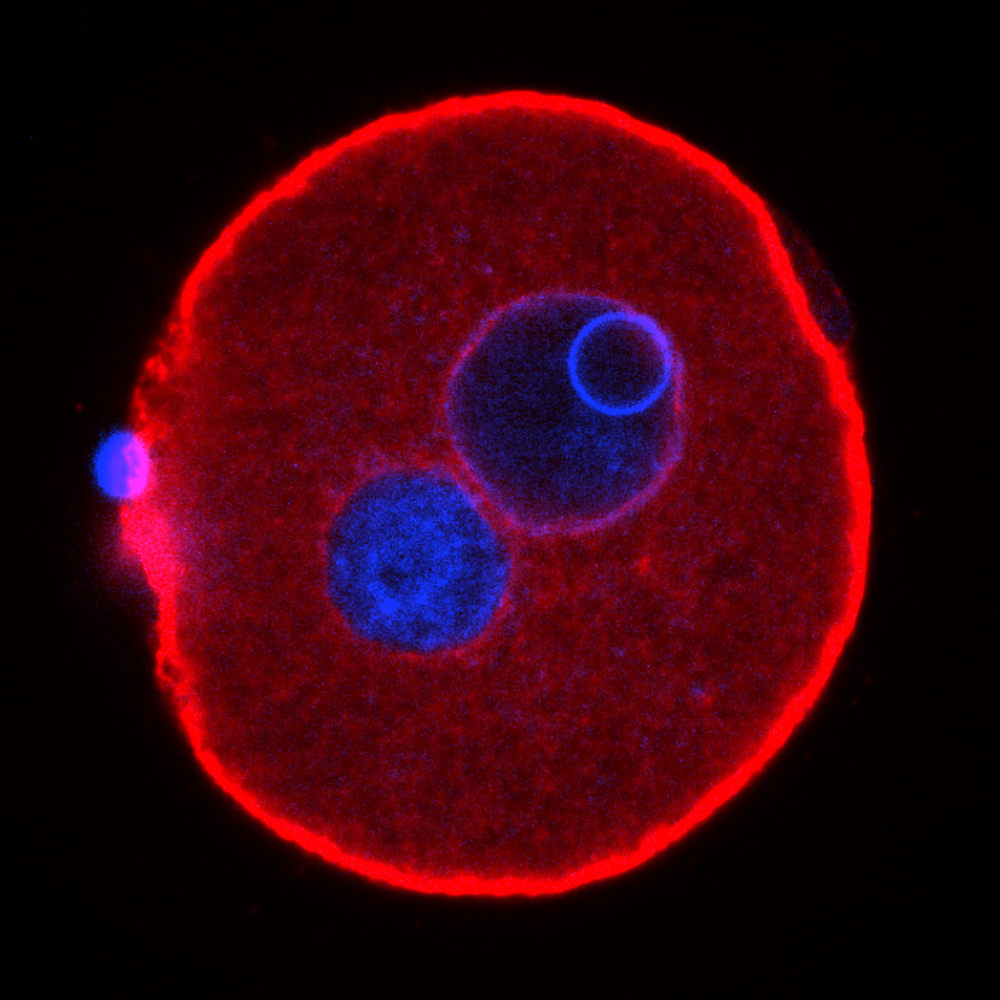

Supplement: Supplementary file 8 — Source data Fig. 6 [file 44319_2024_267_MOESM8_ESM.zip › Figure 6/6I/IF-EGCG-Merge.tif]

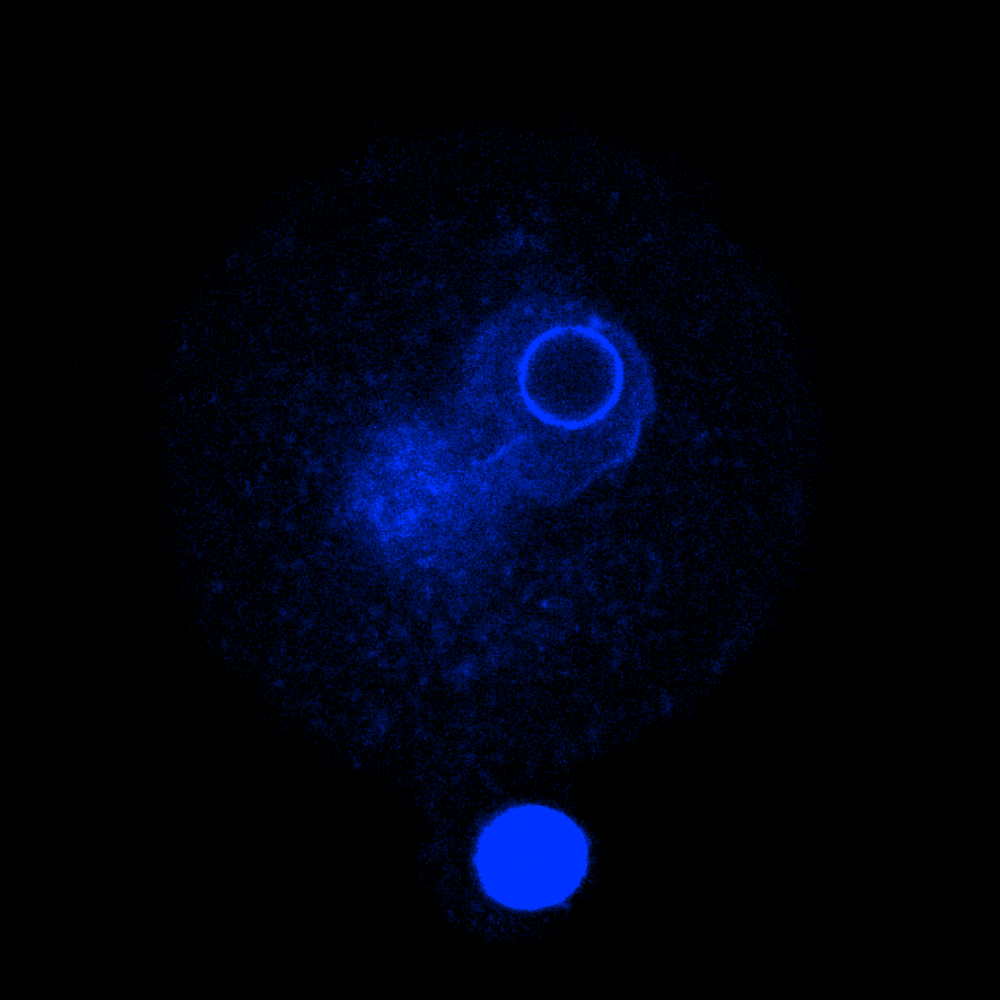

Supplement: Supplementary file 8 — Source data Fig. 6 [file 44319_2024_267_MOESM8_ESM.zip › Figure 6/6J/DMSO-DAPI.tif]

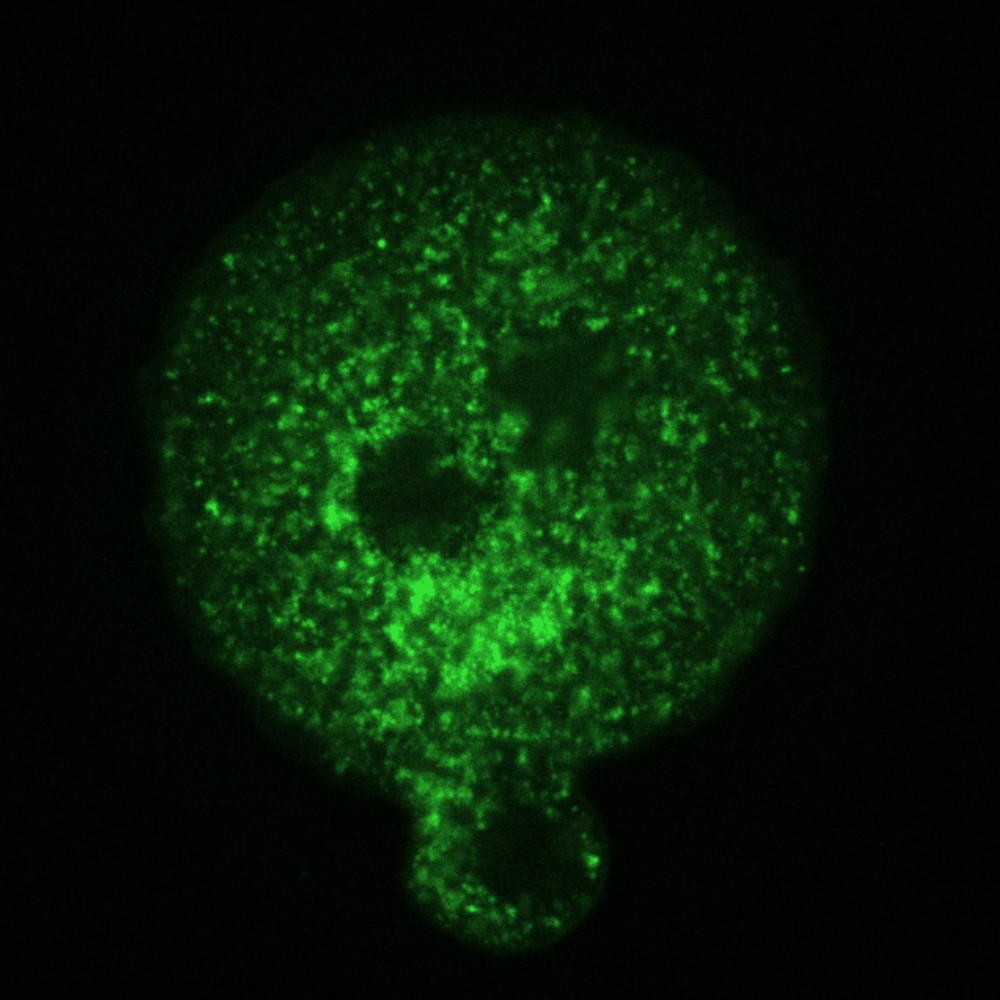

Supplement: Supplementary file 8 — Source data Fig. 6 [file 44319_2024_267_MOESM8_ESM.zip › Figure 6/6J/DMSO-H2O2.tif]

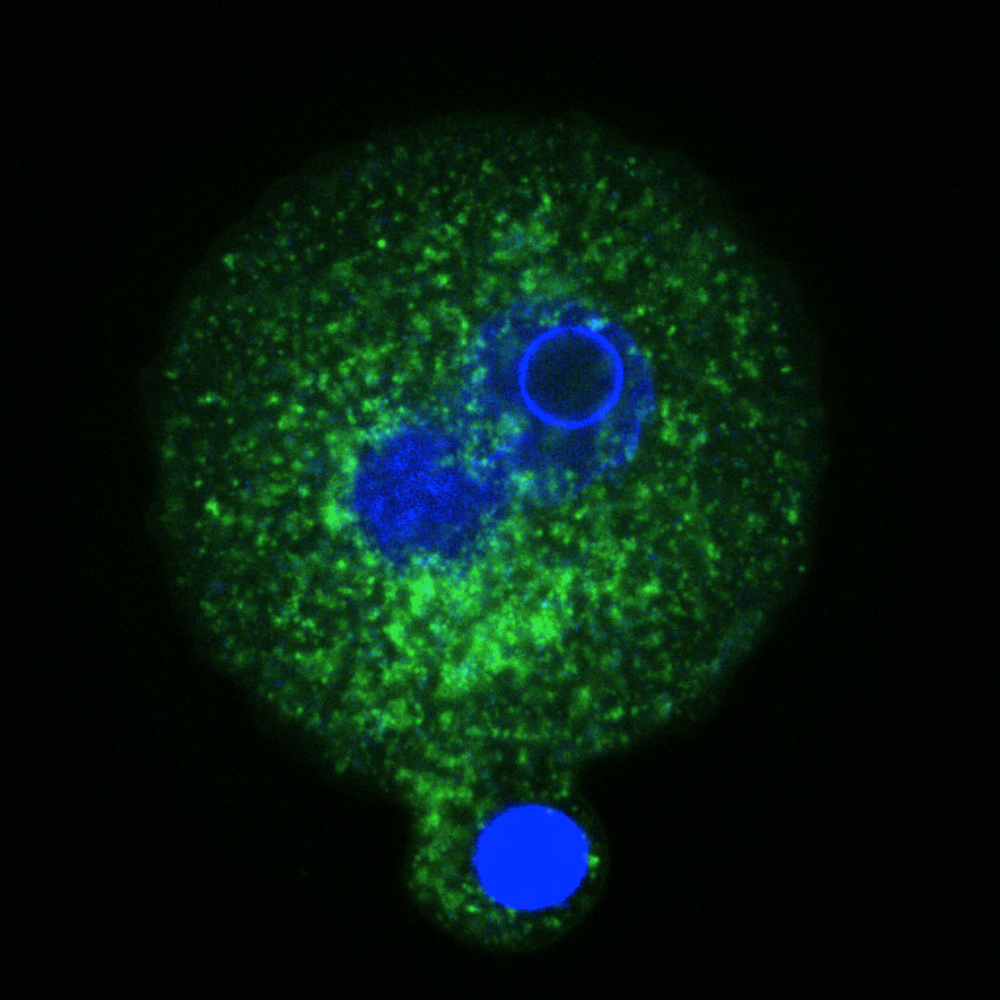

Supplement: Supplementary file 8 — Source data Fig. 6 [file 44319_2024_267_MOESM8_ESM.zip › Figure 6/6J/DMSO-Merge.tif]

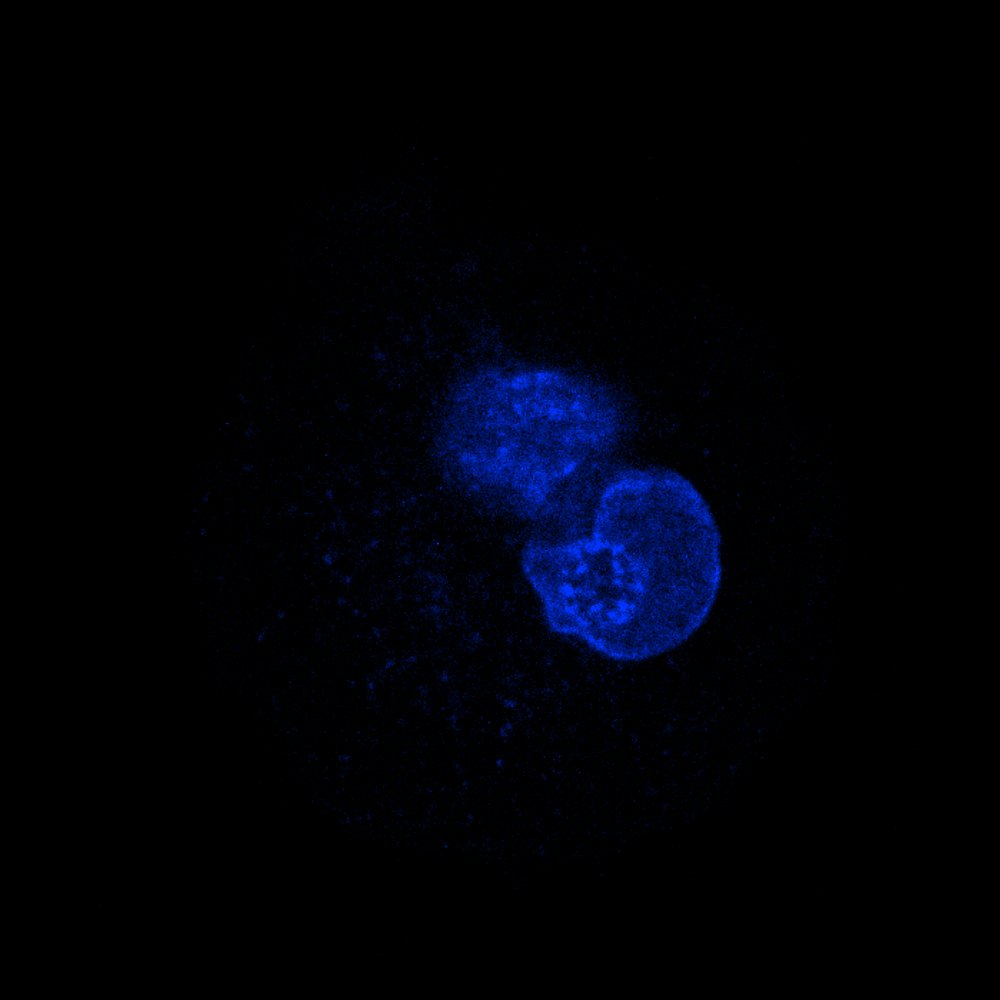

Supplement: Supplementary file 8 — Source data Fig. 6 [file 44319_2024_267_MOESM8_ESM.zip › Figure 6/6J/EGCG-DAPI.tif]

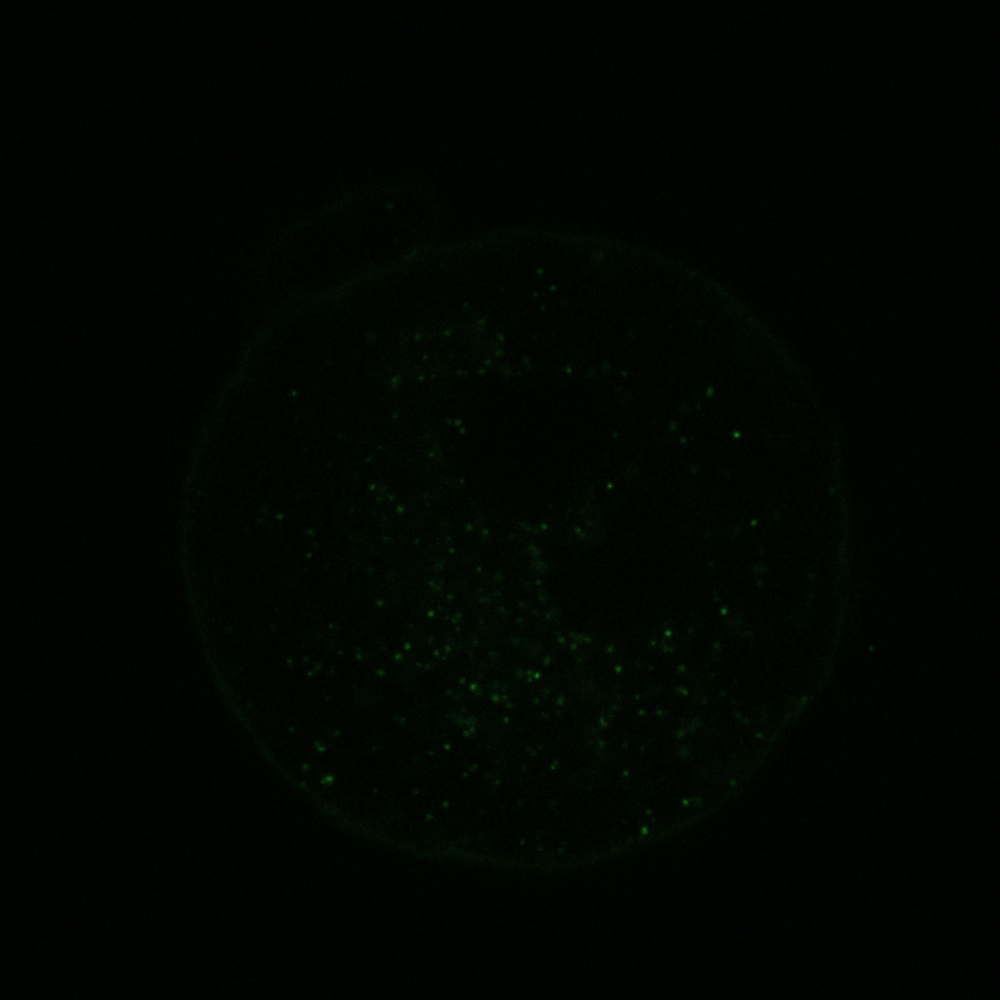

Supplement: Supplementary file 8 — Source data Fig. 6 [file 44319_2024_267_MOESM8_ESM.zip › Figure 6/6J/EGCG-H2O2.tif]

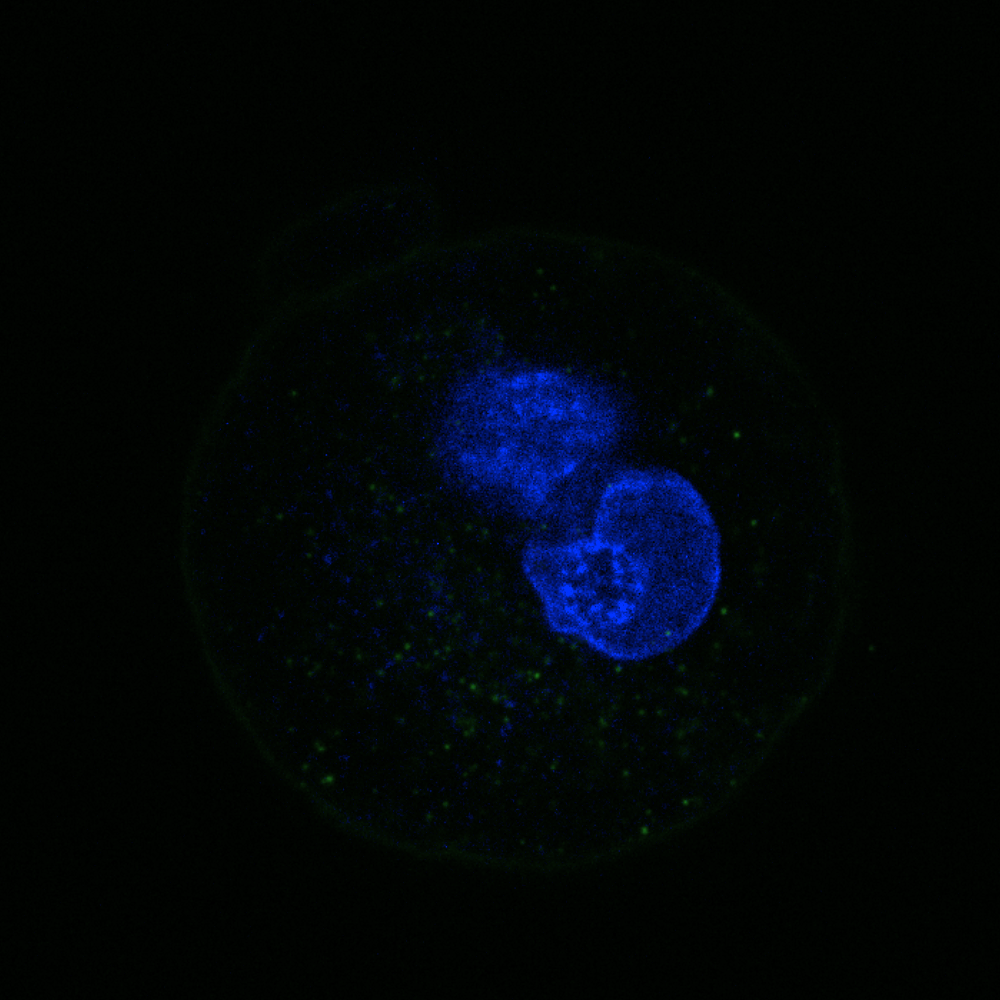

Supplement: Supplementary file 8 — Source data Fig. 6 [file 44319_2024_267_MOESM8_ESM.zip › Figure 6/6J/EGCG-Merge.tif]
