# Supplementary material for: CHK1 controls zygote pronuclear envelope breakdown by regulating F-actin through interacting with MICAL3
Source: EMBO Rep. 2024 Oct 2;25(11):4876–97. doi: 10.1038/s44319-024-00267-7 (PMC11549291; doi:10.1038/s44319-024-00267-7)
Supplement: Supplementary file 9 — Expanded View Figures [file 44319_2024_267_MOESM9_ESM.pdf]

Expanded View Figures

Figure EV1. Replacement of the mutant cytoplasm by pre-pronuclear transfer (PPNT).

(**A**) Time-lapse images reveal the embryos on the first day (Day 1) and the third day (Day 3) after fertilization. The control embryo without mutation is in 2-cell stage on Day 1 and in 8-cell stage on Day 3. While the embryos from two patients separately carrying CHK1 mutations p.R379Q and p.F441fs*16 are still in zygote stage with distinct pronuclei on Day 3. The white arrowheads indicate pronuclei. (**B**) Time-lapse images captured on Day 6 depict the reconstructed embryos. The upper panel displays embryos composed of the patient's female pronucleus (PN) and the donor's enucleated cytoplasm (CP), while the lower panel shows embryos with the donor's pronucleus (PN) and the patient's cytoplasm (CP). (**C**) A diagram illustrates the process of pre-pronuclear transfer (PPNT) conducted between zygotes obtained from a patient with CHK1 mutation (p.R379Q) and a healthy donor. Mature oocytes (MII) are collected from both the patient and the donor, and then subjected to intracytoplasmic sperm injection (ICSI) for fertilization. The first polar bodies (PB1) formed after fertilization are removed. After the second polar bodies (PB2) have been naturally extruded, the female pre-pronuclei (PPN) along with PB2 are isolated and dissociated from both the patient's and donor's zygotes. The PPN from the patient's zygote is transferred into the perivitelline space (PVS) of an enucleated oocyte from the donor. This reconstructed oocyte develops into a zygote containing two pronuclei. Simultaneously, the female PPN from the donor's zygote is injected into the patient's oocyte PVS using the same method. This leads to the formation of a zygote with two pronuclei in the patient's oocyte. (**D**) Quantitative analysis of pluripotency marker signals between the reconstructed stem cell line (ESC_Em-1) and the stem cell line (ESC_PF-1) treated with the inhibitor in (**E**). At least three representative images were employed for the quantitation. Error bars, SEM. (**E**) Representative images showing pluripotency markers of an embryonic stem cell line derived from the patient's blastocyst after treatment with a CHK1 inhibitor. Scale bar: 100 μm. (**F**) A diagram indicates the application of ICM (inner cell mass) and TE (trophoblast) of the treated blastocysts. (**G**) Sanger sequencing chromatograms of Em-1 in both forward and reverse sequencing direction demonstrate wild-type (WT) genotype.

 © The Author(s)

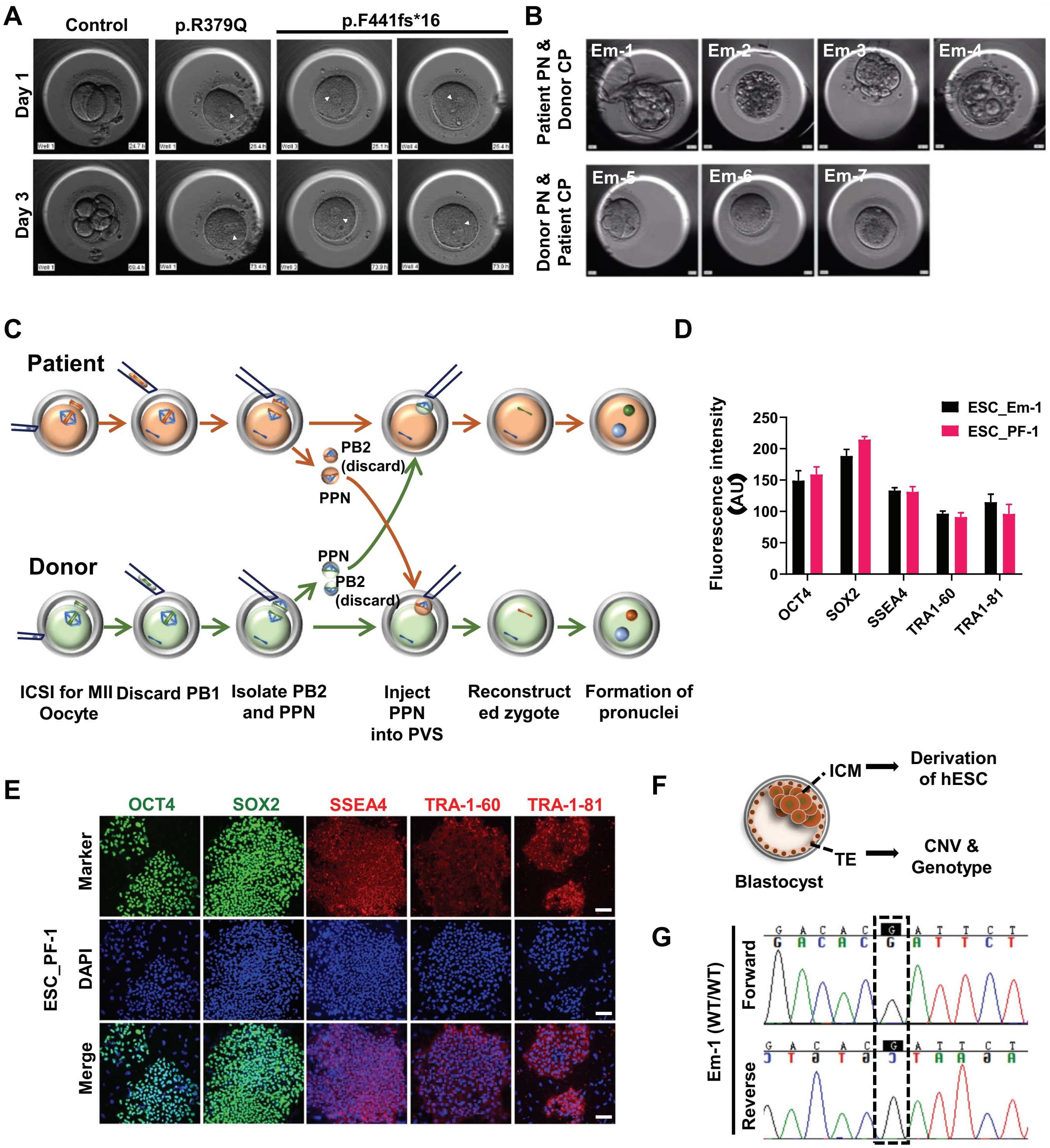
A
Control
p.R379Q
p.F441fs*16
Day 1
Day 3
B
Patient PN & Donor CP
Donor PN & Patient CP
Em-1
Em-2
Em-3
Em-4
Em-5
Em-6
Em-7
C
Patient
Donor
PB2 (discard)
PPN
ICSI for MII Oocyte
Discard PB1
Isolate PB2 and PPN
Inject PPN into PVS
Reconstructed zygote
Formation of pronuclei
D
Fluorescence intensity (AU)
ESC_Em-1
ESC_PF-1
OCT4
SOX2
SSEA4
TRA1-60
TRA1-81
E
OCT4
SOX2
SSEA4
TRA-1-60
TRA-1-81
ESC_PF-1
Marker
DAPI
Merge
F
ICM
Derivation of hESC
TE
CNV & Genotype
Blastocyst
G
Em-1 (WT/WT)
Forward
Reverse

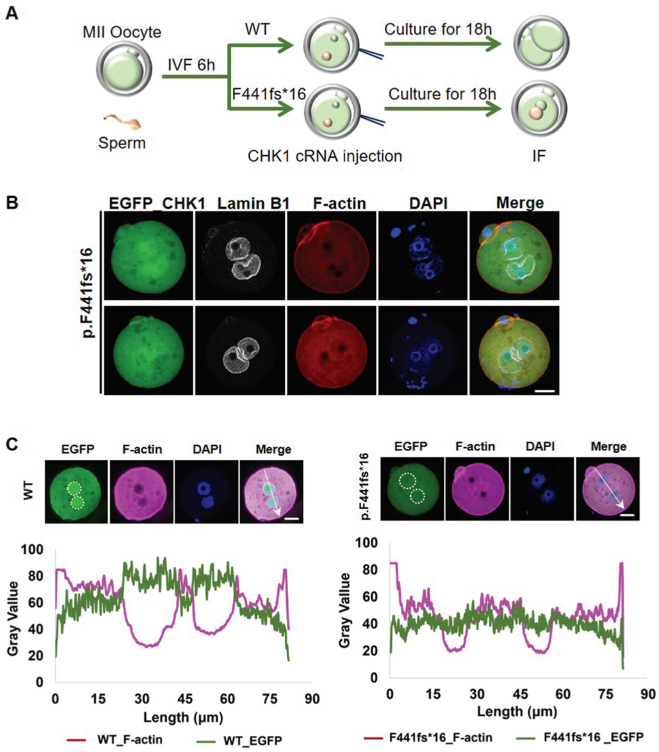

Figure EV2. Zygotes overexpressing mutant CHK1 (p.F441fs*16) arrest with clear pronuclear member.

(**A**) A diagram reveals the cRNA injection procedure in zygotes. Six hours after in vitro fertilization (IVF), the zygotes with clear pronuclei were injected with wild-type (WT) or mutated (p.F441fs*16) CHK1 cRNA, followed by being cultured for 18 h to perform immunofluorescence (IF) staining. (**B**) The IF result shows that the arrested zygotes with CHK1 mutation (p.F441fs*16) have obvious pronuclear member indicated by the Lamin B1 antibody. Scale bar: 20 μm. (**C**) Line profiles were generated across zygotes, encompassing regions that spanned both the pronuclei and cortex. These profiles vividly illustrate the spatial distribution of F-actin and EGFP signal in both wild-type and mutant zygotes. White dashed circles in zygotes indicate the pronuclei, while the white arrowheads reveal the line profiles below. Scale bar: 20 μm. The images used in Fig. EV2C are derived from Fig. 2C for the signal quantification.

 © The Author(s)

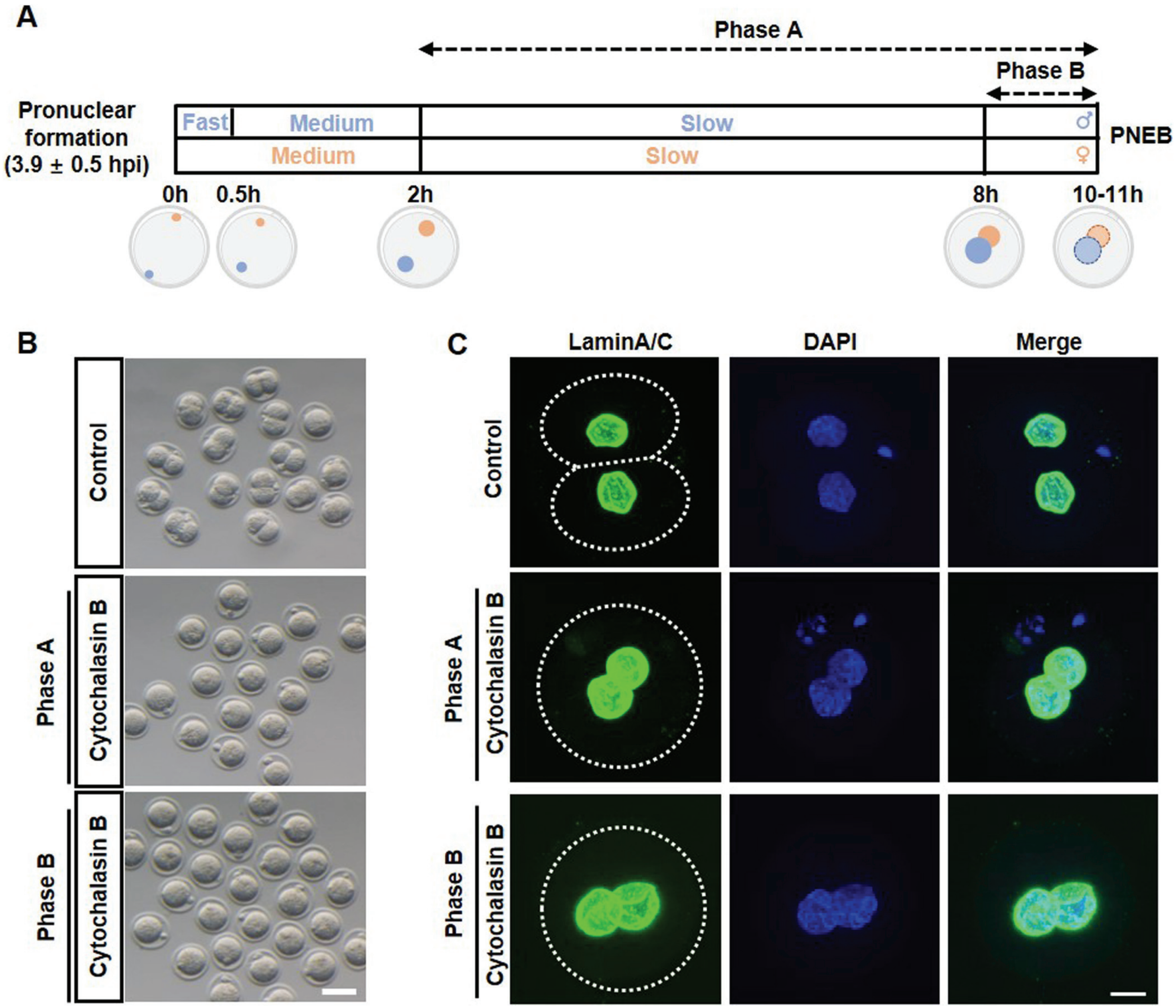

Figure EV3. Inhibiting F-actin can disturb the PNEB event.

(**A**) The provided diagram outlines critical time points subsequent to pronuclei formation, which typically occurs around 3.9 ± 0.5 h post insemination (hpi). Notably, the male pronucleus experiences fast, medium and slow migration phases, while the female pronucleus undergoes medium and slow migration stages, culminating in pronuclear envelope breakdown (PNEB). (**B**) Captured images depict the developmental outcomes of zygotes across various groups subjected to cytochalasin B treatment during phase A and phase B. The scale bar corresponds to 100 µm. (**C**) Immunofluorescence results from the embryos depicted in (**B**) are presented, showcasing specific markers. Scale bar: 20 µm.

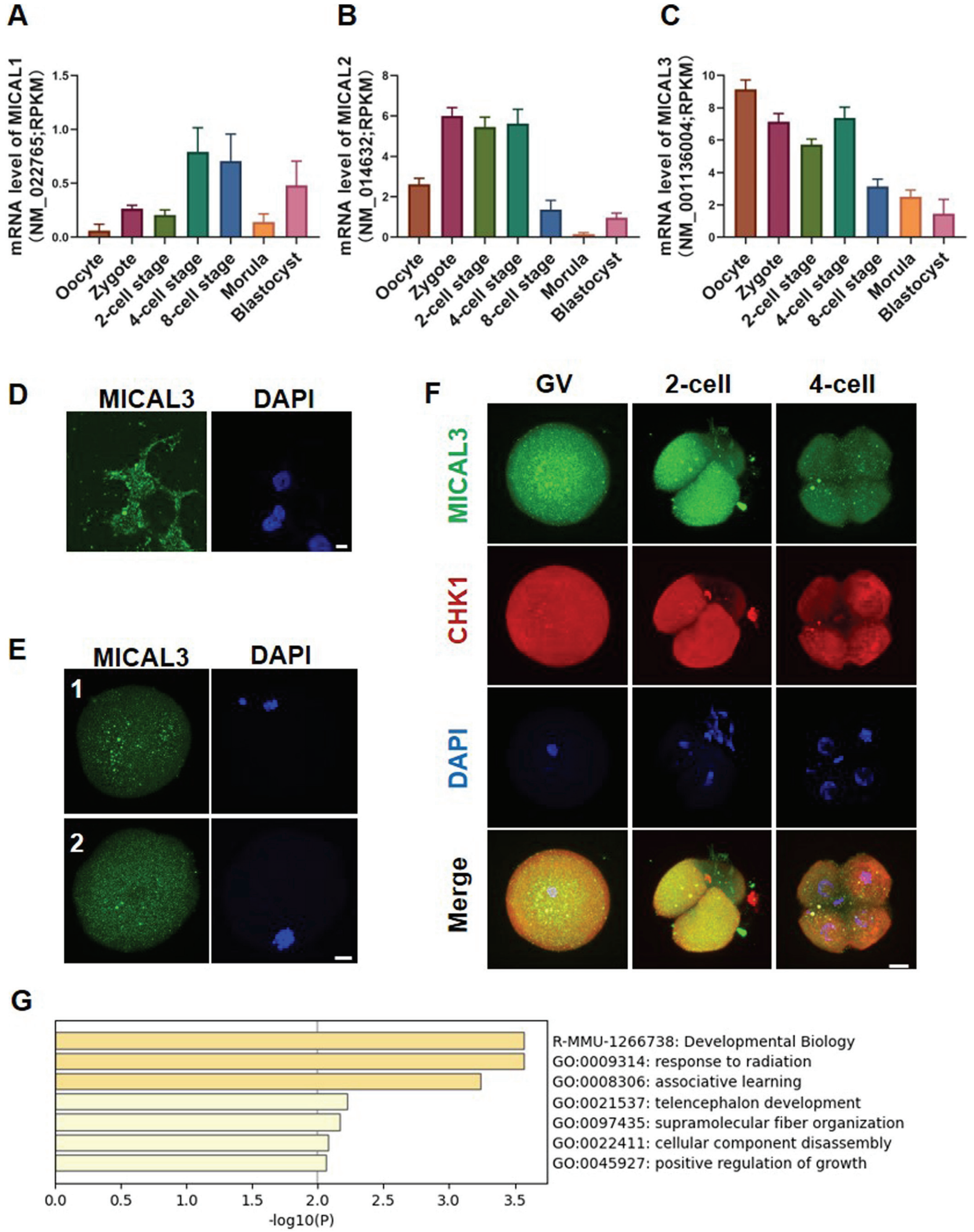
A
mRNA level of MICAL1 (NM_022765;RPKM)
Oocyte
Zygote
2-cell stage
4-cell stage
8-cell stage
Morula
Blastocyst
B
mRNA level of MICAL2 (NM_014632;RPKM)
C
mRNA level of MICAL3 (NM_001136004;RPKM)
D
MICAL3
DAPI
E
1
2
F
GV
2-cell
4-cell
CHK1
Merge
G
R-MMU-1266738: Developmental Biology
GO:0009314: response to radiation
GO:0008306: associative learning
GO:0021537: telencephalon development
GO:0097435: supramolecular fiber organization
GO:0022411: cellular component disassembly
GO:0045927: positive regulation of growth
-log10(P)

Figure EV4. The expression and localization of human MICAL3 protein.

(**A–C**) The mRNA expression levels of MICAL family members (MICAL1/MICAL2/MICAL3) in human oocytes and preimplantation embryos according to the published transcriptome data (Yan et al, 2013; Data ref: Yan et al, 2013). The data included at least three oocyte or embryo replication in each stage. Error bars, SEM. (**D**) The localization of MICAL3 in HEK-293 cells. Scale bars: 20 µm. (**E**) Human oocytes immunofluorescence staining result shows that MICAL3 mainly localizes in cytoplasm. Scale bars: 20 µm. (**F**) Immunofluorescence staining of both MICAL3 and CHK1 in the human GV oocyte, 2-cell and 4-cell embryos. Of note, the oocyte and embryos were reused after completing proximity ligation assay and the experiments in (**E**, **F**) were performed two times because of the rarity of human oocytes. Scale bars: 20 µm. (**G**) Bar graph of enriched terms across proteins identified as potentially interacting with CHK1 in Fig. 4A, colored by *P* values and analyzed by Metascape.

© The Author(s)

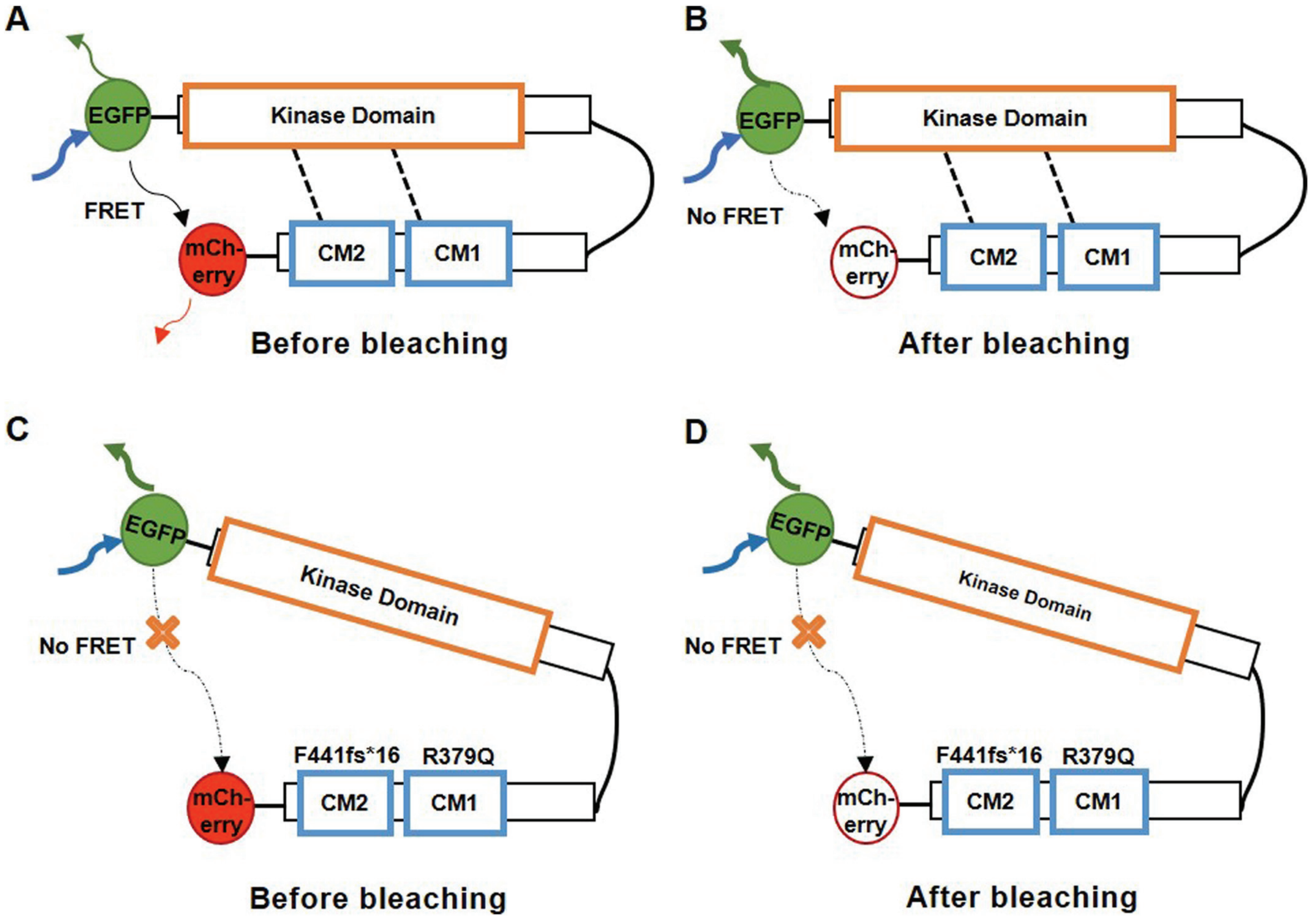

Figure EV5. Diagrams depict the principle of single-molecular fluorescence resonance energy transfer (smFRET).

The CHK1 protein composed of a N-terminal kinase domain and a C-terminal domain with two conserved motifs (CM1 and CM2). For smFRET, CHK1 protein was fused with enhanced green fluorescence protein (EGFP) and red mCherry protein separately in the two terminals. (**A**) Wild-type CHK1 keeps in a "closed" state with interaction between the N-terminal and C-terminal domains. When the two terminal domains are closed enough, the EGFP signal is excited and some of its emission light will be transferred to excite mCherry signal, resulting in reduced EGFP emission signal. (**B**) After bleaching mCherry, the EGFP emission signal can't be transferred to mCherry and thus will be enhanced. (**C**) The N-terminal and C-terminal domains of mutant CHK1 hold an "open" conformation and their distance is not that closed to generate FRET. (**D**) Even though bleaching mCherry, the EGFP emission signal is not disturbed. The blue curves with arrowhead indicate excitation light of EGFP. Green curves with arrowhead show emission light of EGFP. The black curve with arrowhead denotes part of the EGFP emission signal, indicating the happen of smFRET. The dotted black lines with arrowhead indicate no FRET phenomenon.

 © The Author(s)
